# Predicting molecular mechanisms of hereditary diseases by using their tissue‐selective manifestation

**DOI:** 10.15252/msb.202211407

**Published:** 2023-05-26

**Authors:** Eyal Simonovsky, Moran Sharon, Maya Ziv, Omry Mauer, Idan Hekselman, Juman Jubran, Ekaterina Vinogradov, Chanan M Argov, Omer Basha, Lior Kerber, Yuval Yogev, Ayellet V Segrè, Hae Kyung Im, GTEx Consortium, Ohad Birk, Lior Rokach, Esti Yeger‐Lotem

**Affiliations:** ^1^ Department of Clinical Biochemistry and Pharmacology Ben‐Gurion University of the Negev Beer Sheva Israel; ^2^ Morris Kahn Laboratory of Human Genetics and the Genetics Institute at Soroka Medical Center, Faculty of Health Sciences Ben Gurion University of the Negev Beer Sheva Israel; ^3^ Ocular Genomics Institute, Massachusetts Eye and Ear Harvard Medical School Boston MA USA; ^4^ The Broad Institute of MIT and Harvard Cambridge MA USA; ^5^ Section of Genetic Medicine, Department of Medicine The University of Chicago Chicago IL USA; ^6^ The National Institute for Biotechnology in the Negev Ben‐Gurion University of the Negev Beer Sheva Israel; ^7^ Department of Software & Information Systems Engineering Ben‐Gurion University of the Negev Beer Sheva Israel

**Keywords:** data integration, genomic medicine, machine learning, omics, tissue selectivity, Computational Biology, Genetics, Gene Therapy & Genetic Disease, Methods & Resources

## Abstract

How do aberrations in widely expressed genes lead to tissue‐selective hereditary diseases? Previous attempts to answer this question were limited to testing a few candidate mechanisms. To answer this question at a larger scale, we developed “Tissue Risk Assessment of Causality by Expression” (TRACE), a machine learning approach to predict genes that underlie tissue‐selective diseases and selectivity‐related features. TRACE utilized 4,744 biologically interpretable tissue‐specific gene features that were inferred from heterogeneous omics datasets. Application of TRACE to 1,031 disease genes uncovered known and novel selectivity‐related features, the most common of which was previously overlooked. Next, we created a catalog of tissue‐associated risks for 18,927 protein‐coding genes (https://netbio.bgu.ac.il/trace/). As proof‐of‐concept, we prioritized candidate disease genes identified in 48 rare‐disease patients. TRACE ranked the verified disease gene among the patient's candidate genes significantly better than gene prioritization methods that rank by gene constraint or tissue expression. Thus, tissue selectivity combined with machine learning enhances genetic and clinical understanding of hereditary diseases.

## Graphical abstract

An interpretable machine‐learning framework predicts disease genes for tissue‐selective hereditary diseases. The framework highlights known and novel tissue‐selectivity features and enhances genetic diagnosis of rare‐disease patients.

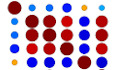

## Introduction

Genetic and clinical studies of Mendelian and rare heritable diseases strive to identify pathogenic variants (Eilbeck *et al*, 2017) and their functional effects (Hekselman & Yeger‐Lotem, 2020) while facing multiple challenges. Exome and whole‐genome sequencing of patients typically yield thousands of variants of unknown significance (McCarthy & MacArthur, 2017). To pinpoint the pathogenic variant among them, variants are scrutinized via multiple strategies, including sequence and conservation analyses (Adzhubei *et al*, 2013; Gelfman *et al*, 2017; Rentzsch *et al*, 2019), mutational constraints (Karczewski *et al*, 2020), clinical variants databases (Landrum *et al*, 2016), and similarity in function or phenotype between candidate genes and disease‐associated genes (Aerts *et al*, 2006; Kumar *et al*, 2018; Deelen *et al*, 2019). Nevertheless, the rate of successful genetic diagnostic of patients stands on 25–60% (Chong *et al*, 2015; 100,000 Genomes Project Pilot Investigators *et al*, 2021), calling for additional strategies.

When a pathogenic variant is identified, the molecular mechanisms by which it leads to disease phenotypes typically remain elusive. Attesting to the complexity of this challenge, molecular mechanisms have remained hidden even for long‐known and well‐established pathogenic variants (Moaven *et al*, 2015; Hernandez *et al*, 2016; Goedert *et al*, 2017; Holmans *et al*, 2017; Huttlin *et al*, 2017). Here, we focused on the large set of Mendelian diseases that mainly affect few selected tissues, such as neurodegenerative disorders, skin diseases, or muscular dystrophies (Hekselman & Yeger‐Lotem, 2020). The molecular mechanisms that underlie tissue‐selective Mendelian diseases are especially intriguing, as often the genes that harbor pathogenic variants (denoted disease genes) are expressed ubiquitously across the human body (Hekselman & Yeger‐Lotem, 2020). For example, familial mutations in the gene dystroglycan 1 (DAG1) cause a rare inherited neuromuscular disorder. Likewise, familial mutations in the gene BRCA1 increase the risk for breast and ovarian cancers. Contrary to their tissue‐selective disease manifestations, DAG1 and BRCA1 are expressed in many tissues (GTEx Consortium, 2020). Notably, knowledge of disease‐affected tissues can help pinpoint pathogenic variants and their mode of action. For example, genetic diagnosis of patients with rare muscle disorders and interpretation of genetic variants were aided by information on transcripts expression in normal skeletal muscle and other tissues, respectively (Cummings *et al*, 2017, 2020).

Some of the efforts to illuminate disease mechanisms utilized the immense molecular characterization of tens of physiological human tissues, including tissue transcriptomes (GTEx Consortium, 2020), proteomes (Uhlen *et al*, 2015), and regulatory and epigenetic signals (Davis *et al*, 2018). Tissue omics datasets, especially tissue transcriptomes, were used for rare variant interpretation (Cummings *et al*, 2017, 2020), and for prioritizing candidate disease genes by their similarity to known disease‐related genes, whereby similarity was based on attributes such as protein interactors or associated Human Phenotype Ontology (HPO) terms, in tools such as Endeavor (Aerts *et al*, 2006), Exomiser (Smedley *et al*, 2015), pBRIT (Kumar *et al*, 2018), and GADO (Deelen *et al*, 2019).

In parallel, studies of tissue‐selective Mendelian diseases revealed various tissue‐based features of disease genes, such as their tendencies for preferential expression (Lage *et al*, 2008; Barshir *et al*, 2014), molecular interactions (Magger *et al*, 2012; Barshir *et al*, 2014; Greene *et al*, 2015; Malod‐Dognin *et al*, 2019; Basha *et al*, 2020), and dosage imbalance with modifier genes (Barshir *et al*, 2018; Jubran *et al*, 2020) in normal samples of disease‐affected tissues. Likewise, studies of tissue‐selective complex traits revealed tendencies of trait‐associated genes to disrupt tissue‐specific regulatory relationships (Marbach *et al*, 2016) and gene modules (Kitsak *et al*, 2016), or to overlap with active eQTLs (Barbeira *et al*, 2018; Gamazon *et al*, 2018, 2019) in normal samples of trait‐manifesting tissues. Yet, these studies and others were limited to testing few candidate features.

Machine learning (ML) methods have been used widely in various biological contexts (Wong *et al*, 2021). Compared with traditional models such as multivariable regression models, ML can handle a larger number of features and consider not‐predetermined and complex interactions between features (including nonlinear relations), thereby providing improved predictive performance. Additionally, ML can handle large amounts of data and automatically select the most relevant features, thereby supporting versatility and scalability. Prominent ML methods include deep learning and decision trees, the latter often preferred when training data are relatively limited and the interpretability of the resulting model is important. In tissue‐specific contexts, deep learning was used to infer gene modules and variants for complex diseases (Dwivedi *et al*, 2020; Wesolowska‐Andersen *et al*, 2020) and to predict cancer dependency of tumors (Chiu *et al*, 2021). Nonnegative matrix tri‐factorization was used to identify interactome‐rewired genes in tissue‐specific cancers (Malod‐Dognin *et al*, 2019). Recently, decision trees were used to prioritize tissue‐relevant genes, though the resulting model had limited interpretability (Somepalli *et al*, 2021). The latter method used supervised classification, where the goal is to train a classifier that maps an input to a predefined set of classes and accurately generalizes to unlabeled cases.

We hypothesized that ML can be used to dramatically expand the mechanistic understanding of tissue‐selective diseases by (i) prioritizing candidate disease genes in tissue contexts and (ii) assessing candidate tissue‐selectivity features. Our approach, termed “Tissue Risk Assessment of Causality by Expression” (TRACE), was designed as a supervised and interpretable ML framework. TRACE was implemented as an early integration ML scheme that utilized 4,744 tissue‐based gene features, which were derived and combined from heterogeneous omics sources. The large variety of gene features greatly exceeded that of previous methods (Somepalli *et al*, 2021), enhancing TRACE interpretability. We trained and tested TRACE on 18,927 protein‐coding genes, including 1,031 disease genes that underlie tissue‐selective Mendelian diseases, which manifest in eight main tissues. Application of TRACE to specific diseases or jointly to diseases that manifest in the same tissue revealed known tissue‐selectivity features, as well as commonly occurring yet previously underexplored features, such as the preferential activity of specific cellular processes (Sharon *et al*, 2022). Next, we harnessed TRACE to create a catalog of tissue‐specific risks for 18,927 human protein‐coding genes (https://netbio.bgu.ac.il/trace/). As proof‐of‐concept, we applied TRACE to prioritize candidate disease genes of patients with rare tissue‐selective Mendelian diseases. Even though no sequence‐based features were used, in 34% of the cases TRACE successfully prioritized the verified pathogenic gene among the top 10% of the patient's candidate genes. Thus, tissue‐aware ML schemes can boost genetic and clinical studies of tissue‐selective Mendelian diseases.

## Results

### Constructing tissue‐based features and dataset for ML

**Figure 1:**
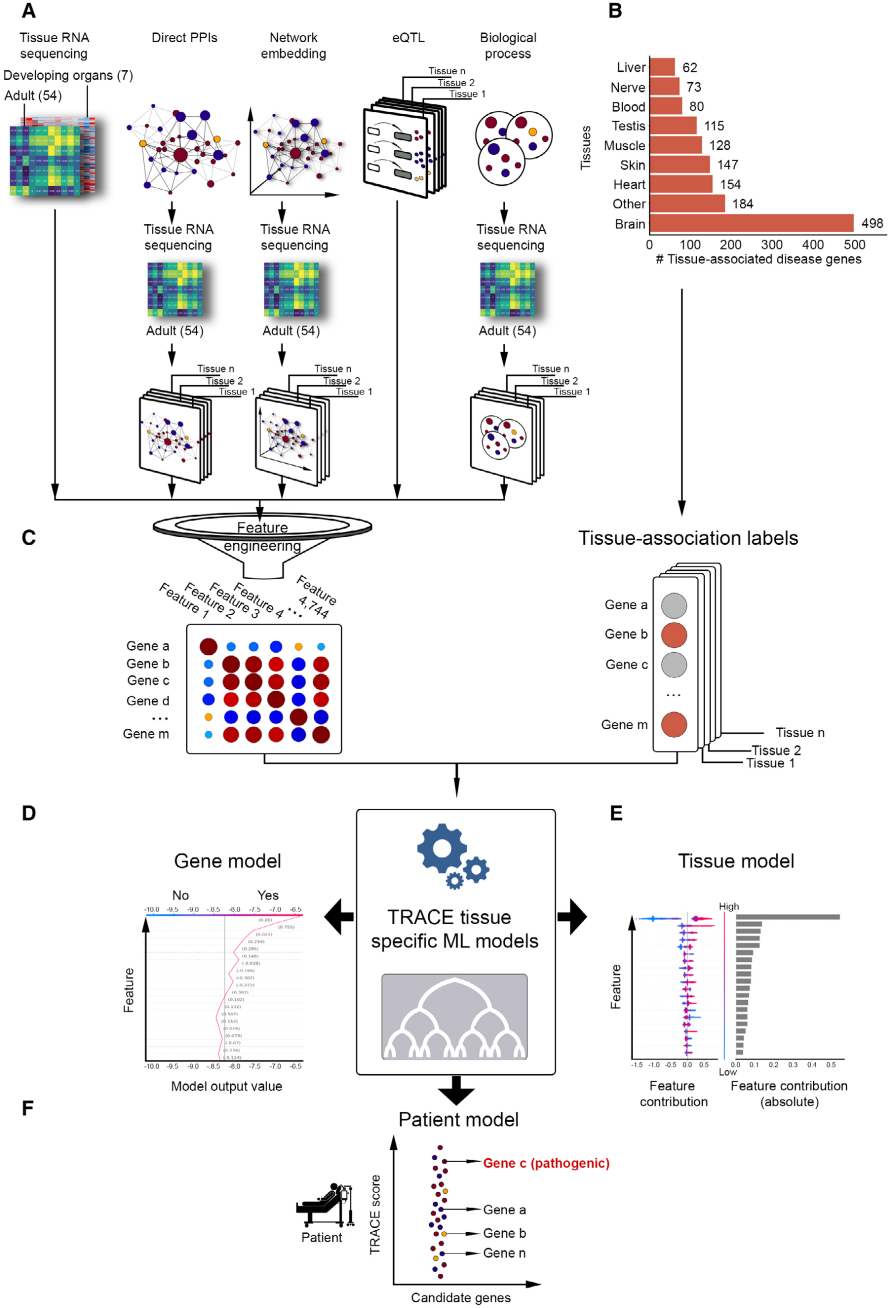
ML scheme for interpreting tissue‐selective disease mechanisms Construction of the tissue‐based gene features dataset used in the analysis. Features were derived from transcriptomes of 54 human adult tissues and seven developing organs, PPIs, tissue eQTLs and gene annotations to biological processes. Datasets were integrated and interpretable features were engineered, resulting in 4,744 features for 18,927 protein‐coding genes.The number of tissue‐associated disease genes, which underlie a Mendelian disease that manifests mainly in the designated tissue or in other tissues (marked “Other”).The input to TRACE included the dataset of tissue‐based gene features and the tissue‐association gene labels for the modeled tissue. Red labels mark tissue‐associated disease genes. Various outputs of the models are described in panels (D–F).Buildup of the output value for the predicted tissue association of a query gene in the modeled tissue. Starting with a neutral value at the bottom, the output value accumulates according to the feature values of the query gene. Features are ordered from bottom to top by their increased contribution to the model, allowing interpretation. The location of the final output value on the *X* axis indicates whether the query gene is predicted to be a tissue‐associated disease gene (yes, right) or not (no, left) in the modeled tissue (the gray vertical bar denotes the baseline value).A quantitative view of the contribution of features to a TRACE tissue model. Features are ordered from bottom to top by their increased absolute contribution to the model (gray bars), allowing interpretation. Per feature, each dot represents the feature value of a different gene (red and blue denote high and low values of the feature, respectively). Dots are spread from left to right by their contribution to disease manifestation in the modeled tissue.TRACE prioritization of candidate disease genes of a patient. TRACE scores candidate genes by the likelihood that they underlie a disease that manifests in the patient's affected tissue. Gene C represents the verified disease gene of that patient.

We constructed a large‐scale dataset consisting of engineered (interpretable) and abstract (performance‐boosting) tissue‐based features per protein‐coding gene (Fig 1A and Appendix Table S1). Tissue‐based features were motivated by studies of tissue‐selective traits and diseases and were derived from diverse data sources, predominantly transcriptomes of adult (GTEx Consortium, 2020) and developing (Cardoso‐Moreira *et al*, 2019) physiological human tissues, as well as tissue eQTLs (GTEx Consortium, 2020), experimentally detected protein–protein interactions (PPIs; e.g., Oughtred *et al*, 2019; Luck *et al*, 2020), and Gene Ontology (GO) biological process terms (Gene Ontology Consortium, 2015). Certain features were inferred from a single data source. For example, the feature “lung expression” reflected the expression of each gene in lung and was inferred from transcriptomes of adult lung. Other features were inferred by integrating multiple data sources, mostly tissue transcriptomes with other types of data (Fig 1A). For example, the feature “lung PPIs” reflected the number of PPIs of each protein in lung and was inferred by integrating data of PPIs with lung transcriptomes (Barshir *et al*, 2014; Appendix Fig S1). To support interpretability, we added tissue‐comparative features. For example, “lung preferential expression” reflected the expression of each gene in lung relative to its expression in other tissues (Sonawane *et al*, 2017). Likewise, the feature “lung differential PPIs” reflected the difference, per protein, in the number of its PPIs between lung and other tissues (Materials and Methods).

In addition to including features with known association to tissue‐selective disease manifestation, such as preferential expression of genes (Lage *et al*, 2008), we introduced features that were not previously assessed at large‐scale. One such feature, denoted “differential process activity,” was based on the differential activity of biological processes in a given tissue relative to other tissues, which was shown to illuminate processes that are preferentially active or underexpressed in specific tissues (Sharon *et al*, 2022). The differential activity of a process was recently estimated per tissue from the differential expression in that tissue of the genes annotated to that process (Sharon *et al*, 2022). To include this measure as a gene feature, we associated each gene with its biological processes according to GO and extracted the differential activities of these processes per tissue from Sharon *et al* (2022) (Materials and Methods). Lastly, to reduce interactomics data loss we added abstract network embedding vectors that represented gene neighborhoods in tissue interactomes (Materials and Methods). The entire features dataset included 4,744 distinct features that were computed per protein‐coding gene (Appendix Table S1). Missing values were imputed, and values were transformed and scaled (Materials and Methods, Dataset EV1).

Next, we labeled genes according to whether they underlie tissue‐selective Mendelian diseases. We retrieved Mendelian disease genes from OMIM and combined them with manually curated associations between Mendelian diseases and affected tissues (Barshir *et al*, 2018; Basha *et al*, 2020). Specifically, a disease was considered as affecting a tissue if its clinical manifestation was mainly in that tissue (Materials and Methods). Next, per disease, we associated the disease genes with the disease‐affected tissues. Lastly, we labeled genes per tissue, such that only disease genes that were associated with that tissue were labeled positive, and all other genes were labeled negative. This resulted in a labeled dataset that was more disease‐focused and stringent than used previously (Somepalli *et al*, 2021). Altogether, our features dataset encompassed 18,927 protein‐coding genes, including 1,105 tissue‐associated disease genes that unitedly affected 22 tissues (Dataset EV1).

Eight of the affected tissues, including blood, brain, heart, liver, nerve, skeletal muscle, skin, and testis, were each associated with over 60 disease genes, summing up to a total of 1,031 disease genes (Fig 1B). The tissue‐based features dataset and the tissue‐association labels of genes provided the basis for our ML scheme, denoted “Tissue Risk Assessment of Causality by Expression” (TRACE, Fig 1C). Below we describe the application of TRACE to uncover tissue‐selectivity features of genes and to prioritize candidate disease genes identified in patients (Fig 1D–F).

### TRACE predictions illuminate tissue‐selectivity mechanisms

Our first goal was to test whether ML can be used to infer tissue‐selectivity mechanisms by applying it to well‐studied disease genes. We used the widely successful XGBoost (Chen & Guestrin, 2016; XGB) gradient‐boosting ML method, since decision forest methods perform well on tabular data with thousands of training instances (Fernandez‐Delgado *et al*, 2014), imbalanced classification tasks (Khalilia *et al*, 2011) and high‐dimensional data that include large numbers of dependent features (Schwarz *et al*, 2010). Given a disease gene and its disease‐affected tissue, we created an XGB model by training the model on all other genes, which were labeled according to their association with that tissue (i.e., disease genes whose disease affected that tissue were labeled positive, otherwise they were labeled negative). We then applied the trained model to the query disease gene (Materials and Methods). To rigorously highlight the features that contributed to the decision of each model, we used the SHAP (SHapley Additive exPlanations) algorithm (Lundberg *et al*, 2020), which is a game‐theoretic method for explaining the prediction of ML models.

**Figure 2:**
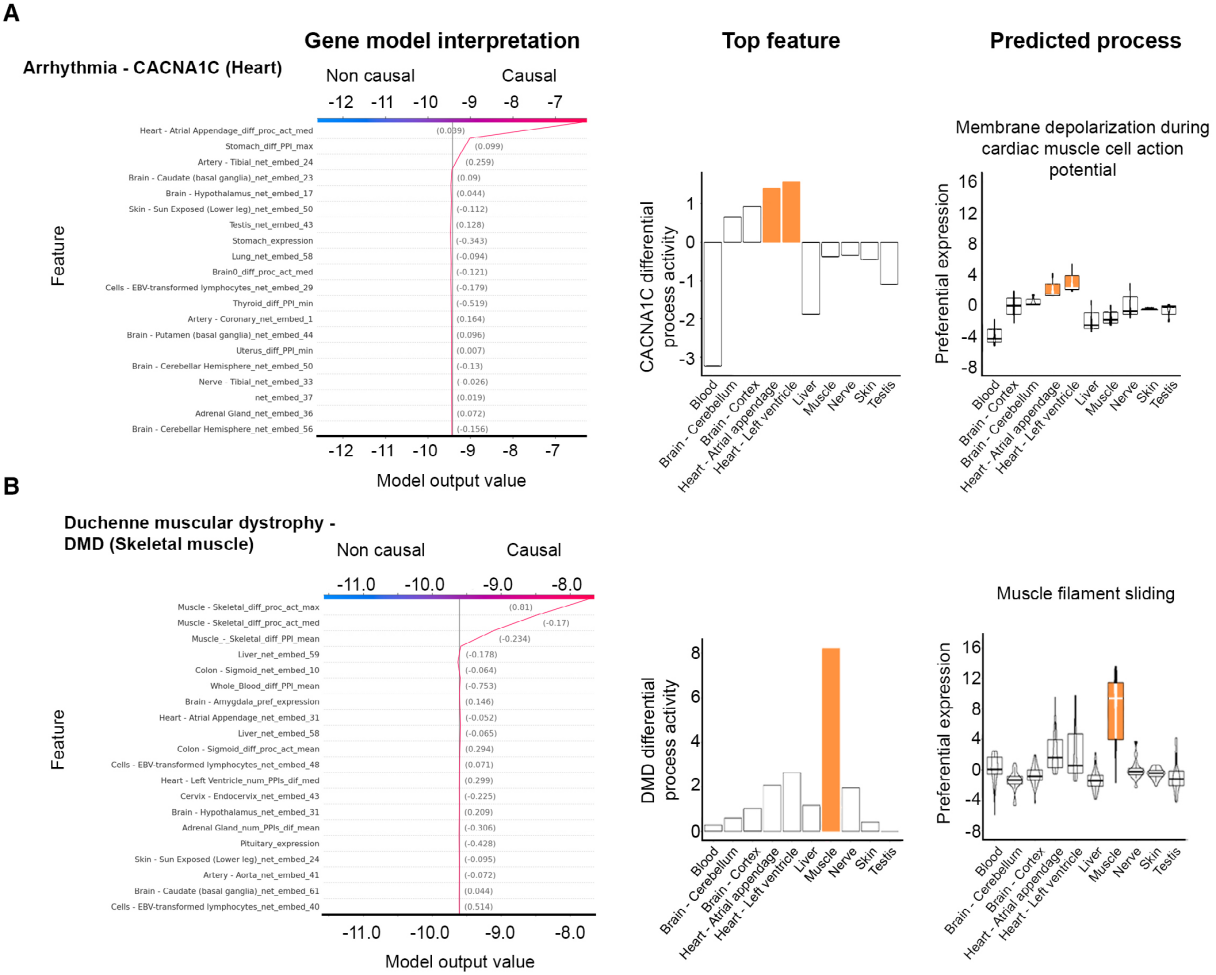
TRACE predictions illuminate disease‐related mechanisms The model for the arrhythmia gene CACNA1C predicted its association with heart (left). The model's topmost contributing feature, “differential process activity in heart,” fits with the high differential process activity of CACNA1C in heart relative to other tissues (middle). This high activity stems from the preferential expression in heart of genes composing arrhythmia–related processes, including “membrane depolarization during atrial cardiac muscle cell action potential” (right).The model for the Duchenne muscular dystrophy gene DMD predicted its association with skeletal muscle (left). The model's topmost contributing feature, “differential process activity in skeletal muscle,” agrees with the exceptionally high differential process activity of DMD in skeletal muscle relative to other tissues (middle). The high activity was due to the preferential expression in skeletal muscle of genes composing the process “muscle filament sliding” (right), previously implicated in Duchenne. Data information: Boxplot central band indicates median; box limits indicate 25^th^ to 75^th^ percentiles; whiskers indicate 1.5 × interquartile range.

We illustrate the resulting inference using the predictions of two broadly expressed disease genes: CACNA1C that underlies arrhythmia, affecting the heart, and DMD that underlies Duchenne muscular dystrophy, affecting skeletal muscle (Fig 2). We selected those genes because they were not expressed exceptionally high in their disease‐affected tissue (Appendix Fig S2). Nevertheless, TRACE successfully classified them as associated with heart and skeletal muscle, respectively. The top contributing feature of each model was the differential process activity of the disease gene, which was highest in the respective disease‐affected tissue (Fig 2). Next, we asked which process contributed to the high value of that feature in the disease‐affected tissue (Appendix Fig S3). In case of the arrhythmia gene CACNA1C, that process was “membrane depolarization during atrial cardiac muscle cell action potential,” in accordance with arrhythmia phenotypes (Fig 2A and Appendix Fig S3A). In case of the Duchenne muscular dystrophy gene DMD, that process was “muscle filament sliding” (Fig 2B and Appendix Fig S3B). This process was indeed found to be impaired in mdx mouse model for Duchenne (Canepari *et al*, 2009). These examples demonstrate that interpretable ML models can point to disease‐related processes.

### Revealing common tissue‐selectivity features of disease genes

**Figure 3:**
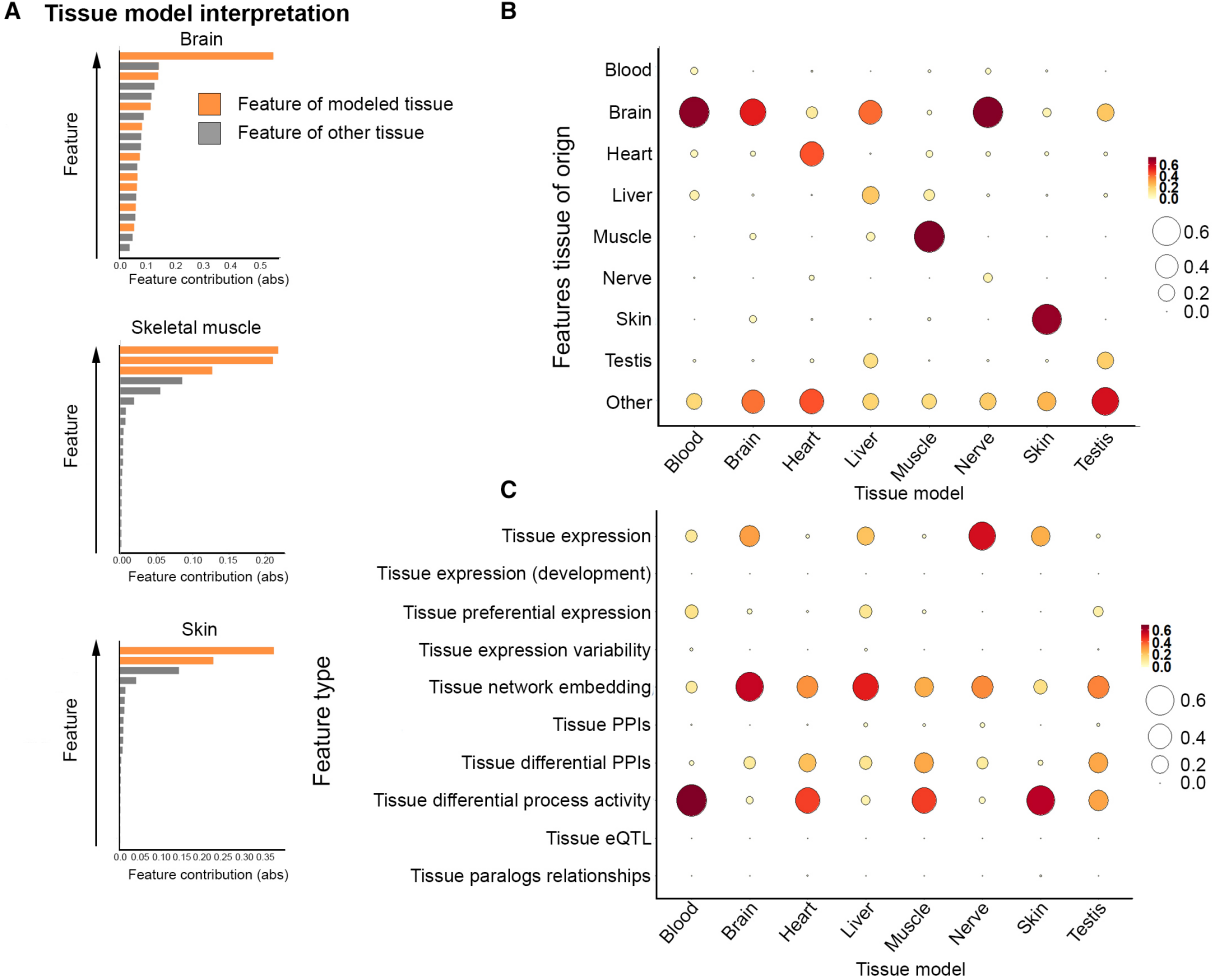
Tissue‐specific models reveal common determinants of tissue selectivity The contribution of the 20 topmost contributing features to the brain, skin, and skeletal muscle models. Features of the modeled tissue, marked by orange bars, were the major contributors to each model. Detailed tissue models appear in Appendix Fig S5.The contribution of tissue‐based features to tissue models upon aggregating features by their associated tissue. Subregions of a tissue were associated with their main tissue. Aggregated features of the modeled tissue were among the three topmost contributing features in the brain, heart, liver, skeletal muscle, skin, and testis models.The contribution of features to tissue models upon aggregating features by their type. Tissue‐differential process activity, tissue network embedding, and tissue expression were the topmost contributing feature types in at least one model.

We extended our analysis to identify common tissue‐selectivity features. For this, we trained XGB classification models that aimed to distinguish tissue‐associated disease genes from other genes. We applied this model to each tissue; however, robust models were created only for eight tissues with over 60 disease genes (Fig 1B, Materials and Methods). We then assessed the performance of the eight models by using 10‐fold cross‐validation. The average area under the receiver operating characteristic curve (AUC) obtained by the various tissue models was 0.71–0.87 (Appendix Fig S4A and B), attesting to discriminative power of the models. Next, we used SHAP to assess the contribution of each feature to each tissue model (Appendix Fig S5). Features that were associated with the modeled tissue, for example, “brain cortex expression” in the brain model and “differential process activity in skin” in the skin model, were among the top six most important features per model, and among the top three in 6/8 models (Fig 3A and Appendix Fig S5). These features were complemented by features of other tissues. For example, the third most contributing feature in the skin model was “differential process activity in subcutaneous fat,” a tissue located just beneath the skin (Appendix Fig S5).

Next, we used SHAP to identify common tissue‐selectivity features in an unbiased manner. For this, we summarized the contribution of the different features per tissue model by aggregating their normalized SHAP importance (Materials and Methods). First, per tissue model we aggregated the contribution of features that were associated with the same tissue (Fig 3B). For example, the contribution of the feature “brain cortex expression” was added to the contribution of other brain‐associated features. In 7/8 tissue models, the modeled tissue was among the top three most contributing tissues out of 54 tissues. This implies that tissue‐selective disease manifestation frequently stems from signifying attributes of the disease‐affected tissue.

Next, we aggregated the contribution of features belonging to the same type of mechanism, regardless of the associated tissue. For example, “brain cortex expression” and “liver expression” were both associated with “tissue expression” (Fig 3C). Tissue expression, indeed a major and well‐established determinant of tissue selectivity, was the topmost mechanism in 1/8 models. Tissue network embedding, which captures interactome neighborhood, another recognized factor, was topmost in 3/8 models. Notably, differential process activity, which was not previously recognized as a determinant of tissue selectivity, was topmost in 4/8 tissue models, attesting to its wide relevance. This implies that in many diseases, tissue‐selective manifestation is driven by a process whose integrity is essential for tissue physiology. Information on the identity of that process could enhance our understanding of the disease and consequently help open avenues for therapy (Fig 2). Differential PPIs and preferential expression were also among the top features across models, suggesting that tissue‐selective disease manifestation stems from both absolute (e.g., expression) and relative (e.g., preferential expression) tissue‐based features of disease genes.

### Cataloging the tissue‐specific risks of protein‐coding genes

**Figure 4:**
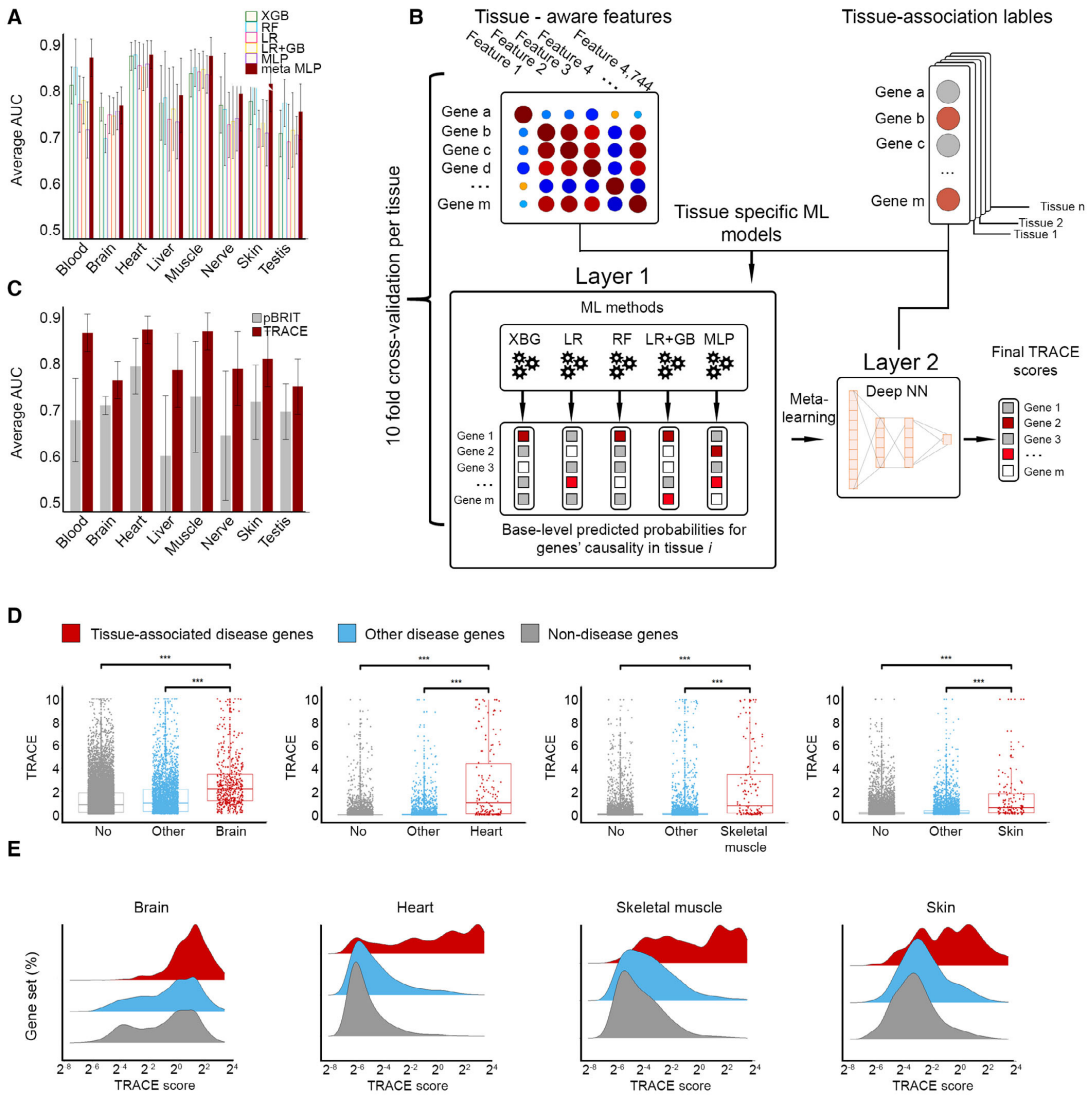
TRACE prioritization of tissue‐associated disease genes The average AUC obtained per ML method and tissue model. The highest AUC was typically obtained by the meta‐learner.A schematic view of the final TRACE scheme. Input to TRACE included the dataset of tissue‐based gene features and tissue‐association gene labels for the modeled tissue (red labels mark disease genes for Mendelian diseases that manifest in the modeled tissue). The first layer includes independent application of five ML methods. Each method outputs gene scores reflecting the predicted probability that the gene underlies a disease that manifests in the modeled tissue. The second layer uses gene scores as input to a neural network (NN) meta‐learner that produces the final TRACE score (red labels mark genes with higher predicted scores).The average AUC obtained with pBRIT (gray) and TRACE (crimson) per tissue model.Gene TRACE scores in brain, heart, skeletal muscle, and skin models. Each dot represents a different gene. Genes were divided into disease genes whose disease manifests in the modeled tissue (tissue‐associated disease genes, red), disease genes whose disease does not manifest in the modeled tissue (other disease genes, blue), and nondisease genes (gray). Tissue‐associated disease genes had significantly higher TRACE scores compared with nondisease genes and to other disease genes (*** = 3E‐16; MW, adjusted *P*‐values).Density ridge plots of gene TRACE scores in brain, heart, skeletal muscle, and skin models. Tissue‐associated disease genes were over‐represented among genes with high TRACE scores. Data information: Bars and error bars indicate the mean ± SD obtained via 10‐fold cross‐validation. Boxplot central band indicates median; box limits indicate 25^th^ to 75^th^ percentiles; whiskers indicate 1.5 × interquartile range. Plots for additional tissue models appear in Fig EV1A and B.

Our next step was to create a ML‐based catalog of tissue‐associated risks of protein‐coding genes. For this, we tested the performance of five ML classifiers including logistic regression (LR), a multilayer perceptron (MLP) neural network, and three tree‐based ensemble methods that included XGB, random forest (RF), and gradient boosted trees initiated by a logistic regression model (LR + GB). For each of the eight modeled tissues, each classifier computed the probability of each gene to underlie a disease that manifests in that tissue. The performance of each classifier was then assessed per modeled tissue via 10‐fold cross‐validation by using the average AUC and area under the precision–recall curve (auPRC; Materials and Methods, Appendix Fig S6A and B). Best performance across tissue models was achieved by different ML tools. For example, XGB performed better than other ML tools in two tissue models with respect to AUC and in two other tissue models with respect to auPRC (Appendix Fig S6A and B). Therefore, we next tested whether the combination of ML methods could lead to better results. For this, we employed a deep neural network meta‐learner (Vilalta & Drissi, 2002; meta‐MLP). Per gene, the meta‐MLP received as input the gene values obtained by the five classifiers and produced a final score. The meta‐MLP typically obtained top AUC (0.75–0.87, Fig 4A) and top auPRC (Appendix Fig S6B). Thus, our final TRACE scheme was composed of two layers: The first layer combined the five ML classifiers, which computed the probability of each gene to underlie a disease that manifested in the modeled tissue, scaled to values between 0 and 10 per classifier. The second layer consisted of a meta‐MLP that accepted as input the output of the five classifiers and produced a final TRACE score, also scaled between 0 and 10 (Fig 4B, Materials and Methods). Across tissue models, TRACE achieved average AUC and auPRC of 0.82 and 0.12 (expected 0.04), respectively (Fig 4C, Appendix Fig S7). Better performing models corresponded to tissues with larger numbers of tissue‐associated disease genes (Fig 1B), suggesting that increased annotation efforts could improve prediction. We compared TRACE to pBRIT, a computational gene prioritization tool that correlates functional and phenotypic gene annotations through intermediate data fusion (Kumar *et al*, 2018; Materials and Methods). Although pBRIT relied on more types of functional and phenotypic gene annotations, TRACE performance was favorable, especially with respect to AUC (Fig 4C and Appendix Fig S7).

**Figure EV1:**
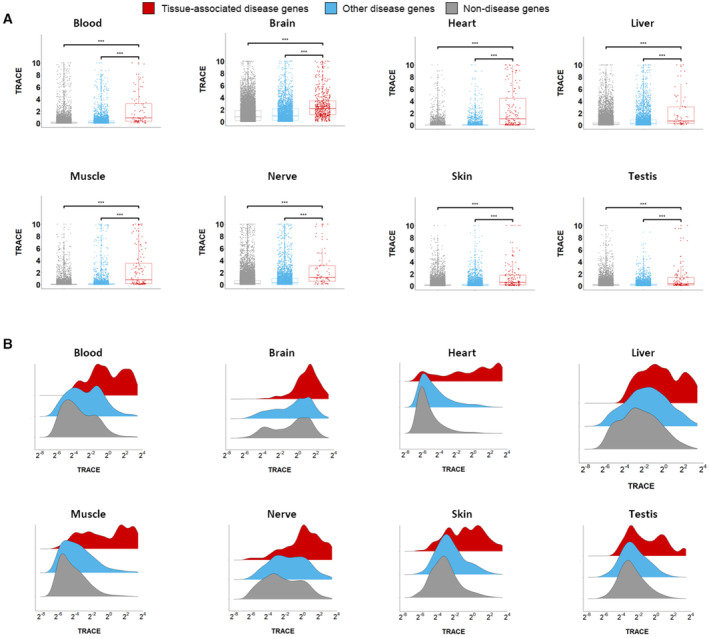
TRACE prioritization of genes associated with diseases that manifest in distinct tissues Gene TRACE scores in the different tissue models. Each dot represents a different gene. Genes were divided into genes that are causal for a disease that manifests in the modeled tissue (tissue‐associated, red), genes that are causal for a disease that does not manifest in the modeled tissue (other disease genes, blue), and nondisease genes (gray). Tissue‐associated disease genes had significantly higher TRACE scores compared with nondisease genes and to other disease genes. Mann–Whitney *U* test (MW) adjusted *P*‐values for the comparison of tissue‐associated genes to noncausal genes: Blood (2.85*10^−16^), brain (2.85*10^−16^), heart (2.85*10^−16^), liver (4.44*10^−13^), skeletal muscle (2.85*10^−16^), nerve (2.85*10^−16^), skin (2.85*10^−16^), testis (2.85*10^−16^). MW adjusted *P*‐values for the comparison of tissue‐associated genes to other disease genes: Blood (2.22*10^−16^), brain (2.22*10^−16^), heart (2.22*10^−16^), liver (1.10*10^−6^), skeletal muscle (2.22*10^−16^), nerve (3.70*10^−11^), skin (2.22*10^−16^), and testis (9.80*10^−12^). Boxplot central band indicates median; box limits indicate 25^th^ to 75^th^ percentiles; whiskers indicate 1.5 × interquartile range. The number of analyzed values was ~60–500 for tissue‐associated genes, ~3,430–3,860 for other disease genes, and ~15,000 for nondisease genes. Panels relating to brain, heart, skeletal muscle, and skin are the same as those in Fig 4D.Density ridge plots of gene TRACE scores in the different tissue models. Tissue‐associated genes (red) were over‐represented among genes with high TRACE scores. Panels relating to brain, heart, skeletal muscle, and skin are the same as those in Fig 4E.

To further assess the tissue selectivity of TRACE, we divided protein‐coding genes into three separate groups per tissue model. The first group consisted of tissue‐associated disease genes, namely disease genes whose disease manifests in the modeled tissue. The second group consisted of other disease genes, namely disease genes whose disease does not manifest in the modeled tissue. The last group consisted of nondisease genes. We then compared between the TRACE scores of the different gene groups (Figs 4D and E, and EV1A and B). Across models, tissue‐associated disease genes were enriched among genes with high TRACE scores. They had significantly higher TRACE scores when compared to nondisease genes (*P* ≤ 4.4E‐13, Mann–Whitney *U* test [MW]) and to other disease genes (*P* ≤ 1.1E‐6, MW), attesting to the tissue specificity of the models. To test whether high TRACE scores could predict tissue association in the absence of tissue‐specific gene expression, we applied TRACE to the subset of 8,749 genes that were expressed in at least 80% of the tissues. We then compared between the TRACE scores of the three groups of genes described above. Apart from the liver tissue model, tissue‐associated disease genes had significantly higher TRACE scores compared with nondisease genes and to other disease genes in all tissue models (Appendix Fig S8, *P* < 0.029, MW). Hence, TRACE predictions were not limited to tissue‐specific genes. A similar tendency was observed for the subset of genes that were not overexpressed in any tissue (Appendix Fig S9). Lastly, we tested how often TRACE ranks disease genes highest in their associated tissue. For that, we compared TRACE ranks of disease genes between models of associated and other tissues. The median rank in the associated tissue model was higher than the median rank in other tissue models, except for blood‐associated genes whose median rank was higher in liver (Appendix Fig S10).

**Figure EV2:**
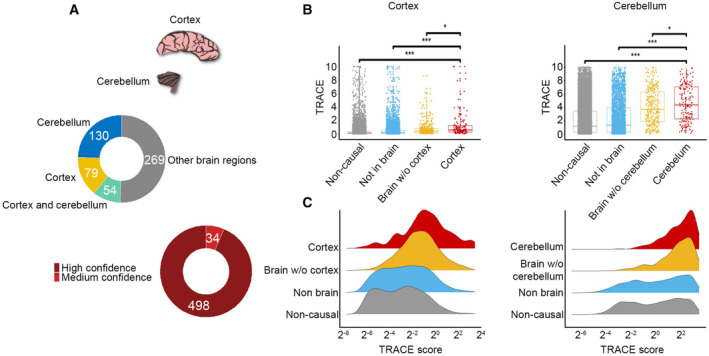
TRACE prioritization of genes associated with diseases that manifest in brain cortex and cerebellum The 532 genes known to be causal for brain diseases were associated with brain regions that manifest the disease at medium to high confidence. Most genes were associated with cerebellum and cortex.Gene TRACE scores in brain cortex and cerebellum models. Each dot represents a different gene. Genes were divided into nondisease genes (noncausal, gray), disease genes that are not causal for brain diseases (nonbrain, blue), disease genes that are causal for brain diseases that do not manifest in the modeled brain region (brain w/o modeled region, orange), and disease genes that are causal for brain diseases that manifest in the modeled brain region (modeled brain region, red). Genes associated with the modeled brain region had significantly higher TRACE scores compared with all other gene sets (Cortex: *** = 3.3E‐16, * = 0.028; cerebellum: *** = 3.3E‐16, * = 0.033; MW, adjusted *P*‐values shown). Boxplot central band indicates median; box limits indicate 25^th^ to 75^th^ percentiles; whiskers indicate 1.5 × interquartile range. The number of analyzed values was ~130–180 for disease genes of the modeled brain region, ~320–370 other brain disease genes, ~3,420 for other disease genes, and ~15,000 for nondisease genes.Density ridge plots of gene TRACE scores in brain cortex and cerebellum models. Genes associated with the modeled region were over‐represented among genes with high TRACE scores.

So far, we applied TRACE to disease‐affected tissues that were physiologically distinct from each other. Next, we turned to analyze the complex and often‐uncertain selectivity of brain diseases to brain regions (Moustafa *et al*, 2016). We divided brain into six regions with available transcriptomic profiles, including cortex, cerebellum, basal ganglia, spinal cord, hypothalamus, and amygdala (Dataset EV2). We manually associated brain diseases to inflicted brain regions based on anatomical findings (see Materials and Methods). Altogether, we associated with low to high confidence 649 diseases and 832 disease genes to inflicted brain regions. 594 diseases and 532 disease genes were associated with medium or high confidence and were labeled as associated with those regions. Cortex and cerebellum were associated with over 60 disease genes and were henceforth modeled (Fig EV2A and Dataset EV2). As observed for physiologically distinct tissues, TRACE models were discriminative (AUC of 0.77 in both). Cortex‐associated and cerebellum‐associated disease genes had significantly high TRACE scores when compared to nondisease genes and to brain‐unrelated disease genes (*P* ≤ 3.3E‐16, MW, Fig EV2B and C). In support of the models' specificity, cortex‐associated and cerebellum‐associated disease genes also had significantly high TRACE scores relative to other brain‐associated disease genes (adjusted *P*‐value of 0.028 and 0.033, respectively, MW, Fig EV2B). Hence, TRACE models favored tissue‐associated disease genes over disease genes that manifested in physiologically related tissues.

A catalog of the tissue‐associated risks of each protein‐coding gene in our dataset is available through the TRACE webserver (https://netbio.bgu.ac.il/trace/). Users can download the catalog, or upload a gene list or a VCF file, select a disease‐inflicted tissue, and obtain the respective TRACE scores.

### TRACE application to genetic diagnosis of rare‐disease patients

**Figure 5:**
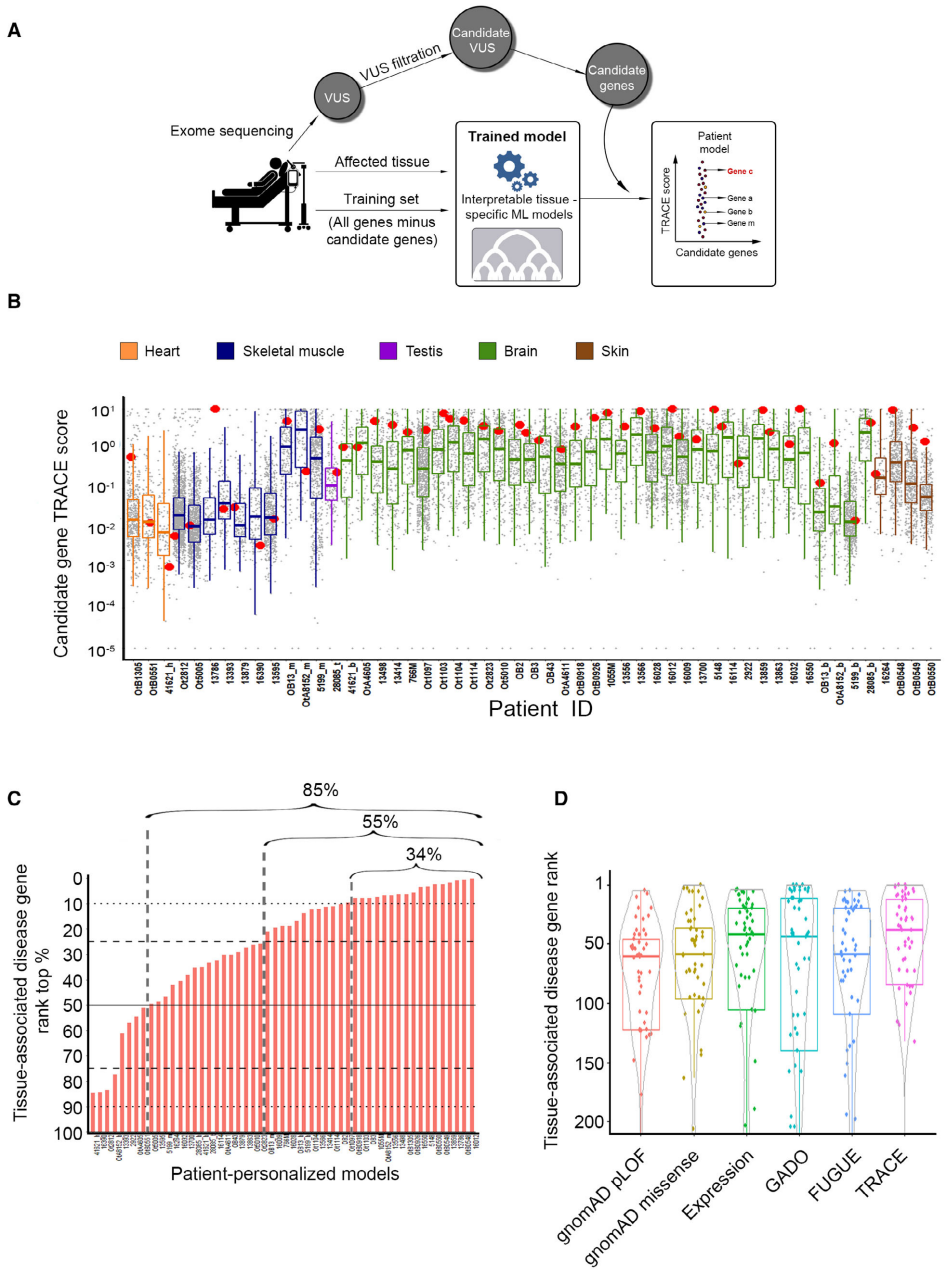
TRACE prioritization of patient‐derived candidate disease genes associated with rare diseases A schematic view of a patient‐tailored TRACE modeling. Variants identified via exome sequencing of the patient were filtered by standard approaches, and remaining variants were mapped to their harboring genes, denoted candidate disease genes. Next, a TRACE model was trained on all genes except for the patient's candidate disease genes, using gene labels that match the affected tissue of the patient. Lastly, the trained model was applied to the patient's candidate disease genes, which were then prioritized according to their TRACE scores. The red‐labeled gene is the verified disease gene, which contains the pathogenic variant of that patient.TRACE scores of candidate disease genes in 53 models for 48 patients with tissue‐selective rare diseases (five patients were modeled in two distinct tissues). Per model, each dot represents a candidate disease gene; the red dot marks the verified disease gene of that patient. Boxplot color reflect the modeled tissue (orange = heart, blue = skeletal muscle, purple = testis, green = brain, brown = skin). In 46/53 models, the verified disease gene was ranked above the median.The rank of the verified disease genes out of the patient's candidate genes. Per model, ranks were determined based on gene TRACE scores, such that top scoring gene was ranked first. 85% of the verified disease genes ranked above the median; 55% of the verified disease genes ranked at the top quartile; and 34% of the verified disease genes ranked at the top 10%.Comparison between the rank of the verified disease genes out of the patient's candidate disease genes between gnomAD (Karczewski *et al*, 2020), expression‐based prioritization, GADO (Deelen *et al*, 2019), FUGUE (Somepalli *et al*, 2021), and TRACE. Median rank of TRACE: 39; gnomAD pLoF: 61, missense: 60; expression‐based prioritization: 43; GADO: 45; FUGUE: 60. TRACE prioritization was better than prioritization by the other methods (adjusted *P* = 0.00155, 0.01, 0.01, 0.01, 0.01, respectively, Wilcoxon signed‐rank test; ranking outside top 200 appear in Appendix Fig S11B). Data information: Boxplot central band indicates median; box limits indicate 25^th^ to 75^th^ percentiles; whiskers indicate 1.5 × interquartile range.

Tissue contexts may play a role in the genetic diagnosis of patients with rare diseases that manifest in a tissue‐selective manner. However, popular prioritization tools in clinical settings, such as CADD (Rentzsch *et al*, 2019) and gnomAD (Karczewski *et al*, 2020), are oblivious to tissue contexts (Eilbeck *et al*, 2017). To test the relevance of tissue contexts in such settings, we applied TRACE to a test set of 48 patients. Patients previously underwent whole‐exome sequencing and variant characterization, and their pathogenic variant had been reported, as described in Materials and Methods (Fig 5A, Dataset EV3A).

To assess TRACE per patient, we compiled a list of the patient's genes that contained variants, henceforth referred to as candidate disease genes. The median number of candidate genes per patient was 186. Next, we created a TRACE model of the disease‐affected tissue of the patient. For five patients that had two affected tissues, we created two TRACE models per patient. To ensure unbiased testing and avoid data leakage, we trained each TRACE model on all genes except for the patient's candidate disease genes; the genes used for training were labeled according to their association with the disease‐affected tissue of the patient. We then applied the trained model to the patient's candidate disease genes to predict their association with the disease‐affected tissue and prioritized them by their TRACE scores. Lastly, we ranked the gene containing the pathogenic variant, denoted the verified disease gene of the patient, relative to the patient's candidate disease genes (Fig 5B).

Though TRACE analysis of candidate disease genes per patient was literature blind and relied only of large‐scale profiling data, in patients whose verified disease gene was well‐established (though some contained novel pathogenic variants), TRACE ranked the verified disease gene at the top 1% of the patient's candidate genes. Examples include OPA1 that was verified in a patient with optic atrophy and neuropsychiatric disorders (MIM #125250); Sarcoglycan Gamma (SGCG) that was verified in a patient with muscular dystrophy (MIM #253700); and tumor protein p63 (TP63) that was verified in a patient with for ectodermal dysplasia syndrome (MIM #103285, #604292).

Analysis of patients whose verified disease gene was recently discovered further demonstrated the utility of TRACE. In one example, a familial case of lethal, severe microcephaly with various neurological features, the patient was found to have a unique mutation in SEC31A (Halperin *et al*, 2019). TRACE ranked SEC31A as the eighth most likely disease gene out of 107 patient's candidate disease genes. In a different case, a familial syndrome of muscle hypotonia, failure to thrive, and developmental delay, a variant in PAX7 was identified as pathogenic (Proskorovski‐Ohayon *et al*, 2017). This disease affects multiple organs but manifests most severely in skeletal muscle. TRACE ranked PAX7 in skeletal muscle as the 10^th^ most likely disease gene out of 150 candidate disease genes.

In general, TRACE ranked the verified disease gene above the median in 85% of the cases, and at the top quartile or top 10% of the patient's candidate disease genes in 55 and 34% of the cases, respectively (Fig 5C). These results suggest that genes that TRACE ranks below the median can typically be removed from further consideration. TRACE is intended for usage after automatic filtration of candidate genes by widely used tools. Sieving through the remaining candidate genes is time‐consuming and often done manually by geneticists, hence cutting the list of candidate genes by half can alleviate geneticists' workload. Yet, additional advancements are required for efficient clinical usage.

These results also show that tissue contexts are important and relevant for genetic diagnosis. For example, ranks of verified disease genes were higher in the correct (affected) tissue model than in other tissue models (Appendix Fig S11A). Highest success rates were observed for brain and skin, which had relatively large numbers of tissue‐associated disease genes and performed well in cross‐validation: In 20/33 (61%) of the patients with brain‐related diseases, the verified disease gene ranked at the top quartile, and in 3/4 patients with skin diseases, the verified disease gene ranked at the top 3%. To further assess TRACE performance, we tested whether the five top‐ranking genes per patient could be functionally relevant specifically in the patient's affected tissue. Indeed, we found that 75% of them were associated with a GO process that was specific to the disease‐affected tissue of the patient (Materials and Methods). For example, the five top‐ranking candidate genes of patient #13786 that was diagnosed with muscular dystrophy, including the verified disease gene SGCG, were all associated with muscle‐specific processes, such as “muscle contraction” and “muscle organ development” (Dataset EV3D).

We compared TRACE prioritization to other gene prioritization schemes (Fig 5D and Dataset EV3B). The first scheme was prioritization by the expression level of candidate disease genes in the disease‐affected tissue. The second scheme was GADO, a recently published computational tool that uses human tissue transcriptomes to prioritize genes based on disease phenotypes (Deelen *et al*, 2019). The third scheme was pLoF or missense scores of gnomAD, a state‐of‐the‐art gene‐level metric that assessed gene constraints by the observed versus expected frequency of gene variants in the population (Karczewski *et al*, 2020). The fourth scheme was FUGUE, a recently published method for prioritizing tissue‐relevant genes (Somepalli *et al*, 2021). Since the patients in our dataset were diagnosed with the help of prioritization tools, their performance was evidently high (Fig 5D). Nevertheless, TRACE performed well, especially with respect to the median rank of the verified disease gene (Fig 5D and Dataset EV3C). gnomAD, which was used to assemble a list of candidate disease genes per patient, was less effective in the final prioritization. Its reduced performance relative to expression‐based scheme showed that knowledge of the disease‐affected tissue can contribute greatly to gene prioritization. The improved performance of TRACE over the expression‐based scheme and FUGUE implied that other features, beyond expression, were meaningful for prioritization.

## Discussion

We presented a ML approach for revealing and explaining tissue‐specific functional consequences of genetic variation that leads to tissue‐selective diseases. Our approach was motivated by the success of ML methods in biology (Gligorijevic & Przulj, 2015) and by known determinants of tissue selectivity (Lage *et al*, 2008; Magger *et al*, 2012; Barshir *et al*, 2014, 2018; Greene *et al*, 2015; Kitsak *et al*, 2016; Marbach *et al*, 2016; Barbeira *et al*, 2018; preprint: Barbeira *et al*, 2019; Hekselman & Yeger‐Lotem, 2020). Thus, we harnessed various omics data to create tissue‐based multiomics gene signatures with mechanistic interpretation (Appendix Table S1), which were much richer relative to previous efforts (Somepalli *et al*, 2021). By employing early data integration techniques, rather than late (Aerts *et al*, 2006) or intermediate (Kumar *et al*, 2018), and interpretable ML methods, TRACE was able to asses elaborated combinations of different types of gene features simultaneously (Gligorijevic & Przulj, 2015), estimate their relative importance (Figs 2 and 3), and use them for genetic diagnosis of patients (Fig 4).

As part of the work on TRACE, we created three large‐scale resources for scientists and clinicians in the field of genomic medicine. First, we created a manually curated dataset of 532 brain diseases and their affected brain subregions (Dataset EV2). Second, we created a tabular ML‐oriented dataset of 18,927 protein‐coding genes multiomics signatures, which can be readily utilized by other ML frameworks. The signatures combined multiple types of data, such as transcriptomics and PPIs. Though some of the features in this dataset were correlated, we preferred to let models decide which features add information with respect to other dominant features of the model and applied feature selection where necessary to avoid overfitting (Materials and Methods). Lastly, we created a catalog of the tissue‐associated risks for 18,927 protein‐coding genes. The value of this catalog was demonstrated by analyzing data from patients (Fig 5).

Like other ML schemes, TRACE is inherently incomplete. The features dataset could be extended to include other gene features. In particular, many rare diseases have fetal origin; however, TRACE features were based mostly on adult tissue profiles since fetal tissue profiles were available only for seven organs (Cardoso‐Moreira *et al*, 2019; Appendix Table S1). Likewise, the labeled dataset could be extended to include more tissue‐associated disease genes. This could help improve prediction, as demonstrated by the enhanced performance of models based on larger numbers of such genes, and allow modeling of additional tissues. Alternatively, the goal of the classification and consequently the labeling could change. For example, by labeling genes that were not expressed in a tissue as negatives, the ML scheme could be used to prioritize genes with tissue‐specific functions rather than disease–tissue associations (Somepalli *et al*, 2021). Additionally, tissue association of disease genes was represented by a binary variable. This dichotomy fits well with diseases that have clear tissue‐specific manifestation, such as neurodegenerative disorders or skin diseases, yet might be less suitable for syndromes that affect multiple tissues to varying extents, or diseases with unclear or indirect tissue manifestations. The latter class of diseases could benefit greatly from multiclass classification; however, current data for training such models are scarce. Lastly, TRACE ranked verified disease genes high relative to other candidate genes of the same patient (median rank of 39/186 genes) and relative to other methods (Fig 5). However, additional advancements are needed to make this approach clinically actionable. This is especially important upon considering that patient exomes contain a much larger number of candidate genetic variants to which the current scheme is oblivious.

Despite limitations, TRACE demonstrated the power of ML to boost the understanding of tissue‐selective diseases. The relative contribution of certain features to TRACE models could be used to generate hypotheses regarding disease mechanisms, such as their developmental origins. Another strength of ML is in unbiased estimation of novel features. This was exemplified by our assessment of biological process activity, which revealed its common, previously understudied role (Fig 3C). Importantly, once identified, this feature highlighted disease‐related processes (Fig 2). Lastly, TRACE prioritized candidate disease genes in patients with rare tissue‐selective diseases (Fig 5). Since common variant prioritization schemes rely primarily on genomic attributes of variant sequences and are typically oblivious to tissue contexts (Eilbeck *et al*, 2017), TRACE provides a powerful complementary addition to current variant prioritization pipelines.

The application of TRACE provided insight into common determinants of tissue selectivity (Fig 3). Some of them were already shown to be common, such as preferential expression of disease genes in disease‐inflicted tissues or their involvement in tissue‐specific PPIs (Lage *et al*, 2008; Barshir *et al*, 2014; Fig 3B and C). Other known determinants of tissue selectivity were not highlighted. Tissue‐selective underexpression of paralogs (Barshir *et al*, 2018; Jubran *et al*, 2020) was potentially masked by other features (Barshir *et al*, 2018). Involvement in tissue‐specific eQTLs, which was previously observed in trait‐associated genes (Barbeira *et al*, 2018; preprint: Barbeira *et al*, 2019), was less frequent in Mendelian diseases (Yao *et al*, 2020). And tissue‐selective underexpression, which was previously observed in loss‐of‐function cancer genes (Lage *et al*, 2008), was probably masked by the preferential expression of disease genes, which was found to be much more common (Lage *et al*, 2008; Hekselman & Yeger‐Lotem, 2020). Notably, the most common feature was tissue‐preferential activity of biological processes, which involve multiple genes beside the disease gene. This suggests a mechanistic resemblance between monogenic diseases and multigenic complex traits. In the future, it will be interesting to extend TRACE toward additional phenotypes whose genetic mapping is routine yet mechanistic understanding is lagging, such as genome‐wide association studies of complex traits, and toward multitissue disorders with potentially common disease‐related features.

Several conclusions of broader impact can be drawn from our study. First, we showed that information on tissue‐selective clinical manifestation of a disease is important for identifying its molecular mechanisms. Since this information is largely missing from databases devoted to diseases or clinical variants, such as OMIM and ClinVar, we strongly suggest to document this information in an easily retrievable format (e.g., Hekselman *et al*, 2022). Second, by utilizing high‐level observational patient data (which of the patient's tissues were affected by disease) and combining it with general multiomics data, our study demonstrated that a personalized‐to‐nonpersonalized data channel is extremely practical and, in many cases, could be essential for personalized medicine. Whereas in some cases acquiring patient‐specific omics data is feasible (Cummings *et al*, 2017), in many clinical settings throughout the world and for patients with, for example, brain diseases, this could be impractical and unachievable. This by itself greatly limits the ability to translate omics‐based knowledge to clinical use. Therefore, frameworks that leverage non‐patient‐specific omics data that was acquired at large scale and combine it with data from individual patients, such as patients' exomes or symptoms, are greatly needed. Such frameworks could also bring real value and more immediate clinical impact to basic‐science endeavors, which tend to translate to clinical use much later on. Third, ML schemes that utilize both interpretable and noninterpretable data are a favorable choice when developing ML frameworks for biological and clinical applications. Whereas “black box” methods, such as various deep learning architectures, could achieve some performance gain, they lack the ability to convey to human researchers and clinician the rational for ML recommendations and the biological signals that they are based on. As we showed, it is possible to embed “black‐box”‐style performance‐boosting data in a way that does not undermine the interpretability of the ML models. Fourth, our study highlighted the importance of examining the behavior of disease genes in disease‐inflicted and disease‐unaffected tissues, leading toward a holistic approach to their investigation. The limited mechanistic understanding of rare diseases and the numbers of undiagnosed patients worldwide (Investigators *et al*, 2021) call for additional efforts and ready‐to‐use tools for boosting disease diagnosis and research.

## Materials and Methods

### Gene expression datasets and processing

Transcriptomic profiles of adult human tissues measured via RNA‐sequencing were obtained from GTEx v8 and consisted of 17,382 profiles sampled from 54 tissues (GTEx Consortium, 2020). Expression values were available for 43,025 genes, including 18,927 protein‐coding genes. Raw reads were normalized to obtain the same library size for every sample by using the trimmed mean of M‐values (TMM) method by the edgeR package (Robinson *et al*, 2010). Genes with at most 10 raw counts in every sample were removed before normalization, as these genes were typically regarded as noise. To exclude protein‐coding genes that were not reliably expressed in a specific tissue, only genes expressed at least 7 counts per million (cpm, linear scale) in at least half the samples of a tissue were considered as expressed in that tissue. Only protein‐coding genes that were expressed in at least one tissue were further considered.

Preferential expression values per gene and tissue were computed from normalized counts as in Sonawane *et al* (2017) (see equation 1). Similarly to Sonawane *et al* (2017), genes with preferential expression ≥ 2 in a tissue were considered as preferentially expressed in that tissue. Transcriptomic profiles of developing human organs consisted of gene normalized counts that were measured at several time points during development and in adulthood in seven human organs, including cerebrum, cerebellum, heart, kidney, liver, ovary, and testis (Cardoso‐Moreira *et al*, 2019). The expression of a gene in an organ and a time point was set to its median (*med*) normalized count in samples from the same time point and organ, resulting in a total of 133 profiles.

(1) $$\forall t\in T,\forall g\in G:{\text{pref}}_{g}^{\left(t\right)}=\frac{\left[\mathrm{med}\left(e_{g}^{\left(t\right)}\right)-\mathrm{med}\left(\mathrm{med}\left(e_{g}^{\left(\forall t\in T\right)}\right)\right)\right]}{\mathrm{IQR}\left(\mathrm{med}\left(e_{g}^{\left(\forall t\in T\right)}\right)\right)}$$

*T* denotes the set of tissues; *G* denotes the set of genes; *pref* denotes preferential expression; *e* denotes normalized count; *IQR* denotes interquartile range, that is, the difference between the 75^th^ and 25^th^ percentiles of the medians.

### Construction of the dataset of tissue‐based gene features

The features dataset consisted of 4,744 features per protein‐coding gene. Below we describe the different types of features (see Appendix Table S1), as well as imputation and scaling of the dataset.

#### Transcriptomic features

Per tissue, each gene was associated with several transcriptomic features. The first set of features reflected the expression level of the gene in the given tissue according to GTEx (if a gene was not expressed in the given tissue its value was set to zero). The second set of features corresponded to the gene's preferential expression in the given tissue relative to all tissues according to GTEx: Positive values reflected overexpression in the given tissue, and negative values reflected underexpression. Another set of transcriptomic features reflected the expression of genes per organ and per timepoint during development according to Cardoso‐Moreira *et al* (2019).

#### eQTL features

Data of tissue eGenes, that is, genes involved in a tissue eQTL, were downloaded from the GTEX portal (March 20^th^, 2019, “GTEx_Analysis_v7_eQTL.tar.gz”). Each gene was associated with a feature per tissue that corresponded to its eGene *q*‐value in that tissue.

#### PPI features

Data of experimentally detected PPIs were downloaded from publicly available databases using the default option of the MyProteinNet web tool (Basha *et al*, 2015). Each gene was associated with the set of interactors of its corresponding protein. Next, we integrated PPIs with GTEx expression data. Specifically, per tissue, we assigned each gene with three tissue‐based primary PPI‐related features, as follows: (i) Tissue interactors: Every gene that was considered to be expressed in the given tissue was assigned with the number of its interactors that were also considered to be expressed in that tissue; otherwise its value was set to 0. (ii) Preferential tissue interactors: Every gene that was considered to be expressed in the given tissue was assigned with the number of its interactors that were considered to be preferentially expressed in that tissue; otherwise its value was set to 0. (iii) Tissue‐specific interactors: For every gene with nonzero tissue interactors in that tissue, the gene was assigned with the number of tissue interactors with which it interacted in at most 20% of the tissues (i.e., both the gene and its interactor were expressed in the same tissue in at most 20% of the tissues). For each primary feature (i)–(iii), we calculated two additional features per gene and tissue, to reflect the difference between the primary gene value in that tissue and its expected value. The expected value was calculated in two different ways, once according to the gene's mean primary value across tissues, and once according to the gene's median primary value across tissues.

#### Tissue‐differential PPI features

Data of differential PPIs per gene and tissue were downloaded from The DifferentialNet database (Basha *et al*, 2018). This database assigns each PPI with a score per tissue that reflects whether the two interacting proteins are overexpressed or underexpressed in that tissue relative to all other tissues. Using these data, we associated each gene with the set of its differential interaction scores per tissue, and created four gene features per tissue, as follows: (i) the minimum differential interaction score of the gene in the given tissue. (ii) The maximum differential interaction score of the gene in the given tissue. (iii) The median differential interaction score of the gene in the given tissue. (iv) The mean differential interaction score of the gene in the given tissue. We included this set of potentially correlated features as we preferred to let the model decide which feature adds more information with respect to other dominant features in the model.

#### Network embedding features

Network embedding features were designed to represent the interactome neighborhood of a gene in a given PPI network (interactome). We started by creating a general interactome that contained all experimentally detected PPIs (see PPI features section above). Next, we integrated the general interactome with GTEx expression data to create tissue interactomes. Specifically, the interactome of a given tissue was set to include all PPIs between protein products of genes that were considered as expressed in that tissue. Lastly, we applied network embedding to the general human interactome and to each of the tissue interactomes. For each interactome, network embedding was computed by using the node2vec algorithm (Grover & Leskovec, 2016). Each gene was sampled by 20 walks of length 10, and every interactome was represented by embedding vectors of 64 dimensions.

#### Expression variability features

Data of expression variability scores per gene and tissue were obtained from Simonovsky *et al* (2019). Scores reflected the variability in expression levels of a gene across samples of a given tissue and were available for 19 tissues. Expression variability scores during development per gene and organ were computed based on Cardoso‐Moreira *et al* (2019). Per organ and time point, each gene was assigned with its median normalized counts over the respective samples. Next, the expression variability of a gene in that organ was set to the coefficient of variation computed over the gene's median normalized counts in all developmental time points of the given organ.

#### Paralogous genes' features

Paralogous genes were defined as gene pairs whose reciprocal sequence identity was ≥ 40% according to Ensembl‐BioMart. We assessed the quantitative relationships between paralogous genes as described in Barshir *et al* (2018). Specifically, we calculated per sample the expression ratio between a gene and its best‐matching paralog, where the best‐matching paralog was defined as the paralog with the highest sequence identity that was expressed in any tissue according to GTEx. Next, we created a feature per tissue where each gene was assigned with the median expression ratio computed across samples of that tissue. To account for genes with multiple paralogs, we calculated per sample the ratio between the expression of a gene and the summed expression of all its paralogs with over 40% reciprocal sequence identity. Next, we created another feature per tissue where each gene was assigned with the median ratio computed across samples of that tissue.

#### Differential process activity features

We associated genes with biological processes by using Gene Ontology (The Gene Ontology Consortium, 2019; GO) terms and gene annotations, which were downloaded from Ensembl‐BioMart. We favored specific rather than general biological processes and thus considered only GO terms to which 3–100 human genes were annotated. We associated each term with a differential activity score per tissue, which reflected the expression of its corresponding process in that tissue relative to other tissues (Sharon *et al*, 2022). Per tissue, the differential activity score of a term was set to the average log2 fold‐change values of its genes, where log2 fold‐change value of each gene was computed based on its expression in that tissue relative to other tissues (Basha *et al*, 2017; Sharon *et al*, 2022). Using these scores, we associated each gene with the set of terms to which it was annotated, and created four gene features per tissue, as follows: (i) The minimum differential activity score of the gene's terms in the given tissue. (ii) The maximum differential activity score of the gene's terms in the given tissue. (iii) The median differential activity score of the gene's terms in the given tissue. (iv) The mean differential activity score of the gene's terms in the given tissue. We included this set of potentially correlated features as we preferred to let the model decide which feature adds more information with respect to other dominant features in the model.

#### Data imputation

Imputation of missing values was achieved via a MICE‐inspired iterative imputation function (van Buuren & Groothuis‐Oudshoorn, 2011). Each feature with missing values was considered as the target of a regression model, while using the rest of the features dataset as training data. First, per feature, all missing values were imputed by the median value of the respective feature. Then, per feature, the imputed values were reassessed by a Bayesian ridge regression model that used the 100 nearest features as training. Nearest features were evaluated based on the absolute correlation coefficient between each feature and the target feature. The reassessment of each feature was iterated 10 times, such that each iteration was initialized with values assessed in the preceding iteration.

#### Data transformation and scaling

Unlike tree‐based models, logistic regression and neural network models could be confounded by nonsymmetrical distributions of data per feature. The main features that showed nonsymmetrical distributions were gene expression and preferential expression. These features were transformed to make them more symmetric by applying a Yeo‐Johnson power transformation (Yeo & Johnson, 2000). Transformation was applied after these data were used for the construction of other features and before applying the different machine learning (ML) models. Following transformation, each feature in the complete dataset was scaled per tissue to values between −1 and 1 while preserving the shape of its data distribution.

### The dataset of diseases, disease genes, and affected tissues

The set of Mendelian diseases was obtained from OMIM (McKusick‐Nathans Institute of Genetic Medicine, n.d.) and included only Mendelian diseases with a known molecular basis (OMIM Phenotype mapping key 3). The genes associated with each disease, denoted disease genes (also marked with Phenotype mapping key 3), were also retrieved from OMIM. Overall, our dataset included 3,924 disease genes.

The association between Mendelian diseases and their affected tissues (i.e., the tissue that clinically manifests the disease) was obtained from manually curated datasets (Barshir *et al*, 2018; Basha *et al*, 2020). There, tissues were considered as affected by a disease if they presented disease‐related clinical manifestations in most patients according to OMIM, HPO or the literature, and these manifestations were not secondary to disease sequela (e.g., we would exclude muscle wasting or contractures due to lack of ambulation, which results from severe neurological disease; for a detailed description, see (Hekselman *et al*, 2022)). Each disease gene whose disease affected a certain tissue was associated with that tissue, resulting 1,105 tissue‐associated disease genes.

#### The labeling of genes per tissue

To support the application of ML methods, we labeled genes per tissue *t* according to their association with *t*. Disease genes that were associated with *t* were labeled as positive for *t*; all other genes were labeled as negative for *t* (Dataset EV1). In the analyses per tissue model *t*, the set of disease genes that were associated with *t* was denoted “tissue‐associated disease genes.” The set of remaining Mendelian disease genes was denoted “other disease genes.” The set of “non‐disease genes” included all genes except for Mendelian disease genes.

#### The annotation of diseases to brain regions

We further curated brain disorders to brain regions. For this, we associated brain subregions that were sampled by GTEx with six distinct regions: Cortex (including anterior cingulate cortex (BA24), hippocampus, cortex, frontal cortex); cerebellum (including cerebellum, cerebellar hemisphere); basal ganglia (including caudate, nucleus accumbens, and putamen); spinal cord; hypothalamus; and amygdala. Next, we manually associated brain disorders with their affected brain region(s). Associations were based on anatomical findings per disease that were detailed in disease pages of OMIM (Amberger *et al*, 2019). We assigned each association with a confidence level between 3 (high) and 1 (low), as follows: Associations based on clinical synopsis of the disease were assigned a confidence level of 3, unless they were described as pertinent only to some of the patients, in which case they are assigned a confidence level of 2. Associations based on disease description were assigned a confidence level of 2. Associations based on clinical features, which in some cases described finding relevant to a small subset of the patients, were assigned a confidence level of 1. Brain diseases that were not associated with any of the above regions (i.e., were associated with a different region, or were associated with brain but not with a specific region) were defined as “Other.” The union of all diseases that were associated with any region was designated as “whole brain.” The resulting dataset appears in Dataset EV2. To support the application of ML methods, we labeled genes per brain region *b*. Disease genes whose disease manifests in *b* at a confidence level of 2 and above were labeled as positive for *b*; all other genes were labeled as negative for *b* (Dataset EV2).

### Using machine learning (ML) models to illuminate tissue‐selectivity features

Below we describe the ML method used for interpretability analysis, its application to specific genes and to all tissue‐associated disease genes, and the SHAP (SHapley Additive exPlanations) analysis of feature importance that was used to interpret the resulting models.

#### ML method for interpretability analysis

To create interpretable models, we used the gradient boosted tree (GBT) algorithm. GBT trains a sequence of logistic regression trees, where each successive tree aims to predict the pseudo‐residuals of the preceding trees assuming that the loss function is logistic loss. This method allows combining a huge number of shallow logistic regression trees by setting the learning rate to a small value. We employed the popular variant of GBT named “Extreme Gradient Boosting” (XGBoost, XGB), which is considered the state‐of‐the‐art algorithm for training GBT (Chen & Guestrin, 2016). We applied XGB to interpret the tissue selectivity of query genes, and the tissue selectivity of tissue‐associated disease genes for eight tissues, as described below. The input to XGB included the features dataset and the gene labels corresponding to the specific classification task. To reduce run time owing to the size of the features dataset and to reduce noise derived from noncontributing features, each application of XGB was preceded by feature selection that limited the number of relevant features per application to 50. Features were selected by applying support vector machines (SVM) with L1 regularization. We used SVM due to its capability to address high‐dimensional data. SVM was trained on, and fitted to, each relevant training set. By this, we selected different tailored sets of relevant features per application.

For more details on XGB implementation see “ML implementation details” below.

#### XGB application to specific genes and to all genes

To interpret the tissue selectivity of query genes (e.g., Fig 2), we trained an XGB model on all genes in our dataset except the query gene. For a query gene whose disease manifests in tissue *t*, disease genes that were associated with *t* were labeled as positive; other genes were labeled as negative. The query gene was then tested by the model.

To interpret the tissue selectivity of tissue‐associated disease genes for a tissue *t* (e.g., Fig 3A), we trained an XGB model on all genes in our features dataset. Disease genes that were associated with *t* were labeled as positive, all other genes were labeled as negative. We analyzed several tissues, however were able to create satisfactory performing models only for tissues with over 60 associated disease genes (positive genes). These tissues included blood, brain, heart, liver, nerve, skeletal muscle, skin and, testis. We tested the validity of the XGB model per tissue *t* by using 10‐fold cross‐validation (Appendix Fig S4).

#### SHAP analysis of feature importance

To identify the importance of the different features per model, we applied SHAP tree algorithm to trained XGB models using default parameters (preprint: Lundberg *et al*, 2018). To enable a summarized perspective on feature importance across different models, we normalized the SHAP feature values per model by dividing the value of each feature by the sum of values of all features in that model.

To identify recurrent patterns of features per model, we associated each feature with its feature type and with its tissue of origin. For example, the feature “brain preferential expression” was associated with “preferential expression” as its feature type and with “brain” as its tissue of origin. Tissues‐of‐origin that were not part of the eight modeled tissues were considered as “Other,” and their corresponding features were grouped together. Next, to identify recurrent feature types per model, we summed up the normalized SHAP values of features belonging to the same feature type. Likewise, to identify recurrent tissues‐of‐origin per model, we summed up the normalized SHAP values of features belonging to the same tissue of origin.

### The TRACE ML framework for prioritizing tissue‐associated disease genes

Below we describe the TRACE framework and the application of TRACE to predict genes that underlie tissue‐selective diseases.

#### The TRACE framework

TRACE was composed of stacking of two layers. The first layer of TRACE consisted of five ML methods for training classifiers, denoted as base learners. The ML methods included logistic regression (LR), the tree‐based ensemble methods XGB (described above), random forest (RF), and GBT (in the Scikit‐learn implementation) initiated by a logistic regression model (LR + GB), and a multilayer perceptron (MLP) with one hidden layer. The second layer of TRACE consisted of a meta‐learner MLP with two hidden layers.

In general, the input to TRACE included the features dataset and the gene labels corresponding to the specific classification task. Each base learner was applied independently to the input and produced a tissue‐association score per gene. Tissue‐association scores per base learner were scaled between 0 (not tissue‐associated) and 10 (tissue‐associated disease gene). The output of the five base learners was the input to the meta‐learner, which produced a final TRACE score per gene. The TRACE score was also scaled between 0 and 10.

To reduce run time owing to the size of the features dataset and to reduce noise derived from noncontributing features, all applications and folds of XGB, LR, and LR + GB were preceded by feature selection that limited the number of relevant features. Features were selected by applying SVM with L1 regularization and setting a feature contribution threshold. Specifically, we used the python scikit‐learn LinearSVC function with the C regularization parameter set to 0.1. SVM was trained on and fitted to each relevant training set, which resulted in sets of relevant features that were tailored per application and per fold. To guarantee that the SVM‐based feature selection will not entirely eliminate features that will contribute through nonlinear relationships, feature selection was not applied to RF and MLP, which inherently handle noncontributing features by not selecting trees that rely on them (RF) or minimizing their weights (MLP).

We also assessed the performance of TRACE upon using only features that were derived from transcriptomics and PPIs (a total of 594 features; network embedding features were excluded). Relative to the full TRACE models, TRACE models based only on transcriptomics and PPIs had a lower AUC in 7/8 cases and a lower PR in 6/8 cases (Appendix Fig S12). This demonstrates both the major value of tissue transcriptomes and PPIs and the added value of other features.

#### TRACE application to predict tissue‐associated disease genes

We applied TRACE to each tissue with over 60 associated disease genes, to predict tissue‐associated disease genes. Per tissue *t*, the input to TRACE included the features dataset and the gene labels corresponding to their association with that tissue (see “The labeling of genes per tissue” above). We assessed the validity of each of the base‐learner models and of the meta‐learner model by using 10‐fold cross‐validation (Appendix Fig S6). Specifically, all genes in our dataset were randomly partitioned into 10 disjoint subsets, while preserving the ratio of tissue‐associated to nonassociated genes across subsets. Per fold, prior to running the base learners XGB, LR, and LR + GB, we performed feature selection and then trained the models using the selected features. Per base learner, the probabilities of genes within each subset to be associated with *t* were computed once by using a model that was trained on genes in the other nine subsets. Next, the scores of the five base learners were used as features for the second‐level MLP meta‐learner model. The meta‐leaner model was trained on the scores of genes in the other nine subsets and was then applied to predict the final TRACE scores of the genes within the given subset.

To evaluate each model, we used the AUC, where each point on the curve corresponded to a particular cutoff, representing a trade‐off between sensitivity and specificity, and the auPRC that computes the weighted mean of precisions achieved at each cutoff of the precision–recall curve. False‐positive and false‐negative rates per model appear in Appendix Fig S13.

#### Comparison to pBRIT

We applied pBRIT to model each of the eight tissues that were modeled by TRACE (Kumar et al, 2018). To mimic the input to TRACE per tissue, pBRIT was applied to the same distinct subsets that were used in TRACE. pBRIT was run via its web interface, by using the data fusion method of “TFIDF.” Query gene labels were withheld from the regression.

### ML implementation details

All ML methods were implemented using the Scikit‐learn python package (Pedregosa *et al*, 2011), except for XGB, which was implemented using the Scikit‐learn API of the XGBoost package (Chen & Guestrin, 2016). Per ML method, all hyperparameters of the models were tuned manually to achieve higher AUC and auPRC scores. Since AUC and auPRC scores of the different tissue models showed small differences per method, tuning per method was done simultaneously for the eight tissue models. The same hyperparameter values were then applied to all tissue models. For tissue‐association models and for patient models, data contained mostly nondisease genes. To deal with class imbalance, the balance of positive to negative weights was set to 0.01 when training LR, XGB, RF, and the LR part of LR + GB.

#### XGB

The hyperparameters of XGBoost were set to build a decision forest consisting of 150 trees. Each tree had a maximum tree depth of nine. Gamma was set to 0. To prevent overfitting, we set the step size shrinkage (eta) to 0.1.

#### RF

The number of trees was set to 1,000.

#### LR

LR was used with a lbfgs solver and maximum of 100,000 iterations.

#### LR + GB

LR + GB was implemented by using the “GradientBoostingClassifier” function with LR parameters set with a lbfgs solver and maximum of 100,000 iterations and consisting of 80 trees.

#### MLP

The base‐learner MLP was implemented by using the “MLPClassifier” function with two hidden layers of size 10 each and ReLU activation function. Alpha was set to 0.5. Batch size was set to 200. The meta‐learner MLP was similarly implemented except that alpha was set to 0.1 and the learning rate was adaptive and initiated at 0.01.

#### SVM

SVM was implemented by “LinearSVC” function with a maximum of 10,000 iterations. For interpretability models, C was set to 2 and number of features was limited to 50. For TRACE prediction models, C was set to 0.1.

### Analysis of tissue selectivity for distinct brain regions

We focused on the subset of diseases that were associated with brain regions with a confidence level ≥ 2 and on brain regions that were associated with at least 60 disease genes. TRACE analysis was similar to the analysis of other tissues. To assess the selectivity of diseases to a specific brain region, we compared between the scores of genes that were associated with the specific region (confidence level ≥ 2), the scores of all other brain‐associated disease genes (confidence level of 3), and the scores of brain‐associated disease genes that were not annotated to a specific brain region (denoted as “other” in the manually curated dataset, confidence level of 2).

### The application of TRACE to prioritize candidate disease genes in patients with rare tissue‐selective diseases

#### The criteria for selecting patient cases

All cases corresponded to genetic diseases with Mendelian inheritance that were investigated by the lab of co‐author Prof. Ohad Birk. We focused on cases whose affected tissue could be modeled by TRACE, including cases presenting neurological and developmental abnormalities (modeled as brain), skin disease, muscle disease, cardiac disease, and azoospermia (modeled as testis). All cases had extensive clinical data available, undergone NGS investigations using modern techniques, high‐quality data files were available, and were published. The pathogenic variant in each patient was previously successfully identified following genetic and functional analyses (Dataset EV3A).

#### Identification of candidate variants per patient

Patients were previously genetically diagnosed via exome sequencing and subsequent analysis, as previously described (Yogev *et al*, 2017; Drabkin *et al*, 2018; Wormser *et al*, 2019). The data per patient were deidentified, and variants were filtered as follows:

- Kept variants with call quality at least 20.0 in cases or at least 20.0 in controls AND outside top 5.0% most exonically variable 100 base windows in healthy public genomes (1,000 genomes).
- Excluded variants that were observed with an allele frequency greater than or equal to 0.5% of the genomes in the 1000 genomes project OR greater than or equal to 0.5% of the NHLBI ESP exomes (All); or greater than or equal to 0.5% of the ExAC Frequency; or greater than or equal to 0.5% of the gnomAD Frequency; or filter variants unless established pathogenic common variant.
- Kept variants (up to 20 bases into intron) that were experimentally observed to be associated with a phenotype: Pathogenic, possibly pathogenic or disease‐associated according to HGMD; or clinically relevant variants from CentoMD; or frameshift, in‐frame indel, or stop codon change, or missense, or predicted deleterious by having CADD score > 15.0; or predicted to disrupt splicing by MaxEntScan; or within 2 bases into intron.
- In case of dominant genes, kept variants which are associated with gain of function, or hemizygous, or heterozygous, or heterozygous‐amb, or compound heterozygous, or homozygous, or heterozygous‐alt, or haploinsufficient and occur in at least one of the Case samples at the variant level; and not variants which are associated with gain of function, or hemizygous, or heterozygous, or heterozygous‐amb, or compound heterozygous, or homozygous, or heterozygous‐alt, or haploinsufficient, and occur in at least one of the control samples at the variant level in the control samples.

In case of autosomal recessive genes, kept variants which are hemizygous, or compound heterozygous, or haploinsufficient, or homozygous, and occur in at least one of the case samples at the gene level in the Case samples; and not variants which are hemizygous, or compound heterozygous, or haploinsufficient, or homozygous, and occur in at least 1 of the control samples at the variant level in the control samples.

Analyses were based on Ingenuity Variant Analysis version 5.4.20181019. Content versions: CADD (v1.3), Allele Frequency Community (2018‐09‐06), EVS (ESP6500SI‐V2), Refseq Gene Model (2018‐07‐10), JASPAR (2013‐11), Ingenuity Knowledge Base Snapshot Timestamp (2019‐01‐06 00:23:50.0), Vista Enhancer (2012‐07), Clinical Trials (Stepford 190106.000), PolyPhen‐2 (v2.2.2), 1000 Genome Frequency (phase3v5b), ExAC (0.3.1), iva (Oct 4 11:04 iva‐1.0.736.jar), PhyloP (2009‐11), DbSNP (151), TargetScan (6.2), GENCODE (Release 28), CentoMD (5.0), Ingenuity Knowledge Base (Stepford 190106.000), OMIM (May 26, 2017), gnomAD (2.0.1), BSIFT (2016‐02‐23), TCGA (2013‐09‐05), Clinvar (2018‐08‐01), DGV (2016‐05‐15), COSMIC (v86), HGMD (2018.3), and SIFT4G (2016‐02‐23).

#### TRACE analysis of patient's candidate disease genes

The data per patient were deidentified and included (i) the disease‐affected tissue of the patients, and (ii) a list of variants identified in that patient that remained after the filtering described in the preceding section. Each variant was associated with its respective gene, denoted henceforth as candidate disease gene. Per patient, we created a TRACE model for each of her disease‐affected tissues. Five of the 48 patients had two affected tissues, resulting in 53 ranked cases. To ensure that the model is not trained and tested on the same genes, the patient's candidate disease genes were entirely withheld from the features dataset, in order to be later prioritized by an independently trained TRACE model. The remaining, noncandidate genes, were labeled according to their association with the patient's affected tissue. In the first layer of TRACE, TRACE base learners predicted the tissue association of all noncandidate genes through 10‐fold cross‐validation procedure. Each base learner then predicted the tissue association of the patient's candidate disease genes by training a model on all noncandidate genes. In the second layer of TRACE, the scores of the base learners were used (once, no cross‐validation), as features for the TRACE meta‐learner. The meta‐learner was trained on all noncandidate genes and was then used to predict the TRACE scores of the patient's candidate disease genes.

#### Summary of TRACE results across patients

Per patient, we ranked patient's candidate disease genes by their TRACE scores. Next, we associated the verified disease gene of the patient with its rank.

#### Comparison to prioritization by other methods

In the prioritization by expression levels, we ranked the candidate genes of each patient by their expression level in the modeled tissue. In the prioritization by GADO (Deelen *et al*, 2019), GADO prioritizes genes according to their similarity to genes associated with a user‐selected Human Phenotype Ontology (HPO) term. Per patient, we selected the HPO term(s) corresponding to the patient's phenotypes and the disease gene information in OMIM, unless GADO recommended a different, typically more generic, HPO term (Dataset EV3). We then ranked genes according to GADO output. In the prioritization by gnomAD (Karczewski *et al*, 2020), we downloaded from gnomAD the pLoF LOEUF score and the missense score of each gene (gnomAD download page, Table “pLoF Metrics by gene TSV,” downloaded on May 1, 2022). We used the upper bound scores (oe_lof_upper, oe_mis_upper) as recommended by gnomAD. We ranked the genes of each patient once by their pLoF score and once by the missense score. Per method, we associated the verified disease gene of each patient with its rank and compared the ranking by the method to the ranking by TRACE by using the Wilcoxon signed‐rank test.

#### Analysis of top five candidate genes per patient

Per patient, we collected the top five candidate genes according to TRACE (265 genes total). We then checked how many of them participated in a gene ontology (GO) biological process that was specific to the disease‐affected tissue of the patient and hence could lead to tissue‐selective phenotypes. Data of tissue‐specific GO biological processes were obtained from Sharon *et al* (2022).

### Statistical tests

To test the null hypothesis that TRACE scores of two distinct gene sets have similar probabilities to be smaller or greater than the other, we used the Mann–Whitney *U* test. Correction for multiple hypothesis testing was done via the Benjamini–Hochberg procedure. To test the null hypothesis that ranking by expression, by GADO (Deelen *et al*, 2019), or by gnomAD scores (Karczewski *et al*, 2020) was better or equal to TRACE ranking, we used Wilcoxon signed‐rank test.

### TRACE webserver

The TRACE webserver was implemented in Python by using the Flask framework with data stored on a MySQL database. The website client was developed using the ReactJS framework and designed with Semantic‐UI. The charts were displayed by the Google Charts library. The TRACE webserver supports all major browsers. The webserver presents the TRACE scores of input genes in the user‐selected tissue. TRACE scores were based on 10‐fold cross‐validation and computed as described in section “TRACE application to predict tissue‐associated disease genes.”

## Author contributions

**Eyal Simonovsky:** Conceptualization; formal analysis; investigation; methodology; writing – original draft; writing – review and editing. **Moran Sharon:** Data curation; formal analysis; validation; investigation; methodology; writing – original draft; writing – review and editing. **Maya Ziv:** Data curation; formal analysis; investigation; methodology. **Omry Mauer:** Software; visualization. **Idan Hekselman:** Data curation; investigation. **Juman Jubran:** Formal analysis; investigation. **Ekaterina Vinogradov:** Visualization. **Chanan M Argov:** Investigation. **Omer Basha:** Resources; software. **Lior Kerber:** Data curation. **Yuval Yogev:** Resources; formal analysis; validation; investigation; writing – original draft; writing – review and editing. **Ayellet V Segrè:** Resources; software; methodology. **Hae Kyung Im:** Resources; software. **Ohad Birk:** Resources. **Lior Rokach:** Methodology; writing – original draft; writing – review and editing. **Esti Yeger‐Lotem:** Conceptualization; resources; supervision; validation; investigation; methodology; writing – original draft; project administration; writing – review and editing.

In addition to the CRediT author contributions listed above, the contributions in detail are:

Conceptualization: ES and EY‐L; Methodology: ES, LR and EY‐L; Investigation: ES, MS, MZ, JJ, CMA; Formal analysis: ES, MS, MZ, JJ, CMA, YY; Webserver: OM, OB; Visualization: EV; Data acquisition: IH, LK; Data contribution: GC, AVS, HKI, YY, OB; Writing: ES, EYL; Supervision: EYL; Funding acquisition: LR, EY‐L.

## Disclosure and competing interests statement

The authors declare that they have no conflict of interest.

## Supporting information



Appendix

Expanded View Figures PDF

Dataset EV1

Dataset EV2

Dataset EV3

PDF+

## Data Availability

All data are available as EV Datasets and in https://sandbox.zenodo.org/record/1185590#.ZES8M3ZByUk. The code for running TRACE is available in https://github.com/eyalsim/trace.
