# Supplementary material for: Predicting molecular mechanisms of hereditary diseases by using their tissue‐selective manifestation
Source: Mol Syst Biol. 2023 May 26;19(8):e11407. doi: 10.15252/msb.202211407 (PMC10407743; doi:10.15252/msb.202211407)
Supplement: Supplementary file 1 — Appendix [file MSB-19-e11407-s007.pdf]

APPENDIX: Predicting molecular mechanisms of hereditary diseases by using their tissue-selective manifestation

Eyal Simonovsky, Moran Sharon, Maya Ziv, Omry Mauer, Idan Hekselman, Juman Jubran, Ekaterina Vinogradov, Chanan M. Argov, Omer Basha, Lior Kerber, Yuval Yogev, Ayellet V. Segrè, Hae Kyung Im, GTEx Consortium, Ohad Birk, Lior Rokach, Esti Yeger-Lotem

Contents

This file contains Appendix Figures S1-13 and Appendix Table S1

Appendix Figure S1 ………….. 2

Appendix Figure S2 ………….. 3

Appendix Figure S3 ………….. 4

Appendix Figure S4 ………….. 6

Appendix Figure S5 ………….. 7

Appendix Figure S6 ………….. 9

Appendix Figure S7 ………….. 10

Appendix Figure S8 ………….. 11

Appendix Figure S9 ………….. 12

Appendix Figure S10 ………….. 13

Appendix Figure S11 ………….. 14

Appendix Figure S12 ………….. 15

Appendix Figure S13 ………….. 16

Appendix Table S1 ………….. 18

Membership of the GTEx Consortium … 22

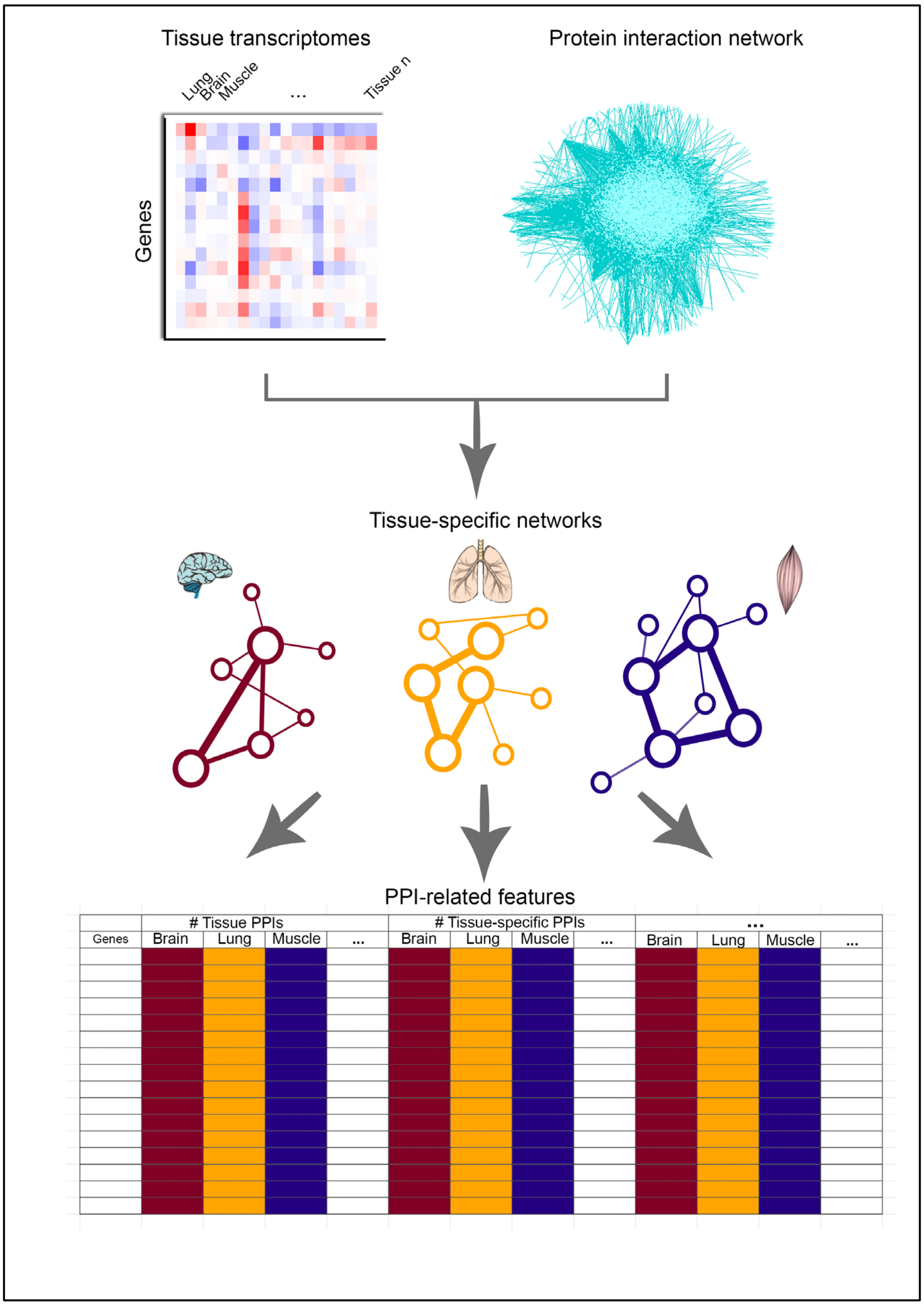

Appendix Fig. S1. The generation of gene features by integrating protein-protein interactions (PPI) and tissue transcriptomes. Tissue transcriptomes were combined with data of experimentally-detected PPI between human proteins to create tissue-specific PPI networks. Each tissue-specific network included the subset of tissue-expressed genes and the PPIs of their protein products (node size and edge width reflect the relative expression levels of corresponding genes). The resulting networks gave rise to multiple gene-based features per tissue network. For example, # tissue PPIs counts the number of gene-related PPIs in that tissue network; # tissue-specific PPIs counts the number of gene-related PPIs in that tissue network that do not appear in most other tissue networks.

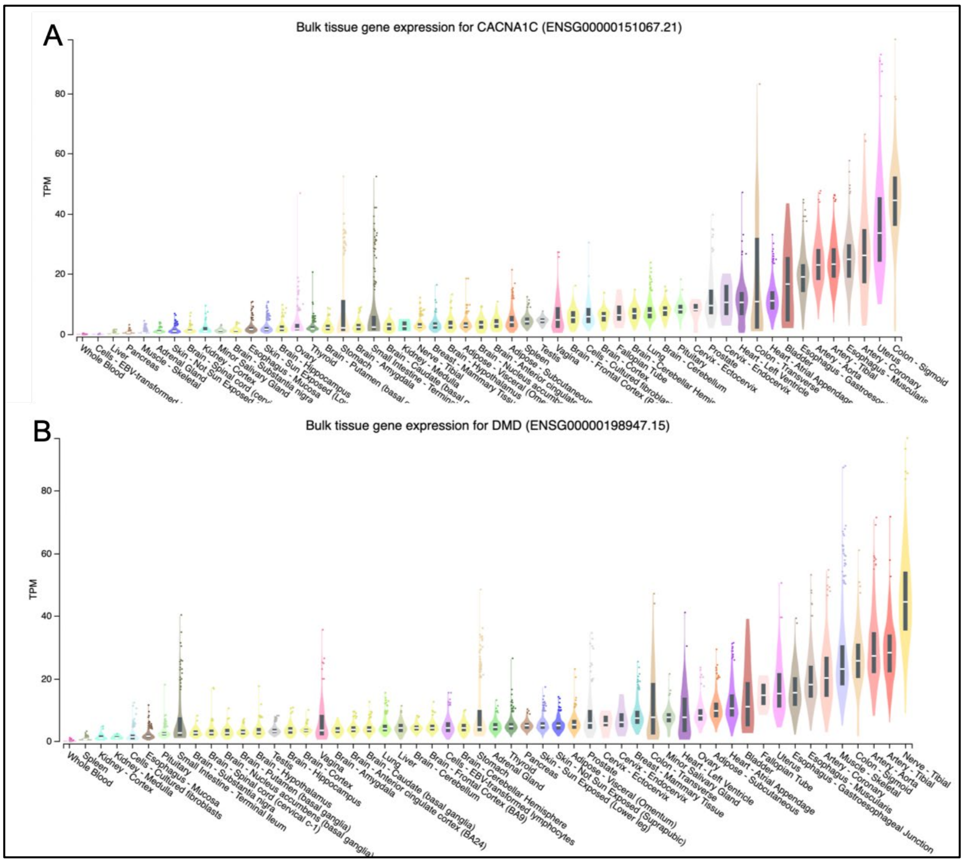

Appendix Fig. S2. The expression levels of CACNA1C and DMD in adult human tissues. Figures were obtained from the GTEx portal (https://www.gtexportal.org/home/), with tissues ordered according to the median expression level of the query gene. The median expression level of CACNA1C and DMD were above 1TPM in over 80% of the tissues. The median expression level of CACNA1C in heart, its disease-affected tissue, was 25% of its maximal median expression level in any tissue. The median expression level of DMD in skeletal muscle, its disease-affected tissue, was 52% of its maximal median expression level in any tissue.

A

The gene CACNA1C is associated with 18 processes:

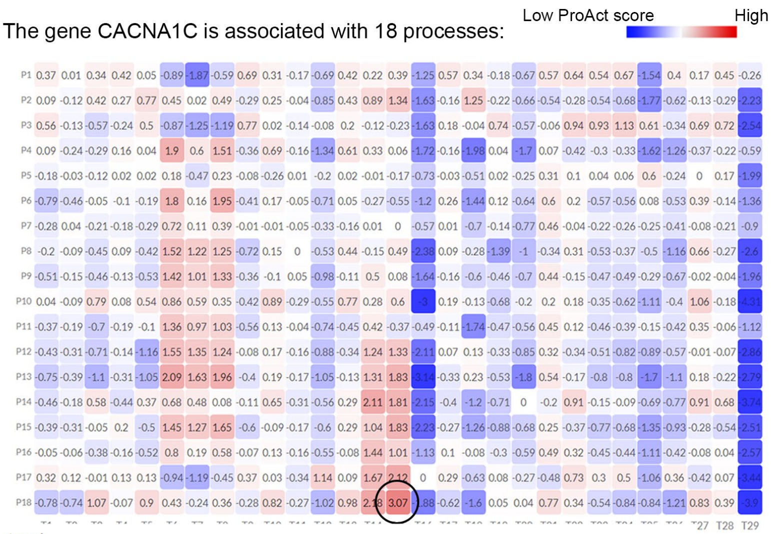

Legend

Tissue index	Tissue name	Tissue/Sub-tissue name
T1	Adipose0	Adipose-Subcutaneous
T2	Adipose1	Adipose-Visceral (Omentum)
T3	Artery0	Artery-Aorta
T4	Artery1	Artery-Coronary
T5	Artery2	Artery – Tibial
T6	Brain0	Spinal cord (cervical c-1), Hypothalamus, Hippocampus, Frontal cortex (BA9), Cortex, Anterior cingulate cortex (BA24), Amygdala
T7	Brain1	Cerebellum, Cerebellar Hemisphere
T8	Brain2	Putamen (basal ganglia), Nucleus Accumbens (basal ganglia), Caudate (basal ganglia)
T9	Breast-Mammary Tissue	Breast - Mammary Tissue
T10	Colon-Sigmoid	Colon-Sigmoid
T11	Esophagus0	Esophagus - Gastroesophageal Junction
T12	Esophagus1	Esophagus – Mucosa
T13	Esophagus2	Esophagus – Muscularis
T14	Heart0	Heart - Atrial Appendage
T15	Heart1	Heart - Left Ventricle
T16	Liver	Liver
T17	Lung	Lung
T18	Muscle - Skeletal	Muscle – Skeletal
T19	Nerve - Tibial	Nerve – Tibial
T20	Ovary	Ovary
T21	Pituitary	Pituitary
T22	Prostate	Prostate
T23	Skin0	Skin - Not Sun Exposed (Suprapubic)
T24	Skin1	Skin-Sun Exposed (Lower leg)
T25	Testis	Testis
T26	Thyroid	Thyroid
T27	Uterus	Uterus
T28	Vagina	Vagina
T29	Whole Blood	Whole Blood

Process index	Process GO name	GO term accession
P1	immune system development	GO:0002520
P2	regulation of cardiac muscle contraction by regulation of the release of sequestered calcium ion	GO:0010881
P3	embryonic forelimb morphogenesis	GO:0035115
P4	calcium-mediated signaling using extracellular calcium source	GO:0035585
P5	camera-type eye development	GO:0043010
P6	positive regulation of adenylate cyclase activity	GO:0045762
P7	regulation of insulin secretion	GO:0050796
P8	calcium ion transport into cytosol	GO:0060402
P9	cardiac conduction	GO:0061337
P10	calcium ion transmembrane transport via high voltage-gated calcium channel	GO:0061577
P11	calcium ion import	GO:0070509
P12	cardiac muscle cell action potential involved in contraction	GO:0086002
P13	membrane depolarization during cardiac muscle cell action potential	GO:0086012
P14	membrane depolarization during AV node cell action potential	GO:0086045
P15	cell communication by electrical coupling involved in cardiac conduction	GO:0086064
P16	regulation of heart rate by cardiac conduction	GO:0086091
P17	regulation of ventricular cardiac muscle cell action potential	GO:0098911
P18	membrane depolarization during atrial cardiac muscle cell action potential	GO:0098912

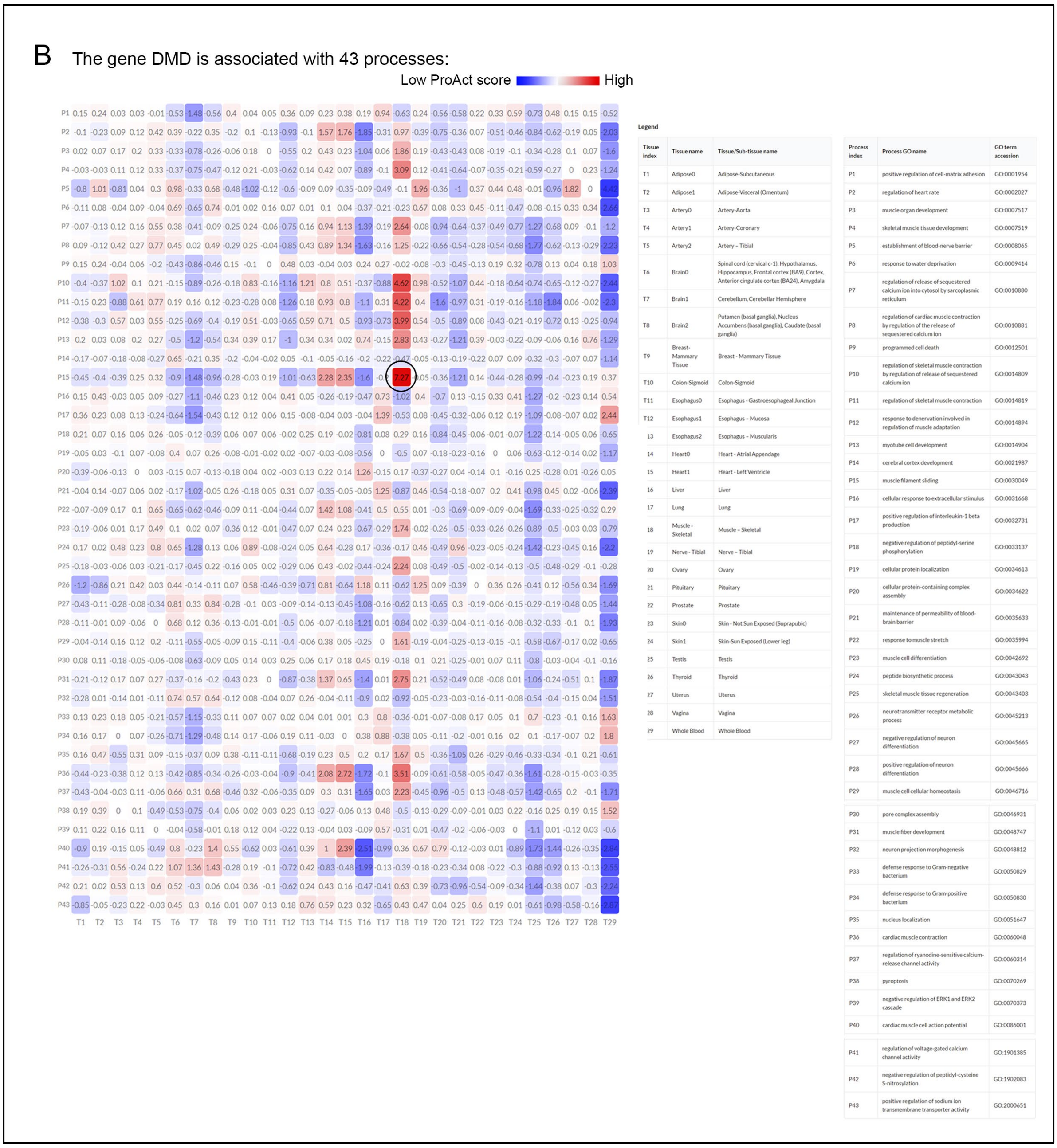

Appendix Fig. S3. TRACE predictions illuminate disease-related mechanisms.

A. The arrhythmia gene CACNA1C was annotated by GO to 18 processes. The heatmap shows the differential activity of these processes in 29 main tissues (red and blue denote high and low activity, respectively) (Sharon *et al*, 2022). The most highly active process was 'membrane depolarization during atrial cardiac muscle cell action potential' in heart left ventricle (circled), in accordance with arrhythmia phenotypes.

B. The Duchenne muscular dystrophy gene DMD was annotated by GO to 43 processes. The heatmap shows the differential activity of these processes in 29 main tissues (red and blue denote high and low activity, respectively) (Sharon *et al.*, 2022). The most highly active process was 'muscle filament sliding' in skeletal muscle (circled), in accordance with Duchenne phenotypes and the impairment observed in mdx mouse model for Duchenne.

Figures were obtained from https://netbio.bgu.ac.il/ProAct/

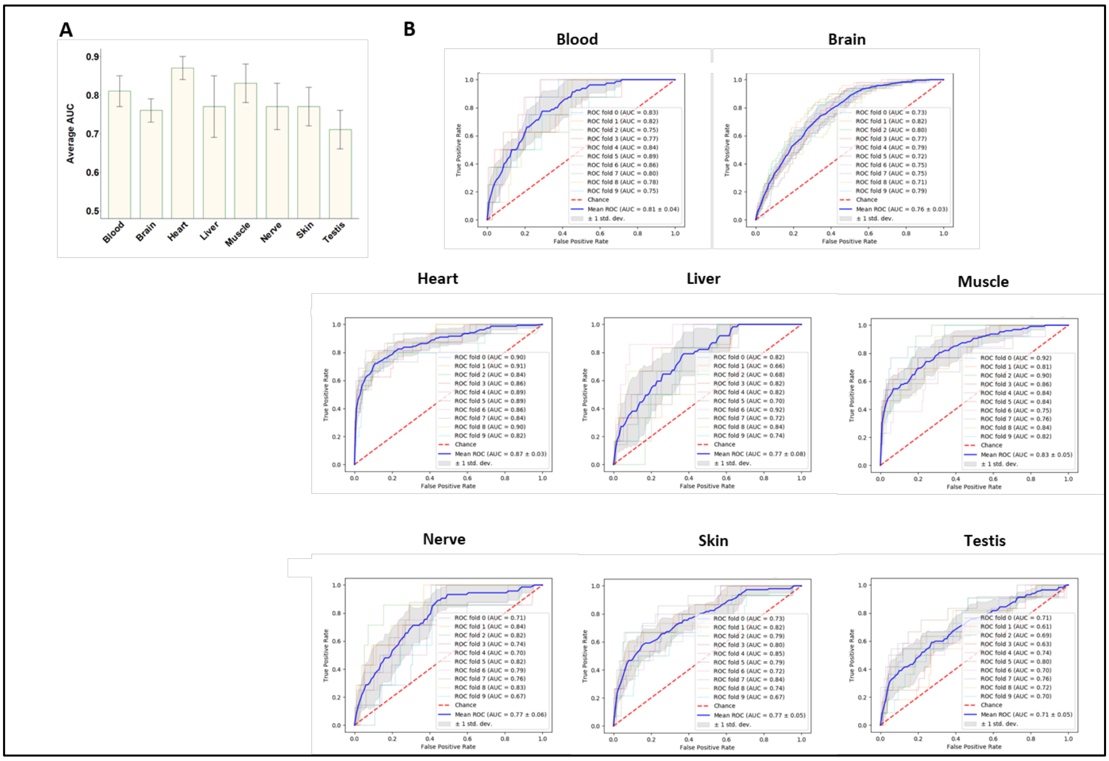

Appendix Fig. S4. Application of XGB to tissue models.

A. The average area under the receiver operating characteristic curve (AUC) obtained per tissue model following 10-fold cross-validation.

B. The receiver operating characteristic curves obtained per tissue model following 10-fold cross-validation.

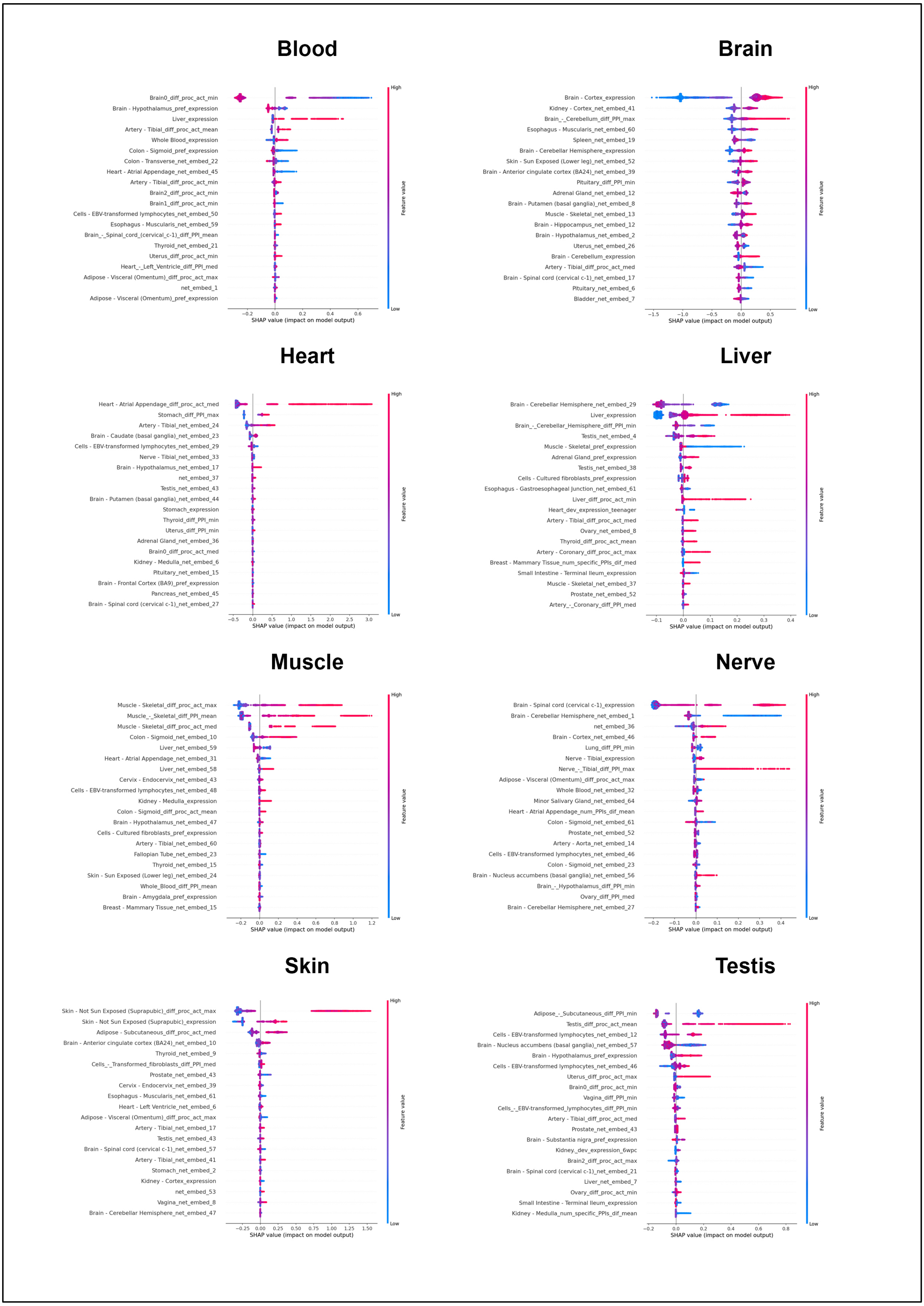
Blood
Brain0_diff_proc_act_min
Brain - Hypothalamus_pref_expression
Liver_expression
Artery - Tibial_diff_proc_act_mean
Whole Blood_expression
Colon - Sigmoid_pref_expression
Colon - Transverse_net_embed_22
Heart - Atrial Appendage_net_embed_45
Artery - Tibial_diff_proc_act_min
Brain2_diff_proc_act_min
Brain1_diff_proc_act_min
Cells - EBV-transformed lymphocytes_net_embed_50
Esophagus - Muscularis_net_embed_59
Brain_-_Spinal_cord_(cervical_c-1)_diff_PPI_mean
Thyroid_net_embed_21
Uterus_diff_proc_act_min
Heart_-_Left_Ventricle_diff_PPI_med
Adipose - Visceral (Omentum)_diff_proc_act_max
net_embed_1
Adipose - Visceral (Omentum)_pref_expression
SHAP value (impact on model output)
Feature value
High
Low
Brain
Brain - Cortex_expression
Kidney - Cortex_net_embed_41
Brain_-_Cerebellum_diff_PPI_max
Esophagus - Muscularis_net_embed_60
Spleen_net_embed_19
Brain - Cerebellar Hemisphere_expression
Skin - Sun Exposed (Lower leg)_net_embed_52
Brain - Anterior cingulate cortex (BA24)_net_embed_39
Pituitary_diff_PPI_min
Adrenal Gland_net_embed_12
Brain - Putamen (basal ganglia)_net_embed_8
Muscle - Skeletal_net_embed_13
Brain - Hippocampus_net_embed_12
Brain - Hypothalamus_net_embed_2
Uterus_net_embed_26
Brain - Cerebellum_expression
Artery - Tibial_diff_proc_act_med
Brain - Spinal cord (cervical c-1)_net_embed_17
Pituitary_net_embed_6
Bladder_net_embed_7
SHAP value (impact on model output)
Heart
Heart - Atrial Appendage_diff_proc_act_med
Stomach_diff_PPI_max
Artery - Tibial_net_embed_24
Brain - Caudate (basal ganglia)_net_embed_23
Cells - EBV-transformed lymphocytes_net_embed_29
Nerve - Tibial_net_embed_33
Brain - Hypothalamus_net_embed_17
net_embed_37
Testis_net_embed_43
Brain - Putamen (basal ganglia)_net_embed_44
Stomach_expression
Thyroid_diff_PPI_min
Uterus_diff_PPI_min
Adrenal Gland_net_embed_36
Brain0_diff_proc_act_med
Kidney - Medulla_net_embed_6
Pituitary_net_embed_15
Brain - Frontal Cortex (BA9)_pref_expression
Pancreas_net_embed_45
Brain - Spinal cord (cervical c-1)_net_embed_27
SHAP value (impact on model output)
Liver
Brain - Cerebellar Hemisphere_net_embed_29
Liver_expression
Brain_-_Cerebellar_Hemisphere_diff_PPI_min
Testis_net_embed_4
Muscle - Skeletal_pref_expression
Adrenal Gland_pref_expression
Testis_net_embed_38
Cells - Cultured fibroblasts_pref_expression
Esophagus - Gastroesophageal Junction_net_embed_61
Liver_diff_proc_act_min
Heart_dev_expression_teenager
Artery - Tibial_diff_proc_act_med
Ovary_net_embed_8
Thyroid_diff_proc_act_mean
Artery - Coronary_diff_proc_act_max
Breast - Mammary Tissue_num_specific_PPIs_dif_med
Small Intestine - Terminal Ileum_expression
Muscle - Skeletal_net_embed_37
Prostate_net_embed_52
Artery_-_Coronary_diff_PPI_med
SHAP value (impact on model output)
Muscle
Muscle - Skeletal_diff_proc_act_max
Muscle_-_Skeletal_diff_PPI_mean
Muscle - Skeletal_diff_proc_act_med
Colon - Sigmoid_net_embed_10
Liver_net_embed_59
Heart - Atrial Appendage_net_embed_31
Liver_net_embed_58
Cervix - Endocervix_net_embed_43
Cells - EBV-transformed lymphocytes_net_embed_48
Kidney - Medulla_expression
Colon - Sigmoid_diff_proc_act_mean
Brain - Hypothalamus_net_embed_47
Cells - Cultured fibroblasts_pref_expression
Artery - Tibial_net_embed_60
Fallopian Tube_net_embed_23
Thyroid_net_embed_15
Skin - Sun Exposed (Lower leg)_net_embed_24
Whole_Blood_diff_PPI_mean
Brain - Amygdala_pref_expression
Breast - Mammary Tissue_net_embed_15
SHAP value (impact on model output)
Nerve
Brain - Spinal cord (cervical c-1)_expression
Brain - Cerebellar Hemisphere_net_embed_1
net_embed_36
Brain - Cortex_net_embed_46
Lung_diff_PPI_min
Nerve - Tibial_expression
Nerve_-_Tibial_diff_PPI_max
Adipose - Visceral (Omentum)_diff_proc_act_max
Whole Blood_net_embed_32
Minor Salivary Gland_net_embed_64
Heart - Atrial Appendage_num_PPIs_dif_mean
Colon - Sigmoid_net_embed_61
Prostate_net_embed_52
Artery - Aorta_net_embed_14
Cells - EBV-transformed lymphocytes_net_embed_46
Colon - Sigmoid_net_embed_23
Brain - Nucleus accumbens (basal ganglia)_net_embed_56
Brain_-_Hypothalamus_diff_PPI_min
Ovary_diff_PPI_med
Brain - Cerebellar Hemisphere_net_embed_27
SHAP value (impact on model output)
Skin
Skin - Not Sun Exposed (Suprapubic)_diff_proc_act_max
Skin - Not Sun Exposed (Suprapubic)_expression
Adipose - Subcutaneous_diff_proc_act_med
Brain - Anterior cingulate cortex (BA24)_net_embed_10
Thyroid_net_embed_9
Cells_-_Transformed_fibroblasts_diff_PPI_med
Prostate_net_embed_43
Cervix - Endocervix_net_embed_39
Esophagus - Muscularis_net_embed_61
Heart - Left Ventricle_net_embed_6
Adipose - Visceral (Omentum)_diff_proc_act_max
Artery - Tibial_net_embed_17
Testis_net_embed_43
Brain - Spinal cord (cervical c-1)_net_embed_57
Artery - Tibial_net_embed_41
Stomach_net_embed_2
Kidney - Cortex_expression
net_embed_53
Vagina_net_embed_8
Brain - Cerebellar Hemisphere_net_embed_47
SHAP value (impact on model output)
Testis
Adipose_-_Subcutaneous_diff_PPI_min
Testis_diff_proc_act_mean
Cells - EBV-transformed lymphocytes_net_embed_12
Brain - Nucleus accumbens (basal ganglia)_net_embed_57
Brain - Hypothalamus_pref_expression
Cells - EBV-transformed lymphocytes_net_embed_46
Uterus_diff_proc_act_max
Brain0_diff_proc_act_min
Vagina_diff_PPI_min
Cells_-_EBV-transformed_lymphocytes_diff_PPI_min
Artery - Tibial_diff_proc_act_med
Prostate_net_embed_43
Brain - Substantia nigra_pref_expression
Kidney_dev_expression_6wpc
Brain2_diff_proc_act_max
Brain - Spinal cord (cervical c-1)_net_embed_21
Liver_net_embed_7
Ovary_diff_proc_act_min
Small Intestine - Terminal Ileum_expression
Kidney - Medulla_num_specific_PPIs_dif_mean
SHAP value (impact on model output)

Appendix Fig. S5. SHAP assessment of the contribution of features to tissue models based on XGB. The 20 topmost contributing features to each tissue model. Features were ordered from bottom to top by their increased absolute contribution to the model, allowing interpretation. Per feature, each dot represents the feature value of a different gene; red and blue denote high and low values of the feature, respectively. Dots were spread from left to right by their contribution to prediction of the gene as leading to a disease that manifests in the modeled tissue (left) or not (left). Diff_net = differential network; diff_proc_act = differential process activity.

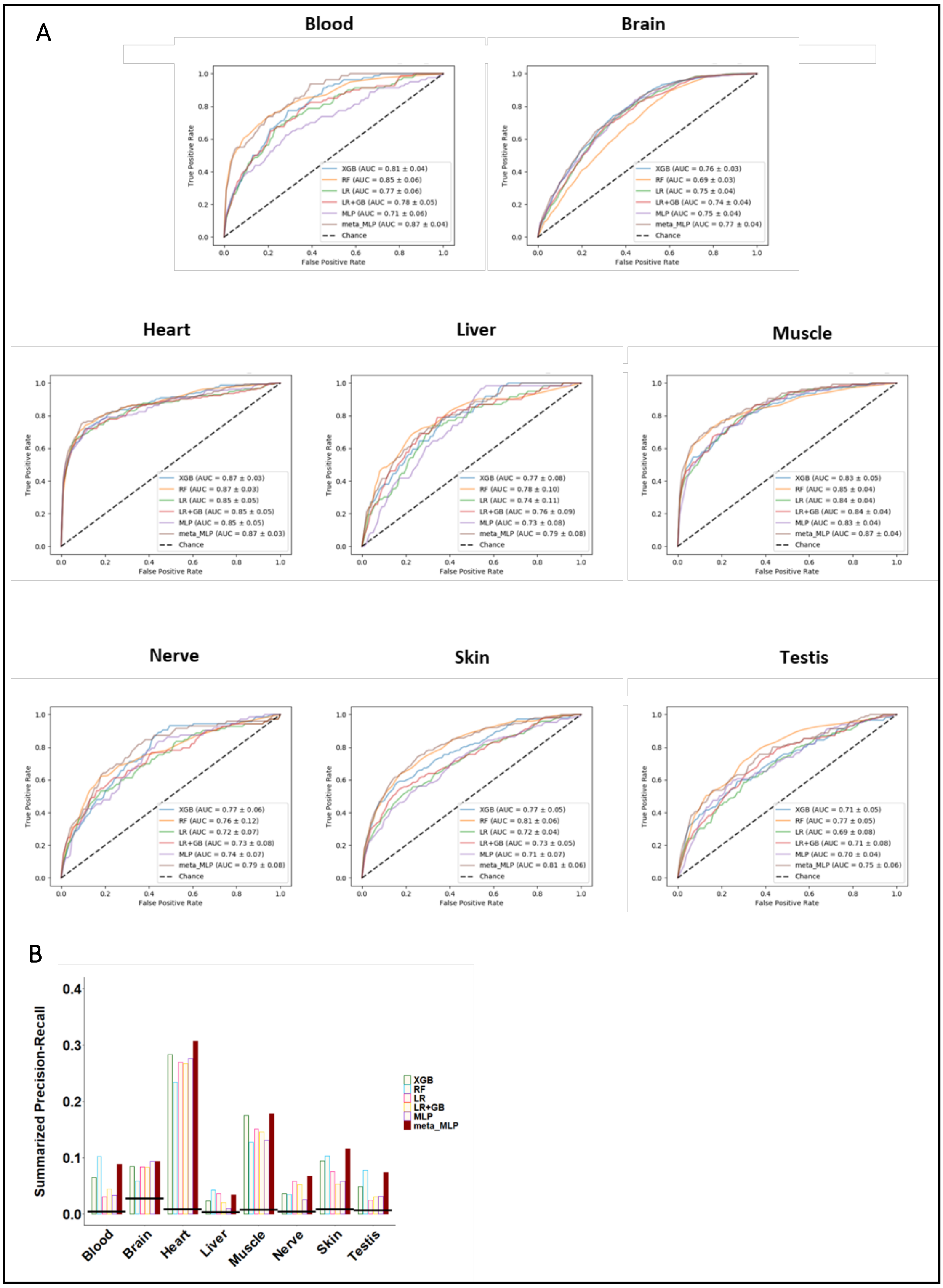

Appendix Fig. S6. The performance of the different classifiers in tissue models.

A. average ROC curves obtained per ML method and tissue model following 10-fold cross-validation.

B. The summarized precision recall scores obtained per ML method and tissue model following 10-fold cross-validation. Black horizontal lines depict the random prediction baseline, calculated as positive-to-negative ratio.

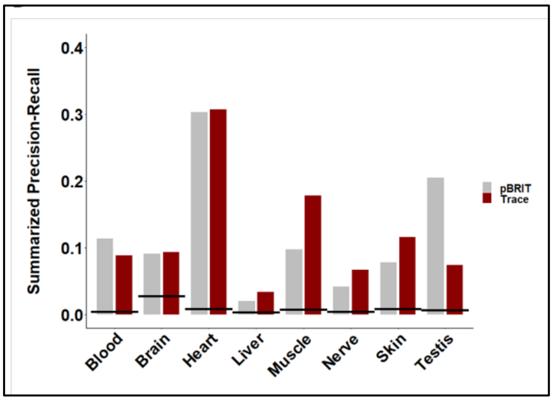

Appendix Fig. S7. The summarized precision recall scores obtained by pBRIT and TRACE per tissue model. Performance was assessed via 10-fold cross-validation. The exact same folds were used. The scores of TRACE were higher in 6/8 tissue models. Black horizontal lines depict the random prediction baseline, calculated as positive-to-negative ratio.

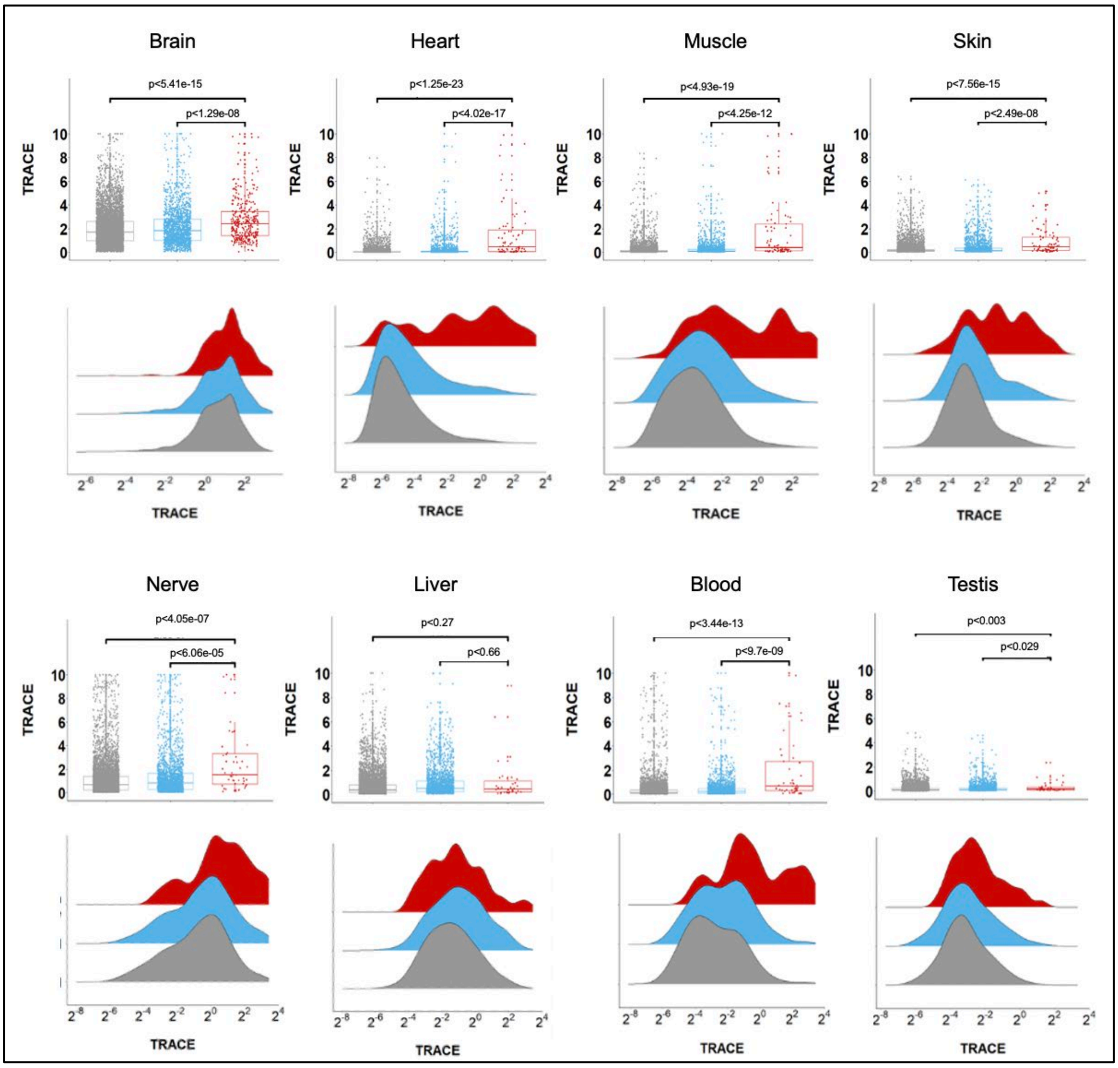

Appendix Fig. S8. TRACE prioritization of non-tissue-specific genes associated with diseases that manifest in distinct tissues. This analysis included 8,749 genes that were expressed in at least 80% of the tissues. The figure shows gene TRACE scores in the different tissue models as dot and density ridge plots. Each dot in a dot plot represents the TRACE score of a different gene. Genes were divided into genes that are causal for a disease that manifests in the modeled tissue (tissue-associated, red), genes that are causal for a disease that does not manifest in the modeled tissue (other disease genes, blue), and non-disease genes (grey). Tissue-associated disease genes had significantly higher TRACE scores compared to non-disease genes and to other disease genes in all tissue models except for liver (Mann-Whitney U test, adjusted p-values are shown).

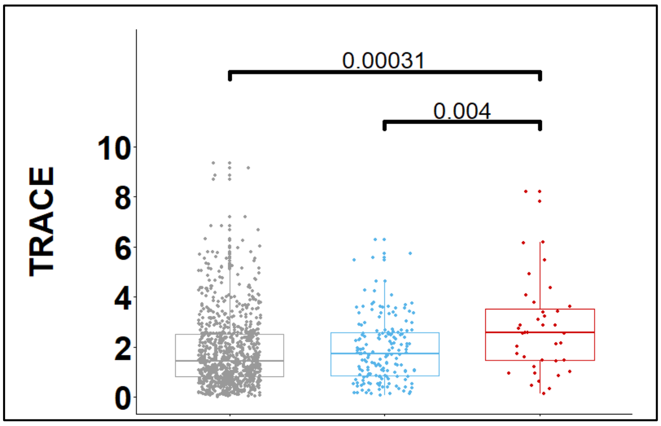

Appendix Fig. S9. TRACE prioritization of non-overexpressed genes. This analysis included 1,094 non-preferentially expressed genes (preferential expression <2 across all tissues), of which 208 were disease genes and 72 were associated with the tissues that we modeled. Results are shown for the brain tissue model, since brain was the only modeled tissue with ≥10 tissue-associated disease genes (specifically 38). Genes were divided into genes that are causal for a disease that manifests in brain (red), genes that are causal for a disease that does not manifest in brain (blue), and non-disease genes (grey). Brain-associated disease genes had significantly higher TRACE scores compared to non-disease genes and to other disease genes (Mann-Whitney U test).

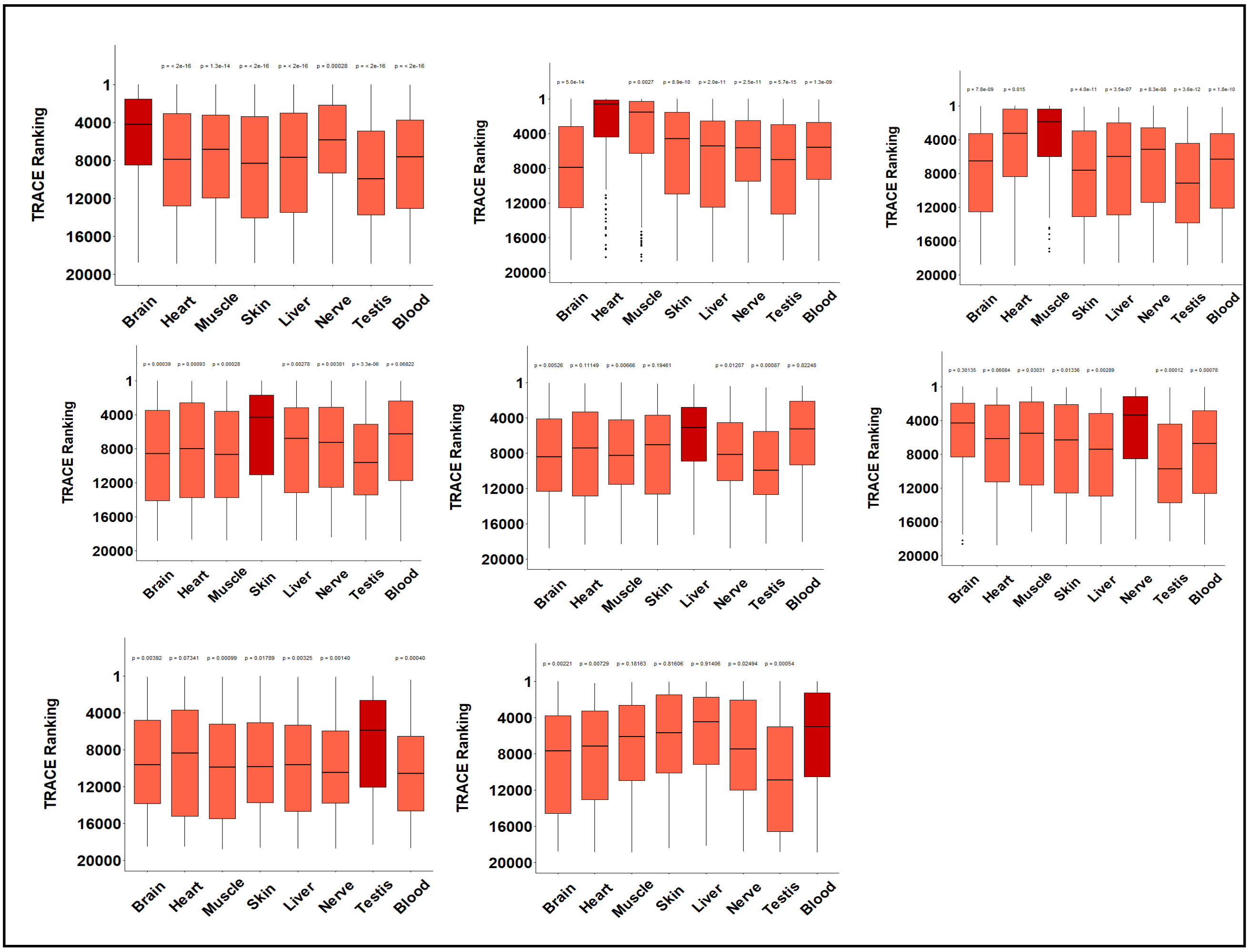

Appendix Fig. S10. TRACE rankings of tissue-associated disease genes in each tissue model. Boxplots in each panel show the ranks of the same set of tissue-associated disease genes per tissue model; the red boxplot annotates the correct affected tissue. For example, the upper left panel shows the ranks of brain-associated disease genes in each tissue model. The median rank in the model of the correct affected tissue (e.g., brain) was higher than the median rank in other tissue models, except for blood-associated genes whose median was higher in liver (p-values appearing above each boxplot were calculated using paired Wilcoxon tests).

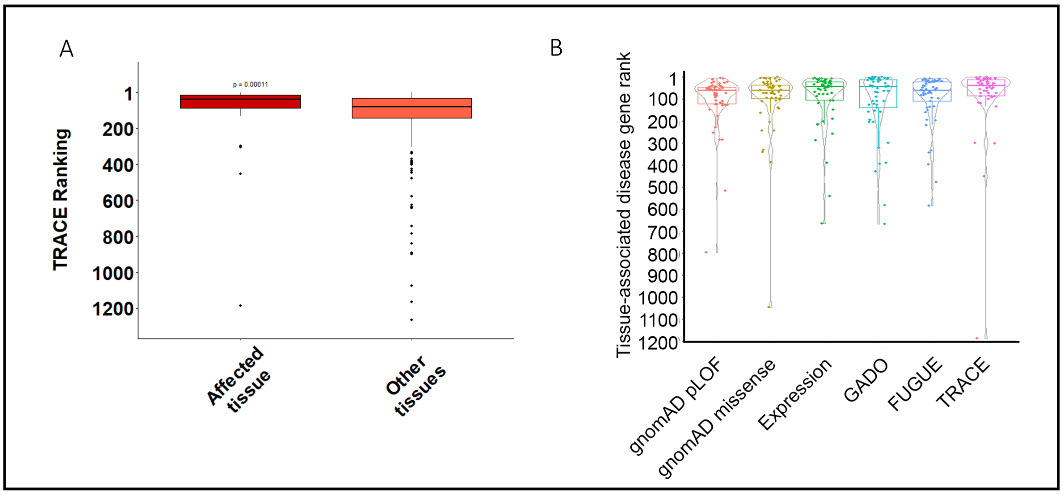

Appendix Fig. S11. TRACE ranking of verified disease genes.

A. TRACE rankings of verified disease genes of patients in the model of the patients' correct tissue (affected tissue, red boxplot) and in other models. The ranks in the model of the patients' correct tissue were significantly higher (p=0.00011, MW).

B. Comparison between the rank of the verified disease genes out of the patient's candidate genes between TRACE and other methods. Median rank of TRACE: 39; gnomAD (Karczewski *et al*, 2020) pLoF: 61 and missense: 60; expression-based prioritization: 43; GADO (Deelen *et al*, 2019): 45; FUGUE (Somepalli *et al*, 2021): 60. TRACE prioritization was better than prioritization by other methods (adjusted p=0.00155, 0.01, 0.01, 0.01, 0.01, respectively, Wilcoxon signed-rank test).

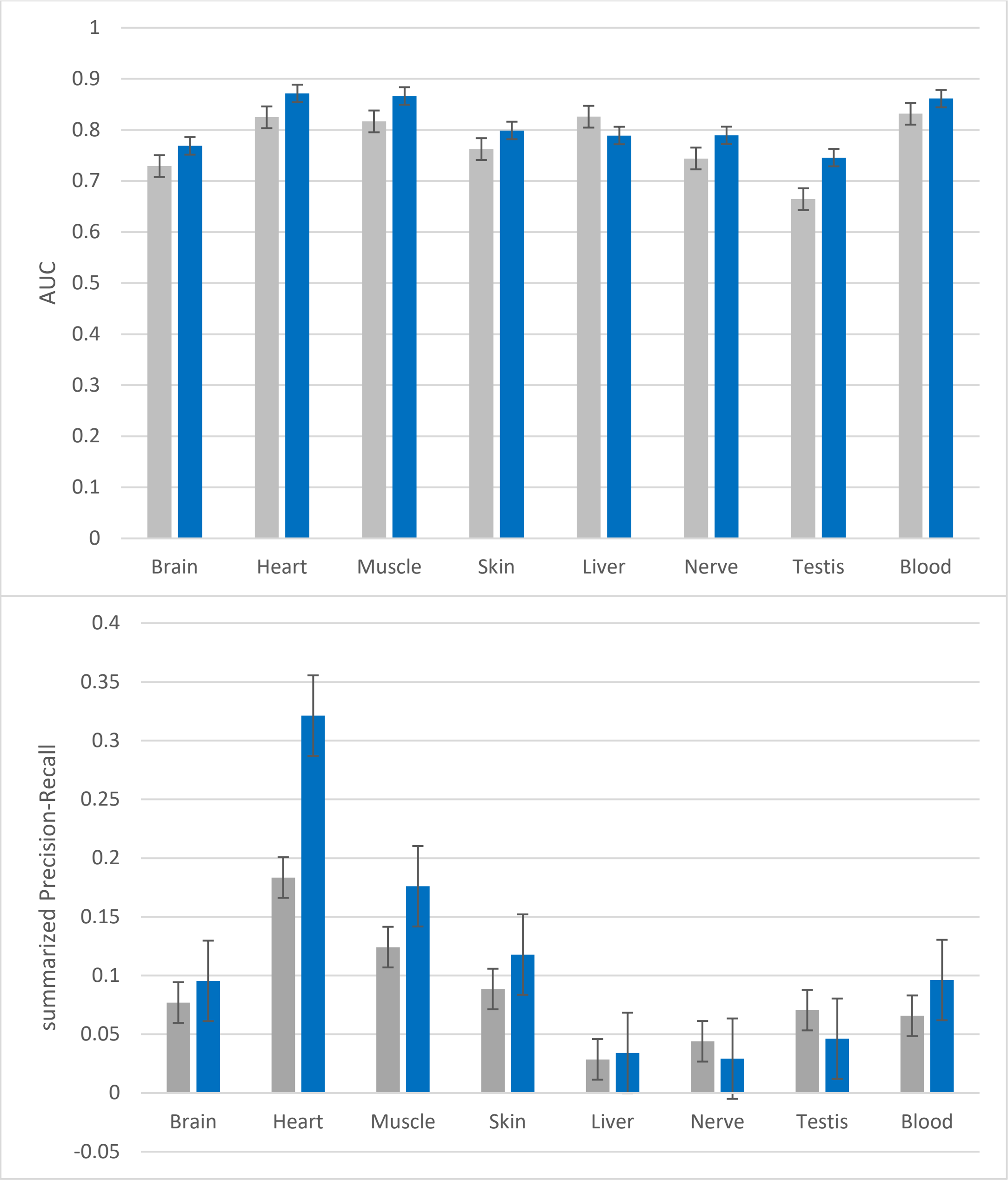

Appendix Fig. S12. The performance of TRACE models that used a partial features' dataset (grey) and models that used the full features dataset (blue). The partial dataset included 594 features that were derived from transcriptomics and PPIs were used; network embedding features were excluded.

A. The area under the receiver-operating characteristic curve (AUC) by each model. TRACE models based on the full dataset performed better than models that used the limited dataset in 7/8 cases (Wilcoxon signed-rank test p=0.039).

B. The summarized precision-recall. TRACE models based on the full dataset performed better than models that used the limited dataset in 6/8 cases (Wilcoxon signed-rank test p=0.11).

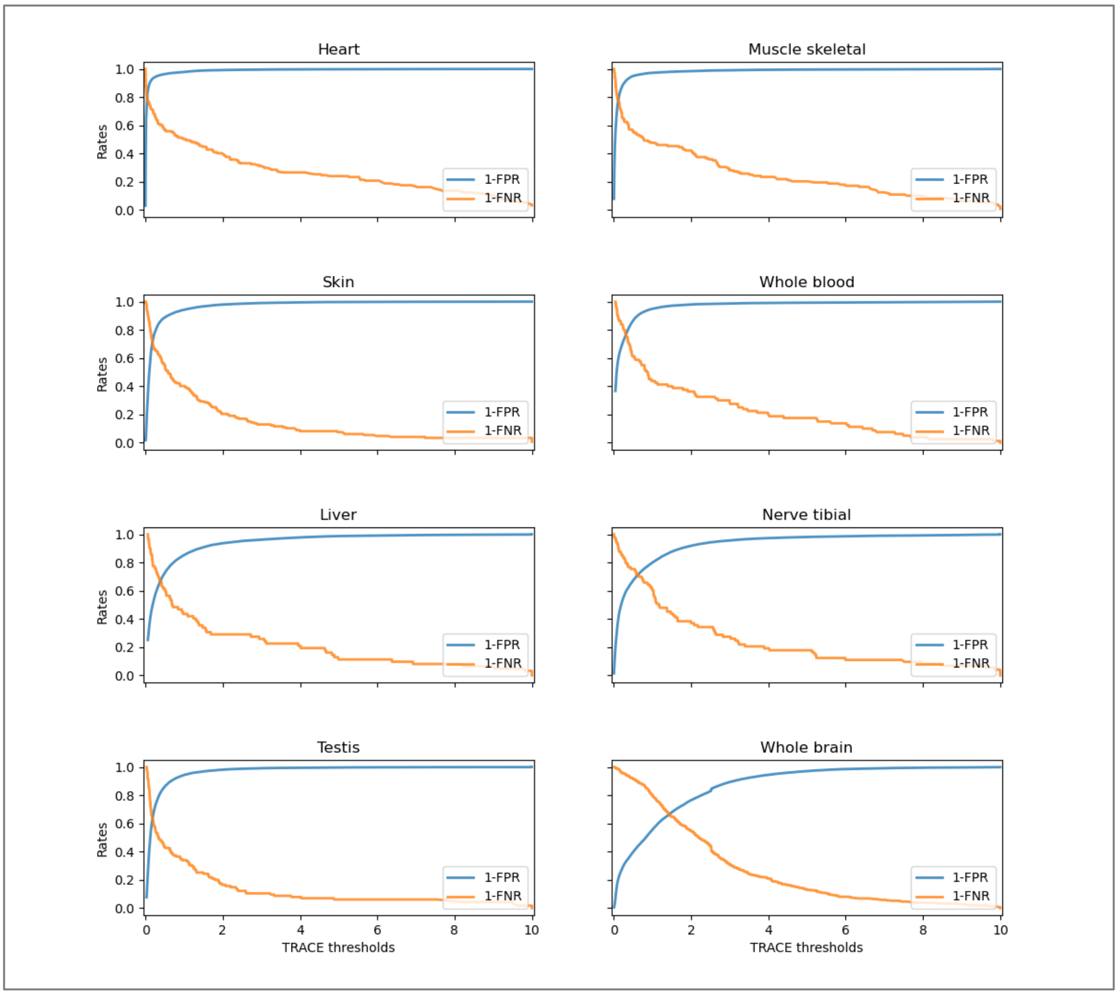

Appendix Fig. S13. Assessment of TRACE per model. The true positive rate versus true negative rate of each tissue model (FPR = false positive rate; FNR = false negative rate).

REFERENCES

Deelen P, van Dam S, Herkert JC, Karjalainen JM, Brugge H, Abbott KM, van Diemen CC, van der Zwaag PA, Gerkes EH, Zonneveld-Huijssoon E *et al* (2019) Improving the diagnostic yield of exome- sequencing by predicting gene-phenotype associations using large-scale gene expression analysis. *Nat Commun* 10: 2837

Karczewski KJ, Francioli LC, Tiao G, Cummings BB, Alföldi J, Wang Q, Collins RL, Laricchia KM, Ganna A, Birnbaum DP *et al* (2020) The mutational constraint spectrum quantified from variation in 141,456 humans. *Nature* 581: 434-443

Sharon M, Vinogradov E, Argov CM, Lazarescu O, Zoabi Y, Hekselman I, Yeger-Lotem E (2022) The differential activity of biological processes in tissues and cell subsets can illuminate disease-related processes and cell type identities. *Bioinformatics*

Somepalli G, Sahoo S, Singh A, Hannenhalli S (2021) Prioritizing and characterizing functionally relevant genes across human tissues. *PLoS Comput Biol* 17: e1009194

Table S1. The tissue-aware gene features used in the analyses.

Feature type	Dataset type	Feature description per gene *g*	Feature name (*t* denotes tissue name)	Available for # of tissues	Total # of features
**Tissue expression**	Transcriptomics	Median expression level of *g* in tissue or organ *t*[1]	*t_expression*	54 adult tissues	54
		Median expression in developmental time points of *g* in organ *t*[2]	*t_dev_expression_#* (*# ranges from 1 to 20 according to developmental time point in organ t)*	7 organs	134
**Tissue preferential expression**	Transcriptomics	Preferential expression (overexpression) of *g* in tissue *t* relative to other tissues	*t_pref_expression*	54 adult tissues	54
**Tissue eQTL (eQTL= expression quantitative trait loci)**	eQTL	An indicator of whether *g* is involved in at least one eQTL in tissue *t* (based on data from GTEx portal)	*t_eQTL*	48 adult tissues	48
**Tissue PPIs (PPIs=protein-protein interactions)** **A. Tissue interactors**	Transcriptomics + PPIs	Number of PPIs of *g*'s encoded protein in tissue *t*	*t_num_PPIs*	54 adult tissues	54
		Difference between number of PPIs of *g*'s encoded protein in tissue *t* and the median number of its PPIs across tissues	*t_num_PPIs_dif_med*	54 adult tissues	54
		Difference between number of PPIs of *g*'s encoded protein in tissue *t* and the mean number of its PPIs across tissues	*t_num_PPIs_dif_mean*	54 adult tissues	54
**Tissue PPIs** **B. Tissue preferential interactors**	Transcriptomics + PPIs	Number of PPIs of *g*'s encoded protein that involved preferentially expressed interactors in tissue *t*	*t_num_pref_PPIs*	54 adult tissues	54

		Difference between number of preferential PPIs of *g's* encoded protein in tissue *t* and the median number of its preferential PPIs across tissues	*t_num_pref_PPIs_dif_med*	54 adult tissues	54
		Difference between number of preferential PPIs of *g's* encoded protein in tissue *t* and the mean number of its preferential PPIs across tissues	*t_num_pref_PPIs_dif_mean*	54 adult tissues	54
**Tissue PPIs**<br>**C. Tissue-specific interactors**	Transcriptomics + PPIs	Number of PPIs of *g's* encoded protein in tissue *t* that were present in at most 20% of the tissues	*t_num_specific_PPIs*	54 adult tissues	54
		Difference between number of tissue specific PPIs of *g's* encoded protein in tissue *t* and the median number of its tissue specific PPIs across tissues	*t_num_specific_PPIs_dif_med*	54 adult tissues	54
		Difference between number of tissue specific PPIs of *g's* encoded protein in tissue *t* and the mean number of its tissue specific PPIs across tissues	*t_num_specific_PPIs_dif_mean*	54 adult tissues	54
**Tissue differential PPIs**[3]	Transcriptomics + PPIs	The minimum differential PPI score of *g's* encoded protein in tissue *t*	*t_diff_PPI_min*	44 adult tissues	44
		The maximum differential PPI score of *g's* encoded	*t_diff_PPI_max*	44 adult tissues	44

		protein in tissue *t*			
		The median differential PPI score of *g*'s encoded protein in tissue *t*	*t_diff_PPI_med*	44 adult tissues	44
		The mean differential PPI score of *g*'s encoded protein in tissue *t*	*t_diff_PPI_mean*	44 adult tissues	44
**Tissue network embedding**	Transcriptomics + PPIs	64 vectors representing the interactome neighborhood of *g*'s encoded protein in the PPI interactome of tissue *t*	*t_net_embed_#* *(# ranges between 1 and 64)*	54 adult tissues +generic interactome	3,520
**Tissue expression variability**	Transcriptomics	The variation in expression level of *g* across samples of tissue *t*[4]	*t_expression_var*	19 adult tissues	19
		The variation in the median expression level of *g* across developmental time points of organ *t*[2]	*t_dev_expression_var*	7 organs	7
**Tissue expression relationship between paralogous genes (ref barshir)**	Transcriptomics + homology	Ratio between the expression levels of *g* and its paralog with highest sequence identity in tissue *t*	*t_paralog_ratio_single*	53 adult tissues	53
		Ratio between the expression levels of *g* and the median expression level of all its paralogs in tissue *t*	*t_paralogs_ratio_all*	53 adult tissues	53
**Differential process activity**	Transcriptomics + gene ontology[5] (GO)	The number of GO biological process terms associated with *g* (denoted *g*'s GO terms)	*t_num_processes*	28 adult tissues	28
		Minimum differential activity score of *g*'s GO terms in tissue *t*	*t_diff_proc_act_min*	28 adult tissues	28
		Maximum differential activity score of	*t_diff_proc_act_max*	28 adult tissues	28

		g's GO terms in tissue *t*			
		Median differential activity score of *g*'s GO terms in tissue *t*	*t_diff_proc_act_med*	28 adult tissues	28
		Mean differential activity score of *g*'s GO terms in tissue *t*	*t_diff_proc_act_mean*	28 adult tissues	28

REFERENCES

1 Aguet, F. et al. The GTEx Consortium atlas of genetic regulatory effects across human tissues. 787903, doi:10.1101/787903 %J bioRxiv (2019).

2 Cardoso-Moreira, M. et al. Gene expression across mammalian organ development. Nature 571, 505-509, doi:10.1038/s41586-019-1338-5 (2019).

3 Basha, O., Shpringer, R., Argov, C. M. & Yeger-Lotem, E. The DifferentialNet database of differential protein-protein interactions in human tissues. Nucleic Acids Res 46, D522-D526, doi:10.1093/nar/gkx981 (2018).

4 Simonovsky, E., Schuster, R. & Yeger-Lotem, E. Large-scale analysis of human gene expression variability associates highly variable drug targets with lower drug effectiveness and safety. Bioinformatics 35, 3028-3037, doi:10.1093/bioinformatics/btz023 (2019).

5 The Gene Ontology, C. The Gene Ontology Resource: 20 years and still GOing strong. Nucleic Acids Res 47, D330-D338, doi:10.1093/nar/gky1055 (2019).

Membership of the GTEx Consortium

Laboratory and Data Analysis Coordinating Center (LDACC): François Aguet[1], Shankara Anand[1], Kristin G Ardlie[1], Stacey Gabriel[1], Gad Getz[1,30,31], Aaron Graubert[1], Kane Hadley[1], Robert E Handsaker[33,34,35], Katherine H Huang[1], Seva Kashin[33,34,35], Xiao Li[1], Daniel G MacArthur[34,36], Samuel R Meier[1], Jared L Nedzel[1], Duyen T Nguyen[1], Ayellet V Segrè[1,17], Ellen Todres[1]

Analysis Working Group (funded by GTEx project grants):
François Aguet[1], Shankara Anand[1], Kristin G Ardlie[1], Brunilda Balliu[41], Alvaro N Barbeira[2], Alexis Battle[18,11], Rodrigo Bonazzola[2], Andrew Brown[3,4], Christopher D Brown[24], Stephane E Castel[5,6], Donald F Conrad[42,43], Daniel J Cotter[29], Nancy Cox[16], Sayantan Das[26], Olivia M de Goede[29], Emmanouil T Dermitzakis[3,27,28], Jonah Einson[44,5], Barbara E Engelhardt[7,8], Eleazar Eskin[45], Tiffany Y Eulalio[46], Nicole M Ferraro[46], Elise D Flynn[5,6], Laure Fresard[12], Eric R Gamazon[13,14,15,16,] Diego Garrido-Martín[22], Nicole R Gay[29], Gad A Getz[1,30,31], Michael J Gloudemans[46], Aaron Graubert[1], Roderic Guigó[22,32], Kane Hadley[1], Andrew R Hamel[17,1], Robert E Handsaker[33,34,35], Yuan He[18], Paul J Hoffman[5], Farhad Hormozdiari[19,1], Lei Hou[47,1], Katherine H Huang[1], Hae Kyung Im[2], Brian Jo[7,8], Silva Kasela[5,6], Seva Kashin[33,34,35], Manolis Kellis[47,1], Sarah Kim-Hellmuth[5,6,9], Alan Kwong[26], Tuuli Lappalainen[5,6], Xiao Li[1], Xin Li[12], Yanyu Liang[2], Daniel G MacArthur[34,36], Serghei Mangul[45,48], Samuel R Meier[1], Pejman Mohammadi[5,6,20,21], Stephen B Montgomery[12,29], Manuel Muñoz-Aguirre[22,23], Daniel C Nachun[12], Jared L Nedzel[1], Duyen T Nguyen[1], Andrew B Nobel[49], Meritxell Oliva[2,10], YoSon Park[24,25], Yongjin Park[47,1], Princy Parsana[11], Abhiram S Rao[50], Ferran Reverter[51], John M Rouhana[17,1], Chiara Sabatti[52], Ashis Saha[11], Ayellet V Segrè[1,17], Andrew D Skol[2,53], Matthew Stephens[37], Barbara E Stranger[2,38], Benjamin J Strober[18], Nicole A Teran[12], Ellen Todres[1], Ana Viñuela[39,3,27,28], Gao Wang[37], Xiaoquan Wen[26], Fred Wright[54], Valentin Wucher[22], Yuxin Zou[40]

Analysis Working Group (not funded by GTEx project grants): Pedro G Ferreira[55,56,57,58], Gen Li[59], Marta Melé[60], Esti Yeger-Lotem[61,62]

Leidos Biomedical - Project Management: Mary E Barcus[63], Debra Bradbury[63], Tanya Krubit[63], Jeffrey A McLean[63], Liqun Qi[63], Karna Robinson[63], Nancy V Roche[63], Anna M Smith[63], Leslie Sobin[63], David E Tabor[63], Anita Undale[63]

Biospecimen collection source sites: Jason Bridge[64], Lori E Brigham[65], Barbara A Foster[66], Bryan M Gillard[66], Richard Hasz[67], Marcus Hunter[68], Christopher Johns[69], Mark Johnson[70], Ellen Karasik[66], Gene Kopen[71], William F Leinweber[71], Alisa McDonald[71], Michael T Moser[66], Kevin Myer[68], Kimberley D Ramsey[66], Brian Roe[68], Saboor Shad[71], Jeffrey A Thomas[71,70], Gary Walters[70], Michael Washington[70], Joseph Wheeler[69]

Biospecimen core resource: Scott D Jewell[72,] Daniel C Rohrer[72], Dana R Valley[72]

Brain bank repository: David A Davis[73], Deborah C Mash[73]

Pathology: Mary E Barcus[63], Philip A Branton[74], Leslie Sobin[63]

ELSI study: Laura K Barker[75], Heather M Gardiner[75], Maghboeba Mosavel[76], Laura A Siminoff[75]

Genome Browser Data Integration & Visualization: Paul Flicek[77], Maximilian Haeussler[78], Thomas Juettemann[77], W James Kent[78], Christopher M Lee[78], Conner C Powell[78], Kate R Rosenbloom[78], Magali Ruffier[77], Dan Sheppard[77], Kieron Taylor[77], Stephen J Trevanion[77], Daniel R Zerbino[77]

eGTEx groups: Nathan S Abell[29], Joshua Akey[79], Lin Chen[10], Kathryn Demanelis[10], Jennifer A Doherty[80], Andrew P Feinberg[81], Kasper D Hansen[82], Peter F Hickey[83], Lei Hou[47,1], Farzana Jasmine[10], Lihua Jiang[29], Rajinder Kaul[84,85], Manolis Kellis[47,1], Muhammad G Kibriya[10], Jin Billy Li[29], Qin Li[29], Shin Lin[86], Sandra E Linder[29], Stephen B Montgomery[12,29], Meritxell Oliva[2,10], Yongjin Park[47,1], Brandon L Pierce[10], Lindsay F Rizzardi[87], Andrew D Skol[2,53], Kevin S Smith[12], Michael Snyder[29], John Stamatoyannopoulos[84,88], Barbara E Stranger[2,38], Hua Tang[29], Meng Wang[29]

NIH program management: Philip A Branton[74], Latarsha J Carithers[74,89], Ping Guan[74], Susan E Koester[90], A. Roger Little[91], Helen M Moore[74], Concepcion R Nierras[92], Abhi K Rao[74], Jimmie B Vaught[74], Simona Volpi[93]

Affiliations

1. The Broad Institute of MIT and Harvard, Cambridge, MA, USA
2. Section of Genetic Medicine, Department of Medicine, The University of Chicago, Chicago, IL, USA
3. Department of Genetic Medicine and Development, University of Geneva Medical School, Geneva, Switzerland
4. Population Health and Genomics, University of Dundee, Dundee, Scotland, UK
5. New York Genome Center, New York, NY, USA
6. Department of Systems Biology, Columbia University, New York, NY, USA
7. Department of Computer Science, Princeton University, Princeton, NJ, USA
8. Center for Statistics and Machine Learning, Princeton University, Princeton, NJ, USA
9. Statistical Genetics, Max Planck Institute of Psychiatry, Munich, Germany
10. Department of Public Health Sciences, The University of Chicago, Chicago, IL, USA
11. Department of Computer Science, Johns Hopkins University, Baltimore, MD, USA
12. Department of Pathology, Stanford University, Stanford, CA, USA
13. Data Science Institute, Vanderbilt University, Nashville, TN, USA
14. Clare Hall, University of Cambridge, Cambridge, UK
15. MRC Epidemiology Unit, University of Cambridge, Cambridge, UK
16. Division of Genetic Medicine, Department of Medicine, Vanderbilt University Medical Center, Nashville, TN, USA
17. Ocular Genomics Institute, Massachusetts Eye and Ear, Harvard Medical School, Boston, MA, USA
18. Department of Biomedical Engineering, Johns Hopkins University, Baltimore, MD, USA
19. Department of Epidemiology, Harvard T.H. Chan School of Public Health, Boston, MA, USA
20. Scripps Research Translational Institute, La Jolla, CA, USA
21. Department of Integrative Structural and Computational Biology, The Scripps Research Institute, La Jolla, CA, USA
22. Centre for Genomic Regulation (CRG), The Barcelona Institute for Science and Technology, Barcelona, Catalonia, Spain
23. Department of Statistics and Operations Research, Universitat Politècnica de Catalunya (UPC), Barcelona, Catalonia, Spain
24. Department of Genetics, University of Pennsylvania, Perelman School of Medicine, Philadelphia, PA, USA
25. Department of Systems Pharmacology and Translational Therapeutics, University of Pennsylvania, Perelman School of Medicine, Philadelphia, PA, USA
26. Department of Biostatistics, University of Michigan, Ann Arbor, MI, USA
27. Institute for Genetics and Genomics in Geneva (iGE3), University of Geneva, Geneva, Switzerland
28. Swiss Institute of Bioinformatics, Geneva, Switzerland
29. Department of Genetics, Stanford University, Stanford, CA, USA
30. Cancer Center and Department of Pathology, Massachusetts General Hospital, Boston, MA, USA
31. Harvard Medical School, Boston, MA, USA
32. Universitat Pompeu Fabra (UPF), Barcelona, Catalonia, Spain
33. Department of Genetics, Harvard Medical School, Boston, MA, USA
34. Program in Medical and Population Genetics, The Broad Institute of Massachusetts Institute of Technology and Harvard University, Cambridge, MA, USA
35. Stanley Center for Psychiatric Research, Broad Institute, Cambridge, MA, USA

36. Analytic and Translational Genetics Unit, Massachusetts General Hospital, Boston, MA, USA
37. Department of Human Genetics, University of Chicago, Chicago, IL, USA
38. Center for Genetic Medicine, Department of Pharmacology, Northwestern University, Feinberg School of Medicine, Chicago, IL, USA
39. Department of Twin Research and Genetic Epidemiology, King's College London, London, UK
40. Department of Statistics, University of Chicago, Chicago, IL, USA
41. Department of Biomathematics, University of California, Los Angeles, Los Angeles, CA, USA
42. Department of Genetics, Washington University School of Medicine, St. Louis, Missouri, USA
43. Division of Genetics, Oregon National Primate Research Center, Oregon Health & Science University, Portland, OR, USA
44. Department of Biomedical Informatics, Columbia University, New York, NY, USA
45. Department of Computer Science, University of California, Los Angeles, Los Angeles, CA, USA
46. Program in Biomedical Informatics, Stanford University School of Medicine, Stanford, CA, USA
47. Computer Science and Artificial Intelligence Laboratory, Massachusetts Institute of Technology, Cambridge, MA, USA
48. Department of Clinical Pharmacy, School of Pharmacy, University of Southern California, Los Angeles, CA, USA
49. Department of Statistics and Operations Research and Department of Biostatistics, University of North Carolina, Chapel Hill, NC, USA
50. Department of Bioengineering, Stanford University, Stanford, CA, USA
51. Department of Genetics, Microbiology and Statistics, University of Barcelona, Barcelona. Spain.
52. Departments of Biomedical Data Science and Statistics, Stanford University, Stanford, CA, USA
53. Department of Pathology and Laboratory Medicine, Ann & Robert H. Lurie Children's Hospital of Chicago, Chicago, IL, USA
54. Bioinformatics Research Center and Departments of Statistics and Biological Sciences, North Carolina State University, Raleigh, NC, USA
55. Department of Computer Sciences, Faculty of Sciences, University of Porto, Porto, Portugal
56. Instituto de Investigação e Inovação em Saúde, University of Porto, Porto, Portugal
57. Institute of Molecular Pathology and Immunology, University of Porto, Porto, Portugal
58. Laboratory of Artificial Intelligence and Decision Support, Institute for Systems and Computer Engineering, Technology and Science, Porto, Portugal
59. Columbia University Mailman School of Public Health, New York, NY, USA
60. Life Sciences Department, Barcelona Supercomputing Center, Barcelona, Spain
61. Department of Clinical Biochemistry and Pharmacology, Ben-Gurion University of the Negev, Beer-Sheva, Israel
62. National Institute for Biotechnology in the Negev, Beer-Sheva, Israel
63. Leidos Biomedical, Rockville, MD, USA
64. UNYTS, Buffalo, NY, USA
65. Washington Regional Transplant Community, Annandale, VA, USA
66. Therapeutics, Roswell Park Comprehensive Cancer Center, Buffalo, NY, USA
67. Gift of Life Donor Program, Philadelphia, PA, USA
68. LifeGift, Houston, TX, USA
69. Center for Organ Recovery and Education, Pittsburgh, PA, USA
70. LifeNet Health, Virginia Beach, VA. USA

71. National Disease Research Interchange, Philadelphia, PA, USA
72. Van Andel Research Institute, Grand Rapids, MI, USA
73. Department of Neurology, University of Miami Miller School of Medicine, Miami, FL, USA
74. Biorepositories and Biospecimen Research Branch, Division of Cancer Treatment and Diagnosis, National Cancer Institute, Bethesda, MD, USA
75. Temple University, Philadelphia, PA, USA
76. Virginia Commonwealth University, Richmond, VA, USA
77. European Molecular Biology Laboratory, European Bioinformatics Institute, Hinxton, United Kingdom
78. Genomics Institute, University of California Santa Cruz, Santa Cruz, CA, USA
79. Carl Icahn Laboratory, Princeton University, Princeton, NJ, USA
80. Department of Population Health Sciences, The University of Utah, Salt Lake City, Utah, USA
81. Departments of Medicine, Biomedical Engineering, and Mental Health, Johns Hopkins University, Baltimore, MD, USA
82. Department of Biostatistics, Bloomberg School of Public Health, Johns Hopkins University, Baltimore, MD, USA
83. Department of Medical Biology, The Walter and Eliza Hall Institute of Medical Research, Parkville, Victoria, Australia
84. Altius Institute for Biomedical Sciences, Seattle, WA, USA
85. Division of Genetics, University of Washington, Seattle, WA, University of Washington, Seattle, WA, USA
86. Department of Cardiology, University of Washington, Seattle, WA, USA
87. HudsonAlpha Institute for Biotechnology, Huntsville, AL, USA
88. Genome Sciences, University of Washington, Seattle, WA, USA
89. National Institute of Dental and Craniofacial Research, Bethesda, MD, USA
90. Division of Neuroscience and Basic Behavioral Science, National Institute of Mental Health, National Institutes of Health, Bethesda, MD, USA
91. National Institute on Drug Abuse, Bethesda, MD, USA
92. Office of Strategic Coordination, Division of Program Coordination, Planning and Strategic Initiatives, Office of the Director, National Institutes of Health, Rockville, MD, USA
93. Division of Genomic Medicine, National Human Genome Research Institute, Bethesda, MD, USA
