# Supplementary material for: Predicting molecular mechanisms of hereditary diseases by using their tissue‐selective manifestation
Source: Mol Syst Biol. 2023 May 26;19(8):e11407. doi: 10.15252/msb.202211407 (PMC10407743; doi:10.15252/msb.202211407)
Supplement: Supplementary file 2 — Expanded View Figures PDF [file MSB-19-e11407-s004.pdf]

Expanded View Figures

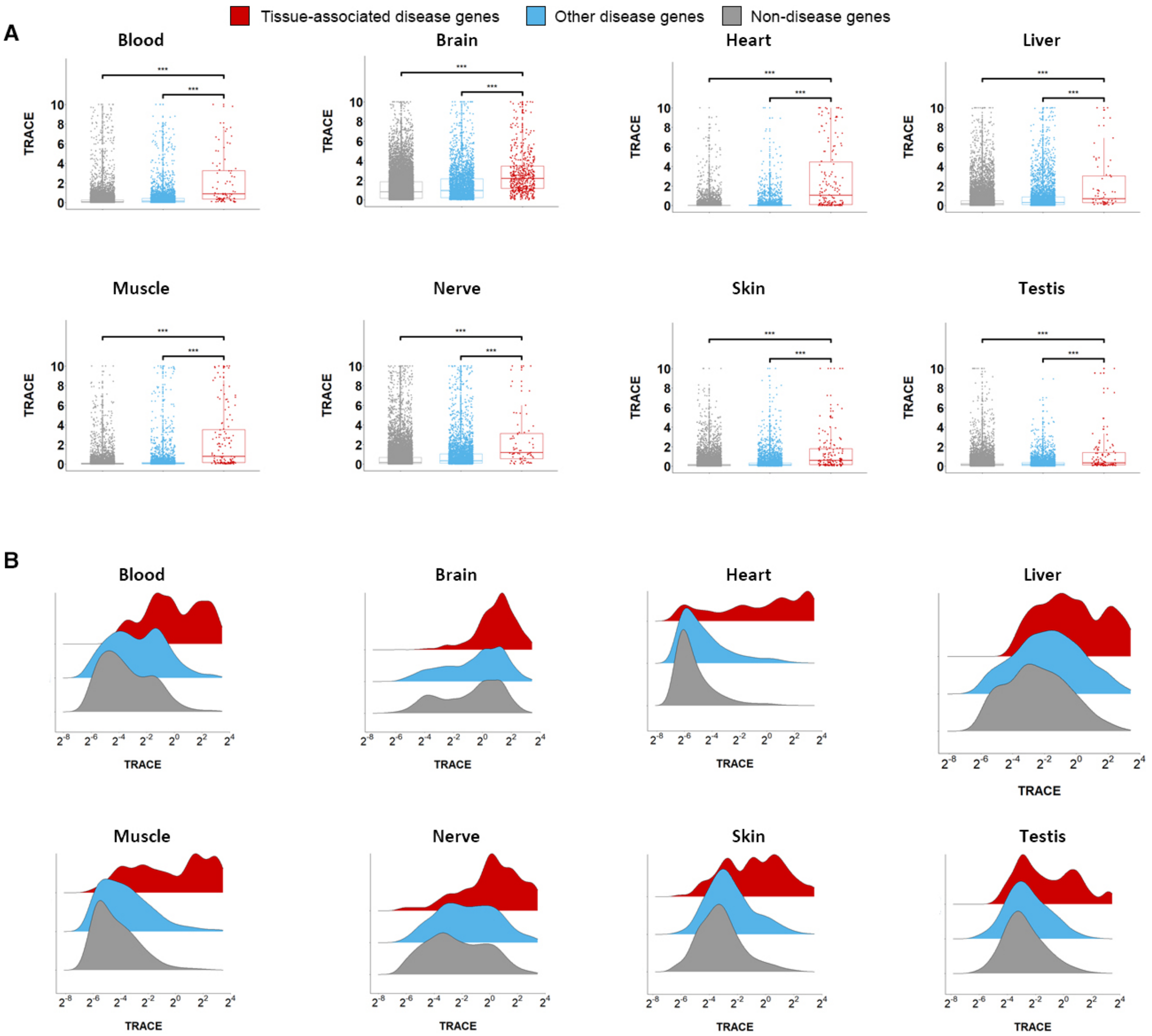

Figure EV1. TRACE prioritization of genes associated with diseases that manifest in distinct tissues.

A Gene TRACE scores in the different tissue models. Each dot represents a different gene. Genes were divided into genes that are causal for a disease that manifests in the modeled tissue (tissue-associated, red), genes that are causal for a disease that does not manifest in the modeled tissue (other disease genes, blue), and nondisease genes (gray). Tissue-associated disease genes had significantly higher TRACE scores compared with nondisease genes and to other disease genes. Mann–Whitney *U* test (MW) adjusted *P*-values for the comparison of tissue-associated genes to noncausal genes: Blood ($2.85*10^{-16}$), brain ($2.85*10^{-16}$), heart ($2.85*10^{-16}$), liver ($4.44*10^{-13}$), skeletal muscle ($2.85*10^{-16}$), nerve ($2.85*10^{-16}$), skin ($2.85*10^{-16}$), testis ($2.85*10^{-16}$). MW adjusted *P*-values for the comparison of tissue-associated genes to other disease genes: Blood ($2.22*10^{-16}$), brain ($2.22*10^{-16}$), heart ($2.22*10^{-16}$), liver ($1.10*10^{-6}$), skeletal muscle ($2.22*10^{-16}$), nerve ($3.70*10^{-11}$), skin ($2.22*10^{-16}$), and testis ($9.80*10^{-12}$). Boxplot central band indicates median; box limits indicate 25th to 75th percentiles; whiskers indicate 1.5 × interquartile range. The number of analyzed values was ~ 60–500 for tissue-associated genes, ~ 3,430–3,860 for other disease genes, and ~ 15,000 for nondisease genes. Panels relating to brain, heart, skeletal muscle, and skin are the same as those in Fig 4D.

B Density ridge plots of gene TRACE scores in the different tissue models. Tissue-associated genes (red) were over-represented among genes with high TRACE scores.

Panels relating to brain, heart, skeletal muscle, and skin are the same as those in Fig 4E.

© 2023 The Authors

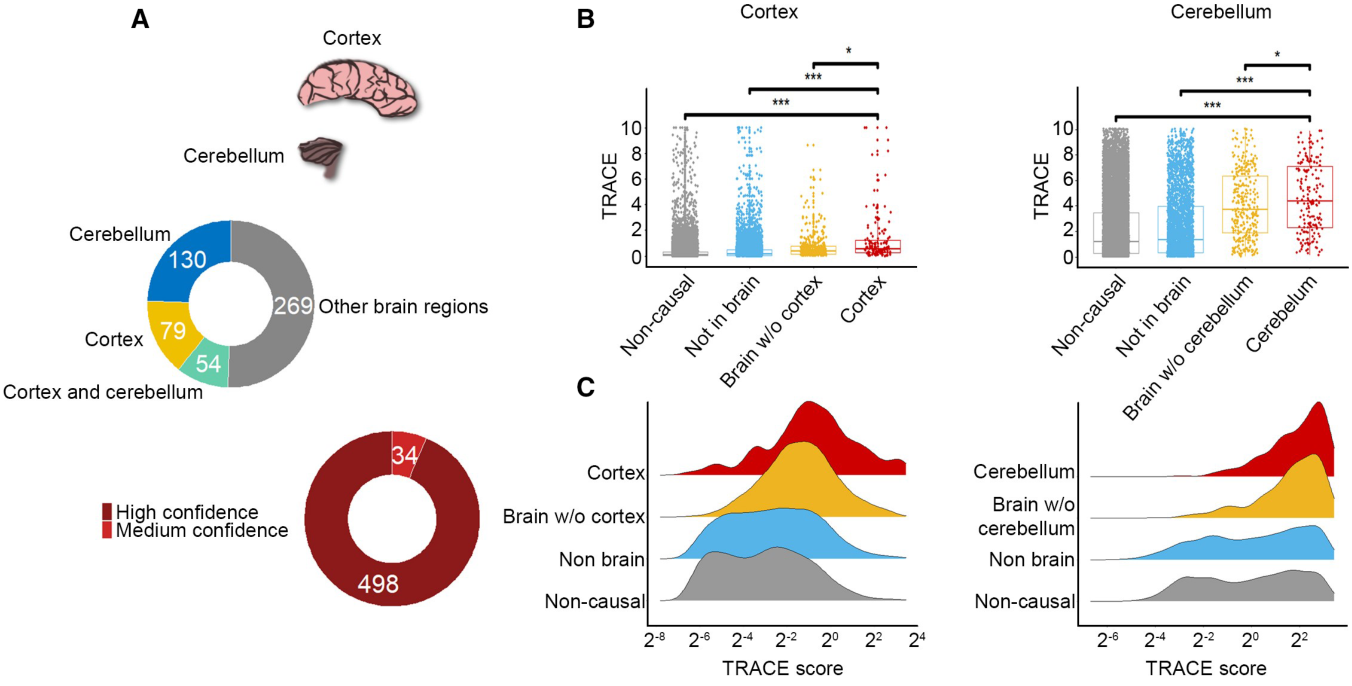

Figure EV2. TRACE prioritization of genes associated with diseases that manifest in brain cortex and cerebellum.

A The 532 genes known to be causal for brain diseases were associated with brain regions that manifest the disease at medium to high confidence. Most genes were associated with cerebellum and cortex.

B Gene TRACE scores in brain cortex and cerebellum models. Each dot represents a different gene. Genes were divided into nondisease genes (noncausal, gray), disease genes that are not causal for brain diseases (nonbrain, blue), disease genes that are causal for brain diseases that do not manifest in the modeled brain region (brain w/o modeled region, orange), and disease genes that are causal for brain diseases that manifest in the modeled brain region (modeled brain region, red). Genes associated with the modeled brain region had significantly higher TRACE scores compared with all other gene sets (Cortex: *** = 3.3E-16, * = 0.028; cerebellum: *** = 3.3E-16, * = 0.033; MW, adjusted *P*-values shown). Boxplot central band indicates median; box limits indicate 25th to 75th percentiles; whiskers indicate 1.5 × interquartile range. The number of analyzed values was ~130–180 for disease genes of the modeled brain region, ~320–370 other brain disease genes, ~3,420 for other disease genes, and ~15,000 for nondisease genes.

C Density ridge plots of gene TRACE scores in brain cortex and cerebellum models. Genes associated with the modeled region were over-represented among genes with high TRACE scores.

 © 2023 The Authors
