# Supplementary material for: Predicting molecular mechanisms of hereditary diseases by using their tissue‐selective manifestation
Source: Mol Syst Biol. 2023 May 26;19(8):e11407. doi: 10.15252/msb.202211407 (PMC10407743; doi:10.15252/msb.202211407)
Supplement: Supplementary file 6 — PDF+ [file MSB-19-e11407-s003.pdf]

Article

TRANSPARENT PROCESS

OPEN ACCESS

molecular systems biology

Predicting molecular mechanisms of hereditary diseases by using their tissue-selective manifestation

Eyal Simonovsky[1], Moran Sharon[1], Maya Ziv[1], Omry Mauer[1], Idan Hekselman[1], Juman Jubran[1], Ekaterina Vinogradov[1], Chanan M Argov[1], Omer Basha[1], Lior Kerber[1], Yuval Yogev[2], Ayellet V Segrè[3,4], Hae Kyung Im[5], GTEx Consortium[†], Ohad Birk[2,6], Lior Rokach[7] & Esti Yeger-Lotem[1,6,*]

Abstract

How do aberrations in widely expressed genes lead to tissue-selective hereditary diseases? Previous attempts to answer this question were limited to testing a few candidate mechanisms. To answer this question at a larger scale, we developed "Tissue Risk Assessment of Causality by Expression" (TRACE), a machine learning approach to predict genes that underlie tissue-selective diseases and selectivity-related features. TRACE utilized 4,744 biologically interpretable tissue-specific gene features that were inferred from heterogeneous omics datasets. Application of TRACE to 1,031 disease genes uncovered known and novel selectivity-related features, the most common of which was previously overlooked. Next, we created a catalog of tissue-associated risks for 18,927 protein-coding genes (https://netbio.bgu.ac.il/trace/). As proof-of-concept, we prioritized candidate disease genes identified in 48 rare-disease patients. TRACE ranked the verified disease gene among the patient's candidate genes significantly better than gene prioritization methods that rank by gene constraint or tissue expression. Thus, tissue selectivity combined with machine learning enhances genetic and clinical understanding of hereditary diseases.

Keywords data integration; genomic medicine; machine learning; omics; tissue selectivity

Subject Categories Computational Biology; Genetics, Gene Therapy & Genetic Disease; Methods & Resources

DOI 10.15252/msb.202211407 | Received 20 October 2022 | Revised 30 April 2023 | Accepted 10 May 2023 | Published online 26 May 2023

Mol Syst Biol. (2023) 19: e11407

Introduction

Genetic and clinical studies of Mendelian and rare heritable diseases strive to identify pathogenic variants (Eilbeck *et al*, 2017) and their functional effects (Hekselman & Yeger-Lotem, 2020) while facing multiple challenges. Exome and whole-genome sequencing of patients typically yield thousands of variants of unknown significance (McCarthy & MacArthur, 2017). To pinpoint the pathogenic variant among them, variants are scrutinized via multiple strategies, including sequence and conservation analyses (Adzhubei *et al*, 2013; Gelfman *et al*, 2017; Rentzsch *et al*, 2019), mutational constraints (Karczewski *et al*, 2020), clinical variants databases (Landrum *et al*, 2016), and similarity in function or phenotype between candidate genes and disease-associated genes (Aerts *et al*, 2006; Kumar *et al*, 2018; Deelen *et al*, 2019). Nevertheless, the rate of successful genetic diagnostic of patients stands on 25–60% (Chong *et al*, 2015; 100,000 Genomes Project Pilot Investigators *et al*, 2021), calling for additional strategies.

When a pathogenic variant is identified, the molecular mechanisms by which it leads to disease phenotypes typically remain elusive. Attesting to the complexity of this challenge, molecular mechanisms have remained hidden even for long-known and well-established pathogenic variants (Moaven *et al*, 2015; Hernandez *et al*, 2016; Goedert *et al*, 2017; Holmans *et al*, 2017; Huttlin *et al*, 2017). Here, we focused on the large set of Mendelian diseases that mainly affect few selected tissues, such as neurodegenerative disorders, skin diseases, or muscular dystrophies (Hekselman & Yeger-Lotem, 2020). The molecular mechanisms that underlie tissue-selective Mendelian diseases are especially intriguing, as

1 Department of Clinical Biochemistry and Pharmacology, Ben-Gurion University of the Negev, Beer Sheva, Israel
2 Morris Kahn Laboratory of Human Genetics and the Genetics Institute at Soroka Medical Center, Faculty of Health Sciences, Ben Gurion University of the Negev, Beer Sheva, Israel
3 Ocular Genomics Institute, Massachusetts Eye and Ear, Harvard Medical School, Boston, MA, USA
4 The Broad Institute of MIT and Harvard, Cambridge, MA, USA
5 Section of Genetic Medicine, Department of Medicine, The University of Chicago, Chicago, IL, USA
6 The National Institute for Biotechnology in the Negev, Ben-Gurion University of the Negev, Beer Sheva, Israel
7 Department of Software & Information Systems Engineering, Ben-Gurion University of the Negev, Beer Sheva, Israel
*Corresponding author. Tel: +972 86428675; E-mail: estiyl@bgu.ac.il
[†]Membership of the GTEx Consortium appears in the Appendix

© 2023 The Authors. Published under the terms of the CC BY 4.0 license.

often the genes that harbor pathogenic variants (denoted disease genes) are expressed ubiquitously across the human body (Hekselman & Yeger-Lotem, 2020). For example, familial mutations in the gene dystroglycan 1 (DAG1) cause a rare inherited neuromuscular disorder. Likewise, familial mutations in the gene BRCA1 increase the risk for breast and ovarian cancers. Contrary to their tissue-selective disease manifestations, DAG1 and BRCA1 are expressed in many tissues (GTEx Consortium, 2020). Notably, knowledge of disease-affected tissues can help pinpoint pathogenic variants and their mode of action. For example, genetic diagnosis of patients with rare muscle disorders and interpretation of genetic variants were aided by information on transcripts expression in normal skeletal muscle and other tissues, respectively (Cummings *et al*, 2017, 2020).

Some of the efforts to illuminate disease mechanisms utilized the immense molecular characterization of tens of physiological human tissues, including tissue transcriptomes (GTEx Consortium, 2020), proteomes (Uhlen *et al*, 2015), and regulatory and epigenetic signals (Davis *et al*, 2018). Tissue omics datasets, especially tissue transcriptomes, were used for rare variant interpretation (Cummings *et al*, 2017, 2020), and for prioritizing candidate disease genes by their similarity to known disease-related genes, whereby similarity was based on attributes such as protein interactors or associated Human Phenotype Ontology (HPO) terms, in tools such as Endeavor (Aerts *et al*, 2006), Exomiser (Smedley *et al*, 2015), pBRIT (Kumar *et al*, 2018), and GADO (Deelen *et al*, 2019).

In parallel, studies of tissue-selective Mendelian diseases revealed various tissue-based features of disease genes, such as their tendencies for preferential expression (Lage *et al*, 2008; Barshir *et al*, 2014), molecular interactions (Magger *et al*, 2012; Barshir *et al*, 2014; Greene *et al*, 2015; Malod-Dognin *et al*, 2019; Basha *et al*, 2020), and dosage imbalance with modifier genes (Barshir *et al*, 2018; Jubran *et al*, 2020) in normal samples of disease-affected tissues. Likewise, studies of tissue-selective complex traits revealed tendencies of trait-associated genes to disrupt tissue-specific regulatory relationships (Marbach *et al*, 2016) and gene modules (Kitsak *et al*, 2016), or to overlap with active eQTLs (Barbeira *et al*, 2018; Gamazon *et al*, 2018, 2019) in normal samples of trait-manifesting tissues. Yet, these studies and others were limited to testing few candidate features.

Machine learning (ML) methods have been used widely in various biological contexts (Wong *et al*, 2021). Compared with traditional models such as multivariable regression models, ML can handle a larger number of features and consider not-predetermined and complex interactions between features (including nonlinear relations), thereby providing improved predictive performance. Additionally, ML can handle large amounts of data and automatically select the most relevant features, thereby supporting versatility and scalability. Prominent ML methods include deep learning and decision trees, the latter often preferred when training data are relatively limited and the interpretability of the resulting model is important. In tissue-specific contexts, deep learning was used to infer gene modules and variants for complex diseases (Dwivedi *et al*, 2020; Wesolowska-Andersen *et al*, 2020) and to predict cancer dependency of tumors (Chiu *et al*, 2021). Nonnegative matrix trifactorization was used to identify interactome-rewired genes in tissue-specific cancers (Malod-Dognin *et al*, 2019). Recently, decision trees were used to prioritize tissue-relevant genes, though the resulting model had limited interpretability (Somepalli *et al*, 2021). The latter method used supervised classification, where the goal is to train a classifier that maps an input to a predefined set of classes and accurately generalizes to unlabeled cases.

We hypothesized that ML can be used to dramatically expand the mechanistic understanding of tissue-selective diseases by (i) prioritizing candidate disease genes in tissue contexts and (ii) assessing candidate tissue-selectivity features. Our approach, termed "Tissue Risk Assessment of Causality by Expression" (TRACE), was designed as a supervised and interpretable ML framework. TRACE was implemented as an early integration ML scheme that utilized 4,744 tissue-based gene features, which were derived and combined from heterogeneous omics sources. The large variety of gene features greatly exceeded that of previous methods (Somepalli *et al*, 2021), enhancing TRACE interpretability. We trained and tested TRACE on 18,927 protein-coding genes, including 1,031 disease genes that underlie tissue-selective Mendelian diseases, which manifest in eight main tissues. Application of TRACE to specific diseases or jointly to diseases that manifest in the same tissue revealed known tissue-selectivity features, as well as commonly occurring yet previously underexplored features, such as the preferential activity of specific cellular processes (Sharon *et al*, 2022). Next, we harnessed TRACE to create a catalog of tissue-specific risks for 18,927 human protein-coding genes (https://netbio.bgu.ac.il/trace/). As proof-of-concept, we applied TRACE to prioritize candidate disease genes of patients with rare tissue-selective Mendelian diseases. Even though no sequence-based features were used, in 34% of the cases TRACE successfully prioritized the verified pathogenic gene among the top 10% of the patient's candidate genes. Thus, tissue-aware ML schemes can boost genetic and clinical studies of tissue-selective Mendelian diseases.

Results

Constructing tissue-based features and dataset for ML

We constructed a large-scale dataset consisting of engineered (interpretable) and abstract (performance-boosting) tissue-based features per protein-coding gene (Fig 1A and Appendix Table S1). Tissue-based features were motivated by studies of tissue-selective traits and diseases and were derived from diverse data sources, predominantly transcriptomes of adult (GTEx Consortium, 2020) and developing (Cardoso-Moreira *et al*, 2019) physiological human tissues, as well as tissue eQTLs (GTEx Consortium, 2020), experimentally detected protein–protein interactions (PPIs; e.g., Oughtred *et al*, 2019; Luck *et al*, 2020), and Gene Ontology (GO) biological process terms (Gene Ontology Consortium, 2015). Certain features were inferred from a single data source. For example, the feature "lung expression" reflected the expression of each gene in lung and was inferred from transcriptomes of adult lung. Other features were inferred by integrating multiple data sources, mostly tissue transcriptomes with other types of data (Fig 1A). For example, the feature "lung PPIs" reflected the number of PPIs of each protein in lung and was inferred by integrating data of PPIs with lung transcriptomes (Barshir *et al*, 2014; Appendix Fig S1). To support interpretability, we added tissue-comparative features. For example, "lung preferential expression" reflected the expression of each gene in

 © 2023 The Authors

lung relative to its expression in other tissues (Sonawane *et al*, 2017). Likewise, the feature "lung differential PPIs" reflected the difference, per protein, in the number of its PPIs between lung and other tissues (Materials and Methods).

In addition to including features with known association to tissue-selective disease manifestation, such as preferential expression of genes (Lage *et al*, 2008), we introduced features that were not previously assessed at large-scale. One such feature, denoted "differential process activity," was based on the differential activity of biological processes in a given tissue relative to other tissues, which was shown to illuminate processes that are preferentially active or underexpressed in specific tissues (Sharon *et al*, 2022). The differential activity of a process was recently estimated per tissue from the differential expression in that tissue of the genes annotated to that process (Sharon *et al*, 2022). To include this measure as a gene feature, we associated each gene with its biological processes according to GO and extracted the differential activities of these processes per tissue from Sharon *et al* (2022) (Materials and Methods). Lastly, to reduce interactomics data loss we added abstract network embedding vectors that represented gene neighborhoods in tissue interactomes (Materials and Methods). The entire features dataset included 4,744 distinct features that were computed per protein-coding gene (Appendix Table S1). Missing values were imputed, and values were transformed and scaled (Materials and Methods, Dataset EV1).

Next, we labeled genes according to whether they underlie tissue-selective Mendelian diseases. We retrieved Mendelian disease genes from OMIM and combined them with manually curated associations between Mendelian diseases and affected tissues (Barshir *et al*, 2018; Basha *et al*, 2020). Specifically, a disease was considered as affecting a tissue if its clinical manifestation was mainly in that tissue (Materials and Methods). Next, per disease, we associated the disease genes with the disease-affected tissues. Lastly, we labeled genes per tissue, such that only disease genes that were associated with that tissue were labeled positive, and all other genes were labeled negative. This resulted in a labeled dataset that was more disease-focused and stringent than used previously (Somepalli *et al*, 2021). Altogether, our features dataset encompassed 18,927 protein-coding genes, including 1,105 tissue-associated disease genes that unitedly affected 22 tissues (Dataset EV1).

Eight of the affected tissues, including blood, brain, heart, liver, nerve, skeletal muscle, skin, and testis, were each associated with over 60 disease genes, summing up to a total of 1,031 disease genes (Fig 1B). The tissue-based features dataset and the tissue-association labels of genes provided the basis for our ML scheme, denoted "Tissue Risk Assessment of Causality by Expression" (TRACE, Fig 1C). Below we describe the application of TRACE to uncover tissue-selectivity features of genes and to prioritize candidate disease genes identified in patients (Fig 1D–F).

TRACE predictions illuminate tissue-selectivity mechanisms

Our first goal was to test whether ML can be used to infer tissue-selectivity mechanisms by applying it to well-studied disease genes. We used the widely successful XGBoost (Chen & Guestrin, 2016; XGB) gradient-boosting ML method, since decision forest methods perform well on tabular data with thousands of training instances (Fernandez-Delgado *et al*, 2014), imbalanced classification tasks (Khalilia *et al*, 2011) and high-dimensional data that include large numbers of dependent features (Schwarz *et al*, 2010). Given a disease gene and its disease-affected tissue, we created an XGB model by training the model on all other genes, which were labeled according to their association with that tissue (i.e., disease genes whose disease affected that tissue were labeled positive, otherwise they were labeled negative). We then applied the trained model to the query disease gene (Materials and Methods). To rigorously highlight the features that contributed to the decision of each model, we used the SHAP (SHapley Additive exPlanations) algorithm (Lundberg *et al*, 2020), which is a game-theoretic method for explaining the prediction of ML models.

We illustrate the resulting inference using the predictions of two broadly expressed disease genes: CACNA1C that underlies arrhythmia, affecting the heart, and DMD that underlies Duchenne muscular dystrophy, affecting skeletal muscle (Fig 2). We selected those genes because they were not expressed exceptionally high in their disease-affected tissue (Appendix Fig S2). Nevertheless, TRACE successfully classified them as associated with heart and skeletal muscle, respectively. The top contributing feature of each model was the differential process activity of the disease gene, which was highest in the respective disease-affected tissue (Fig 2). Next, we asked which process contributed to the high value of that feature in the disease-affected tissue (Appendix Fig S3). In case of the arrhythmia gene CACNA1C, that process was "membrane depolarization during atrial cardiac muscle cell action potential," in accordance with arrhythmia phenotypes (Fig 2A and Appendix Fig S3A). In case of the Duchenne muscular dystrophy gene DMD, that process was "muscle filament sliding" (Fig 2B and Appendix Fig S3B). This process was indeed found to be impaired in mdx mouse model for Duchenne (Canepari *et al*, 2009). These examples demonstrate that interpretable ML models can point to disease-related processes.

Revealing common tissue-selectivity features of disease genes

We extended our analysis to identify common tissue-selectivity features. For this, we trained XGB classification models that aimed to distinguish tissue-associated disease genes from other genes. We applied this model to each tissue; however, robust models were created only for eight tissues with over 60 disease genes (Fig 1B, Materials and Methods). We then assessed the performance of the eight models by using 10-fold cross-validation. The average area under the receiver operating characteristic curve (AUC) obtained by the various tissue models was 0.71–0.87 (Appendix Fig S4A and B), attesting to discriminative power of the models. Next, we used SHAP to assess the contribution of each feature to each tissue model (Appendix Fig S5). Features that were associated with the modeled tissue, for example, "brain cortex expression" in the brain model and "differential process activity in skin" in the skin model, were among the top six most important features per model, and among the top three in 6/8 models (Fig 3A and Appendix Fig S5). These features were complemented by features of other tissues. For example, the third most contributing feature in the skin model was "differential process activity in subcutaneous fat," a tissue located just beneath the skin (Appendix Fig S5).

Next, we used SHAP to identify common tissue-selectivity features in an unbiased manner. For this, we summarized the contribution of the different features per tissue model by aggregating their

© 2023 The Authors

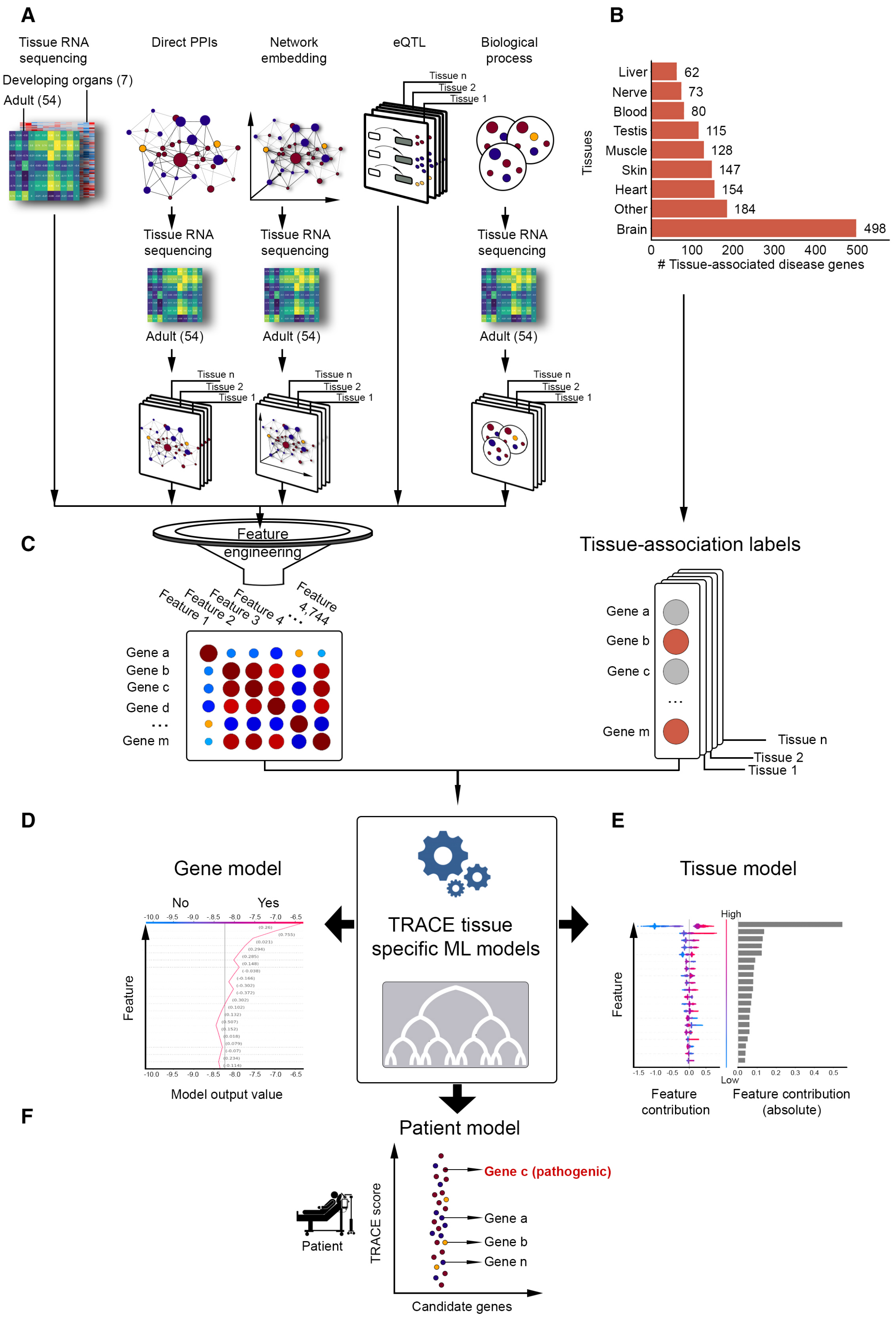

Figure 1.

 © 2023 The Authors

Figure 1. ML scheme for interpreting tissue-selective disease mechanisms.

A Construction of the tissue-based gene features dataset used in the analysis. Features were derived from transcriptomes of 54 human adult tissues and seven developing organs, PPIs, tissue eQTLs and gene annotations to biological processes. Datasets were integrated and interpretable features were engineered, resulting in 4,744 features for 18,927 protein-coding genes.

B The number of tissue-associated disease genes, which underlie a Mendelian disease that manifests mainly in the designated tissue or in other tissues (marked "Other").

C The input to TRACE included the dataset of tissue-based gene features and the tissue-association gene labels for the modeled tissue. Red labels mark tissue-associated disease genes. Various outputs of the models are described in panels (D–F).

D Buildup of the output value for the predicted tissue association of a query gene in the modeled tissue. Starting with a neutral value at the bottom, the output value accumulates according to the feature values of the query gene. Features are ordered from bottom to top by their increased contribution to the model, allowing interpretation. The location of the final output value on the *X* axis indicates whether the query gene is predicted to be a tissue-associated disease gene (yes, right) or not (no, left) in the modeled tissue (the gray vertical bar denotes the baseline value).

E A quantitative view of the contribution of features to a TRACE tissue model. Features are ordered from bottom to top by their increased absolute contribution to the model (gray bars), allowing interpretation. Per feature, each dot represents the feature value of a different gene (red and blue denote high and low values of the feature, respectively). Dots are spread from left to right by their contribution to disease manifestation in the modeled tissue.

F TRACE prioritization of candidate disease genes of a patient. TRACE scores candidate genes by the likelihood that they underlie a disease that manifests in the patient's affected tissue. Gene C represents the verified disease gene of that patient.

normalized SHAP importance (Materials and Methods). First, per tissue model we aggregated the contribution of features that were associated with the same tissue (Fig 3B). For example, the contribution of the feature "brain cortex expression" was added to the contribution of other brain-associated features. In 7/8 tissue models, the modeled tissue was among the top three most contributing tissues out of 54 tissues. This implies that tissue-selective disease manifestation frequently stems from signifying attributes of the disease-affected tissue.

Next, we aggregated the contribution of features belonging to the same type of mechanism, regardless of the associated tissue. For example, "brain cortex expression" and "liver expression" were both associated with "tissue expression" (Fig 3C). Tissue expression, indeed a major and well-established determinant of tissue selectivity, was the topmost mechanism in 1/8 models. Tissue network embedding, which captures interactome neighborhood, another recognized factor, was topmost in 3/8 models. Notably, differential process activity, which was not previously recognized as a determinant of tissue selectivity, was topmost in 4/8 tissue models, attesting to its wide relevance. This implies that in many diseases, tissue-selective manifestation is driven by a process whose integrity is essential for tissue physiology. Information on the identity of that process could enhance our understanding of the disease and consequently help open avenues for therapy (Fig 2). Differential PPIs and preferential expression were also among the top features across models, suggesting that tissue-selective disease manifestation stems from both absolute (e.g., expression) and relative (e.g., preferential expression) tissue-based features of disease genes.

Cataloging the tissue-specific risks of protein-coding genes

Our next step was to create a ML-based catalog of tissue-associated risks of protein-coding genes. For this, we tested the performance of five ML classifiers including logistic regression (LR), a multilayer perceptron (MLP) neural network, and three tree-based ensemble methods that included XGB, random forest (RF), and gradient boosted trees initiated by a logistic regression model (LR + GB). For each of the eight modeled tissues, each classifier computed the probability of each gene to underlie a disease that manifests in that tissue. The performance of each classifier was then assessed per modeled tissue via 10-fold cross-validation by using the average AUC and area under the precision–recall curve (auPRC; Materials and Methods, Appendix Fig S6A and B). Best performance across tissue models was achieved by different ML tools. For example, XGB performed better than other ML tools in two tissue models with respect to AUC and in two other tissue models with respect to auPRC (Appendix Fig S6A and B). Therefore, we next tested whether the combination of ML methods could lead to better results. For this, we employed a deep neural network meta-learner (Vilalta & Drissi, 2002; meta-MLP). Per gene, the meta-MLP received as input the gene values obtained by the five classifiers and produced a final score. The meta-MLP typically obtained top AUC (0.75–0.87, Fig 4A) and top auPRC (Appendix Fig S6B). Thus, our final TRACE scheme was composed of two layers: The first layer combined the five ML classifiers, which computed the probability of each gene to underlie a disease that manifested in the modeled tissue, scaled to values between 0 and 10 per classifier. The second layer consisted of a meta-MLP that accepted as input the output of the five classifiers and produced a final TRACE score, also scaled between 0 and 10 (Fig 4B, Materials and Methods). Across tissue models, TRACE achieved average AUC and auPRC of 0.82 and 0.12 (expected 0.04), respectively (Fig 4C, Appendix Fig S7). Better performing models corresponded to tissues with larger numbers of tissue-associated disease genes (Fig 1B), suggesting that increased annotation efforts could improve prediction. We compared TRACE to pBRIT, a computational gene prioritization tool that correlates functional and phenotypic gene annotations through intermediate data fusion (Kumar *et al*, 2018; Materials and Methods). Although pBRIT relied on more types of functional and phenotypic gene annotations, TRACE performance was favorable, especially with respect to AUC (Fig 4C and Appendix Fig S7).

To further assess the tissue selectivity of TRACE, we divided protein-coding genes into three separate groups per tissue model. The first group consisted of tissue-associated disease genes, namely disease genes whose disease manifests in the modeled tissue. The second group consisted of other disease genes, namely disease genes whose disease does not manifest in the modeled tissue. The last group consisted of nondisease genes. We then compared between the TRACE scores of the different gene groups (Figs 4D and E, and EV1A and B). Across models, tissue-associated disease genes

© 2023 The Authors

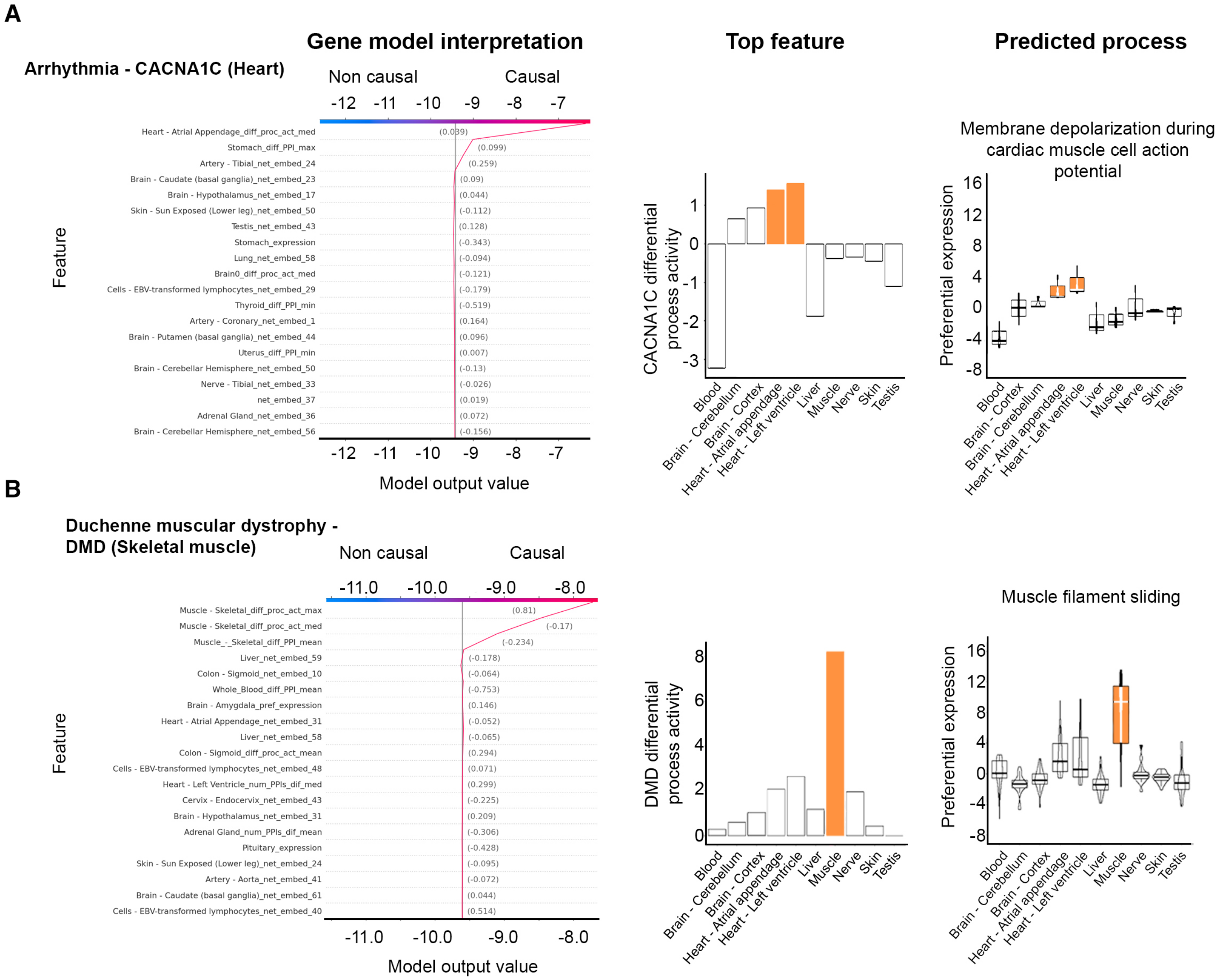

Figure 2. TRACE predictions illuminate disease-related mechanisms.

A The model for the arrhythmia gene CACNA1C predicted its association with heart (left). The model's topmost contributing feature, "differential process activity in heart," fits with the high differential process activity of CACNA1C in heart relative to other tissues (middle). This high activity stems from the preferential expression in heart of genes composing arrhythmia–related processes, including "membrane depolarization during atrial cardiac muscle cell action potential" (right).

B The model for the Duchenne muscular dystrophy gene DMD predicted its association with skeletal muscle (left). The model's topmost contributing feature, "differential process activity in skeletal muscle," agrees with the exceptionally high differential process activity of DMD in skeletal muscle relative to other tissues (middle). The high activity was due to the preferential expression in skeletal muscle of genes composing the process "muscle filament sliding" (right), previously implicated in Duchenne.

Data information: Boxplot central band indicates median; box limits indicate 25th to 75th percentiles; whiskers indicate 1.5 × interquartile range.

were enriched among genes with high TRACE scores. They had significantly higher TRACE scores when compared to nondisease genes ($P \leq 4.4E\text{-}13$, Mann–Whitney U test [MW]) and to other disease genes ($P \leq 1.1E\text{-}6$, MW), attesting to the tissue specificity of the models. To test whether high TRACE scores could predict tissue association in the absence of tissue-specific gene expression, we applied TRACE to the subset of 8,749 genes that were expressed in at least 80% of the tissues. We then compared between the TRACE scores of the three groups of genes described above. Apart from the liver tissue model, tissue-associated disease genes had significantly higher TRACE scores compared with nondisease genes and to other disease genes in all tissue models (Appendix Fig S8, $P < 0.029$, MW). Hence, TRACE predictions were not limited to tissue-specific genes. A similar tendency was observed for the subset of genes that were not overexpressed in any tissue (Appendix Fig S9). Lastly, we tested how often TRACE ranks disease genes highest in their associated tissue. For that, we compared TRACE ranks of disease genes between models of associated and other tissues. The median rank in the associated tissue model was higher than the median rank in other tissue models, except for blood-associated genes whose median rank was higher in liver (Appendix Fig S10).

 © 2023 The Authors

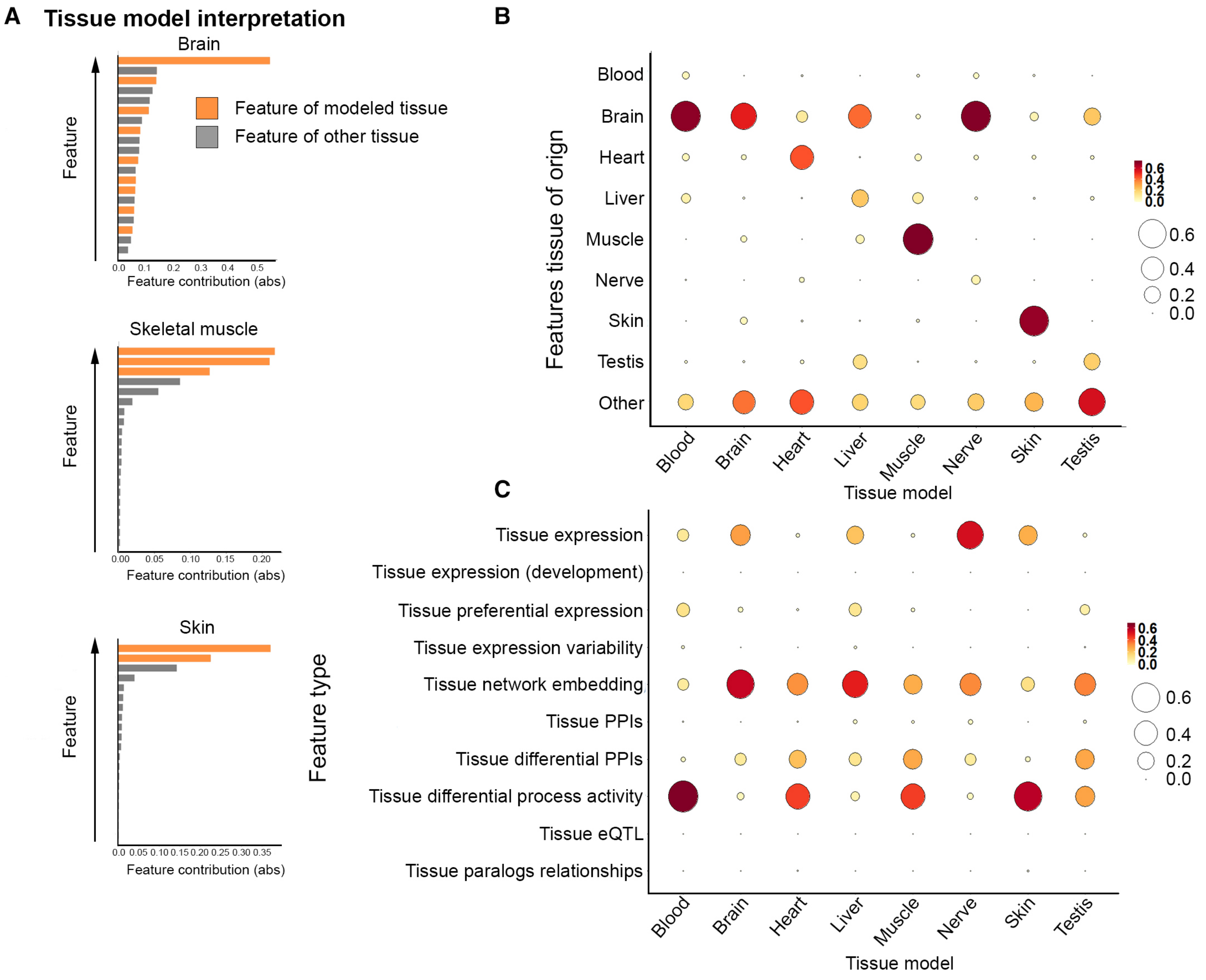

Figure 3. Tissue-specific models reveal common determinants of tissue selectivity.

A The contribution of the 20 topmost contributing features to the brain, skin, and skeletal muscle models. Features of the modeled tissue, marked by orange bars, were the major contributors to each model. Detailed tissue models appear in Appendix Fig S5.

B The contribution of tissue-based features to tissue models upon aggregating features by their associated tissue. Subregions of a tissue were associated with their main tissue. Aggregated features of the modeled tissue were among the three topmost contributing features in the brain, heart, liver, skeletal muscle, skin, and testis models.

C The contribution of features to tissue models upon aggregating features by their type. Tissue-differential process activity, tissue network embedding, and tissue expression were the topmost contributing feature types in at least one model.

So far, we applied TRACE to disease-affected tissues that were physiologically distinct from each other. Next, we turned to analyze the complex and often-uncertain selectivity of brain diseases to brain regions (Moustafa *et al*, 2016). We divided brain into six regions with available transcriptomic profiles, including cortex, cerebellum, basal ganglia, spinal cord, hypothalamus, and amygdala (Dataset EV2). We manually associated brain diseases to inflicted brain regions based on anatomical findings (see Materials and Methods). Altogether, we associated with low to high confidence 649 diseases and 832 disease genes to inflicted brain regions. 594 diseases and 532 disease genes were associated with medium or high confidence and were labeled as associated with those regions. Cortex and cerebellum were associated with over 60 disease genes and were henceforth modeled (Fig EV2A and Dataset EV2). As observed for physiologically distinct tissues, TRACE models were discriminative (AUC of 0.77 in both). Cortex-associated and cerebellum-associated disease genes had significantly high TRACE scores when compared to nondisease genes and to brain-unrelated disease genes ($P \leq 3.3\text{E-}16$, MW, Fig EV2B and C). In support of the models' specificity, cortex-associated and cerebellum-associated disease genes also had significantly high TRACE scores relative to other brain-associated disease genes (adjusted P-value of 0.028 and 0.033,

© 2023 The Authors

respectively, MW, Fig EV2B). Hence, TRACE models favored tissue-associated disease genes over disease genes that manifested in physiologically related tissues.

A catalog of the tissue-associated risks of each protein-coding gene in our dataset is available through the TRACE webserver (https://netbio.bgu.ac.il/trace/). Users can download the catalog, or upload a gene list or a VCF file, select a disease-inflicted tissue, and obtain the respective TRACE scores.

TRACE application to genetic diagnosis of rare-disease patients

Tissue contexts may play a role in the genetic diagnosis of patients with rare diseases that manifest in a tissue-selective manner. However, popular prioritization tools in clinical settings, such as CADD (Rentzsch *et al*, 2019) and gnomAD (Karczewski *et al*, 2020), are oblivious to tissue contexts (Eilbeck *et al*, 2017). To test the relevance of tissue contexts in such settings, we applied TRACE to a test

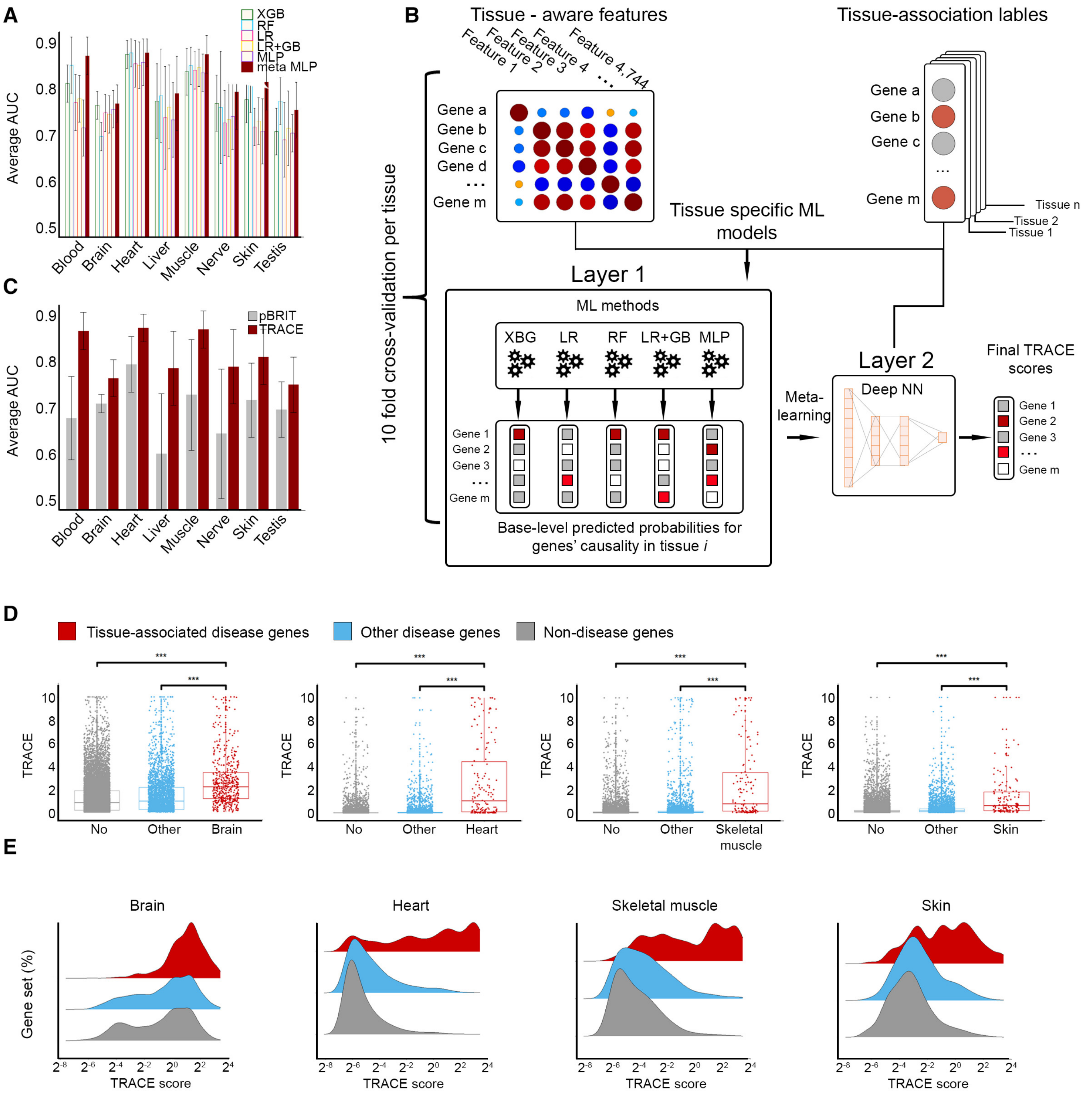

Figure 4.

 © 2023 The Authors

Figure 4. TRACE prioritization of tissue-associated disease genes.

A The average AUC obtained per ML method and tissue model. The highest AUC was typically obtained by the meta-learner.
B A schematic view of the final TRACE scheme. Input to TRACE included the dataset of tissue-based gene features and tissue-association gene labels for the modeled tissue (red labels mark disease genes for Mendelian diseases that manifest in the modeled tissue). The first layer includes independent application of five ML methods. Each method outputs gene scores reflecting the predicted probability that the gene underlies a disease that manifests in the modeled tissue. The second layer uses gene scores as input to a neural network (NN) meta-learner that produces the final TRACE score (red labels mark genes with higher predicted scores).
C The average AUC obtained with pBRIT (gray) and TRACE (crimson) per tissue model.
D Gene TRACE scores in brain, heart, skeletal muscle, and skin models. Each dot represents a different gene. Genes were divided into disease genes whose disease manifests in the modeled tissue (tissue-associated disease genes, red), disease genes whose disease does not manifest in the modeled tissue (other disease genes, blue), and nondisease genes (gray). Tissue-associated disease genes had significantly higher TRACE scores compared with nondisease genes and to other disease genes (*** = 3E-16; MW, adjusted *P*-values).
E Density ridge plots of gene TRACE scores in brain, heart, skeletal muscle, and skin models. Tissue-associated disease genes were over-represented among genes with high TRACE scores.

Data information: Bars and error bars indicate the mean ± SD obtained via 10-fold cross-validation. Boxplot central band indicates median; box limits indicate 25th to 75th percentiles; whiskers indicate 1.5 × interquartile range. Plots for additional tissue models appear in Fig EV1A and B.

set of 48 patients. Patients previously underwent whole-exome sequencing and variant characterization, and their pathogenic variant had been reported, as described in Materials and Methods (Fig 5A, Dataset EV3A).

To assess TRACE per patient, we compiled a list of the patient's genes that contained variants, henceforth referred to as candidate disease genes. The median number of candidate genes per patient was 186. Next, we created a TRACE model of the disease-affected tissue of the patient. For five patients that had two affected tissues, we created two TRACE models per patient. To ensure unbiased testing and avoid data leakage, we trained each TRACE model on all genes except for the patient's candidate disease genes; the genes used for training were labeled according to their association with the disease-affected tissue of the patient. We then applied the trained model to the patient's candidate disease genes to predict their association with the disease-affected tissue and prioritized them by their TRACE scores. Lastly, we ranked the gene containing the pathogenic variant, denoted the verified disease gene of the patient, relative to the patient's candidate disease genes (Fig 5B).

Though TRACE analysis of candidate disease genes per patient was literature blind and relied only of large-scale profiling data, in patients whose verified disease gene was well-established (though some contained novel pathogenic variants), TRACE ranked the verified disease gene at the top 1% of the patient's candidate genes. Examples include OPA1 that was verified in a patient with optic atrophy and neuropsychiatric disorders (MIM #125250); Sarcoglycan Gamma (SGCG) that was verified in a patient with muscular dystrophy (MIM #253700); and tumor protein p63 (TP63) that was verified in a patient with for ectodermal dysplasia syndrome (MIM #103285, #604292).

Analysis of patients whose verified disease gene was recently discovered further demonstrated the utility of TRACE. In one example, a familial case of lethal, severe microcephaly with various neurological features, the patient was found to have a unique mutation in SEC31A (Halperin *et al*, 2019). TRACE ranked SEC31A as the eighth most likely disease gene out of 107 patient's candidate disease genes. In a different case, a familial syndrome of muscle hypotonia, failure to thrive, and developmental delay, a variant in PAX7 was identified as pathogenic (Proskorovski-Ohayon *et al*, 2017). This disease affects multiple organs but manifests most severely in skeletal muscle. TRACE ranked PAX7 in skeletal muscle as the 10th most likely disease gene out of 150 candidate disease genes.

In general, TRACE ranked the verified disease gene above the median in 85% of the cases, and at the top quartile or top 10% of the patient's candidate disease genes in 55 and 34% of the cases, respectively (Fig 5C). These results suggest that genes that TRACE ranks below the median can typically be removed from further consideration. TRACE is intended for usage after automatic filtration of candidate genes by widely used tools. Sieving through the remaining candidate genes is time-consuming and often done manually by geneticists, hence cutting the list of candidate genes by half can alleviate geneticists' workload. Yet, additional advancements are required for efficient clinical usage.

These results also show that tissue contexts are important and relevant for genetic diagnosis. For example, ranks of verified disease genes were higher in the correct (affected) tissue model than in other tissue models (Appendix Fig S11A). Highest success rates were observed for brain and skin, which had relatively large numbers of tissue-associated disease genes and performed well in cross-validation: In 20/33 (61%) of the patients with brain-related diseases, the verified disease gene ranked at the top quartile, and in 3/4 patients with skin diseases, the verified disease gene ranked at the top 3%. To further assess TRACE performance, we tested whether the five top-ranking genes per patient could be functionally relevant specifically in the patient's affected tissue. Indeed, we found that 75% of them were associated with a GO process that was specific to the disease-affected tissue of the patient (Materials and Methods). For example, the five top-ranking candidate genes of patient #13786 that was diagnosed with muscular dystrophy, including the verified disease gene SGCG, were all associated with muscle-specific processes, such as "muscle contraction" and "muscle organ development" (Dataset EV3D).

We compared TRACE prioritization to other gene prioritization schemes (Fig 5D and Dataset EV3B). The first scheme was prioritization by the expression level of candidate disease genes in the disease-affected tissue. The second scheme was GADO, a recently published computational tool that uses human tissue transcriptomes to prioritize genes based on disease phenotypes (Deelen *et al*, 2019). The third scheme was pLoF or missense scores of gnomAD, a state-of-the-art gene-level metric that assessed gene constraints by the observed versus expected frequency of gene variants in the population (Karczewski *et al*, 2020). The fourth scheme was FUGUE, a recently published method for prioritizing tissue-relevant genes (Somepalli *et al*, 2021). Since the patients in our dataset were

© 2023 The Authors

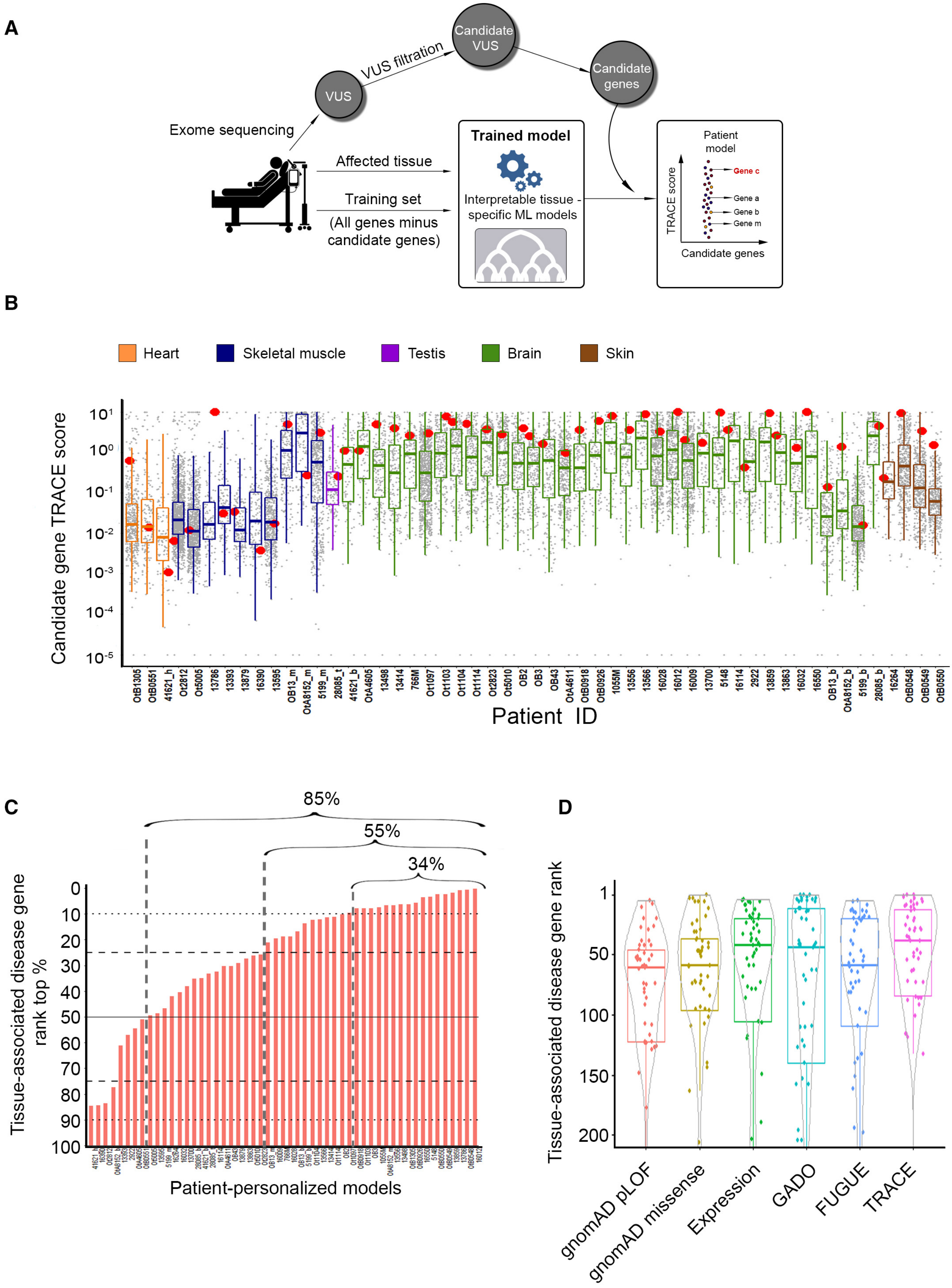

Figure 5.

 © 2023 The Authors

Figure 5. TRACE prioritization of patient-derived candidate disease genes associated with rare diseases.

A A schematic view of a patient-tailored TRACE modeling. Variants identified via exome sequencing of the patient were filtered by standard approaches, and remaining variants were mapped to their harboring genes, denoted candidate disease genes. Next, a TRACE model was trained on all genes except for the patient's candidate disease genes, using gene labels that match the affected tissue of the patient. Lastly, the trained model was applied to the patient's candidate disease genes, which were then prioritized according to their TRACE scores. The red-labeled gene is the verified disease gene, which contains the pathogenic variant of that patient.

B TRACE scores of candidate disease genes in 53 models for 48 patients with tissue-selective rare diseases (five patients were modeled in two distinct tissues). Per model, each dot represents a candidate disease gene; the red dot marks the verified disease gene of that patient. Boxplot color reflect the modeled tissue (orange = heart, blue = skeletal muscle, purple = testis, green = brain, brown = skin). In 46/53 models, the verified disease gene was ranked above the median.

C The rank of the verified disease genes out of the patient's candidate genes. Per model, ranks were determined based on gene TRACE scores, such that top scoring gene was ranked first. 85% of the verified disease genes ranked above the median; 55% of the verified disease genes ranked at the top quartile; and 34% of the verified disease genes ranked at the top 10%.

D Comparison between the rank of the verified disease genes out of the patient's candidate disease genes between gnomAD (Karczewski *et al*, 2020), expression-based prioritization, GADO (Deelen *et al*, 2019), FUGUE (Somepalli *et al*, 2021), and TRACE. Median rank of TRACE: 39; gnomAD pLoF: 61, missense: 60; expression-based prioritization: 43; GADO: 45; FUGUE: 60. TRACE prioritization was better than prioritization by the other methods (adjusted $P = 0.00155$, 0.01, 0.01, 0.01, 0.01, respectively, Wilcoxon signed-rank test; ranking outside top 200 appear in Appendix Fig S11B).

Data information: Boxplot central band indicates median; box limits indicate 25th to 75th percentiles; whiskers indicate $1.5 \times$ interquartile range.

diagnosed with the help of prioritization tools, their performance was evidently high (Fig 5D). Nevertheless, TRACE performed well, especially with respect to the median rank of the verified disease gene (Fig 5D and Dataset EV3C). gnomAD, which was used to assemble a list of candidate disease genes per patient, was less effective in the final prioritization. Its reduced performance relative to expression-based scheme showed that knowledge of the disease-affected tissue can contribute greatly to gene prioritization. The improved performance of TRACE over the expression-based scheme and FUGUE implied that other features, beyond expression, were meaningful for prioritization.

Discussion

We presented a ML approach for revealing and explaining tissue-specific functional consequences of genetic variation that leads to tissue-selective diseases. Our approach was motivated by the success of ML methods in biology (Gligorijevic & Przulj, 2015) and by known determinants of tissue selectivity (Lage *et al*, 2008; Magger *et al*, 2012; Barshir *et al*, 2014, 2018; Greene *et al*, 2015; Kitsak *et al*, 2016; Marbach *et al*, 2016; Barbeira *et al*, 2018; preprint: Barbeira *et al*, 2019; Hekselman & Yeger-Lotem, 2020). Thus, we harnessed various omics data to create tissue-based multiomics gene signatures with mechanistic interpretation (Appendix Table S1), which were much richer relative to previous efforts (Somepalli *et al*, 2021). By employing early data integration techniques, rather than late (Aerts *et al*, 2006) or intermediate (Kumar *et al*, 2018), and interpretable ML methods, TRACE was able to asses elaborated combinations of different types of gene features simultaneously (Gligorijevic & Przulj, 2015), estimate their relative importance (Figs 2 and 3), and use them for genetic diagnosis of patients (Fig 4).

As part of the work on TRACE, we created three large-scale resources for scientists and clinicians in the field of genomic medicine. First, we created a manually curated dataset of 532 brain diseases and their affected brain subregions (Dataset EV2). Second, we created a tabular ML-oriented dataset of 18,927 protein-coding genes multiomics signatures, which can be readily utilized by other ML frameworks. The signatures combined multiple types of data, such as transcriptomics and PPIs. Though some of the features in this dataset were correlated, we preferred to let models decide which features add information with respect to other dominant features of the model and applied feature selection where necessary to avoid overfitting (Materials and Methods). Lastly, we created a catalog of the tissue-associated risks for 18,927 protein-coding genes. The value of this catalog was demonstrated by analyzing data from patients (Fig 5).

Like other ML schemes, TRACE is inherently incomplete. The features dataset could be extended to include other gene features. In particular, many rare diseases have fetal origin; however, TRACE features were based mostly on adult tissue profiles since fetal tissue profiles were available only for seven organs (Cardoso-Moreira *et al*, 2019; Appendix Table S1). Likewise, the labeled dataset could be extended to include more tissue-associated disease genes. This could help improve prediction, as demonstrated by the enhanced performance of models based on larger numbers of such genes, and allow modeling of additional tissues. Alternatively, the goal of the classification and consequently the labeling could change. For example, by labeling genes that were not expressed in a tissue as negatives, the ML scheme could be used to prioritize genes with tissue-specific functions rather than disease–tissue associations (Somepalli *et al*, 2021). Additionally, tissue association of disease genes was represented by a binary variable. This dichotomy fits well with diseases that have clear tissue-specific manifestation, such as neurodegenerative disorders or skin diseases, yet might be less suitable for syndromes that affect multiple tissues to varying extents, or diseases with unclear or indirect tissue manifestations. The latter class of diseases could benefit greatly from multiclass classification; however, current data for training such models are scarce. Lastly, TRACE ranked verified disease genes high relative to other candidate genes of the same patient (median rank of 39/186 genes) and relative to other methods (Fig 5). However, additional advancements are needed to make this approach clinically actionable. This is especially important upon considering that patient exomes contain a much larger number of candidate genetic variants to which the current scheme is oblivious.

Despite limitations, TRACE demonstrated the power of ML to boost the understanding of tissue-selective diseases. The relative contribution of certain features to TRACE models could be used to generate hypotheses regarding disease mechanisms, such as their developmental origins. Another strength of ML is in unbiased estimation of novel features. This was exemplified by our assessment of biological process activity, which revealed its common,

previously understudied role (Fig 3C). Importantly, once identified, this feature highlighted disease-related processes (Fig 2). Lastly, TRACE prioritized candidate disease genes in patients with rare tissue-selective diseases (Fig 5). Since common variant prioritization schemes rely primarily on genomic attributes of variant sequences and are typically oblivious to tissue contexts (Eilbeck *et al*, 2017), TRACE provides a powerful complementary addition to current variant prioritization pipelines.

The application of TRACE provided insight into common determinants of tissue selectivity (Fig 3). Some of them were already shown to be common, such as preferential expression of disease genes in disease-inflicted tissues or their involvement in tissue-specific PPIs (Lage *et al*, 2008; Barshir *et al*, 2014; Fig 3B and C). Other known determinants of tissue selectivity were not highlighted. Tissue-selective underexpression of paralogs (Barshir *et al*, 2018; Jubran *et al*, 2020) was potentially masked by other features (Barshir *et al*, 2018). Involvement in tissue-specific eQTLs, which was previously observed in trait-associated genes (Barbeira *et al*, 2018; preprint: Barbeira *et al*, 2019), was less frequent in Mendelian diseases (Yao *et al*, 2020). And tissue-selective underexpression, which was previously observed in loss-of-function cancer genes (Lage *et al*, 2008), was probably masked by the preferential expression of disease genes, which was found to be much more common (Lage *et al*, 2008; Hekselman & Yeger-Lotem, 2020). Notably, the most common feature was tissue-preferential activity of biological processes, which involve multiple genes beside the disease gene. This suggests a mechanistic resemblance between monogenic diseases and multigenic complex traits. In the future, it will be interesting to extend TRACE toward additional phenotypes whose genetic mapping is routine yet mechanistic understanding is lagging, such as genome-wide association studies of complex traits, and toward multitissue disorders with potentially common disease-related features.

Several conclusions of broader impact can be drawn from our study. First, we showed that information on tissue-selective clinical manifestation of a disease is important for identifying its molecular mechanisms. Since this information is largely missing from databases devoted to diseases or clinical variants, such as OMIM and ClinVar, we strongly suggest to document this information in an easily retrievable format (e.g., Hekselman *et al*, 2022). Second, by utilizing high-level observational patient data (which of the patient's tissues were affected by disease) and combining it with general multiomics data, our study demonstrated that a personalized-to-nonpersonalized data channel is extremely practical and, in many cases, could be essential for personalized medicine. Whereas in some cases acquiring patient-specific omics data is feasible (Cummings *et al*, 2017), in many clinical settings throughout the world and for patients with, for example, brain diseases, this could be impractical and unachievable. This by itself greatly limits the ability to translate omics-based knowledge to clinical use. Therefore, frameworks that leverage non-patient-specific omics data that was acquired at large scale and combine it with data from individual patients, such as patients' exomes or symptoms, are greatly needed. Such frameworks could also bring real value and more immediate clinical impact to basic-science endeavors, which tend to translate to clinical use much later on. Third, ML schemes that utilize both interpretable and noninterpretable data are a favorable choice when developing ML frameworks for biological and clinical applications. Whereas "black box" methods, such as various deep learning architectures, could achieve some performance gain, they lack the ability to convey to human researchers and clinician the rational for ML recommendations and the biological signals that they are based on. As we showed, it is possible to embed "black-box"-style performance-boosting data in a way that does not undermine the interpretability of the ML models. Fourth, our study highlighted the importance of examining the behavior of disease genes in disease-inflicted and disease-unaffected tissues, leading toward a holistic approach to their investigation. The limited mechanistic understanding of rare diseases and the numbers of undiagnosed patients worldwide (Investigators *et al*, 2021) call for additional efforts and ready-to-use tools for boosting disease diagnosis and research.

Materials and Methods

Gene expression datasets and processing

Transcriptomic profiles of adult human tissues measured via RNA-sequencing were obtained from GTEx v8 and consisted of 17,382 profiles sampled from 54 tissues (GTEx Consortium, 2020). Expression values were available for 43,025 genes, including 18,927 protein-coding genes. Raw reads were normalized to obtain the same library size for every sample by using the trimmed mean of M-values (TMM) method by the edgeR package (Robinson *et al*, 2010). Genes with at most 10 raw counts in every sample were removed before normalization, as these genes were typically regarded as noise. To exclude protein-coding genes that were not reliably expressed in a specific tissue, only genes expressed at least 7 counts per million (cpm, linear scale) in at least half the samples of a tissue were considered as expressed in that tissue. Only protein-coding genes that were expressed in at least one tissue were further considered.

Preferential expression values per gene and tissue were computed from normalized counts as in Sonawane *et al* (2017) (see equation 1). Similarly to Sonawane *et al* (2017), genes with preferential expression ≥ 2 in a tissue were considered as preferentially expressed in that tissue. Transcriptomic profiles of developing human organs consisted of gene normalized counts that were measured at several time points during development and in adulthood in seven human organs, including cerebrum, cerebellum, heart, kidney, liver, ovary, and testis (Cardoso-Moreira *et al*, 2019). The expression of a gene in an organ and a time point was set to its median (*med*) normalized count in samples from the same time point and organ, resulting in a total of 133 profiles.

$$\forall t \in T, \forall g \in G : pref_g^{(t)} = \frac{\left[med\left(e_g^{(t)}\right) - med\left(med\left(e_g^{(\forall t \in T)}\right)\right)\right]}{IQR\left(med\left(e_g^{(\forall t \in T)}\right)\right)} \quad (1)$$

T denotes the set of tissues; G denotes the set of genes; *pref* denotes preferential expression; *e* denotes normalized count; *IQR* denotes interquartile range, that is, the difference between the 75th and 25th percentiles of the medians.

 © 2023 The Authors

Construction of the dataset of tissue-based gene features

The features dataset consisted of 4,744 features per protein-coding gene. Below we describe the different types of features (see Appendix Table S1), as well as imputation and scaling of the dataset.

Transcriptomic features

Per tissue, each gene was associated with several transcriptomic features. The first set of features reflected the expression level of the gene in the given tissue according to GTEx (if a gene was not expressed in the given tissue its value was set to zero). The second set of features corresponded to the gene's preferential expression in the given tissue relative to all tissues according to GTEx: Positive values reflected overexpression in the given tissue, and negative values reflected underexpression. Another set of transcriptomic features reflected the expression of genes per organ and per timepoint during development according to Cardoso-Moreira *et al* (2019).

eQTL features

Data of tissue eGenes, that is, genes involved in a tissue eQTL, were downloaded from the GTEX portal (March 20th, 2019, "GTEx_Analysis_v7_eQTL.tar.gz"). Each gene was associated with a feature per tissue that corresponded to its eGene q-value in that tissue.

PPI features

Data of experimentally detected PPIs were downloaded from publicly available databases using the default option of the MyProteinNet web tool (Basha *et al*, 2015). Each gene was associated with the set of interactors of its corresponding protein. Next, we integrated PPIs with GTEx expression data. Specifically, per tissue, we assigned each gene with three tissue-based primary PPI-related features, as follows: (i) Tissue interactors: Every gene that was considered to be expressed in the given tissue was assigned with the number of its interactors that were also considered to be expressed in that tissue; otherwise its value was set to 0. (ii) Preferential tissue interactors: Every gene that was considered to be expressed in the given tissue was assigned with the number of its interactors that were considered to be preferentially expressed in that tissue; otherwise its value was set to 0. (iii) Tissue-specific interactors: For every gene with nonzero tissue interactors in that tissue, the gene was assigned with the number of tissue interactors with which it interacted in at most 20% of the tissues (i.e., both the gene and its interactor were expressed in the same tissue in at most 20% of the tissues). For each primary feature (i)–(iii), we calculated two additional features per gene and tissue, to reflect the difference between the primary gene value in that tissue and its expected value. The expected value was calculated in two different ways, once according to the gene's mean primary value across tissues, and once according to the gene's median primary value across tissues.

Tissue-differential PPI features

Data of differential PPIs per gene and tissue were downloaded from The DifferentialNet database (Basha *et al*, 2018). This database assigns each PPI with a score per tissue that reflects whether the two interacting proteins are overexpressed or underexpressed in that tissue relative to all other tissues. Using these data, we associated each gene with the set of its differential interaction scores per tissue, and created four gene features per tissue, as follows: (i) the minimum differential interaction score of the gene in the given tissue. (ii) The maximum differential interaction score of the gene in the given tissue. (iii) The median differential interaction score of the gene in the given tissue. (iv) The mean differential interaction score of the gene in the given tissue. We included this set of potentially correlated features as we preferred to let the model decide which feature adds more information with respect to other dominant features in the model.

Network embedding features

Network embedding features were designed to represent the interactome neighborhood of a gene in a given PPI network (interactome). We started by creating a general interactome that contained all experimentally detected PPIs (see PPI features section above). Next, we integrated the general interactome with GTEx expression data to create tissue interactomes. Specifically, the interactome of a given tissue was set to include all PPIs between protein products of genes that were considered as expressed in that tissue. Lastly, we applied network embedding to the general human interactome and to each of the tissue interactomes. For each interactome, network embedding was computed by using the node2vec algorithm (Grover & Leskovec, 2016). Each gene was sampled by 20 walks of length 10, and every interactome was represented by embedding vectors of 64 dimensions.

Expression variability features

Data of expression variability scores per gene and tissue were obtained from Simonovsky *et al* (2019). Scores reflected the variability in expression levels of a gene across samples of a given tissue and were available for 19 tissues. Expression variability scores during development per gene and organ were computed based on Cardoso-Moreira *et al* (2019). Per organ and time point, each gene was assigned with its median normalized counts over the respective samples. Next, the expression variability of a gene in that organ was set to the coefficient of variation computed over the gene's median normalized counts in all developmental time points of the given organ.

Paralogous genes' features

Paralogous genes were defined as gene pairs whose reciprocal sequence identity was $\geq 40\%$ according to Ensembl-BioMart. We assessed the quantitative relationships between paralogous genes as described in Barshir *et al* (2018). Specifically, we calculated per sample the expression ratio between a gene and its best-matching paralog, where the best-matching paralog was defined as the paralog with the highest sequence identity that was expressed in any tissue according to GTEx. Next, we created a feature per tissue where each gene was assigned with the median expression ratio computed across samples of that tissue. To account for genes with multiple paralogs, we calculated per sample the ratio between the expression of a gene and the summed expression of all its paralogs with over 40% reciprocal sequence identity. Next, we created another feature per tissue where each gene was assigned with the median ratio computed across samples of that tissue.

Differential process activity features

We associated genes with biological processes by using Gene Ontology (The Gene Ontology Consortium, 2019; GO) terms and gene

annotations, which were downloaded from Ensembl-BioMart. We favored specific rather than general biological processes and thus considered only GO terms to which 3–100 human genes were annotated. We associated each term with a differential activity score per tissue, which reflected the expression of its corresponding process in that tissue relative to other tissues (Sharon *et al*, 2022). Per tissue, the differential activity score of a term was set to the average log2 fold-change values of its genes, where log2 fold-change value of each gene was computed based on its expression in that tissue relative to other tissues (Basha *et al*, 2017; Sharon *et al*, 2022). Using these scores, we associated each gene with the set of terms to which it was annotated, and created four gene features per tissue, as follows: (i) The minimum differential activity score of the gene's terms in the given tissue. (ii) The maximum differential activity score of the gene's terms in the given tissue. (iii) The median differential activity score of the gene's terms in the given tissue. (iv) The mean differential activity score of the gene's terms in the given tissue. We included this set of potentially correlated features as we preferred to let the model decide which feature adds more information with respect to other dominant features in the model.

Data imputation

Imputation of missing values was achieved via a MICE-inspired iterative imputation function (van Buuren & Groothuis-Oudshoorn, 2011). Each feature with missing values was considered as the target of a regression model, while using the rest of the features dataset as training data. First, per feature, all missing values were imputed by the median value of the respective feature. Then, per feature, the imputed values were reassessed by a Bayesian ridge regression model that used the 100 nearest features as training. Nearest features were evaluated based on the absolute correlation coefficient between each feature and the target feature. The reassessment of each feature was iterated 10 times, such that each iteration was initialized with values assessed in the preceding iteration.

Data transformation and scaling

Unlike tree-based models, logistic regression and neural network models could be confounded by nonsymmetrical distributions of data per feature. The main features that showed nonsymmetrical distributions were gene expression and preferential expression. These features were transformed to make them more symmetric by applying a Yeo-Johnson power transformation (Yeo & Johnson, 2000). Transformation was applied after these data were used for the construction of other features and before applying the different machine learning (ML) models. Following transformation, each feature in the complete dataset was scaled per tissue to values between −1 and 1 while preserving the shape of its data distribution.

The dataset of diseases, disease genes, and affected tissues

The set of Mendelian diseases was obtained from OMIM (McKusick-Nathans Institute of Genetic Medicine, n.d.) and included only Mendelian diseases with a known molecular basis (OMIM Phenotype mapping key 3). The genes associated with each disease, denoted disease genes (also marked with Phenotype mapping key 3), were also retrieved from OMIM. Overall, our dataset included 3,924 disease genes.

The association between Mendelian diseases and their affected tissues (i.e., the tissue that clinically manifests the disease) was obtained from manually curated datasets (Barshir *et al*, 2018; Basha *et al*, 2020). There, tissues were considered as affected by a disease if they presented disease-related clinical manifestations in most patients according to OMIM, HPO or the literature, and these manifestations were not secondary to disease sequela (e.g., we would exclude muscle wasting or contractures due to lack of ambulation, which results from severe neurological disease; for a detailed description, see (Hekselman *et al*, 2022)). Each disease gene whose disease affected a certain tissue was associated with that tissue, resulting 1,105 tissue-associated disease genes.

The labeling of genes per tissue

To support the application of ML methods, we labeled genes per tissue t according to their association with t. Disease genes that were associated with t were labeled as positive for t; all other genes were labeled as negative for t (Dataset EV1). In the analyses per tissue model t, the set of disease genes that were associated with t was denoted "tissue-associated disease genes." The set of remaining Mendelian disease genes was denoted "other disease genes." The set of "non-disease genes" included all genes except for Mendelian disease genes.

The annotation of diseases to brain regions

We further curated brain disorders to brain regions. For this, we associated brain subregions that were sampled by GTEx with six distinct regions: Cortex (including anterior cingulate cortex (BA24), hippocampus, cortex, frontal cortex); cerebellum (including cerebellum, cerebellar hemisphere); basal ganglia (including caudate, nucleus accumbens, and putamen); spinal cord; hypothalamus; and amygdala. Next, we manually associated brain disorders with their affected brain region(s). Associations were based on anatomical findings per disease that were detailed in disease pages of OMIM (Amberger *et al*, 2019). We assigned each association with a confidence level between 3 (high) and 1 (low), as follows: Associations based on clinical synopsis of the disease were assigned a confidence level of 3, unless they were described as pertinent only to some of the patients, in which case they are assigned a confidence level of 2. Associations based on disease description were assigned a confidence level of 2. Associations based on clinical features, which in some cases described finding relevant to a small subset of the patients, were assigned a confidence level of 1. Brain diseases that were not associated with any of the above regions (i.e., were associated with a different region, or were associated with brain but not with a specific region) were defined as "Other." The union of all diseases that were associated with any region was designated as "whole brain." The resulting dataset appears in Dataset EV2. To support the application of ML methods, we labeled genes per brain region b. Disease genes whose disease manifests in b at a confidence level of 2 and above were labeled as positive for b; all other genes were labeled as negative for b (Dataset EV2).

Using machine learning (ML) models to illuminate tissue-selectivity features

Below we describe the ML method used for interpretability analysis, its application to specific genes and to all tissue-associated disease

© 2023 The Authors

genes, and the SHAP (SHapley Additive exPlanations) analysis of feature importance that was used to interpret the resulting models.

ML method for interpretability analysis

To create interpretable models, we used the gradient boosted tree (GBT) algorithm. GBT trains a sequence of logistic regression trees, where each successive tree aims to predict the pseudo-residuals of the preceding trees assuming that the loss function is logistic loss. This method allows combining a huge number of shallow logistic regression trees by setting the learning rate to a small value. We employed the popular variant of GBT named "Extreme Gradient Boosting" (XGBoost, XGB), which is considered the state-of-the-art algorithm for training GBT (Chen & Guestrin, 2016). We applied XGB to interpret the tissue selectivity of query genes, and the tissue selectivity of tissue-associated disease genes for eight tissues, as described below. The input to XGB included the features dataset and the gene labels corresponding to the specific classification task. To reduce run time owing to the size of the features dataset and to reduce noise derived from noncontributing features, each application of XGB was preceded by feature selection that limited the number of relevant features per application to 50. Features were selected by applying support vector machines (SVM) with L1 regularization. We used SVM due to its capability to address high-dimensional data. SVM was trained on, and fitted to, each relevant training set. By this, we selected different tailored sets of relevant features per application.

For more details on XGB implementation see "ML implementation details" below.

XGB application to specific genes and to all genes

To interpret the tissue selectivity of query genes (e.g., Fig 2), we trained an XGB model on all genes in our dataset except the query gene. For a query gene whose disease manifests in tissue *t*, disease genes that were associated with *t* were labeled as positive; other genes were labeled as negative. The query gene was then tested by the model.

To interpret the tissue selectivity of tissue-associated disease genes for a tissue *t* (e.g., Fig 3A), we trained an XGB model on all genes in our features dataset. Disease genes that were associated with *t* were labeled as positive, all other genes were labeled as negative. We analyzed several tissues, however were able to create satisfactory performing models only for tissues with over 60 associated disease genes (positive genes). These tissues included blood, brain, heart, liver, nerve, skeletal muscle, skin and, testis. We tested the validity of the XGB model per tissue *t* by using 10-fold cross-validation (Appendix Fig S4).

SHAP analysis of feature importance

To identify the importance of the different features per model, we applied SHAP tree algorithm to trained XGB models using default parameters (preprint: Lundberg *et al*, 2018). To enable a summarized perspective on feature importance across different models, we normalized the SHAP feature values per model by dividing the value of each feature by the sum of values of all features in that model.

To identify recurrent patterns of features per model, we associated each feature with its feature type and with its tissue of origin. For example, the feature "brain preferential expression" was associated with "preferential expression" as its feature type and with "brain" as its tissue of origin. Tissues-of-origin that were not part of the eight modeled tissues were considered as "Other," and their corresponding features were grouped together. Next, to identify recurrent feature types per model, we summed up the normalized SHAP values of features belonging to the same feature type. Likewise, to identify recurrent tissues-of-origin per model, we summed up the normalized SHAP values of features belonging to the same tissue of origin.

The TRACE ML framework for prioritizing tissue-associated disease genes

Below we describe the TRACE framework and the application of TRACE to predict genes that underlie tissue-selective diseases.

The TRACE framework

TRACE was composed of stacking of two layers. The first layer of TRACE consisted of five ML methods for training classifiers, denoted as base learners. The ML methods included logistic regression (LR), the tree-based ensemble methods XGB (described above), random forest (RF), and GBT (in the Scikit-learn implementation) initiated by a logistic regression model (LR + GB), and a multilayer perceptron (MLP) with one hidden layer. The second layer of TRACE consisted of a meta-learner MLP with two hidden layers.

In general, the input to TRACE included the features dataset and the gene labels corresponding to the specific classification task. Each base learner was applied independently to the input and produced a tissue-association score per gene. Tissue-association scores per base learner were scaled between 0 (not tissue-associated) and 10 (tissue-associated disease gene). The output of the five base learners was the input to the meta-learner, which produced a final TRACE score per gene. The TRACE score was also scaled between 0 and 10.

To reduce run time owing to the size of the features dataset and to reduce noise derived from noncontributing features, all applications and folds of XGB, LR, and LR + GB were preceded by feature selection that limited the number of relevant features. Features were selected by applying SVM with L1 regularization and setting a feature contribution threshold. Specifically, we used the python scikit-learn LinearSVC function with the C regularization parameter set to 0.1. SVM was trained on and fitted to each relevant training set, which resulted in sets of relevant features that were tailored per application and per fold. To guarantee that the SVM-based feature selection will not entirely eliminate features that will contribute through nonlinear relationships, feature selection was not applied to RF and MLP, which inherently handle noncontributing features by not selecting trees that rely on them (RF) or minimizing their weights (MLP).

We also assessed the performance of TRACE upon using only features that were derived from transcriptomics and PPIs (a total of 594 features; network embedding features were excluded). Relative to the full TRACE models, TRACE models based only on transcriptomics and PPIs had a lower AUC in 7/8 cases and a lower PR in 6/8 cases (Appendix Fig S12). This demonstrates both the major value of tissue transcriptomes and PPIs and the added value of other features.

TRACE application to predict tissue-associated disease genes

We applied TRACE to each tissue with over 60 associated disease genes, to predict tissue-associated disease genes. Per tissue *t*, the input to TRACE included the features dataset and the gene labels

corresponding to their association with that tissue (see "The labeling of genes per tissue" above). We assessed the validity of each of the base-learner models and of the meta-learner model by using 10-fold cross-validation (Appendix Fig S6). Specifically, all genes in our dataset were randomly partitioned into 10 disjoint subsets, while preserving the ratio of tissue-associated to nonassociated genes across subsets. Per fold, prior to running the base learners XGB, LR, and LR + GB, we performed feature selection and then trained the models using the selected features. Per base learner, the probabilities of genes within each subset to be associated with *t* were computed once by using a model that was trained on genes in the other nine subsets. Next, the scores of the five base learners were used as features for the second-level MLP meta-learner model. The meta-leaner model was trained on the scores of genes in the other nine subsets and was then applied to predict the final TRACE scores of the genes within the given subset.

To evaluate each model, we used the AUC, where each point on the curve corresponded to a particular cutoff, representing a trade-off between sensitivity and specificity, and the auPRC that computes the weighted mean of precisions achieved at each cutoff of the precision–recall curve. False-positive and false-negative rates per model appear in Appendix Fig S13.

Comparison to pBRIT

We applied pBRIT to model each of the eight tissues that were modeled by TRACE (Kumar et al, 2018). To mimic the input to TRACE per tissue, pBRIT was applied to the same distinct subsets that were used in TRACE. pBRIT was run via its web interface, by using the data fusion method of "TFIDF." Query gene labels were withheld from the regression.

ML implementation details

All ML methods were implemented using the Scikit-learn python package (Pedregosa *et al*, 2011), except for XGB, which was implemented using the Scikit-learn API of the XGBoost package (Chen & Guestrin, 2016). Per ML method, all hyperparameters of the models were tuned manually to achieve higher AUC and auPRC scores. Since AUC and auPRC scores of the different tissue models showed small differences per method, tuning per method was done simultaneously for the eight tissue models. The same hyperparameter values were then applied to all tissue models. For tissue-association models and for patient models, data contained mostly nondisease genes. To deal with class imbalance, the balance of positive to negative weights was set to 0.01 when training LR, XGB, RF, and the LR part of LR + GB.

XGB

The hyperparameters of XGBoost were set to build a decision forest consisting of 150 trees. Each tree had a maximum tree depth of nine. Gamma was set to 0. To prevent overfitting, we set the step size shrinkage (eta) to 0.1.

RF

The number of trees was set to 1,000.

LR

LR was used with a lbfgs solver and maximum of 100,000 iterations.

LR + GB

LR + GB was implemented by using the "GradientBoostingClassifier" function with LR parameters set with a lbfgs solver and maximum of 100,000 iterations and consisting of 80 trees.

MLP

The base-learner MLP was implemented by using the "MLPClassifier" function with two hidden layers of size 10 each and ReLU activation function. Alpha was set to 0.5. Batch size was set to 200. The meta-learner MLP was similarly implemented except that alpha was set to 0.1 and the learning rate was adaptive and initiated at 0.01.

SVM

SVM was implemented by "LinearSVC" function with a maximum of 10,000 iterations. For interpretability models, C was set to 2 and number of features was limited to 50. For TRACE prediction models, C was set to 0.1.

Analysis of tissue selectivity for distinct brain regions

We focused on the subset of diseases that were associated with brain regions with a confidence level ≥ 2 and on brain regions that were associated with at least 60 disease genes. TRACE analysis was similar to the analysis of other tissues. To assess the selectivity of diseases to a specific brain region, we compared between the scores of genes that were associated with the specific region (confidence level ≥ 2), the scores of all other brain-associated disease genes (confidence level of 3), and the scores of brain-associated disease genes that were not annotated to a specific brain region (denoted as "other" in the manually curated dataset, confidence level of 2).

The application of TRACE to prioritize candidate disease genes in patients with rare tissue-selective diseases

The criteria for selecting patient cases

All cases corresponded to genetic diseases with Mendelian inheritance that were investigated by the lab of co-author Prof. Ohad Birk. We focused on cases whose affected tissue could be modeled by TRACE, including cases presenting neurological and developmental abnormalities (modeled as brain), skin disease, muscle disease, cardiac disease, and azoospermia (modeled as testis). All cases had extensive clinical data available, undergone NGS investigations using modern techniques, high-quality data files were available, and were published. The pathogenic variant in each patient was previously successfully identified following genetic and functional analyses (Dataset EV3A).

Identification of candidate variants per patient

Patients were previously genetically diagnosed via exome sequencing and subsequent analysis, as previously described (Yogev *et al*, 2017; Drabkin *et al*, 2018; Wormser *et al*, 2019). The data per patient were deidentified, and variants were filtered as follows:

i Kept variants with call quality at least 20.0 in cases or at least 20.0 in controls AND outside top 5.0% most exonically variable 100 base windows in healthy public genomes (1,000 genomes).
ii Excluded variants that were observed with an allele frequency greater than or equal to 0.5% of the genomes in the 1000 genomes project OR greater than or equal to 0.5% of the NHLBI

 © 2023 The Authors

ESP exomes (All); or greater than or equal to 0.5% of the ExAC Frequency; or greater than or equal to 0.5% of the gnomAD Frequency; or filter variants unless established pathogenic common variant.

iii Kept variants (up to 20 bases into intron) that were experimentally observed to be associated with a phenotype: Pathogenic, possibly pathogenic or disease-associated according to HGMD; or clinically relevant variants from CentoMD; or frameshift, in-frame indel, or stop codon change, or missense, or predicted deleterious by having CADD score > 15.0; or predicted to disrupt splicing by MaxEntScan; or within 2 bases into intron.

iv In case of dominant genes, kept variants which are associated with gain of function, or hemizygous, or heterozygous, or heterozygous-amb, or compound heterozygous, or homozygous, or heterozygous-alt, or haploinsufficient and occur in at least one of the Case samples at the variant level; and not variants which are associated with gain of function, or hemizygous, or heterozygous, or heterozygous-amb, or compound heterozygous, or homozygous, or heterozygous-alt, or haploinsufficient, and occur in at least one of the control samples at the variant level in the control samples.

In case of autosomal recessive genes, kept variants which are hemizygous, or compound heterozygous, or haploinsufficient, or homozygous, and occur in at least one of the case samples at the gene level in the Case samples; and not variants which are hemizygous, or compound heterozygous, or haploinsufficient, or homozygous, and occur in at least 1 of the control samples at the variant level in the control samples.

Analyses were based on Ingenuity Variant Analysis version 5.4.20181019. Content versions: CADD (v1.3), Allele Frequency Community (2018-09-06), EVS (ESP6500SI-V2), Refseq Gene Model (2018-07-10), JASPAR (2013-11), Ingenuity Knowledge Base Snapshot Timestamp (2019-01-06 00:23:50.0), Vista Enhancer (2012-07), Clinical Trials (Stepford 190106.000), PolyPhen-2 (v2.2.2), 1000 Genome Frequency (phase3v5b), ExAC (0.3.1), iva (Oct 4 11:04 iva-1.0.736.jar), PhyloP (2009-11), DbSNP (151), TargetScan (6.2), GENCODE (Release 28), CentoMD (5.0), Ingenuity Knowledge Base (Stepford 190106.000), OMIM (May 26, 2017), gnomAD (2.0.1), BSIFT (2016-02-23), TCGA (2013-09-05), Clinvar (2018-08-01), DGV (2016-05-15), COSMIC (v86), HGMD (2018.3), and SIFT4G (2016-02-23).

TRACE analysis of patient's candidate disease genes

The data per patient were deidentified and included (i) the disease-affected tissue of the patients, and (ii) a list of variants identified in that patient that remained after the filtering described in the preceding section. Each variant was associated with its respective gene, denoted henceforth as candidate disease gene. Per patient, we created a TRACE model for each of her disease-affected tissues. Five of the 48 patients had two affected tissues, resulting in 53 ranked cases. To ensure that the model is not trained and tested on the same genes, the patient's candidate disease genes were entirely withheld from the features dataset, in order to be later prioritized by an independently trained TRACE model. The remaining, noncandidate genes, were labeled according to their association with the patient's affected tissue. In the first layer of TRACE, TRACE base learners predicted the tissue association of all noncandidate genes through 10-fold cross-validation procedure. Each base learner then predicted the tissue association of the patient's candidate disease genes by training a model on all noncandidate genes. In the second layer of TRACE, the scores of the base learners were used (once, no cross-validation), as features for the TRACE meta-learner. The meta-learner was trained on all noncandidate genes and was then used to predict the TRACE scores of the patient's candidate disease genes.

Summary of TRACE results across patients

Per patient, we ranked patient's candidate disease genes by their TRACE scores. Next, we associated the verified disease gene of the patient with its rank.

Comparison to prioritization by other methods

In the prioritization by expression levels, we ranked the candidate genes of each patient by their expression level in the modeled tissue. In the prioritization by GADO (Deelen *et al*, 2019), GADO prioritizes genes according to their similarity to genes associated with a user-selected Human Phenotype Ontology (HPO) term. Per patient, we selected the HPO term(s) corresponding to the patient's phenotypes and the disease gene information in OMIM, unless GADO recommended a different, typically more generic, HPO term (Dataset EV3). We then ranked genes according to GADO output. In the prioritization by gnomAD (Karczewski *et al*, 2020), we downloaded from gnomAD the pLoF LOEUF score and the missense score of each gene (gnomAD download page, Table "pLoF Metrics by gene TSV," downloaded on May 1, 2022). We used the upper bound scores (oe_lof_upper, oe_mis_upper) as recommended by gnomAD. We ranked the genes of each patient once by their pLoF score and once by the missense score. Per method, we associated the verified disease gene of each patient with its rank and compared the ranking by the method to the ranking by TRACE by using the Wilcoxon signed-rank test.

Analysis of top five candidate genes per patient

Per patient, we collected the top five candidate genes according to TRACE (265 genes total). We then checked how many of them participated in a gene ontology (GO) biological process that was specific to the disease-affected tissue of the patient and hence could lead to tissue-selective phenotypes. Data of tissue-specific GO biological processes were obtained from Sharon *et al* (2022).

Statistical tests

To test the null hypothesis that TRACE scores of two distinct gene sets have similar probabilities to be smaller or greater than the other, we used the Mann–Whitney U test. Correction for multiple hypothesis testing was done via the Benjamini–Hochberg procedure. To test the null hypothesis that ranking by expression, by GADO (Deelen *et al*, 2019), or by gnomAD scores (Karczewski *et al*, 2020) was better or equal to TRACE ranking, we used Wilcoxon signed-rank test.

TRACE webserver

The TRACE webserver was implemented in Python by using the Flask framework with data stored on a MySQL database. The website client was developed using the ReactJS framework and designed with Semantic-UI. The charts were displayed by the Google

© 2023 The Authors

Charts library. The TRACE webserver supports all major browsers. The webserver presents the TRACE scores of input genes in the user-selected tissue. TRACE scores were based on 10-fold cross-validation and computed as described in section "TRACE application to predict tissue-associated disease genes."

Data availability

All data are available as EV Datasets and in https://sandbox.zenodo.org/record/1185590#.ZES8M3ZByUk. The code for running TRACE is available in https://github.com/eyalsim/trace.

Expanded View for this article is available online.

Acknowledgements

This study was funded by the Israel Science Foundation (317/19 and 401/22 to EY-L) and by a Ben-Gurion University grant (to EY-L and LR).

Author contributions

Eyal Simonovsky: Conceptualization; formal analysis; investigation; methodology; writing – original draft; writing – review and editing. **Moran Sharon:** Data curation; formal analysis; validation; investigation; methodology; writing – original draft; writing – review and editing. **Maya Ziv:** Data curation; formal analysis; investigation; methodology. **Omry Mauer:** Software; visualization. **Idan Hekselman:** Data curation; investigation. **Juman Jubran:** Formal analysis; investigation. **Ekaterina Vinogradov:** Visualization. **Chanan M Argov:** Investigation. **Omer Basha:** Resources; software. **Lior Kerber:** Data curation. **Yuval Yogev:** Resources; formal analysis; validation; investigation; writing – original draft; writing – review and editing. **Ayellet V Segrè:** Resources; software; methodology. **Hae Kyung Im:** Resources; software. **Ohad Birk:** Resources. **Lior Rokach:** Methodology; writing – original draft; writing – review and editing. **Esti Yeger-Lotem:** Conceptualization; resources; supervision; validation; investigation; methodology; writing – original draft; project administration; writing – review and editing.

In addition to the CRediT author contributions listed above, the contributions in detail are:

Conceptualization: ES and EY-L; Methodology: ES, LR and EY-L; Investigation: ES, MS, MZ, JJ, CMA; Formal analysis: ES, MS, MZ, JJ, CMA, YY; Webserver: OM, OB; Visualization: EV; Data acquisition: IH, LK; Data contribution: GC, AVS, HKI, YY, OB; Writing: ES, EYL; Supervision: EYL; Funding acquisition: LR, EY-L.

Disclosure and competing interests statement

The authors declare that they have no conflict of interest.

References

Adzhubei I, Jordan DM, Sunyaev SR (2013) Predicting functional effect of human missense mutations using PolyPhen-2. *Curr Protoc Hum Genet* Chapter 7: Unit7.20

Aerts S, Lambrechts D, Maity S, Van Loo P, Coessens B, De Smet F, Tranchevent LC, De Moor B, Marynen P, Hassan B *et al* (2006) Gene prioritization through genomic data fusion. *Nat Biotechnol* 24: 537–544

Amberger JS, Bocchini CA, Scott AF, Hamosh A (2019) OMIM.Org: leveraging knowledge across phenotype-gene relationships. *Nucleic Acids Res* 47: D1038–D1043

Barbeira AN, Dickinson SP, Bonazzola R, Zheng J, Wheeler HE, Torres JM, Torstenson ES, Shah KP, Garcia T, Edwards TL *et al* (2018) Exploring the phenotypic consequences of tissue specific gene expression variation inferred from GWAS summary statistics. *Nat Commun* 9: 1825

Barbeira AN, Bonazzola R, Gamazon ER, Liang Y, Park Y, Kim-Hellmuth S, Wang G, Jiang Z, Zhou D, Hormozdiari F *et al* (2019) Widespread dose-dependent effects of RNA expression and splicing on complex diseases and traits. *bioRxiv* https://doi.org/10.1101/814350 [PREPRINT]

Barshir R, Shwartz O, Smoly IY, Yeger-Lotem E (2014) Comparative analysis of human tissue interactomes reveals factors leading to tissue-specific manifestation of hereditary diseases. *PLoS Comput Biol* 10: e1003632

Barshir R, Hekselman I, Shemesh N, Sharon M, Novack L, Yeger-Lotem E (2018) Role of duplicate genes in determining the tissue-selectivity of hereditary diseases. *PLoS Genet* 14: e1007327

Basha O, Flom D, Barshir R, Smoly I, Tirman S, Yeger-Lotem E (2015) MyProteinNet: build up-to-date protein interaction networks for organisms, tissues and user-defined contexts. *Nucleic Acids Res* 43: W258–W263

Basha O, Barshir R, Sharon M, Lerman E, Kirson BF, Hekselman I, Yeger-Lotem E (2017) The TissueNet v.2 database: a quantitative view of protein-protein interactions across human tissues. *Nucleic Acids Res* 45: D427–D431

Basha O, Shpringer R, Argov CM, Yeger-Lotem E (2018) The DifferentialNet database of differential protein-protein interactions in human tissues. *Nucleic Acids Res* 46: D522–D526

Basha O, Argov CM, Artzy R, Zoabi Y, Hekselman I, Alfandari L, Chalifa-Caspi V, Yeger-Lotem E (2020) Differential network analysis of multiple human tissue interactomes highlights tissue-selective processes and genetic disorder genes. *Bioinformatics* 36: 2821–2828

Canepari M, Rossi R, Pansarasa O, Maffei M, Bottinelli R (2009) Actin sliding velocity on pure myosin isoforms from dystrophic mouse muscles. *Muscle Nerve* 40: 249–256

Cardoso-Moreira M, Halbert J, Valloton D, Velten B, Chen C, Shao Y, Liechti A, Ascencao K, Rummel C, Ovchinnikova S *et al* (2019) Gene expression across mammalian organ development. *Nature* 571: 505–509

Chen T, Guestrin C (2016) XGBoost: a scalable tree boosting system. *Proceedings of the 22nd ACM SIGKDD International Conference on Knowledge Discovery and Data Mining*: 785–794

Chiu YC, Zheng S, Wang LJ, Iskra BS, Rao MK, Houghton PJ, Huang Y, Chen Y (2021) Predicting and characterizing a cancer dependency map of tumors with deep learning. *Sci Adv* 7: eabh1275

Chong JX, Buckingham KJ, Jhangiani SN, Boehm C, Sobreira N, Smith JD, Harrell TM, McMillin MJ, Wiszniewski W, Gambin T *et al* (2015) The genetic basis of mendelian phenotypes: discoveries, challenges, and opportunities. *Am J Hum Genet* 97: 199–215

Cummings BB, Marshall JL, Tukiainen T, Lek M, Donkervoort S, Foley AR, Bolduc V, Waddell LB, Sandaradura SA, O'Grady GL *et al* (2017) Improving genetic diagnosis in mendelian disease with transcriptome sequencing. *Sci Transl Med* 9: eaal5209

Cummings BB, Karczewski KJ, Kosmicki JA, Seaby EG, Watts NA, Singer-Berk M, Mudge JM, Karjalainen J, Satterstrom FK, O'Donnell-Luria AH *et al* (2020) Transcript expression-aware annotation improves rare variant interpretation. *Nature* 581: 452–458

Davis CA, Hitz BC, Sloan CA, Chan ET, Davidson JM, Gabdank I, Hilton JA, Jain K, Baymuradov UK, Narayanan AK *et al* (2018) The encyclopedia of DNA elements (ENCODE): data portal update. *Nucleic Acids Res* 46: D794–D801

Deelen P, van Dam S, Herkert JC, Karjalainen JM, Brugge H, Abbott KM, van Diemen CC, van der Zwaag PA, Gerkes EH, Zonneveld-Huijssoon E *et al*

 © 2023 The Authors

(2019) Improving the diagnostic yield of exome-sequencing by predicting gene-phenotype associations using large-scale gene expression analysis. *Nat Commun* 10: 2837

Drabkin M, Zilberberg N, Menahem S, Mulla W, Halperin D, Yogev Y, Wormser O, Perez Y, Kadir R, Etzion Y *et al* (2018) Nocturnal atrial fibrillation caused by mutation in KCND2, encoding pore-forming (alpha) subunit of the cardiac Kv4.2 Potassium Channel. *Circ Genom Precis Med* 11: e002293

Dwivedi SK, Tjarnberg A, Tegner J, Gustafsson M (2020) Deriving disease modules from the compressed transcriptional space embedded in a deep autoencoder. *Nat Commun* 11: 856

Eilbeck K, Quinlan A, Yandell M (2017) Settling the score: variant prioritization and mendelian disease. *Nat Rev Genet* 18: 599–612

Fernandez-Delgado M, Cernadas E, Barro S, Amorim D (2014) Do we need hundreds of classifiers to solve real world classification problems? *J Mach Learn Res* 15: 3133–3181

Gamazon ER, Segre AV, van de Bunt M, Wen X, Xi HS, Hormozdiari F, Ongen H, Konkashbaev A, Derks EM, Aguet F *et al* (2018) Using an atlas of gene regulation across 44 human tissues to inform complex disease- and trait-associated variation. *Nat Genet* 50: 956–967

Gamazon ER, Zwinderman AH, Cox NJ, Denys D, Derks EM (2019) Multi-tissue transcriptome analyses identify genetic mechanisms underlying neuropsychiatric traits. *Nat Genet* 51: 933–940

Gelfman S, Wang Q, McSweeney KM, Ren Z, La Carpia F, Halvorsen M, Schoch K, Ratzon F, Heinzen EL, Boland MJ *et al* (2017) Annotating pathogenic non-coding variants in genic regions. *Nat Commun* 8: 236

Gene Ontology Consortium (2015) Gene Ontology consortium: going forward. *Nucleic Acids Res* 43: D1049–D1056

Gligorijevic V, Przulj N (2015) Methods for biological data integration: perspectives and challenges. *J R Soc Interface* 12: 20150571

Goedert M, Jakes R, Spillantini MG (2017) The Synucleinopathies: twenty years on. *J Parkinsons Dis* 7: S51–S69

Greene CS, Krishnan A, Wong AK, Ricciotti E, Zelaya RA, Himmelstein DS, Zhang R, Hartmann BM, Zaslavsky E, Sealfon SC *et al* (2015) Understanding multicellular function and disease with human tissue-specific networks. *Nat Genet* 47: 569–576

Grover A, Leskovec J (2016) node2vec: scalable feature learning for networks. *KDD* 2016: 855–864

GTEx Consortium (2020) The GTEx consortium atlas of genetic regulatory effects across human tissues. *Science* 369: 1318–1330

Halperin D, Kadir R, Perez Y, Drabkin M, Yogev Y, Wormser O, Berman EM, Eremenko E, Rotblat B, Shorer Z *et al* (2019) SEC31A mutation affects ER homeostasis, causing a neurological syndrome. *J Med Genet* 56: 139–148

Hekselman I, Yeger-Lotem E (2020) Mechanisms of tissue and cell-type specificity in heritable traits and diseases. *Nat Rev Genet* 21: 137–150

Hekselman I, Kerber L, Ziv M, Gruber G, Yeger-Lotem E (2022) The organ-disease annotations (ODiseA) database of hereditary diseases and inflicted tissues. *J Mol Biol* 434: 167619

Hernandez DG, Reed X, Singleton AB (2016) Genetics in Parkinson disease: mendelian versus non-mendelian inheritance. *J Neurochem* 139: 59–74

Holmans PA, Massey TH, Jones L (2017) Genetic modifiers of mendelian disease: Huntington's disease and the trinucleotide repeat disorders. *Hum Mol Genet* 26: R83–R90

Huttlin EL, Bruckner RJ, Paulo JA, Cannon JR, Ting L, Baltier K, Colby G, Gebreab F, Gygi MP, Parzen H *et al* (2017) Architecture of the human interactome defines protein communities and disease networks. *Nature* 545: 505–509

100,000 Genomes Project Pilot Investigators, Smedley D, Smith KR, Martin A, Thomas EA, McDonagh EM, Cipriani V, Ellingford JM, Arno G, Tucci A *et al* (2021) 100,000 genomes pilot on rare-disease diagnosis in health care – preliminary report. *N Engl J Med* 385: 1868–1880

Jubran J, Hekselman I, Novack L, Yeger-Lotem E (2020) Dosage-sensitive molecular mechanisms are associated with the tissue-specificity of traits and diseases. *Comput Struct Biotechnol J* 18: 4024–4032

Karczewski KJ, Francioli LC, Tiao G, Cummings BB, Alföldi J, Wang Q, Collins RL, Laricchia KM, Ganna A, Birnbaum DP *et al* (2020) The mutational constraint spectrum quantified from variation in 141,456 humans. *Nature* 581: 434–443

Khalilia M, Chakraborty S, Popescu M (2011) Predicting disease risks from highly imbalanced data using random forest. *BMC Med Inform Decis Mak* 11: 51

Kitsak M, Sharma A, Menche J, Guney E, Ghiassian SD, Loscalzo J, Barabasi AL (2016) Tissue specificity of human disease module. *Sci Rep* 6: 35241

Kumar AA, Van Laer L, Alaerts M, Ardeshirdavani A, Moreau Y, Laukens K, Loeys B, Vandeweyer G (2018) pBRIT: gene prioritization by correlating functional and phenotypic annotations through integrative data fusion. *Bioinformatics* 34: 2254–2262

Lage K, Hansen NT, Karlberg EO, Eklund AC, Roque FS, Donahoe PK, Szallasi Z, Jensen TS, Brunak S (2008) A large-scale analysis of tissue-specific pathology and gene expression of human disease genes and complexes. *Proc Natl Acad Sci USA* 105: 20870–20875

Landrum MJ, Lee JM, Benson M, Brown G, Chao C, Chitipiralla S, Gu B, Hart J, Hoffman D, Hoover J *et al* (2016) ClinVar: public archive of interpretations of clinically relevant variants. *Nucleic Acids Res* 44: D862–D868

Luck K, Kim DK, Lambourne L, Spirohn K, Begg BE, Bian W, Brignall R, Cafarelli T, Campos-Laborie FJ, Charloteaux B *et al* (2020) A reference map of the human binary protein interactome. *Nature* 580: 402–408

Lundberg S, Erion G, Lee S (2018) Consistent individualized feature attribution for tree ensembles. *arXiv* https://doi.org/10.48550/arXiv.1802.03888 [PREPRINT]

Lundberg SM, Erion G, Chen H, DeGrave A, Prutkin JM, Nair B, Katz R, Himmelfarb J, Bansal N, Lee SI (2020) From local explanations to global understanding with explainable AI for trees. *Nat Mach Intell* 2: 56–67

Magger O, Waldman YY, Ruppin E, Sharan R (2012) Enhancing the prioritization of disease-causing genes through tissue specific protein interaction networks. *PLoS Comput Biol* 8: e1002690

Malod-Dognin N, Petschnigg J, Windels SFL, Povh J, Hemingway H, Ketteler R, Przulj N (2019) Towards a data-integrated cell. *Nat Commun* 10: 805

Marbach D, Lamparter D, Quon G, Kellis M, Kutalik Z, Bergmann S (2016) Tissue-specific regulatory circuits reveal variable modular perturbations across complex diseases. *Nat Methods* 13: 366–370

McCarthy MI, MacArthur DG (2017) Human disease genomics: from variants to biology. *Genome Biol* 18: 20

McKusick-Nathans Institute of Genetic Medicine (JHUB, MD) (n.d.) *Online mendelian inheritance in man, OMIM®*. Baltimore, MD: McKusick-Nathans Institute of Genetic Medicine, Johns Hopkins University. https://omim.org

Moaven N, Tayebi N, Goldin E, Sidransky E (2015) Complexity of genotype-phenotype correlations in mendelian disorders: lessons from Gaucher disease. In *Rare diseases*, pp 69–90. Heidelberg: Springer Netherlands

Moustafa AA, Phillips J, Keri S, Misiak B, Frydecka D (2016) On the complexity of brain disorders: a symptom-based approach. *Front Comput Neurosci* 10: 16

Oughtred R, Stark C, Breitkreutz BJ, Rust J, Boucher L, Chang C, Kolas N, O'Donnell L, Leung G, McAdam R *et al* (2019) The BioGRID interaction database: 2019 update. *Nucleic Acids Res* 47: D529–D541

© 2023 The Authors

Pedregosa F, Varoquaux G, Gramfort A, Michel V, Thirion B, Grisel O, Blondel M, Prettenhofer P, Weiss R, Dubourg V *et al* (2011) Scikit-learn: machine learning in python. *J Mach Learn Res* 12: 2825–2830

Proskorovski-Ohayon R, Kadir R, Michalowski A, Flusser H, Perez Y, Hershkovitz E, Sivan S, Birk OS (2017) PAX7 mutation in a syndrome of failure to thrive, hypotonia, and global neurodevelopmental delay. *Hum Mutat* 38: 1671–1683

Rentzsch P, Witten D, Cooper GM, Shendure J, Kircher M (2019) CADD: predicting the deleteriousness of variants throughout the human genome. *Nucleic Acids Res* 47: D886–D894

Robinson MD, McCarthy DJ, Smyth GK (2010) edgeR: a Bioconductor package for differential expression analysis of digital gene expression data. *Bioinformatics* 26: 139–140

Schwarz DF, Konig IR, Ziegler A (2010) On safari to random jungle: a fast implementation of random forests for high-dimensional data. *Bioinformatics* 26: 1752–1758

Sharon M, Vinogradov E, Argov CM, Lazarescu O, Zoabi Y, Hekselman I, Yeger-Lotem E (2022) The differential activity of biological processes in tissues and cell subsets can illuminate disease-related processes and cell type identities. *Bioinformatics* 38: 1584–1592

Simonovsky E, Schuster R, Yeger-Lotem E (2019) Large-scale analysis of human gene expression variability associates highly variable drug targets with lower drug effectiveness and safety. *Bioinformatics* 35: 3028–3037

Smedley D, Jacobsen JO, Jager M, Kohler S, Holtgrewe M, Schubach M, Siragusa E, Zemojtel T, Buske OJ, Washington NL *et al* (2015) Next-generation diagnostics and disease-gene discovery with the exomiser. *Nat Protoc* 10: 2004–2015

Somepalli G, Sahoo S, Singh A, Hannenhalli S (2021) Prioritizing and characterizing functionally relevant genes across human tissues. *PLoS Comput Biol* 17: e1009194

Sonawane AR, Platig J, Fagny M, Chen CY, Paulson JN, Lopes-Ramos CM, DeMeo DL, Quackenbush J, Glass K, Kuijjer ML (2017) Understanding tissue-specific gene regulation. *Cell Rep* 21: 1077–1088

The Gene Ontology Consortium (2019) The Gene Ontology resource: 20 years and still GOing strong. *Nucleic Acids Res* 47: D330–D338

Uhlen M, Fagerberg L, Hallstrom BM, Lindskog C, Oksvold P, Mardinoglu A, Sivertsson A, Kampf C, Sjostedt E, Asplund A *et al* (2015) Proteomics. tissue-based map of the human proteome. *Science* 347: 1260419

van Buuren S, Groothuis-Oudshoorn K (2011) Mice: multivariate imputation by chained equations in R. *J Stat Softw* 45: 1–67

Vilalta R, Drissi Y (2002) A perspective view and survey of meta-learning. *Artif Intell Rev* 18: 77–95

Wesolowska-Andersen A, Zhuo YG, Nylander V, Abaitua F, Thurner M, Torres JM, Mahajan A, Gloyn AL, McCarthy MI (2020) Deep learning models predict regulatory variants in pancreatic islets and refine type 2 diabetes association signals. *Elife* 9: e51503

Wong AK, Sealfon RSG, Theesfeld CL, Troyanskaya OG (2021) Decoding disease: from genomes to networks to phenotypes. *Nat Rev Genet* 22: 774–790

Wormser O, Gradstein L, Yogev Y, Perez Y, Kadir R, Goliand I, Sadka Y, El Riati S, Flusser H, Nachmias D *et al* (2019) SCAPER localizes to primary cilia and its mutation affects cilia length, causing Bardet-Biedl syndrome. *Eur J Hum Genet* 27: 928–940

Yao DW, O'Connor LJ, Price AL, Gusev A (2020) Quantifying genetic effects on disease mediated by assayed gene expression levels. *Nat Genet* 52: 626–633

Yeo IK, Johnson RA (2000) A new family of power transformations to improve normality or symmetry. *Biometrika* 87: 954–959

Yogev Y, Perez Y, Noyman I, Madegem AA, Flusser H, Shorer Z, Cohen E, Kachko L, Michaelovsky A, Birk R *et al* (2017) Progressive hereditary spastic paraplegia caused by a homozygous KY mutation. *Eur J Hum Genet* 25: 966–972

License: This is an open access article under the terms of the Creative Commons Attribution License, which permits use, distribution and reproduction in any medium, provided the original work is properly cited.

 © 2023 The Authors

Expanded View Figures

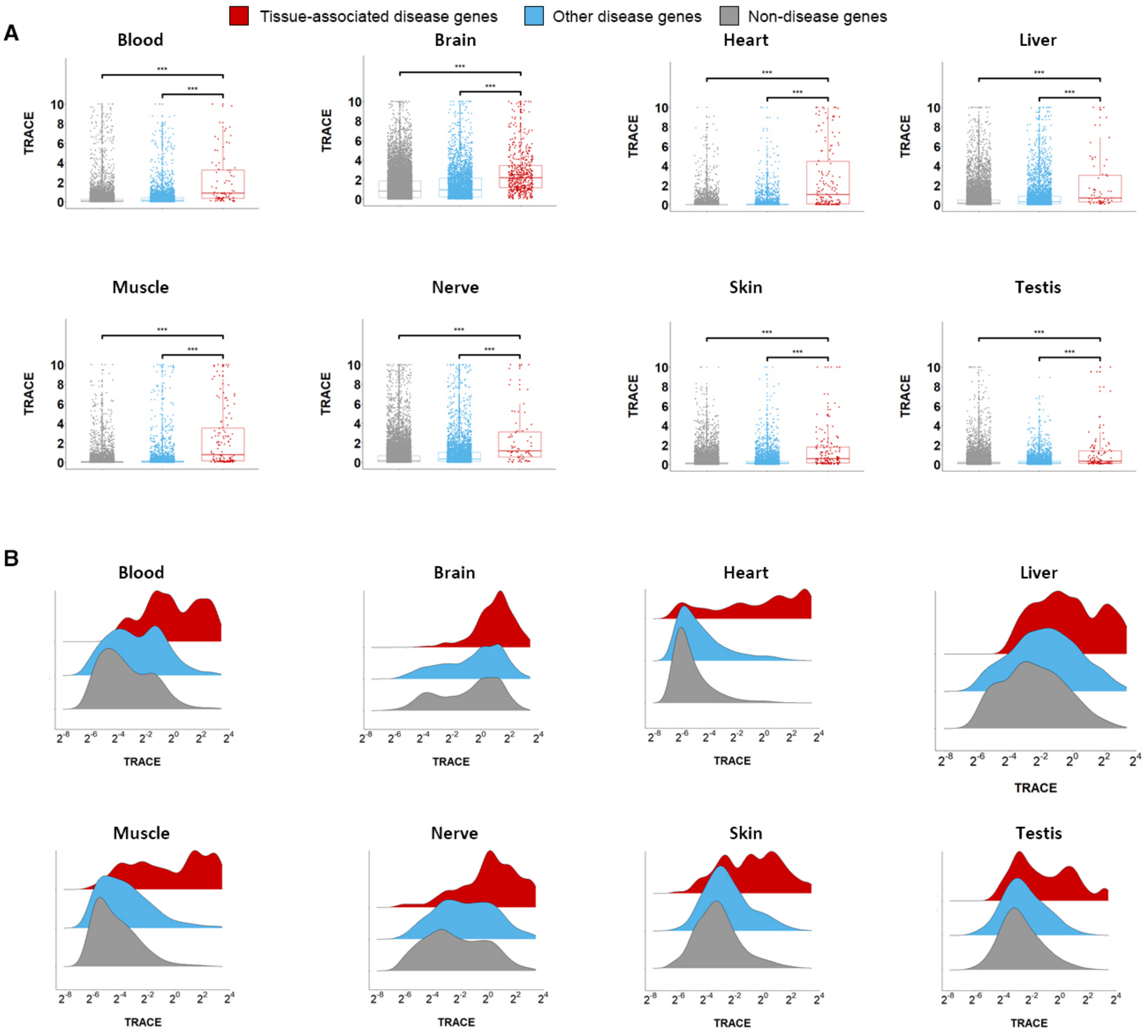

Figure EV1. TRACE prioritization of genes associated with diseases that manifest in distinct tissues.

A Gene TRACE scores in the different tissue models. Each dot represents a different gene. Genes were divided into genes that are causal for a disease that manifests in the modeled tissue (tissue-associated, red), genes that are causal for a disease that does not manifest in the modeled tissue (other disease genes, blue), and nondisease genes (gray). Tissue-associated disease genes had significantly higher TRACE scores compared with nondisease genes and to other disease genes. Mann–Whitney *U* test (MW) adjusted *P*-values for the comparison of tissue-associated genes to noncausal genes: Blood ($2.85*10^{-16}$), brain ($2.85*10^{-16}$), heart ($2.85*10^{-16}$), liver ($4.44*10^{-13}$), skeletal muscle ($2.85*10^{-16}$), nerve ($2.85*10^{-16}$), skin ($2.85*10^{-16}$), testis ($2.85*10^{-16}$). MW adjusted *P*-values for the comparison of tissue-associated genes to other disease genes: Blood ($2.22*10^{-16}$), brain ($2.22*10^{-16}$), heart ($2.22*10^{-16}$), liver ($1.10*10^{-6}$), skeletal muscle ($2.22*10^{-16}$), nerve ($3.70*10^{-11}$), skin ($2.22*10^{-16}$), and testis ($9.80*10^{-12}$). Boxplot central band indicates median; box limits indicate 25th to 75th percentiles; whiskers indicate 1.5 × interquartile range. The number of analyzed values was ~ 60–500 for tissue-associated genes, ~ 3,430–3,860 for other disease genes, and ~ 15,000 for nondisease genes. Panels relating to brain, heart, skeletal muscle, and skin are the same as those in Fig 4D.

B Density ridge plots of gene TRACE scores in the different tissue models. Tissue-associated genes (red) were over-represented among genes with high TRACE scores.

Panels relating to brain, heart, skeletal muscle, and skin are the same as those in Fig 4E.

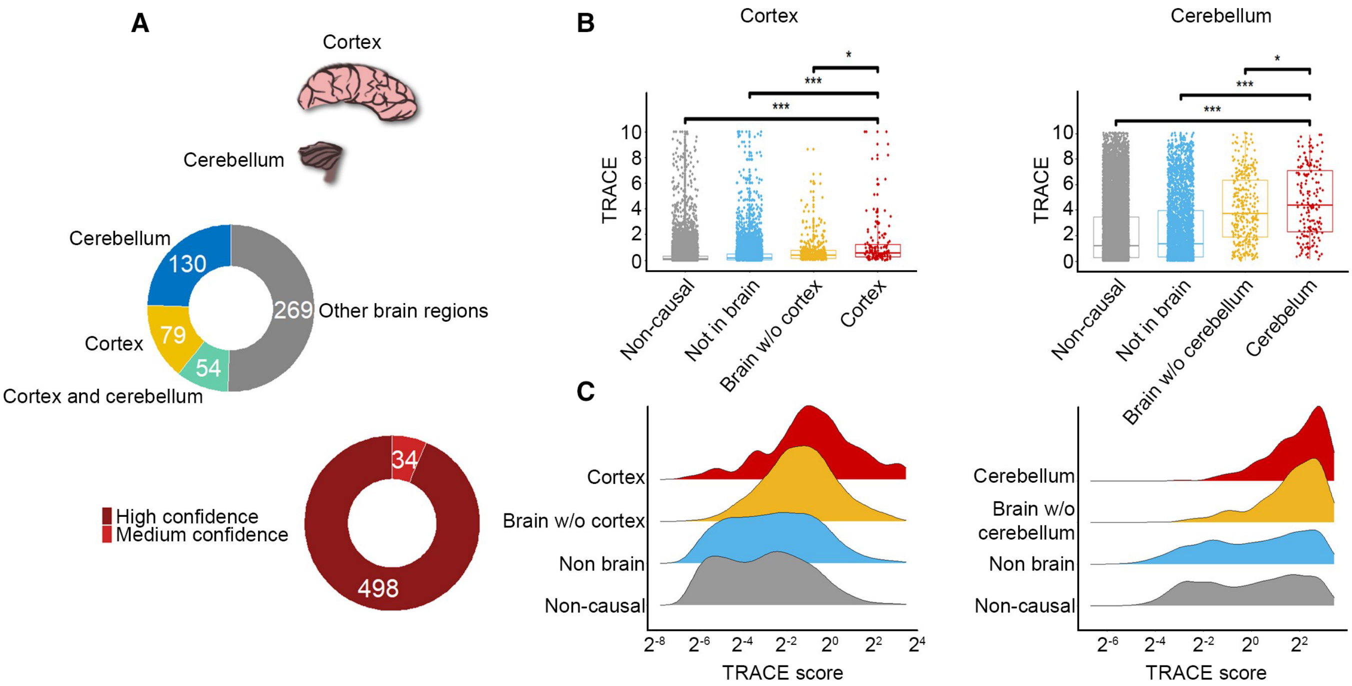

Figure EV2. TRACE prioritization of genes associated with diseases that manifest in brain cortex and cerebellum.

A The 532 genes known to be causal for brain diseases were associated with brain regions that manifest the disease at medium to high confidence. Most genes were associated with cerebellum and cortex.

B Gene TRACE scores in brain cortex and cerebellum models. Each dot represents a different gene. Genes were divided into nondisease genes (noncausal, gray), disease genes that are not causal for brain diseases (nonbrain, blue), disease genes that are causal for brain diseases that do not manifest in the modeled brain region (brain w/o modeled region, orange), and disease genes that are causal for brain diseases that manifest in the modeled brain region (modeled brain region, red). Genes associated with the modeled brain region had significantly higher TRACE scores compared with all other gene sets (Cortex: *** = 3.3E-16, * = 0.028; cerebellum: *** = 3.3E-16, * = 0.033; MW, adjusted *P*-values shown). Boxplot central band indicates median; box limits indicate 25th to 75th percentiles; whiskers indicate 1.5 × interquartile range. The number of analyzed values was ~130–180 for disease genes of the modeled brain region, ~320–370 other brain disease genes, ~3,420 for other disease genes, and ~15,000 for nondisease genes.

C Density ridge plots of gene TRACE scores in brain cortex and cerebellum models. Genes associated with the modeled region were over-represented among genes with high TRACE scores.

 © 2023 The Authors

APPENDIX: Predicting molecular mechanisms of hereditary diseases by using their tissue-selective manifestation

Eyal Simonovsky, Moran Sharon, Maya Ziv, Omry Mauer, Idan Hekselman, Juman Jubran, Ekaterina Vinogradov, Chanan M. Argov, Omer Basha, Lior Kerber, Yuval Yogev, Ayellet V. Segrè, Hae Kyung Im, GTEx Consortium, Ohad Birk, Lior Rokach, Esti Yeger-Lotem

Contents

This file contains Appendix Figures S1-13 and Appendix Table S1

Appendix Figure S1 ………….. 2

Appendix Figure S2 ………….. 3

Appendix Figure S3 ………….. 4

Appendix Figure S4 ………….. 6

Appendix Figure S5 ………….. 7

Appendix Figure S6 ………….. 9

Appendix Figure S7 ………….. 10

Appendix Figure S8 ………….. 11

Appendix Figure S9 ………….. 12

Appendix Figure S10 ………….. 13

Appendix Figure S11 ………….. 14

Appendix Figure S12 ………….. 15

Appendix Figure S13 ………….. 16

Appendix Table S1 ………….. 18

Membership of the GTEx Consortium … 22

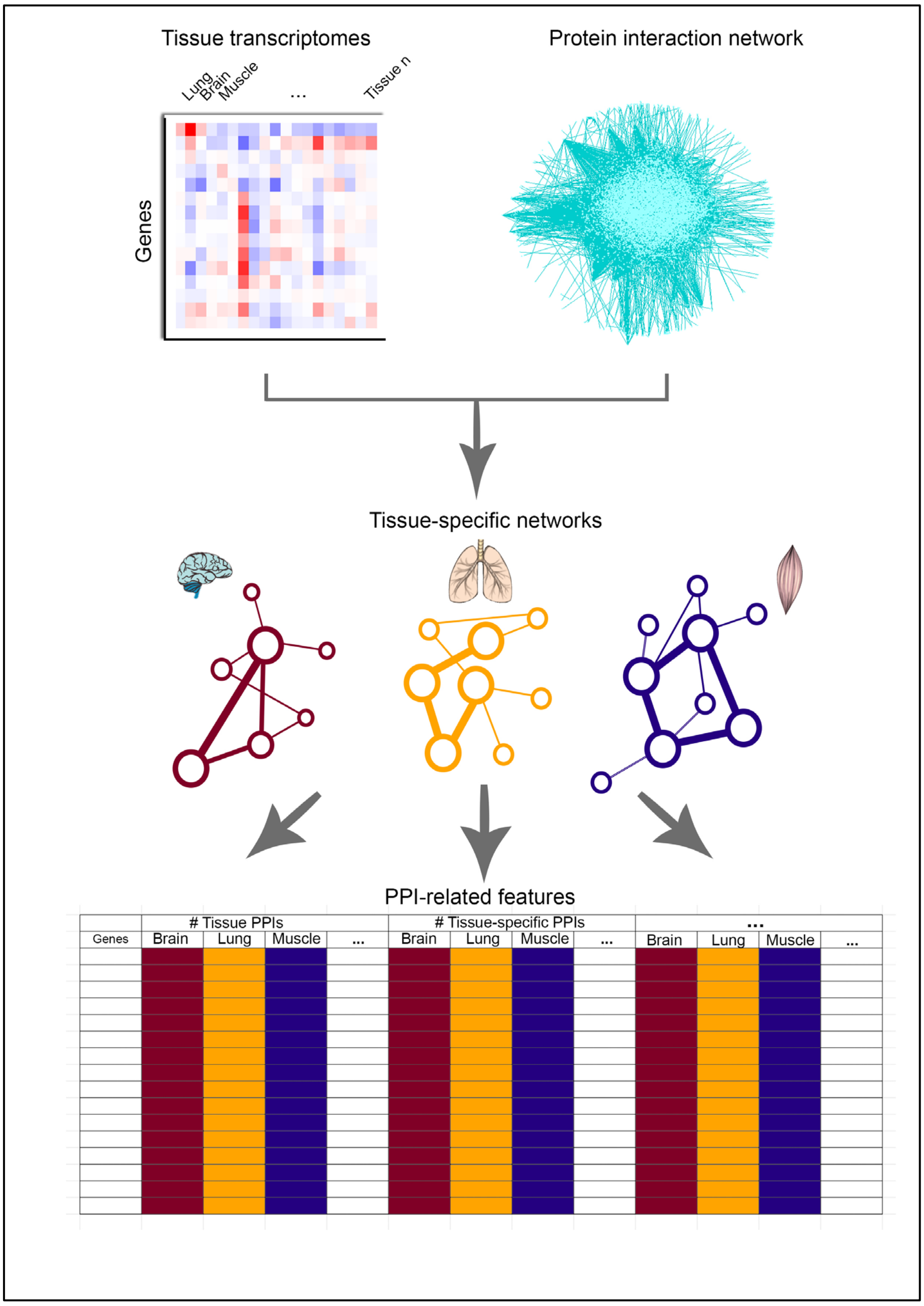

Appendix Fig. S1. The generation of gene features by integrating protein-protein interactions (PPI) and tissue transcriptomes. Tissue transcriptomes were combined with data of experimentally-detected PPI between human proteins to create tissue-specific PPI networks. Each tissue-specific network included the subset of tissue-expressed genes and the PPIs of their protein products (node size and edge width reflect the relative expression levels of corresponding genes). The resulting networks gave rise to multiple gene-based features per tissue network. For example, # tissue PPIs counts the number of gene-related PPIs in that tissue network; # tissue-specific PPIs counts the number of gene-related PPIs in that tissue network that do not appear in most other tissue networks.

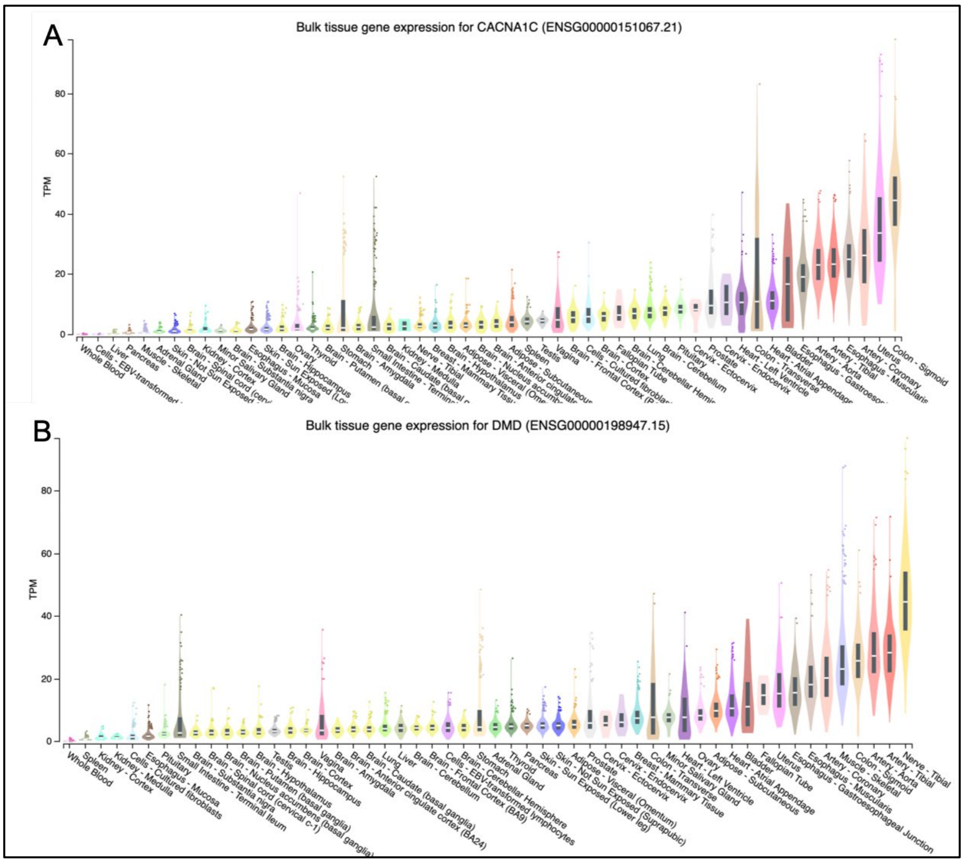

Appendix Fig. S2. The expression levels of CACNA1C and DMD in adult human tissues. Figures were obtained from the GTEx portal (https://www.gtexportal.org/home/), with tissues ordered according to the median expression level of the query gene. The median expression level of CACNA1C and DMD were above 1TPM in over 80% of the tissues. The median expression level of CACNA1C in heart, its disease-affected tissue, was 25% of its maximal median expression level in any tissue. The median expression level of DMD in skeletal muscle, its disease-affected tissue, was 52% of its maximal median expression level in any tissue.

A

The gene CACNA1C is associated with 18 processes:

Low ProAct score High

																											T27	T28	T29
P1	0.37	0.01	0.34	0.42	0.05	-0.89	-1.87	-0.59	0.69	0.31	-0.17	-0.69	0.42	0.22	0.39	-1.25	0.57	0.34	-0.18	-0.67	0.57	0.64	0.54	0.67	-1.54	0.4	0.17	0.45	-0.26
P2	0.09	-0.12	0.42	0.27	0.77	0.45	0.02	0.49	-0.29	0.25	-0.04	-0.85	0.43	0.89	1.34	-1.63	-0.16	1.25	-0.22	-0.66	-0.54	-0.28	-0.54	-0.68	-1.77	-0.62	-0.13	-0.29	-2.23
P3	0.56	-0.13	-0.57	-0.24	0.5	-0.87	-1.25	-1.19	0.77	0.02	-0.14	-0.08	0.2	-0.12	-0.23	-1.63	0.18	-0.04	0.74	-0.57	-0.06	0.94	0.93	1.13	0.61	-0.34	0.69	0.72	-2.54
P4	0.09	-0.24	-0.29	0.16	0.04	1.9	0.6	1.51	-0.36	0.69	-0.16	-1.34	0.61	0.33	0.06	-1.72	-0.16	-1.98	0.04	-1.7	0.07	-0.42	-0.3	-0.33	-1.62	-1.26	-0.37	-0.22	-0.59
P5	-0.18	-0.03	-0.12	0.02	0.02	0.18	-0.47	0.23	-0.08	-0.26	0.01	-0.2	0.02	-0.01	-0.17	-0.73	-0.03	-0.51	0.02	-0.25	0.31	0.1	0.04	0.06	0.6	-0.24	0	0.17	-1.99
P6	-0.79	-0.46	-0.05	-0.1	-0.19	1.8	0.16	1.95	-0.41	0.17	-0.05	-0.71	0.05	-0.27	-0.55	-1.2	0.26	-1.44	0.12	-0.64	0.6	0.2	-0.57	-0.56	0.08	-0.53	0.39	-0.14	-1.36
P7	-0.28	0.04	-0.21	-0.18	-0.29	0.72	0.11	0.39	-0.01	-0.01	-0.05	-0.33	-0.16	0.01	0	-0.57	0.01	-0.7	-0.14	-0.77	0.46	-0.04	-0.22	-0.26	-0.25	-0.41	-0.08	-0.21	-0.9
P8	-0.2	-0.09	-0.45	0.09	-0.42	1.52	1.22	1.25	-0.72	0.15	0	-0.53	0.44	-0.15	0.49	-2.38	0.09	-0.28	-1.39	-1	-0.34	0.31	-0.53	-0.37	-0.5	-1.16	0.66	-0.27	-2.6
P9	-0.51	-0.15	-0.46	-0.13	-0.53	1.42	1.01	1.33	-0.36	-0.1	0.05	-0.98	-0.11	0.5	0.08	-1.64	-0.16	-0.6	-0.46	-0.7	0.44	-0.15	-0.47	-0.49	-0.29	-0.67	-0.02	-0.04	-1.96
P10	0.04	-0.09	0.79	0.08	0.54	0.86	0.59	0.35	-0.42	0.89	-0.29	-0.55	0.77	0.28	0.6	-3	0.19	-0.13	-0.68	-0.2	0.2	0.18	-0.35	-0.62	-1.11	-0.4	1.06	-0.18	-4.31
P11	-0.37	-0.19	-0.7	-0.19	-0.1	1.36	0.97	1.03	-0.56	0.13	-0.04	-0.74	-0.45	0.42	-0.37	-0.49	-0.11	-1.74	-0.47	-0.56	0.45	0.12	-0.46	-0.39	-0.15	-0.42	0.35	-0.06	-1.12
P12	-0.43	-0.31	-0.71	-0.14	-1.16	1.55	1.35	1.24	-0.08	0.17	-0.16	-0.88	-0.34	1.24	1.33	-2.11	0.07	0.13	-0.33	-0.85	0.32	-0.34	-0.51	-0.82	-0.89	-0.57	-0.01	-0.07	-2.86
P13	-0.75	-0.39	-1.1	-0.31	-1.05	2.09	1.63	1.96	-0.4	0.19	-0.17	-1.05	-0.13	1.31	1.83	-3.14	-0.33	0.23	-0.53	-1.8	0.54	-0.17	-0.8	-0.8	-1.7	-1.1	0.18	-0.22	-2.79
P14	-0.46	-0.18	0.58	-0.44	0.37	0.68	0.48	0.08	-0.11	0.65	-0.31	-0.56	0.29	2.11	1.81	-2.15	-0.4	-1.2	-0.71	0	-0.2	0.91	-0.15	-0.09	-0.69	-0.77	0.91	0.68	-3.74
P15	-0.39	-0.31	-0.05	0.2	-0.5	1.45	1.27	1.65	-0.6	-0.09	-0.17	-0.6	0.29	1.04	1.83	-2.23	-0.27	-1.26	-0.88	-0.68	0.25	-0.37	-0.77	-0.68	-1.35	-0.93	-0.28	-0.54	-2.51
P16	-0.05	-0.06	-0.38	-0.16	-0.52	0.8	0.19	0.58	-0.07	0.13	-0.16	-0.55	-0.08	1.44	1.01	-1.13	0.1	-0.08	-0.3	-0.59	0.49	0.32	-0.45	-0.44	-1.11	-0.42	-0.08	0.04	-2.57
P17	0.32	0.12	-0.01	0.13	0.13	-0.94	-1.19	-0.45	0.37	0.03	-0.34	1.14	0.09	1.67	2.12	0	0.29	-0.63	0.08	-0.27	-0.48	0.73	0.3	0.5	-1.06	0.36	-0.42	0.07	-3.44
P18	-0.78	-0.74	1.07	-0.07	0.9	0.43	-0.24	0.36	-0.28	0.82	-0.27	-1.02	0.98	2.18	3.07	-1.88	-0.62	-1.6	0.05	0.04	0.77	0.34	-0.54	-0.84	-0.84	-1.21	0.83	0.39	-3.9

Legend

Tissue index	Tissue name	Tissue/Sub-tissue name
T1	Adipose0	Adipose-Subcutaneous
T2	Adipose1	Adipose-Visceral (Omentum)
T3	Artery0	Artery-Aorta
T4	Artery1	Artery-Coronary
T5	Artery2	Artery – Tibial
T6	Brain0	Spinal cord (cervical c-1), Hypothalamus, Hippocampus, Frontal cortex (BA9), Cortex, Anterior cingulate cortex (BA24), Amygdala
T7	Brain1	Cerebellum, Cerebellar Hemisphere
T8	Brain2	Putamen (basal ganglia), Nucleus Accumbens (basal ganglia), Caudate (basal ganglia)
T9	Breast-Mammary Tissue	Breast - Mammary Tissue
T10	Colon-Sigmoid	Colon-Sigmoid
T11	Esophagus0	Esophagus - Gastroesophageal Junction
T12	Esophagus1	Esophagus – Mucosa
T13	Esophagus2	Esophagus – Muscularis
T14	Heart0	Heart - Atrial Appendage
T15	Heart1	Heart - Left Ventricle
T16	Liver	Liver
T17	Lung	Lung
T18	Muscle - Skeletal	Muscle – Skeletal
T19	Nerve - Tibial	Nerve – Tibial
T20	Ovary	Ovary
T21	Pituitary	Pituitary
T22	Prostate	Prostate
T23	Skin0	Skin - Not Sun Exposed (Suprapubic)
T24	Skin1	Skin-Sun Exposed (Lower leg)
T25	Testis	Testis
T26	Thyroid	Thyroid
T27	Uterus	Uterus
T28	Vagina	Vagina
T29	Whole Blood	Whole Blood

Process index	Process GO name	GO term accession
P1	immune system development	GO:0002520
P2	regulation of cardiac muscle contraction by regulation of the release of sequestered calcium ion	GO:0010881
P3	embryonic forelimb morphogenesis	GO:0035115
P4	calcium-mediated signaling using extracellular calcium source	GO:0035585
P5	camera-type eye development	GO:0043010
P6	positive regulation of adenylate cyclase activity	GO:0045762
P7	regulation of insulin secretion	GO:0050796
P8	calcium ion transport into cytosol	GO:0060402
P9	cardiac conduction	GO:0061337
P10	calcium ion transmembrane transport via high voltage-gated calcium channel	GO:0061577
P11	calcium ion import	GO:0070509
P12	cardiac muscle cell action potential involved in contraction	GO:0086002
P13	membrane depolarization during cardiac muscle cell action potential	GO:0086012
P14	membrane depolarization during AV node cell action potential	GO:0086045
P15	cell communication by electrical coupling involved in cardiac conduction	GO:0086064
P16	regulation of heart rate by cardiac conduction	GO:0086091
P17	regulation of ventricular cardiac muscle cell action potential	GO:0098911
P18	membrane depolarization during atrial cardiac muscle cell action potential	GO:0098912

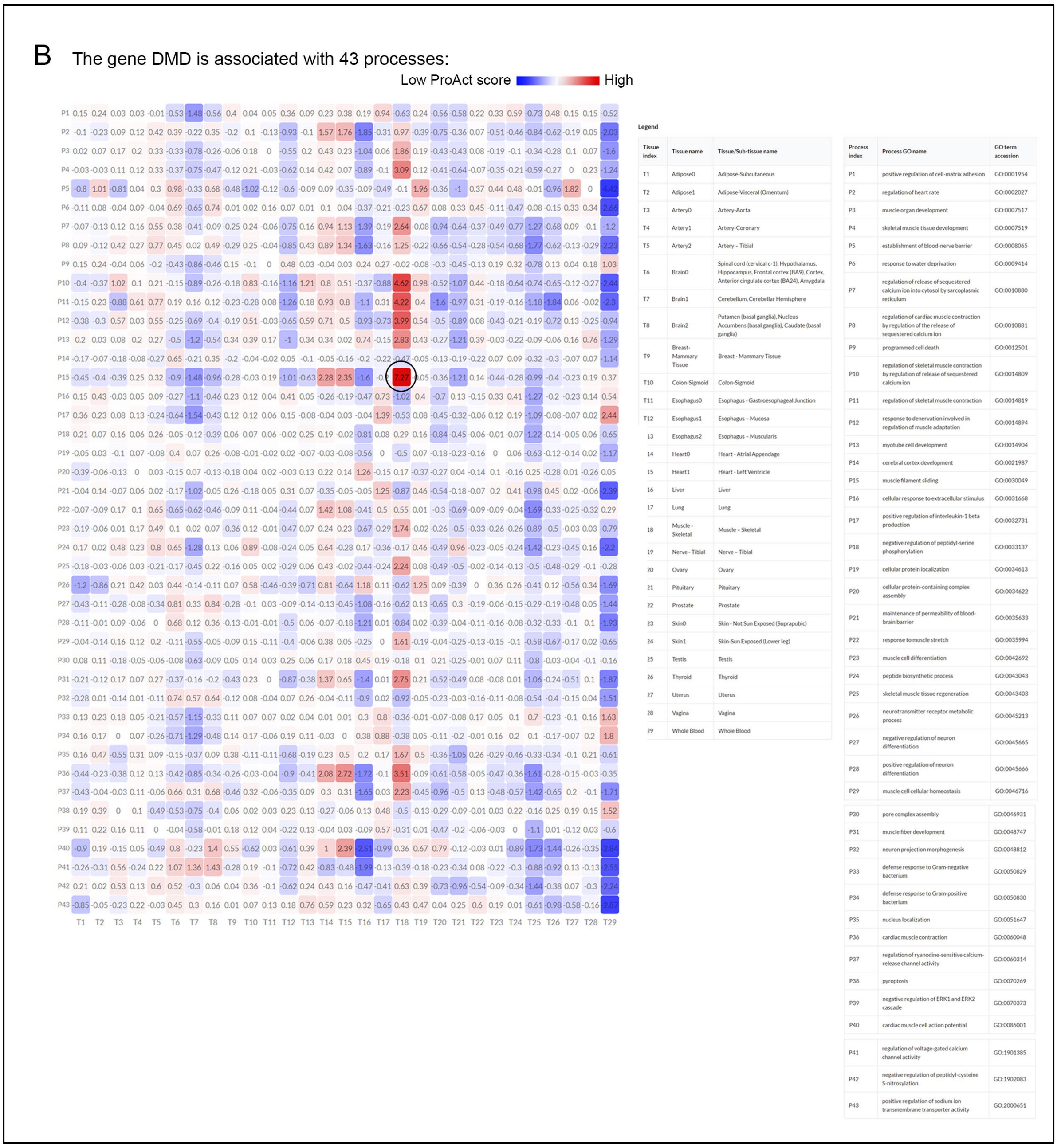

Appendix Fig. S3. TRACE predictions illuminate disease-related mechanisms.

A. The arrhythmia gene CACNA1C was annotated by GO to 18 processes. The heatmap shows the differential activity of these processes in 29 main tissues (red and blue denote high and low activity, respectively) (Sharon *et al*, 2022). The most highly active process was 'membrane depolarization during atrial cardiac muscle cell action potential' in heart left ventricle (circled), in accordance with arrhythmia phenotypes.

B. The Duchenne muscular dystrophy gene DMD was annotated by GO to 43 processes. The heatmap shows the differential activity of these processes in 29 main tissues (red and blue denote high and low activity, respectively) (Sharon *et al.*, 2022). The most highly active process was ‘muscle filament sliding' in skeletal muscle (circled), in accordance with Duchenne phenotypes and the impairment observed in mdx mouse model for Duchenne.

Figures were obtained from https://netbio.bgu.ac.il/ProAct/

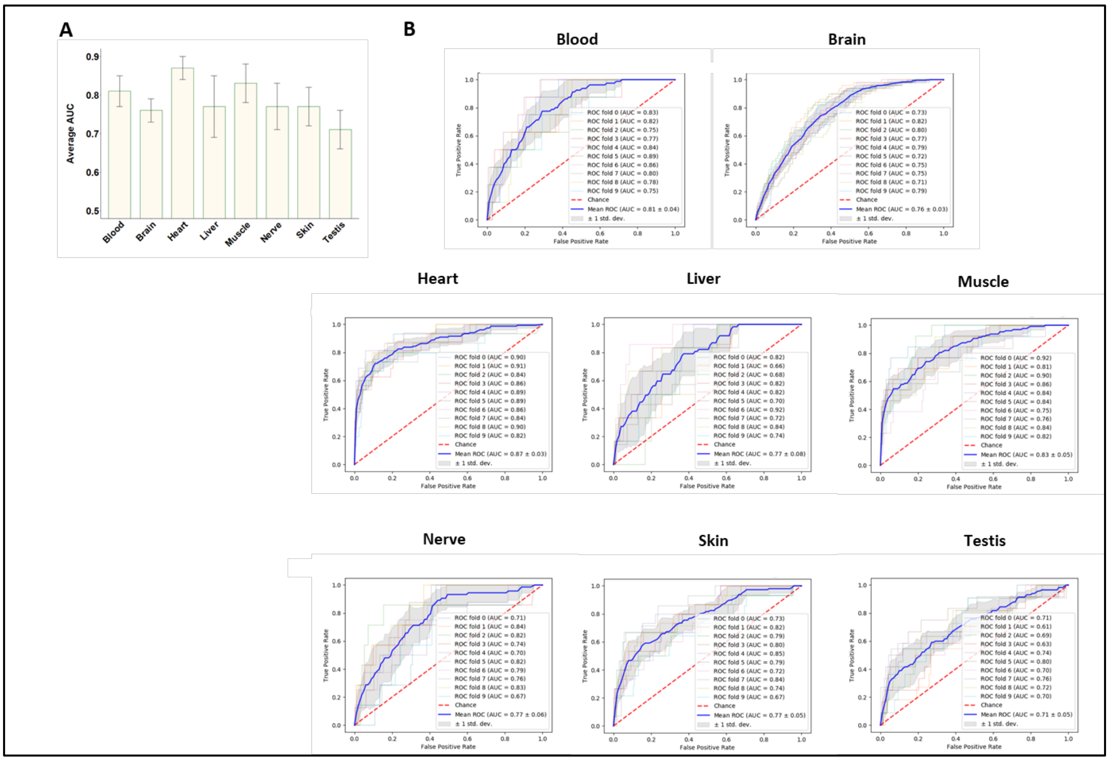

Appendix Fig. S4. Application of XGB to tissue models.

A. The average area under the receiver operating characteristic curve (AUC) obtained per tissue model following 10-fold cross-validation.

B. The receiver operating characteristic curves obtained per tissue model following 10-fold cross-validation.

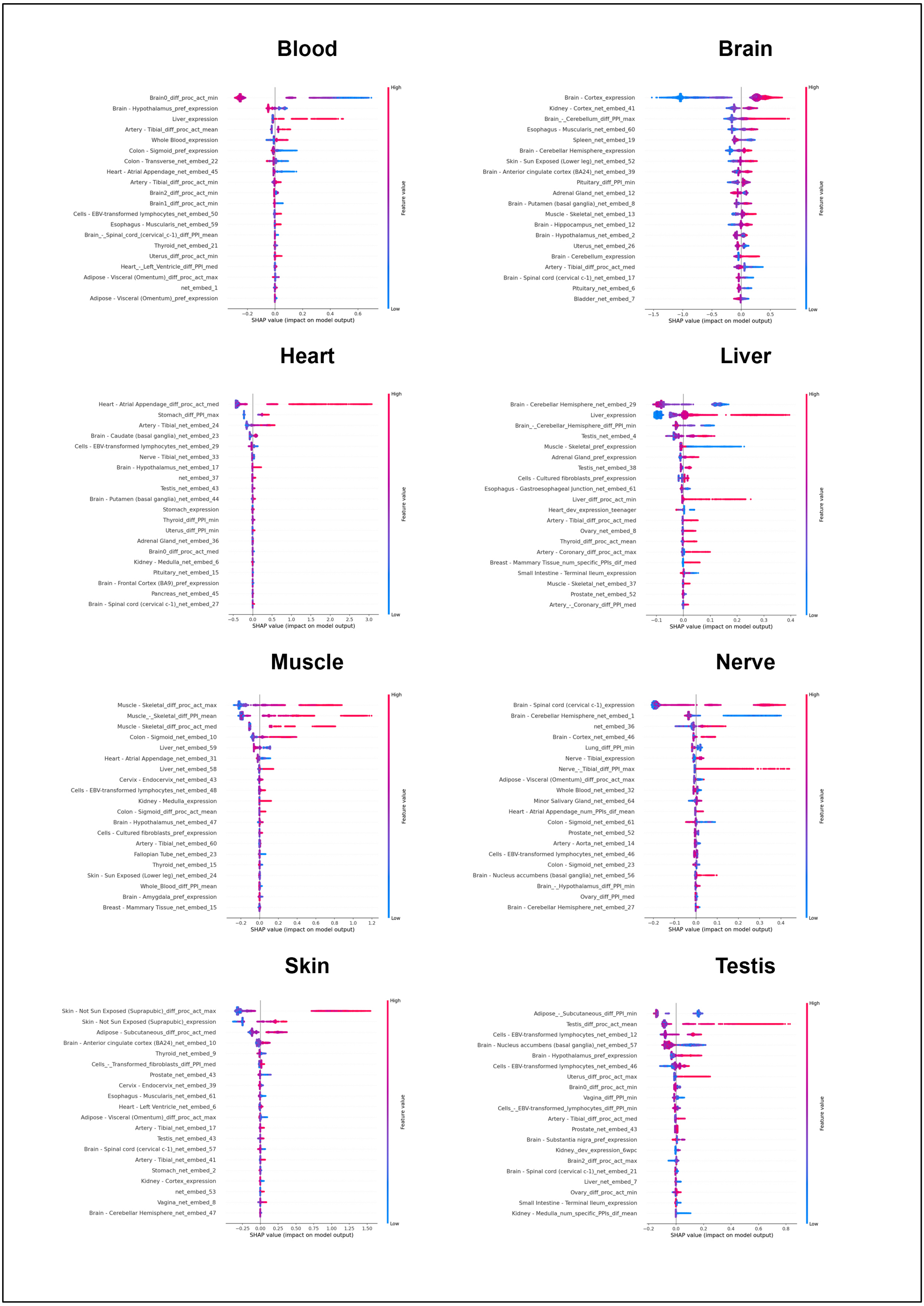
Blood
Brain0_diff_proc_act_min
Brain - Hypothalamus_pref_expression
Liver_expression
Artery - Tibial_diff_proc_act_mean
Whole Blood_expression
Colon - Sigmoid_pref_expression
Colon - Transverse_net_embed_22
Heart - Atrial Appendage_net_embed_45
Artery - Tibial_diff_proc_act_min
Brain2_diff_proc_act_min
Brain1_diff_proc_act_min
Cells - EBV-transformed lymphocytes_net_embed_50
Esophagus - Muscularis_net_embed_59
Brain_-_Spinal_cord_(cervical_c-1)_diff_PPI_mean
Thyroid_net_embed_21
Uterus_diff_proc_act_min
Heart_-_Left_Ventricle_diff_PPI_med
Adipose - Visceral (Omentum)_diff_proc_act_max
net_embed_1
Adipose - Visceral (Omentum)_pref_expression
SHAP value (impact on model output)
Feature value
High
Low
Brain
Brain - Cortex_expression
Kidney - Cortex_net_embed_41
Brain_-_Cerebellum_diff_PPI_max
Esophagus - Muscularis_net_embed_60
Spleen_net_embed_19
Brain - Cerebellar Hemisphere_expression
Skin - Sun Exposed (Lower leg)_net_embed_52
Brain - Anterior cingulate cortex (BA24)_net_embed_39
Pituitary_diff_PPI_min
Adrenal Gland_net_embed_12
Brain - Putamen (basal ganglia)_net_embed_8
Muscle - Skeletal_net_embed_13
Brain - Hippocampus_net_embed_12
Brain - Hypothalamus_net_embed_2
Uterus_net_embed_26
Brain - Cerebellum_expression
Artery - Tibial_diff_proc_act_med
Brain - Spinal cord (cervical c-1)_net_embed_17
Pituitary_net_embed_6
Bladder_net_embed_7
SHAP value (impact on model output)
Feature value
High
Low
Heart
Heart - Atrial Appendage_diff_proc_act_med
Stomach_diff_PPI_max
Artery - Tibial_net_embed_24
Brain - Caudate (basal ganglia)_net_embed_23
Cells - EBV-transformed lymphocytes_net_embed_29
Nerve - Tibial_net_embed_33
Brain - Hypothalamus_net_embed_17
net_embed_37
Testis_net_embed_43
Brain - Putamen (basal ganglia)_net_embed_44
Stomach_expression
Thyroid_diff_PPI_min
Uterus_diff_PPI_min
Adrenal Gland_net_embed_36
Brain0_diff_proc_act_med
Kidney - Medulla_net_embed_6
Pituitary_net_embed_15
Brain - Frontal Cortex (BA9)_pref_expression
Pancreas_net_embed_45
Brain - Spinal cord (cervical c-1)_net_embed_27
SHAP value (impact on model output)
Feature value
High
Low
Liver
Brain - Cerebellar Hemisphere_net_embed_29
Liver_expression
Brain_-_Cerebellar_Hemisphere_diff_PPI_min
Testis_net_embed_4
Muscle - Skeletal_pref_expression
Adrenal Gland_pref_expression
Testis_net_embed_38
Cells - Cultured fibroblasts_pref_expression
Esophagus - Gastroesophageal Junction_net_embed_61
Liver_diff_proc_act_min
Heart_dev_expression_teenager
Artery - Tibial_diff_proc_act_med
Ovary_net_embed_8
Thyroid_diff_proc_act_mean
Artery - Coronary_diff_proc_act_max
Breast - Mammary Tissue_num_specific_PPIs_dif_med
Small Intestine - Terminal Ileum_expression
Muscle - Skeletal_net_embed_37
Prostate_net_embed_52
Artery_-_Coronary_diff_PPI_med
SHAP value (impact on model output)
Feature value
High
Low
Muscle
Muscle - Skeletal_diff_proc_act_max
Muscle_-_Skeletal_diff_PPI_mean
Muscle - Skeletal_diff_proc_act_med
Colon - Sigmoid_net_embed_10
Liver_net_embed_59
Heart - Atrial Appendage_net_embed_31
Liver_net_embed_58
Cervix - Endocervix_net_embed_43
Cells - EBV-transformed lymphocytes_net_embed_48
Kidney - Medulla_expression
Colon - Sigmoid_diff_proc_act_mean
Brain - Hypothalamus_net_embed_47
Cells - Cultured fibroblasts_pref_expression
Artery - Tibial_net_embed_60
Fallopian Tube_net_embed_23
Thyroid_net_embed_15
Skin - Sun Exposed (Lower leg)_net_embed_24
Whole_Blood_diff_PPI_mean
Brain - Amygdala_pref_expression
Breast - Mammary Tissue_net_embed_15
SHAP value (impact on model output)
Feature value
High
Low
Nerve
Brain - Spinal cord (cervical c-1)_expression
Brain - Cerebellar Hemisphere_net_embed_1
net_embed_36
Brain - Cortex_net_embed_46
Lung_diff_PPI_min
Nerve - Tibial_expression
Nerve_-_Tibial_diff_PPI_max
Adipose - Visceral (Omentum)_diff_proc_act_max
Whole Blood_net_embed_32
Minor Salivary Gland_net_embed_64
Heart - Atrial Appendage_num_PPIs_dif_mean
Colon - Sigmoid_net_embed_61
Prostate_net_embed_52
Artery - Aorta_net_embed_14
Cells - EBV-transformed lymphocytes_net_embed_46
Colon - Sigmoid_net_embed_23
Brain - Nucleus accumbens (basal ganglia)_net_embed_56
Brain_-_Hypothalamus_diff_PPI_min
Ovary_diff_PPI_med
Brain - Cerebellar Hemisphere_net_embed_27
SHAP value (impact on model output)
Feature value
High
Low
Skin
Skin - Not Sun Exposed (Suprapubic)_diff_proc_act_max
Skin - Not Sun Exposed (Suprapubic)_expression
Adipose - Subcutaneous_diff_proc_act_med
Brain - Anterior cingulate cortex (BA24)_net_embed_10
Thyroid_net_embed_9
Cells_-_Transformed_fibroblasts_diff_PPI_med
Prostate_net_embed_43
Cervix - Endocervix_net_embed_39
Esophagus - Muscularis_net_embed_61
Heart - Left Ventricle_net_embed_6
Adipose - Visceral (Omentum)_diff_proc_act_max
Artery - Tibial_net_embed_17
Testis_net_embed_43
Brain - Spinal cord (cervical c-1)_net_embed_57
Artery - Tibial_net_embed_41
Stomach_net_embed_2
Kidney - Cortex_expression
net_embed_53
Vagina_net_embed_8
Brain - Cerebellar Hemisphere_net_embed_47
SHAP value (impact on model output)
Feature value
High
Low
Testis
Adipose_-_Subcutaneous_diff_PPI_min
Testis_diff_proc_act_mean
Cells - EBV-transformed lymphocytes_net_embed_12
Brain - Nucleus accumbens (basal ganglia)_net_embed_57
Brain - Hypothalamus_pref_expression
Cells - EBV-transformed lymphocytes_net_embed_46
Uterus_diff_proc_act_max
Brain0_diff_proc_act_min
Vagina_diff_PPI_min
Cells_-_EBV-transformed_lymphocytes_diff_PPI_min
Artery - Tibial_diff_proc_act_med
Prostate_net_embed_43
Brain - Substantia nigra_pref_expression
Kidney_dev_expression_6wpc
Brain2_diff_proc_act_max
Brain - Spinal cord (cervical c-1)_net_embed_21
Liver_net_embed_7
Ovary_diff_proc_act_min
Small Intestine - Terminal Ileum_expression
Kidney - Medulla_num_specific_PPIs_dif_mean
SHAP value (impact on model output)
Feature value
High
Low

Appendix Fig. S5. SHAP assessment of the contribution of features to tissue models based on XGB. The 20 topmost contributing features to each tissue model. Features were ordered from bottom to top by their increased absolute contribution to the model, allowing interpretation. Per feature, each dot represents the feature value of a different gene; red and blue denote high and low values of the feature, respectively. Dots were spread from left to right by their contribution to prediction of the gene as leading to a disease that manifests in the modeled tissue (left) or not (left). Diff_net = differential network; diff_proc_act = differential process activity.

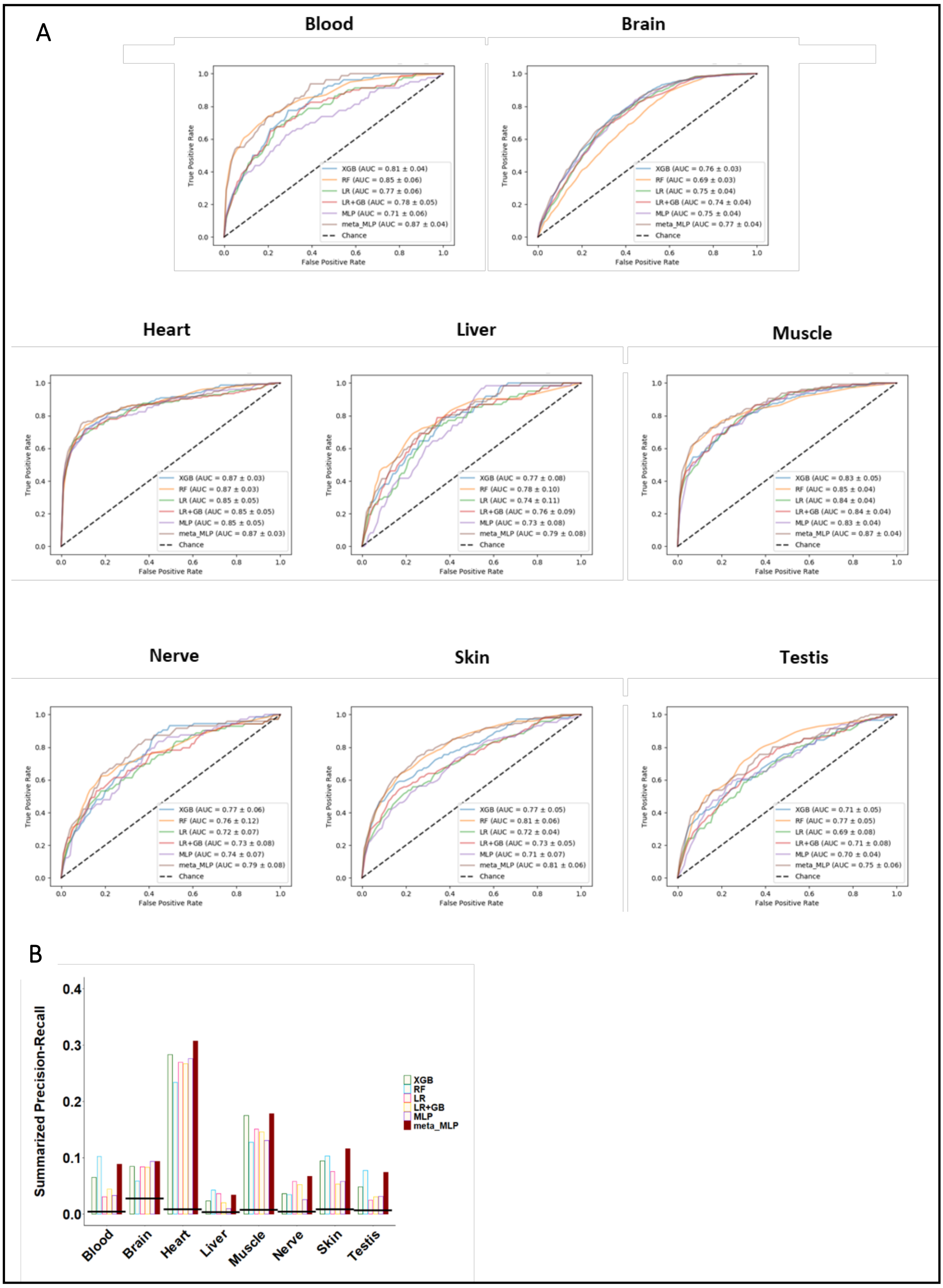

Appendix Fig. S6. The performance of the different classifiers in tissue models.

A. average ROC curves obtained per ML method and tissue model following 10-fold cross-validation.

B. The summarized precision recall scores obtained per ML method and tissue model following 10-fold cross-validation. Black horizontal lines depict the random prediction baseline, calculated as positive-to-negative ratio.

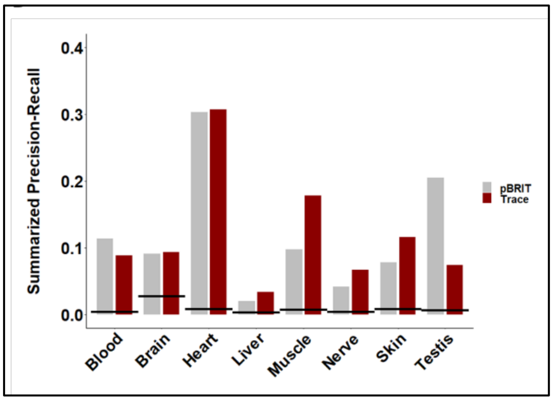

Appendix Fig. S7. The summarized precision recall scores obtained by pBRIT and TRACE per tissue model. Performance was assessed via 10-fold cross-validation. The exact same folds were used. The scores of TRACE were higher in 6/8 tissue models. Black horizontal lines depict the random prediction baseline, calculated as positive-to-negative ratio.

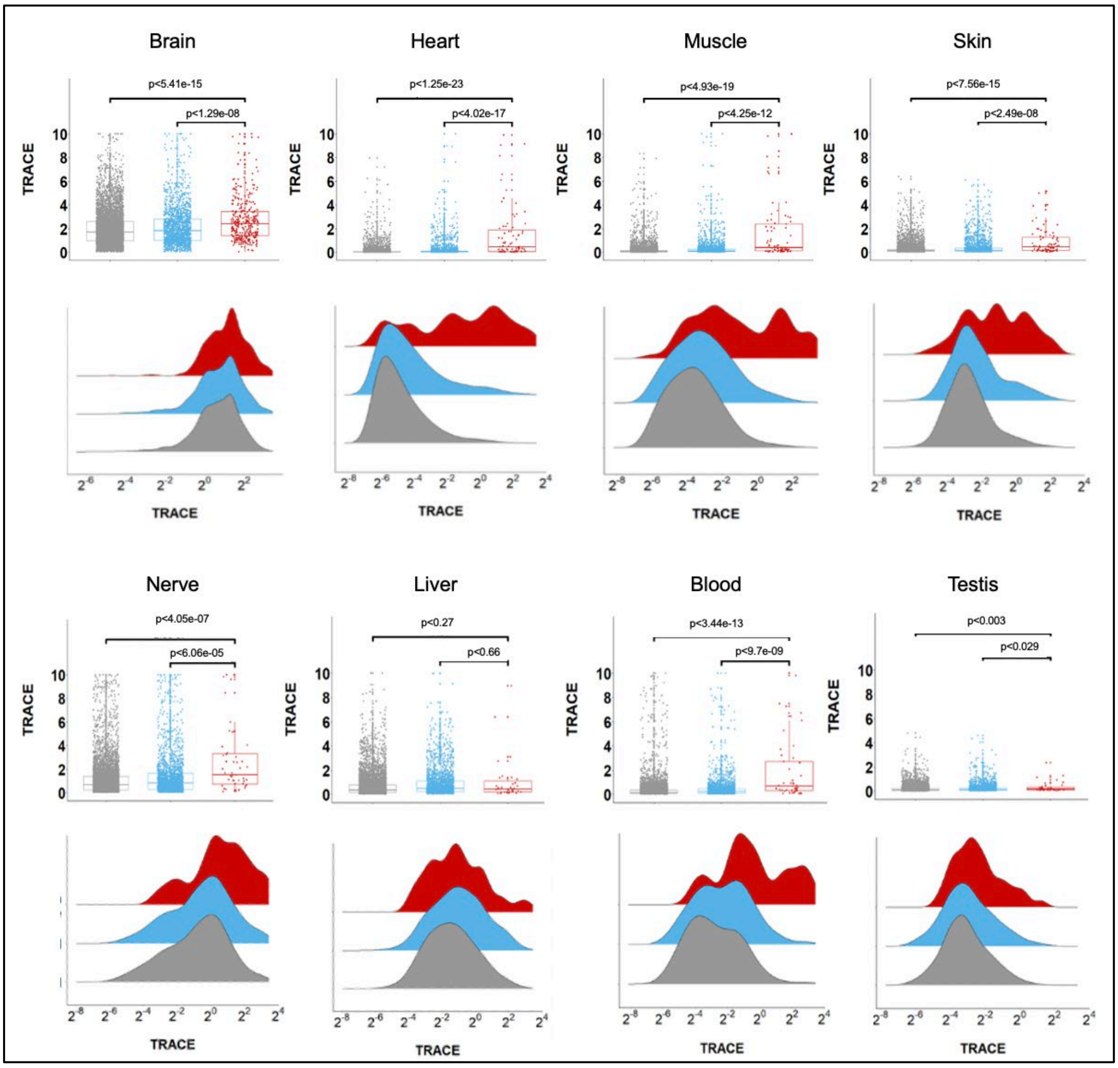

Appendix Fig. S8. TRACE prioritization of non-tissue-specific genes associated with diseases that manifest in distinct tissues. This analysis included 8,749 genes that were expressed in at least 80% of the tissues. The figure shows gene TRACE scores in the different tissue models as dot and density ridge plots. Each dot in a dot plot represents the TRACE score of a different gene. Genes were divided into genes that are causal for a disease that manifests in the modeled tissue (tissue-associated, red), genes that are causal for a disease that does not manifest in the modeled tissue (other disease genes, blue), and non-disease genes (grey). Tissue-associated disease genes had significantly higher TRACE scores compared to non-disease genes and to other disease genes in all tissue models except for liver (Mann-Whitney U test, adjusted p-values are shown).

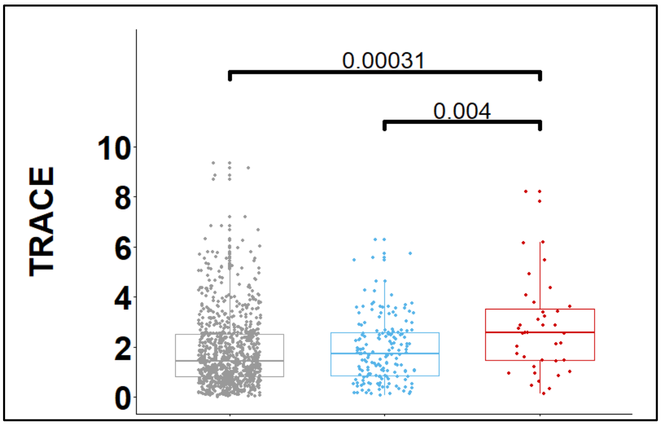

Appendix Fig. S9. TRACE prioritization of non-overexpressed genes. This analysis included 1,094 non-preferentially expressed genes (preferential expression <2 across all tissues), of which 208 were disease genes and 72 were associated with the tissues that we modeled. Results are shown for the brain tissue model, since brain was the only modeled tissue with ≥10 tissue-associated disease genes (specifically 38). Genes were divided into genes that are causal for a disease that manifests in brain (red), genes that are causal for a disease that does not manifest in brain (blue), and non-disease genes (grey). Brain-associated disease genes had significantly higher TRACE scores compared to non-disease genes and to other disease genes (Mann-Whitney U test).

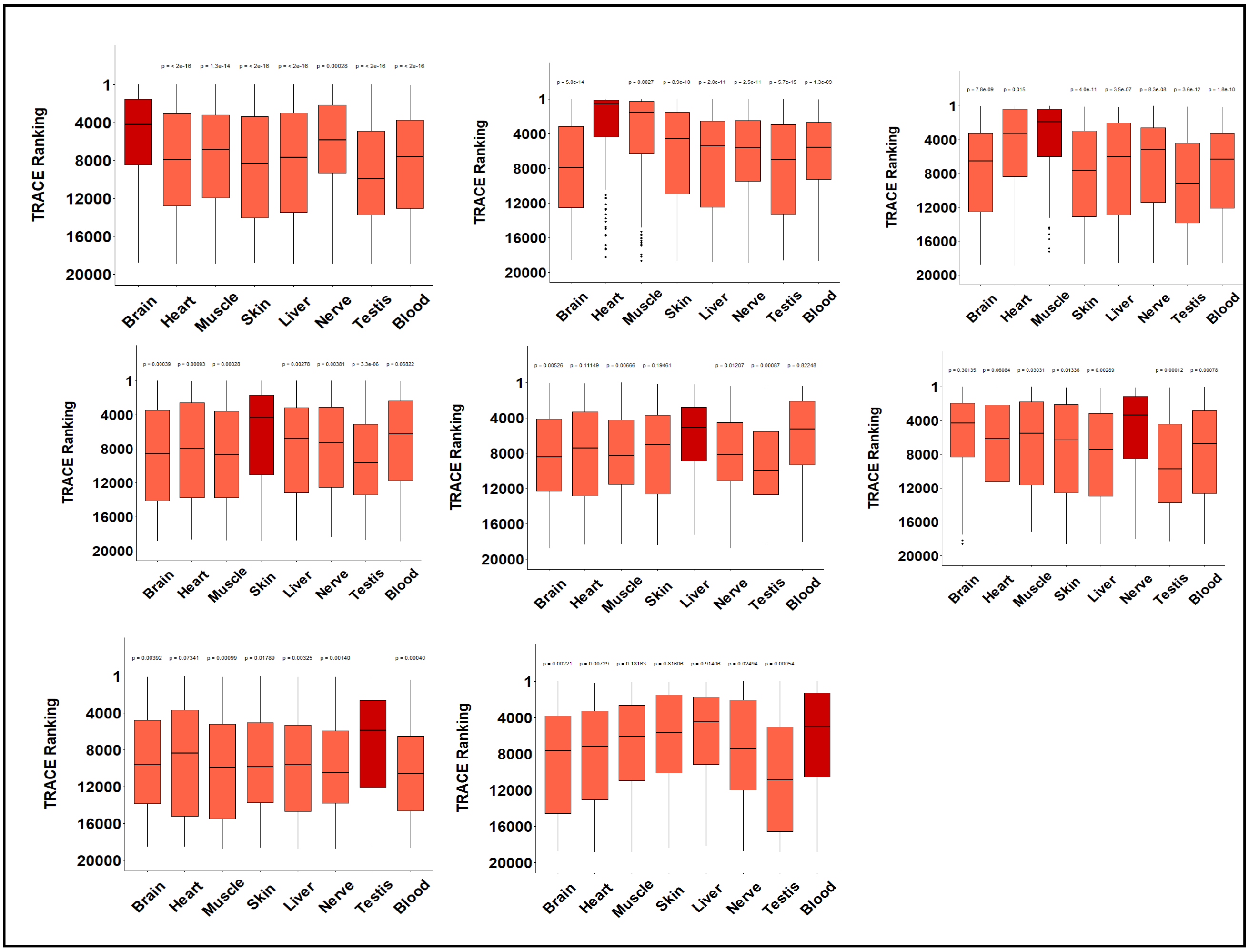

Appendix Fig. S10. TRACE rankings of tissue-associated disease genes in each tissue model. Boxplots in each panel show the ranks of the same set of tissue-associated disease genes per tissue model; the red boxplot annotates the correct affected tissue. For example, the upper left panel shows the ranks of brain-associated disease genes in each tissue model. The median rank in the model of the correct affected tissue (e.g., brain) was higher than the median rank in other tissue models, except for blood-associated genes whose median was higher in liver (p-values appearing above each boxplot were calculated using paired Wilcoxon tests).

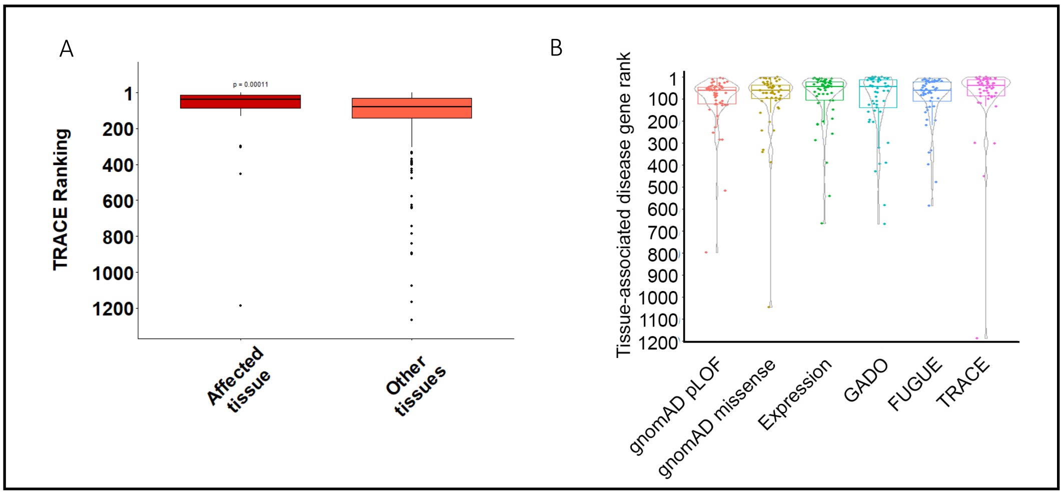

Appendix Fig. S11. TRACE ranking of verified disease genes.

A. TRACE rankings of verified disease genes of patients in the model of the patients' correct tissue (affected tissue, red boxplot) and in other models. The ranks in the model of the patients' correct tissue were significantly higher (p=0.00011, MW).

B. Comparison between the rank of the verified disease genes out of the patient's candidate genes between TRACE and other methods. Median rank of TRACE: 39; gnomAD (Karczewski *et al*, 2020) pLoF: 61 and missense: 60; expression-based prioritization: 43; GADO (Deelen *et al*, 2019): 45; FUGUE (Somepalli *et al*, 2021): 60. TRACE prioritization was better than prioritization by other methods (adjusted p=0.00155, 0.01, 0.01, 0.01, 0.01, respectively, Wilcoxon signed-rank test).

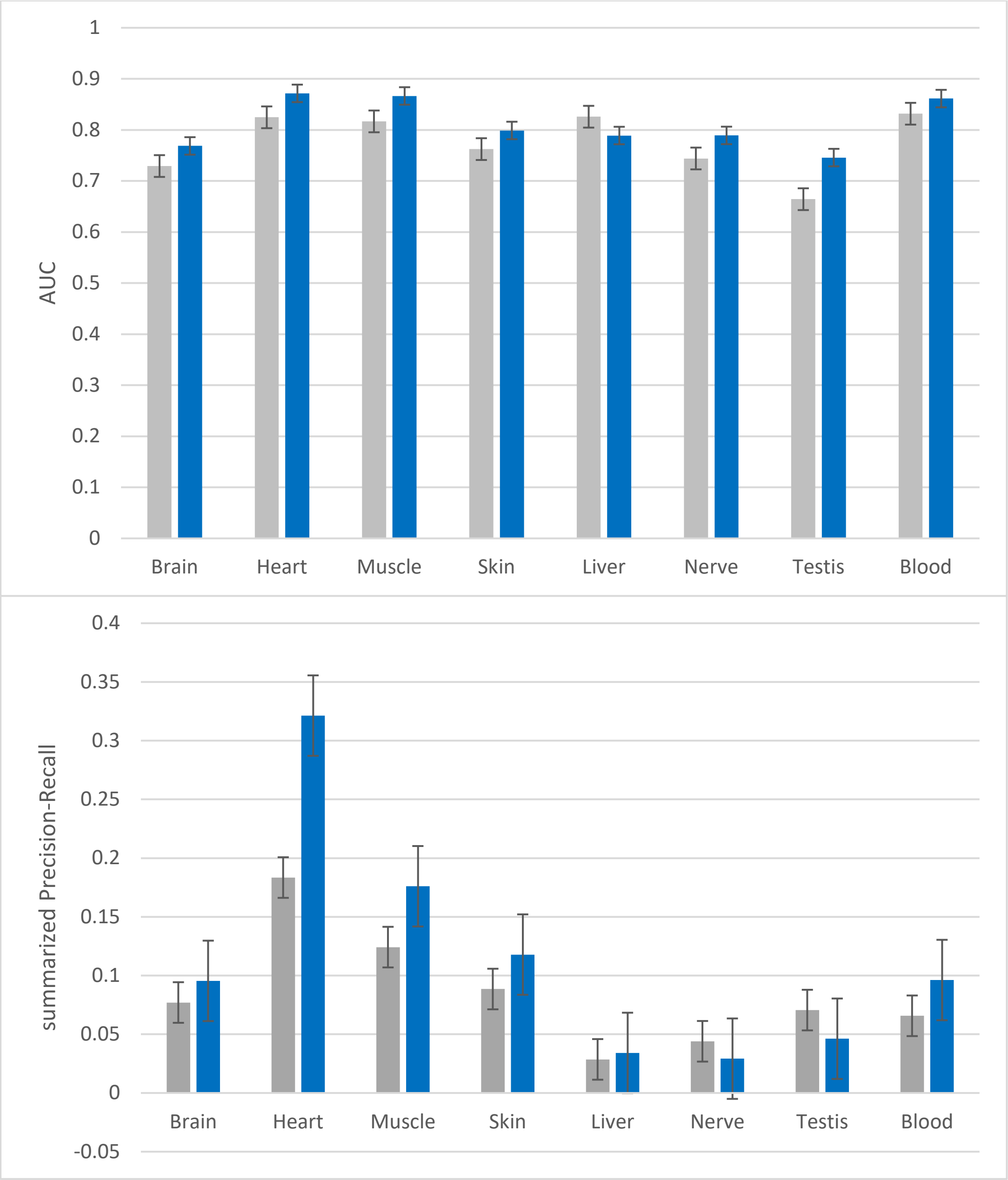

Appendix Fig. S12. The performance of TRACE models that used a partial features' dataset (grey) and models that used the full features dataset (blue). The partial dataset included 594 features that were derived from transcriptomics and PPIs were used; network embedding features were excluded.

A. The area under the receiver-operating characteristic curve (AUC) by each model. TRACE models based on the full dataset performed better than models that used the limited dataset in 7/8 cases (Wilcoxon signed-rank test p=0.039).

B. The summarized precision-recall. TRACE models based on the full dataset performed better than models that used the limited dataset in 6/8 cases (Wilcoxon signed-rank test p=0.11).

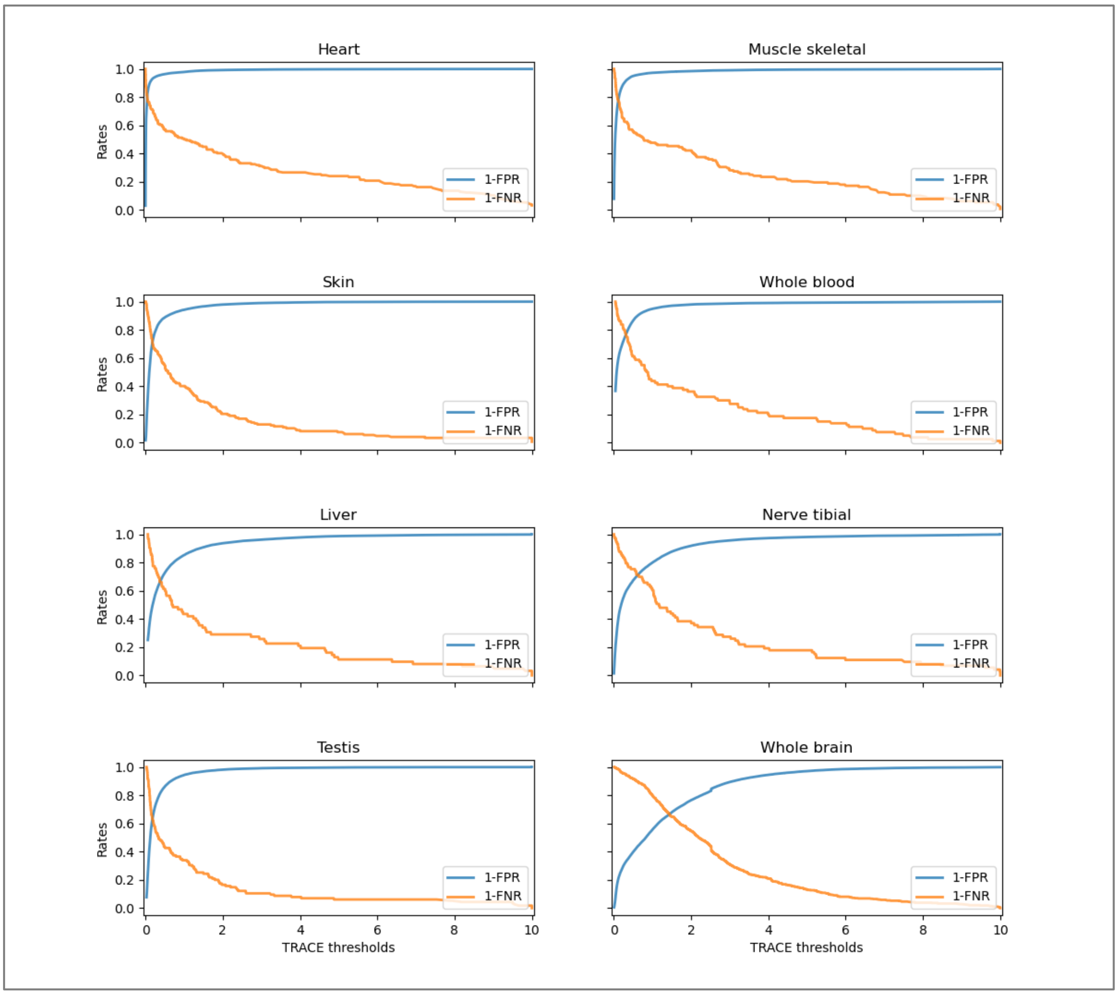

Appendix Fig. S13. Assessment of TRACE per model. The true positive rate versus true negative rate of each tissue model (FPR = false positive rate; FNR = false negative rate).

REFERENCES

Deelen P, van Dam S, Herkert JC, Karjalainen JM, Brugge H, Abbott KM, van Diemen CC, van der Zwaag PA, Gerkes EH, Zonneveld-Huijssoon E *et al* (2019) Improving the diagnostic yield of exome- sequencing by predicting gene-phenotype associations using large-scale gene expression analysis. *Nat Commun* 10: 2837

Karczewski KJ, Francioli LC, Tiao G, Cummings BB, Alföldi J, Wang Q, Collins RL, Laricchia KM, Ganna A, Birnbaum DP *et al* (2020) The mutational constraint spectrum quantified from variation in 141,456 humans. *Nature* 581: 434-443

Sharon M, Vinogradov E, Argov CM, Lazarescu O, Zoabi Y, Hekselman I, Yeger-Lotem E (2022) The differential activity of biological processes in tissues and cell subsets can illuminate disease-related processes and cell type identities. *Bioinformatics*

Somepalli G, Sahoo S, Singh A, Hannenhalli S (2021) Prioritizing and characterizing functionally relevant genes across human tissues. *PLoS Comput Biol* 17: e1009194

Table S1. The tissue-aware gene features used in the analyses.

Feature type	Dataset type	Feature description per gene *g*	Feature name (*t* denotes tissue name)	Available for # of tissues	Total # of features
**Tissue expression**	Transcriptomics	Median expression level of *g* in tissue or organ *t*[1]	*t_expression*	54 adult tissues	54
		Median expression in developmental time points of *g* in organ *t*[2]	*t_dev_expression_#* (*# ranges from 1 to 20 according to developmental time point in organ t)*	7 organs	134
**Tissue preferential expression**	Transcriptomics	Preferential expression (overexpression) of *g* in tissue *t* relative to other tissues	*t_pref_expression*	54 adult tissues	54
**Tissue eQTL (eQTL= expression quantitative trait loci)**	eQTL	An indicator of whether *g* is involved in at least one eQTL in tissue *t* (based on data from GTEx portal)	*t_eQTL*	48 adult tissues	48
**Tissue PPIs (PPIs=protein-protein interactions)** **A. Tissue interactors**	Transcriptomics + PPIs	Number of PPIs of *g*'s encoded protein in tissue *t*	*t_num_PPIs*	54 adult tissues	54
		Difference between number of PPIs of *g*'s encoded protein in tissue *t* and the median number of its PPIs across tissues	*t_num_PPIs_dif_med*	54 adult tissues	54
		Difference between number of PPIs of *g*'s encoded protein in tissue *t* and the mean number of its PPIs across tissues	*t_num_PPIs_dif_mean*	54 adult tissues	54
**Tissue PPIs** **B. Tissue preferential interactors**	Transcriptomics + PPIs	Number of PPIs of *g*'s encoded protein that involved preferentially expressed interactors in tissue *t*	*t_num_pref_PPIs*	54 adult tissues	54

		Difference between number of preferential PPIs of *g’*s encoded protein in tissue *t* and the median number of its preferential PPIs across tissues	*t_num_pref_PPIs_dif_med*	54 adult tissues	54
		Difference between number of preferential PPIs of *g’*s encoded protein in tissue *t* and the mean number of its preferential PPIs across tissues	*t_num_pref_PPIs_dif_mean*	54 adult tissues	54
**Tissue PPIs**<br><br>**C. Tissue-specific interactors**	Transcriptomics + PPIs	Number of PPIs of *g’*s encoded protein in tissue *t* that were present in at most 20% of the tissues	*t_num_specific_PPIs*	54 adult tissues	54
		Difference between number of tissue specific PPIs of *g’*s encoded protein in tissue *t* and the median number of its tissue specific PPIs across tissues	*t_num_specific_PPIs_dif_med*	54 adult tissues	54
		Difference between number of tissue specific PPIs of *g’*s encoded protein in tissue *t* and the mean number of its tissue specific PPIs across tissues	*t_num_specific_PPIs_dif_mean*	54 adult tissues	54
**Tissue differential PPIs[3]**	Transcriptomics + PPIs	The minimum differential PPI score of *g’*s encoded protein in tissue *t*	*t_diff_PPI_min*	44 adult tissues	44
		The maximum differential PPI score of *g’*s encoded	*t_diff_PPI_max*	44 adult tissues	44

		protein in tissue *t*			
		The median differential PPI score of *g*'s encoded protein in tissue *t*	*t_diff_PPI_med*	44 adult tissues	44
		The mean differential PPI score of *g*'s encoded protein in tissue *t*	*t_diff_PPI_mean*	44 adult tissues	44
**Tissue network embedding**	Transcriptomics + PPIs	64 vectors representing the interactome neighborhood of *g*'s encoded protein in the PPI interactome of tissue *t*	*t_net_embed_#* *(# ranges between 1 and 64)*	54 adult tissues +generic interactome	3,520
**Tissue expression variability**	Transcriptomics	The variation in expression level of *g* across samples of tissue *t*[4]	*t_expression_var*	19 adult tissues	19
		The variation in the median expression level of *g* across developmental time points of organ *t*[2]	*t_dev_expression_var*	7 organs	7
**Tissue expression relationship between paralogous genes (ref barshir)**	Transcriptomics + homology	Ratio between the expression levels of *g* and its paralog with highest sequence identity in tissue *t*	*t_paralog_ratio_single*	53 adult tissues	53
		Ratio between the expression levels of *g* and the median expression level of all its paralogs in tissue *t*	*t_paralogs_ratio_all*	53 adult tissues	53
**Differential process activity**	Transcriptomics + gene ontology[5] (GO)	The number of GO biological process terms associated with *g* (denoted *g*'s GO terms)	*t_num_processes*	28 adult tissues	28
		Minimum differential activity score of *g*'s GO terms in tissue *t*	*t_diff_proc_act_min*	28 adult tissues	28
		Maximum differential activity score of	*t_diff_proc_act_max*	28 adult tissues	28

		g's GO terms in tissue *t*			
		Median differential activity score of *g*'s GO terms in tissue *t*	*t_diff_proc_act_med*	28 adult tissues	28
		Mean differential activity score of *g*'s GO terms in tissue *t*	*t_diff_proc_act_mean*	28 adult tissues	28

REFERENCES

1 Aguet, F. et al. The GTEx Consortium atlas of genetic regulatory effects across human tissues. 787903, doi:10.1101/787903 %J bioRxiv (2019).

2 Cardoso-Moreira, M. et al. Gene expression across mammalian organ development. Nature 571, 505-509, doi:10.1038/s41586-019-1338-5 (2019).

3 Basha, O., Shpringer, R., Argov, C. M. & Yeger-Lotem, E. The DifferentialNet database of differential protein-protein interactions in human tissues. Nucleic Acids Res 46, D522-D526, doi:10.1093/nar/gkx981 (2018).

4 Simonovsky, E., Schuster, R. & Yeger-Lotem, E. Large-scale analysis of human gene expression variability associates highly variable drug targets with lower drug effectiveness and safety. Bioinformatics 35, 3028-3037, doi:10.1093/bioinformatics/btz023 (2019).

5 The Gene Ontology, C. The Gene Ontology Resource: 20 years and still GOing strong. Nucleic Acids Res 47, D330-D338, doi:10.1093/nar/gky1055 (2019).

Membership of the GTEx Consortium

Laboratory and Data Analysis Coordinating Center (LDACC): François Aguet[1], Shankara Anand[1], Kristin G Ardlie[1], Stacey Gabriel[1], Gad Getz[1,30,31], Aaron Graubert[1], Kane Hadley[1], Robert E Handsaker[33,34,35], Katherine H Huang[1], Seva Kashin[33,34,35], Xiao Li[1], Daniel G MacArthur[34,36], Samuel R Meier[1], Jared L Nedzel[1], Duyen T Nguyen[1], Ayellet V Segrè[1,17], Ellen Todres[1]

Analysis Working Group (funded by GTEx project grants):
François Aguet[1], Shankara Anand[1], Kristin G Ardlie[1], Brunilda Balliu[41], Alvaro N Barbeira[2], Alexis Battle[18,11], Rodrigo Bonazzola[2], Andrew Brown[3,4], Christopher D Brown[24], Stephane E Castel[5,6], Donald F Conrad[42,43], Daniel J Cotter[29], Nancy Cox[16], Sayantan Das[26], Olivia M de Goede[29], Emmanouil T Dermitzakis[3,27,28], Jonah Einson[44,5], Barbara E Engelhardt[7,8], Eleazar Eskin[45], Tiffany Y Eulalio[46], Nicole M Ferraro[46], Elise D Flynn[5,6], Laure Fresard[12], Eric R Gamazon[13,14,15,16], Diego Garrido-Martín[22], Nicole R Gay[29], Gad A Getz[1,30,31], Michael J Gloudemans[46], Aaron Graubert[1], Roderic Guigó[22,32], Kane Hadley[1], Andrew R Hamel[17,1], Robert E Handsaker[33,34,35], Yuan He[18], Paul J Hoffman[5], Farhad Hormozdiari[19,1], Lei Hou[47,1], Katherine H Huang[1], Hae Kyung Im[2], Brian Jo[7,8], Silva Kasela[5,6], Seva Kashin[33,34,35], Manolis Kellis[47,1], Sarah Kim-Hellmuth[5,6,9], Alan Kwong[26], Tuuli Lappalainen[5,6], Xiao Li[1], Xin Li[12], Yanyu Liang[2], Daniel G MacArthur[34,36], Serghei Mangul[45,48], Samuel R Meier[1], Pejman Mohammadi[5,6,20,21], Stephen B Montgomery[12,29], Manuel Muñoz-Aguirre[22,23], Daniel C Nachun[12], Jared L Nedzel[1], Duyen T Nguyen[1], Andrew B Nobel[49], Meritxell Oliva[2,10], YoSon Park[24,25], Yongjin Park[47,1], Princy Parsana[11], Abhiram S Rao[50], Ferran Reverter[51], John M Rouhana[17,1], Chiara Sabatti[52], Ashis Saha[11], Ayellet V Segrè[1,17], Andrew D Skol[2,53], Matthew Stephens[37], Barbara E Stranger[2,38], Benjamin J Strober[18], Nicole A Teran[12], Ellen Todres[1], Ana Viñuela[39,3,27,28], Gao Wang[37], Xiaoquan Wen[26], Fred Wright[54], Valentin Wucher[22], Yuxin Zou[40]

Analysis Working Group (not funded by GTEx project grants): Pedro G Ferreira[55,56,57,58], Gen Li[59], Marta Melé[60], Esti Yeger-Lotem[61,62]

Leidos Biomedical - Project Management: Mary E Barcus[63], Debra Bradbury[63], Tanya Krubit[63], Jeffrey A McLean[63], Liqun Qi[63], Karna Robinson[63], Nancy V Roche[63], Anna M Smith[63], Leslie Sobin[63], David E Tabor[63], Anita Undale[63]

Biospecimen collection source sites: Jason Bridge[64], Lori E Brigham[65], Barbara A Foster[66], Bryan M Gillard[66], Richard Hasz[67], Marcus Hunter[68], Christopher Johns[69], Mark Johnson[70], Ellen Karasik[66], Gene Kopen[71], William F Leinweber[71], Alisa McDonald[71], Michael T Moser[66], Kevin Myer[68], Kimberley D Ramsey[66], Brian Roe[68], Saboor Shad[71], Jeffrey A Thomas[71,70], Gary Walters[70], Michael Washington[70], Joseph Wheeler[69]

Biospecimen core resource: Scott D Jewell[72], Daniel C Rohrer[72], Dana R Valley[72]

Brain bank repository: David A Davis[73], Deborah C Mash[73]

Pathology: Mary E Barcus[63], Philip A Branton[74], Leslie Sobin[63]

ELSI study: Laura K Barker[75], Heather M Gardiner[75], Maghboeba Mosavel[76], Laura A Siminoff[75]

Genome Browser Data Integration & Visualization: Paul Flicek[77], Maximilian Haeussler[78], Thomas Juettemann[77], W James Kent[78], Christopher M Lee[78], Conner C Powell[78], Kate R Rosenbloom[78], Magali Ruffier[77], Dan Sheppard[77], Kieron Taylor[77], Stephen J Trevanion[77], Daniel R Zerbino[77]

eGTEx groups: Nathan S Abell[29], Joshua Akey[79], Lin Chen[10], Kathryn Demanelis[10], Jennifer A Doherty[80], Andrew P Feinberg[81], Kasper D Hansen[82], Peter F Hickey[83], Lei Hou[47,1], Farzana Jasmine[10], Lihua Jiang[29], Rajinder Kaul[84,85], Manolis Kellis[47,1], Muhammad G Kibriya[10], Jin Billy Li[29], Qin Li[29], Shin Lin[86], Sandra E Linder[29], Stephen B Montgomery[12,29], Meritxell Oliva[2,10], Yongjin Park[47,1], Brandon L Pierce[10], Lindsay F Rizzardi[87], Andrew D Skol[2,53], Kevin S Smith[12], Michael Snyder[29], John Stamatoyannopoulos[84,88], Barbara E Stranger[2,38], Hua Tang[29], Meng Wang[29]

NIH program management: Philip A Branton[74], Latarsha J Carithers[74,89], Ping Guan[74], Susan E Koester[90], A. Roger Little[91], Helen M Moore[74], Concepcion R Nierras[92], Abhi K Rao[74], Jimmie B Vaught[74], Simona Volpi[93]

Affiliations

1. The Broad Institute of MIT and Harvard, Cambridge, MA, USA
2. Section of Genetic Medicine, Department of Medicine, The University of Chicago, Chicago, IL, USA
3. Department of Genetic Medicine and Development, University of Geneva Medical School, Geneva, Switzerland
4. Population Health and Genomics, University of Dundee, Dundee, Scotland, UK
5. New York Genome Center, New York, NY, USA
6. Department of Systems Biology, Columbia University, New York, NY, USA
7. Department of Computer Science, Princeton University, Princeton, NJ, USA
8. Center for Statistics and Machine Learning, Princeton University, Princeton, NJ, USA
9. Statistical Genetics, Max Planck Institute of Psychiatry, Munich, Germany
10. Department of Public Health Sciences, The University of Chicago, Chicago, IL, USA
11. Department of Computer Science, Johns Hopkins University, Baltimore, MD, USA
12. Department of Pathology, Stanford University, Stanford, CA, USA
13. Data Science Institute, Vanderbilt University, Nashville, TN, USA
14. Clare Hall, University of Cambridge, Cambridge, UK
15. MRC Epidemiology Unit, University of Cambridge, Cambridge, UK
16. Division of Genetic Medicine, Department of Medicine, Vanderbilt University Medical Center, Nashville, TN, USA
17. Ocular Genomics Institute, Massachusetts Eye and Ear, Harvard Medical School, Boston, MA, USA
18. Department of Biomedical Engineering, Johns Hopkins University, Baltimore, MD, USA
19. Department of Epidemiology, Harvard T.H. Chan School of Public Health, Boston, MA, USA
20. Scripps Research Translational Institute, La Jolla, CA, USA
21. Department of Integrative Structural and Computational Biology, The Scripps Research Institute, La Jolla, CA, USA
22. Centre for Genomic Regulation (CRG), The Barcelona Institute for Science and Technology, Barcelona, Catalonia, Spain
23. Department of Statistics and Operations Research, Universitat Politècnica de Catalunya (UPC), Barcelona, Catalonia, Spain
24. Department of Genetics, University of Pennsylvania, Perelman School of Medicine, Philadelphia, PA, USA
25. Department of Systems Pharmacology and Translational Therapeutics, University of Pennsylvania, Perelman School of Medicine, Philadelphia, PA, USA
26. Department of Biostatistics, University of Michigan, Ann Arbor, MI, USA
27. Institute for Genetics and Genomics in Geneva (iGE3), University of Geneva, Geneva, Switzerland
28. Swiss Institute of Bioinformatics, Geneva, Switzerland
29. Department of Genetics, Stanford University, Stanford, CA, USA
30. Cancer Center and Department of Pathology, Massachusetts General Hospital, Boston, MA, USA
31. Harvard Medical School, Boston, MA, USA
32. Universitat Pompeu Fabra (UPF), Barcelona, Catalonia, Spain
33. Department of Genetics, Harvard Medical School, Boston, MA, USA
34. Program in Medical and Population Genetics, The Broad Institute of Massachusetts Institute of Technology and Harvard University, Cambridge, MA, USA
35. Stanley Center for Psychiatric Research, Broad Institute, Cambridge, MA, USA

36. Analytic and Translational Genetics Unit, Massachusetts General Hospital, Boston, MA, USA
37. Department of Human Genetics, University of Chicago, Chicago, IL, USA
38. Center for Genetic Medicine, Department of Pharmacology, Northwestern University, Feinberg School of Medicine, Chicago, IL, USA
39. Department of Twin Research and Genetic Epidemiology, King's College London, London, UK
40. Department of Statistics, University of Chicago, Chicago, IL, USA
41. Department of Biomathematics, University of California, Los Angeles, Los Angeles, CA, USA
42. Department of Genetics, Washington University School of Medicine, St. Louis, Missouri, USA
43. Division of Genetics, Oregon National Primate Research Center, Oregon Health & Science University, Portland, OR, USA
44. Department of Biomedical Informatics, Columbia University, New York, NY, USA
45. Department of Computer Science, University of California, Los Angeles, Los Angeles, CA, USA
46. Program in Biomedical Informatics, Stanford University School of Medicine, Stanford, CA, USA
47. Computer Science and Artificial Intelligence Laboratory, Massachusetts Institute of Technology, Cambridge, MA, USA
48. Department of Clinical Pharmacy, School of Pharmacy, University of Southern California, Los Angeles, CA, USA
49. Department of Statistics and Operations Research and Department of Biostatistics, University of North Carolina, Chapel Hill, NC, USA
50. Department of Bioengineering, Stanford University, Stanford, CA, USA
51. Department of Genetics, Microbiology and Statistics, University of Barcelona, Barcelona. Spain.
52. Departments of Biomedical Data Science and Statistics, Stanford University, Stanford, CA, USA
53. Department of Pathology and Laboratory Medicine, Ann & Robert H. Lurie Children's Hospital of Chicago, Chicago, IL, USA
54. Bioinformatics Research Center and Departments of Statistics and Biological Sciences, North Carolina State University, Raleigh, NC, USA
55. Department of Computer Sciences, Faculty of Sciences, University of Porto, Porto, Portugal
56. Instituto de Investigação e Inovação em Saúde, University of Porto, Porto, Portugal
57. Institute of Molecular Pathology and Immunology, University of Porto, Porto, Portugal
58. Laboratory of Artificial Intelligence and Decision Support, Institute for Systems and Computer Engineering, Technology and Science, Porto, Portugal
59. Columbia University Mailman School of Public Health, New York, NY, USA
60. Life Sciences Department, Barcelona Supercomputing Center, Barcelona, Spain
61. Department of Clinical Biochemistry and Pharmacology, Ben-Gurion University of the Negev, Beer-Sheva, Israel
62. National Institute for Biotechnology in the Negev, Beer-Sheva, Israel
63. Leidos Biomedical, Rockville, MD, USA
64. UNYTS, Buffalo, NY, USA
65. Washington Regional Transplant Community, Annandale, VA, USA
66. Therapeutics, Roswell Park Comprehensive Cancer Center, Buffalo, NY, USA
67. Gift of Life Donor Program, Philadelphia, PA, USA
68. LifeGift, Houston, TX, USA
69. Center for Organ Recovery and Education, Pittsburgh, PA, USA
70. LifeNet Health, Virginia Beach, VA. USA

71. National Disease Research Interchange, Philadelphia, PA, USA
72. Van Andel Research Institute, Grand Rapids, MI, USA
73. Department of Neurology, University of Miami Miller School of Medicine, Miami, FL, USA
74. Biorepositories and Biospecimen Research Branch, Division of Cancer Treatment and Diagnosis, National Cancer Institute, Bethesda, MD, USA
75. Temple University, Philadelphia, PA, USA
76. Virginia Commonwealth University, Richmond, VA, USA
77. European Molecular Biology Laboratory, European Bioinformatics Institute, Hinxton, United Kingdom
78. Genomics Institute, University of California Santa Cruz, Santa Cruz, CA, USA
79. Carl Icahn Laboratory, Princeton University, Princeton, NJ, USA
80. Department of Population Health Sciences, The University of Utah, Salt Lake City, Utah, USA
81. Departments of Medicine, Biomedical Engineering, and Mental Health, Johns Hopkins University, Baltimore, MD, USA
82. Department of Biostatistics, Bloomberg School of Public Health, Johns Hopkins University, Baltimore, MD, USA
83. Department of Medical Biology, The Walter and Eliza Hall Institute of Medical Research, Parkville, Victoria, Australia
84. Altius Institute for Biomedical Sciences, Seattle, WA, USA
85. Division of Genetics, University of Washington, Seattle, WA, University of Washington, Seattle, WA, USA
86. Department of Cardiology, University of Washington, Seattle, WA, USA
87. HudsonAlpha Institute for Biotechnology, Huntsville, AL, USA
88. Genome Sciences, University of Washington, Seattle, WA, USA
89. National Institute of Dental and Craniofacial Research, Bethesda, MD, USA
90. Division of Neuroscience and Basic Behavioral Science, National Institute of Mental Health, National Institutes of Health, Bethesda, MD, USA
91. National Institute on Drug Abuse, Bethesda, MD, USA
92. Office of Strategic Coordination, Division of Program Coordination, Planning and Strategic Initiatives, Office of the Director, National Institutes of Health, Rockville, MD, USA
93. Division of Genomic Medicine, National Human Genome Research Institute, Bethesda, MD, USA
