# Supplementary material for: Predicting molecular mechanisms of hereditary diseases by using their tissue‐selective manifestation
Source: Mol Syst Biol. 2023 May 26;19(8):e11407. doi: 10.15252/msb.202211407 (PMC10407743; doi:10.15252/msb.202211407)
Supplement: Supplementary file 7 [file MSB-19-e11407-s006.pdf]

molecular systems biology

Predicting molecular mechanisms of hereditary diseases by using their tissue-selective manifestation

Eyal Simonovsky, Moran Sharon, Maya Ziv, Omry Mauer, Idan Hekselman, Juman Jubran, Ekaterina Vinogradov, Chanan Argov, Omer Basha, Lior Kerber, Yuval Yogev, Ayellet Segre, Hae Im, Ohad Birk, Lior Rokach, and Esti Yeger-Lotem
DOI: 10.15252/msb.202211407

Corresponding author(s): Esti Yeger-Lotem (estiyl@bgu.ac.il)

Review Timeline:

Submission Date:	20th Oct 22
Editorial Decision:	6th Dec 22
Revision Received:	6th Mar 23
Editorial Decision:	3rd Apr 23
Revision Received:	30th Apr 23
Accepted:	10th May 23

Editor: Jingyi Hou / Maria Polychronidou

Transaction Report:

(Note: With the exception of the correction of typographical or spelling errors that could be a source of ambiguity, letters and reports are not edited. Depending on transfer agreements, referee reports obtained elsewhere may or may not be included in this compilation. Referee reports are anonymous unless the Referee chooses to sign their reports.)

6th Dec 2022

Manuscript Number: MSB-2022-11407
Title: Predicting molecular mechanisms of hereditary diseases by using their tissue-selective manifestation
Author: Eyal Simonovsky
Moran Sharon
Maya Ziv
Omry Mauer
Idan Hekselman
Juman Jubran
Ekaterina Vinogradov
Chanan Argov
Omer Basha
Lior Kerber
Yuval Yogev
Ayellet Segre
Hae Im
Ohad Birk
Lior Rokach
Esti Yeger-Lotem

Dear Prof Yeger-Lotem,

Thank you for submitting your work to Molecular Systems Biology. I apologize for the delay in sending you an editorial decision, which was due to the late arrival of the reviewers' report. We have now heard back from the three reviewers who agreed to evaluate your manuscript. As you will see from the reports below, the reviewers acknowledge the potential interest of the study. They raise however a series of concerns, which we would ask you to address in a major revision.

I think the reviewers' recommendations are relatively clear, so there is no need to repeat the points listed below. In particular, Reviewer#1's concerns refer to the need to better demonstrate the superiority of TRACE over existing methods and to include additional analysis and discussion about clinical applications, which should be carefully addressed; Overstatements about novelty should be avoided. Reviewer #3's concern about the method's assessment needs to be addressed.

All other issues raised by the reviewers need to be satisfactorily addressed as well. As you may already know, our editorial policy allows in principle a single round of major revision, and it is therefore essential to provide responses to the reviewers' comments that are as complete as possible. Please feel free to contact me in case you would like to discuss in further detail any of the issues raised by the reviewers.

On a more editorial level, we would ask you to address the following issues:

- Please provide a .docx formatted version of the manuscript text (including legends for main figures, EV figures and tables). Please make sure that the changes are highlighted to be clearly visible.

- Please provide individual production quality figure files as .eps, .tif, .jpg (one file per figure).

-Please provide a .docx formatted letter INCLUDING the reviewers' reports and your detailed point-by-point responses to their comments. As part of the EMBO Press transparent editorial process, the point-by-point response is part of the Review Process File (RPF), which will be published alongside your paper.

-Please note that all corresponding authors are required to supply an ORCID ID for their name upon submission of a revised manuscript.

-We replaced Supplementary Information with Expanded View (EV) Figures and Tables that are collapsible/expandable online (see examples in http://msb.embopress.org/content/11/6/812). A maximum of 5 EV Figures can be typeset. EV Figures should be cited as 'Figure EV1, Figure EV2" etc... in the text and their respective legends should be included in the main text after the legends of regular figures.

Additional Tables/Datasets should be labeled and referred to as Table EV1, Dataset EV1, etc. Legends have to be provided in a separate tab in case of .xls files. Alternatively, the legend can be supplied as a separate text file (README) and zipped together with the Table/Dataset file.

For the figures and tables that you do NOT wish to display as Expanded View figures, they should be bundled together with their legends in a single PDF file called *Appendix*, which should start with a short Table of Content. Each legend should be below the corresponding Figure/Table in the Appendix. Appendix figures and tables should be referred to in the main text as: "Appendix Figure S1, Appendix Figure S2, Appendix Table S1" etc. See detailed instructions regarding expanded view here: https://www.embopress.org/page/journal/17444292/authorguide#expandedview.

-Before submitting your revision, primary datasets (and computer code, where appropriate) produced in this study need to be deposited in an appropriate public database (seehttp://msb.embopress.org/authorguide - dataavailability https://www.embopress.org/page/journal/17444292/authorguide#dataavailability).

Please remember to provide a reviewer password if the datasets are not yet public.

The accession numbers and database should be listed in a formal "Data Availability" section (placed after Materials & Method) that follows the model below (see also https://www.embopress.org/page/journal/17444292/authorguide#dataavailability). Please note that the Data Availability Section is restricted to new primary data that are part of this study.

Data availability

The datasets (and computer code) produced in this study are available in the following databases:

- RNA-Seq data: Gene Expression Omnibus GSE46843 (https://www.ncbi.nlm.nih.gov/geo/query/acc.cgi?acc=GSE46843)
- [data type]: [name of the resource] [accession number/identifier/doi] ([URL or identifiers.org/DATABASE:ACCESSION])

*** Note - All links should resolve to a page where the data can be accessed. ***

-At EMBO Press we ask authors to provide source data for the main figures. Our source data coordinator will contact you to discuss which figure panels we would need source data for and will also provide you with helpful tips on how to upload and organize the files.

- Our journal encourages inclusion of *data citations in the reference list* to directly cite datasets that were re-used and obtained from public databases. Data citations in the article text are distinct from normal bibliographical citations and should directly link to the database records from which the data can be accessed. In the main text, data citations are formatted as follows: "Data ref: Smith et al, 2001". In the Reference list, data citations must be labeled with "[DATASET]". A data reference must provide the database name, accession number/identifiers and a resolvable link to the landing page from which the data can be accessed at the end of the reference. Further instructions are available at .

- We updated our journal's competing interests policy in January 2022 and request authors to consider both actual and perceived competing interests. Please review the policy https://www.embopress.org/competing-interests and update your competing interests if necessary.

Please use the heading "Disclosure statement and competing interests".

- All Materials and Methods need to be described in the main text. We would encourage you to use 'Structured Methods', our new Materials and Methods format. According to this format, the Material and Methods section should include a Reagents and Tools Table (listing key reagents, experimental models, software and relevant equipment and including their sources and relevant identifiers) followed by a Methods and Protocols section in which we encourage the authors to describe their methods using a step-by-step protocol format with bullet points, to facilitate the adoption of the methodologies across labs. More information on how to adhere to this format as well as downloadable templates (.doc or .xls) for the Reagents and Tools Table can be found in our author guidelines: < https://www.embopress.org/page/journal/17444292/authorguide#researcharticleguide>. An example of a Method paper with Structured Methods can be found here: .

-Regarding data quantification:
Please ensure to specify the name of the statistical test used to generate error bars and P values, the number (n) of independent experiments (please specify technical or biological replicates) underlying each data point and the test used to calculate p-values in each figure legend. Discussion of statistical methodology can be reported in the materials and methods section, but figure legends should contain a basic description of n, P and the test applied.
Graphs must include a description of the bars and the error bars (s.d., s.e.m.).
Please also include scale bars in all microscopy images.

- Please provide a "standfirst text" summarizing the study in one or two sentences (approximately 250 characters, including space), three to four "bullet points" highlighting the main findings and a "synopsis image" (550px width and 400-600 px height, PNG format) to highlight the paper on our homepage.
Here are a couple of examples:
https://www.embopress.org/doi/10.15252/msb.20199356

https://www.embopress.org/doi/10.15252/msb.20209475
https://www.embopress.org/doi/10.15252/msb.209495

When you resubmit your manuscript, please download our CHECKLIST (https://www.embopress.org/pb-assets/embo-site/EMBO%20Press%20Author%20Checklist-1642513524327.xlsx) and include the completed form in your submission. *Please note* that the Author Checklist will be published alongside the paper as part of the transparent process (https://www.embopress.org/page/journal/17444292/authorguide#transparentprocess).

If you feel you can satisfactorily deal with these points and those listed by the referees, you may wish to submit a revised version of your manuscript. Please attach a covering letter giving details of the way in which you have handled each of the points raised by the referees. A revised manuscript will be once again subject to review and you probably understand that we can give you no guarantee at this stage that the eventual outcome will be favorable.

Kind regards,
Jingyi

Jingyi Hou, PhD
Scientific Editor
Molecular Systems Biology

We realize that it is difficult to revise to a specific deadline. In the interest of protecting the conceptual advance provided by the work, we recommend a revision within 3 months (6th Mar 2023). Please discuss the revision progress ahead of this time with the editor if you require more time to complete the revisions. Use the link below to submit your revision:

https://msb.msubmit.net/cgi-bin/main.plex

IMPORTANT: When you send your revision, we will require the following items:
1. the manuscript text in LaTeX, RTF or MS Word format
2. a letter with a detailed description of the changes made in response to the referees. Please specify clearly the exact places in the text (pages and paragraphs) where each change has been made in response to each specific comment given
3. three to four 'bullet points' highlighting the main findings of your study
4. a short 'blurb' text summarizing in two sentences the study (max. 250 characters)
5. a 'thumbnail image' (550px width and max 400px height, Illustrator, PowerPoint or jpeg format), which can be used as 'visual title' for the synopsis section of your paper.
6. Please include an author contributions statement after the Acknowledgements section (see https://www.embopress.org/page/journal/17444292/authorguide)
7. Please complete the CHECKLIST available at (https://bit.ly/EMBOPressAuthorChecklist).
Please note that the Author Checklist will be published alongside the paper as part of the transparent process (https://www.embopress.org/page/journal/17444292/authorguide#transparentprocess).
8. When assembling figures, please refer to our figure preparation guideline in order to ensure proper formatting and readability in print as well as on screen:
https://bit.ly/EMBOPressFigurePreparationGuideline
See also figure legend guidelines: https://www.embopress.org/page/journal/17444292/authorguide#figureformat
9. Please note that corresponding authors are required to supply an ORCID ID for their name upon submission of a revised manuscript (EMBO Press signed a joint statement to encourage ORCID adoption).
(https://www.embopress.org/page/journal/17444292/authorguide#editorialprocess)
Currently, our records indicate that the ORCID for your account is 0000-0002-8279-7898.

Please click the link below to modify this ORCID:
Link Not Available

10. At EMBO Press we ask authors to provide source data for the main manuscript figures. Our source data coordinator will contact you to discuss which figure panels we would need source data for and will also provide you with helpful tips on how to upload and organize the files.

The system will prompt you to fill in your funding and payment information. This will allow Wiley to send you a quote for the article processing charge (APC) in case of acceptance. This quote takes into account any reduction or fee waivers that you may be eligible for. Authors do not need to pay any fees before their manuscript is accepted and transferred to the publisher.

EMBO Press participates in many Publish and Read agreements that allow authors to publish Open Access with reduced/no publication charges. Check your eligibility: https://authorservices.wiley.com/author-resources/Journal-Authors/open-

access/affiliation-policies-payments/index.html

As a matter of course, please make sure that you have correctly followed the instructions for authors as given on the submission website.

*** PLEASE NOTE *** As part of the EMBO Press transparent editorial process initiative (see our Editorial at https://dx.doi.org/10.1038/msb.2010.72), Molecular Systems Biology publishes online a Review Process File with each accepted manuscripts. This file will be published in conjunction with your paper and will include the anonymous referee reports, your point-by-point response and all pertinent correspondence relating to the manuscript. If you do NOT want this File to be published, please inform the editorial office at msb@embo.org within 14 days upon receipt of the present letter.

Reviewer #1:

The authors present a ML model, based on thousands of features, to rank genes by their potential to affect tissue-specific diseases. The problem addressed is important and work is well executed and well presented. However, I feel like the novelty is overstated and the impact is a bit exaggerated. My comments are below.

1. I do not I agree with 'we present a first ML approach for revealing and explaining tissue-specific functional consequences of genetic variation...'. The problem of prioritizing genes by their tissue-specific functionality using ML approach has been taken on previously, also cited by the authors, notably, Greene et al. and Somepalli et al. Admittedly, the feature set used in this work is far greater than those used previously, but that is a quantitative, as opposed to qualitative advance. In fact, even the method of choice - XGBoost, is the same as before. I think the authors to up front acknowledge this and clarify the advance their work represents.

2. Gold sets can make a substantial difference in the reported performance. Continuing with comment #1, authors should also clarify how their gold set differs from those previous curated gold sets, which seem to also rely on OMIM and HPO.

3. In terms of prioritization of verified pathogenic variants, in 34% of the cases the correct variant is among the top 10% of the genes. This does represent and advance but far from being clinically actionable, given a typically large number of candidate variants. At the least, the authors should provide a discussion based on number of candidate variants in a patient cohort.

4. Along these lines, it would be useful to take a close look at the top (say, 5) candidates for each patient and do an in-depth biological/functional analysis of those to see to what extent they can be interpreted and represent and potent candidate warranting a follow-up.

5. On Page 10: "The top contributing feature of each model was the high differential
6. process activity of the disease gene in its disease-affected tissue". By "high", do they mean "Max"? Otherwise, this statement doesn't make sense to me.

7. The examples provided in Fig 2 are very nice, but they are after all hand-picked examples. Some indication of how they were arrived at needs to be provided. And does this justify a general statement such as 'Encouraged by the interpretability of TRACE predictions'? I think this needs to be toned down.

8. With regards to accuracy, Somepalli et al report an auROC of 0.82 overall (not tissue-specific) based on XGB and a subset of features that the current work uses. In the current work also, XGB seems to be sufficient. Authors need to more firmly establish the superiority of TRACE over and above those reported before. Also, the integration of 5 different ML tools needs to be justified better - it's not clear based on auROC alone.

9. In terms of feature importance, use of SHAP values is established. However, a more direct demonstration of drop in accuracy when a class of features is excluded is most convincing. I think the discovery that tissue-specific activity of GO functions that a gene is involved is quite interesting. So, at least for this class of features, I would like to see what happens if this class of features are excluded. Do we see a substantial drop in accuracy?

10. A particular type of accuracy assessment is currently missing. It is important to be able to ascribe a gene variant to a specific tissue. While the authors have shown that TRACE can meaningfully rank genes in a tissue, it would be critical to show that the for a given gene, based on its tissue-specific TRACE score, how often it is assigned to the correct tissue.

11. TRACE application to rare disease variants is quite nice. However, I do not understand why the model is considered 'personalized'. As far as I understand, the candidate genes are being excluded and a model is trained still based on the original gold set of tissue-relevant and tissue-non-relevant genes. Neither the gold set, nor the features have anything to do with the patient (except for the exclusion of candidate genes). So what really make this model 'patient-specific'? In other words,

assuming there is no overlap between candidate genes and the gold set genes, what difference could it make if the gene ranking based on original tissue model was used to infer the candidate genes?

12. Also in this context, I would like to see the procedure applied to all tissues and then see if the correct tissue stands out for the verified candidate gene.

In Methods:

13. eQTL features, what is eGene p-value if multiple eQTLs map to a gene?

14. What is the rationale for normalized count >= 7 threshold?

Reviewer #2:

Simonovsky et al presents a dataset and method (TRACE) for prioritization of candidate disease genes from human sequencing. The approach focus on identifying/ranking genes that could be causal in Mendelian and rare heritable diseases. To achieve this the authors collect transcriptomics data, protein-protein interactions data and ontology data and create features as input to machine learning models for prediction of disease/causal genes relevant in particular tissues. They train and apply the model on data sets where they a) hold out the causal gene, b) try to identify disease genes from non-disease genes, c) models disease risks for genes per tissue and d) try to identify disease causing genes in rare-disease patients. Overall, the collection of data sets and feature engineering is interesting and impressive. In the benchmark TRACE has good/better performance compared to other similar methods.

Major comments:

The manuscript is well written, however I found the description of the feature data sets a bit difficult to follow. I think that a supplementary figure of how the integrated tissue-based gene features were created would be very useful. For instance, explaining PPI feature generation.

Additionally, the training scheme(s) was a bit confusing. For instance, for the exome sequenced patients, it is mentioned that the model is retrained? I would think that the model should be trained on the original data sets and then applied to the exome data, ie. without re-training? Please clarify.

When using SHAP for explainability, it is influenced by correlated features. How correlated are the input features and are the SHAP values stable across replicate runs?

Performance for different tissues:

- For some tissues TRACE had overall better performance compared to others (Fig. S10)? Can the authors elaborate a bit on this and perhaps provide guidance for which tissues it makes less sense to use the method?

- The difference in performance across tissue between holding out network embeddings. What is the reason for the difference, ie. for some tissues it has a big impact whereas for others it has less?

- This is also evident when comparing with other methods where for instance pBrit has higher PR compared to TRACE on Blood and Testis (Fig S6). Do the models have different tissues that they are the "best" at?

- Finally, how does this interact with a real clinical setting - here I guess based on the patient a potential disease causing tissue would be known from the clinician?

Following this, will TRACE give a low confidence score if a gene is input that is not associated with a disease in any of the included tissues?

I am not sure the authors can claim that they provide "mechanistic insights" into the diseases, rather they provide associations of genes to diseases?

Minor:

Please define "differential process activity", I could not find it in the manuscript and is not familiar with it.

Equation 1: I am not sure I understand how IQR (a range) can be part of the divisor?

Normalized counts >= 7 used as a threshold, how was this decided?

Tissue specific interactors: A threshold of 20% is chosen, what was the rationale behind this value?

Not possible to see/read what is on Fig. S2

"To assess TRACE per patient, we compiled a list of her genes that contained variants, henceforth referred to as candidate disease genes." It seems that "her" is a typo?

For Blood the most important features (from SHAP) are Brain, Brain, Liver. Why not blood first? (Fig. S4)

Reviewer #3:

In their manuscript entitled "Predicting molecular mechanisms of hereditary diseases by using their tissue-selective manifestation", the authors make an impressive computational effort to address the issue of tissue-selectivity for hereditary diseases, using multiple machine learning techniques.

However, I have some major and other concerns.

Major concerns:
1) I think the pipeline is treating tissue specificity as mostly a problem of a gene-specific overexpression, while overlooking that tissue specificity for a hereditary disease can be the lack of a specific gene expression.
2) I understand that this is a ranking prioritization effort, but the lack of an independent validation dataset does not seem to allow the method's assessment in terms of type II error. For example, throughout the paper the authors consistently report AUC values from cross validation (CV) but i) CV can be overconfident (especially for methods like boosted decision trees which have been shown to be prone to overfitting), and ii) we don't know the algorithms performance in terms of false negatives, or the balance between sensitivity and specificity, or type I and type II error.
3) The previous two concerns result in the lack of confidence for the algorithm's output. For example, while the method seems to outperform other -comparable or not methods- it feels like an overkill to end up selecting half the gene candidates based on median values.

Other concerns
A. I could not find table S1 in the submitted material.
B. While I particularly liked the figures for their appearance and clarity, panels are missing error bars, even if they are reporting averages.
C. The introduction's second paragraph is read in a generic way within a general context of Machine Learning (ML) without providing much useful information for the reader to understand the literature of ML and classification algorithms in the paper's context. The authors use a variety of ML techniques without any clear justification, while machine learning is an umbrella term. More specific examples of past use and their respective outcomes can be useful. Granted that some of this information is provided later within the results section, but it seems odd for someone to have to read the whole paper ta familiarize himself/herself with part of the literature.
D. The excel files are provided in a way that I could not read the content.

Dear Dr. Jingyi Hou and Anonymous Reviewers,

We thank you for your many valuable and insightful comments on our manuscript, titled ‘Predicting molecular mechanisms of hereditary diseases by using their tissue-selective manifestation’.

Following the comments, we revised the manuscript text, performed several additional analyses as requested by the reviewers, added multiple assessments of the algorithm including comparison to a closely-related, recently-published method, and extended the discussion of clinical applications. The resulting manuscript is accompanied by revised figure panels, supplementary figures and supplementary tables.

A detailed point-by-point response to each comment is provided below. The respective changes to the manuscript were marked in blue.

Thank you for your kind consideration,

Sincerely,

Esti Yeger-Lotem

Professor Esti Yeger-Lotem, PhD
Head, Department of Clinical Biochemistry and Pharmacology
Faculty of Health Sciences
Ben-Gurion University of the Negev, Israel

<u>Reviewer #1:</u>

The authors present a ML model, based on thousands of features, to rank genes by their potential to affect tissue-specific diseases. The problem addressed is important and work is well executed and well presented. However, I feel like the novelty is overstated and the impact is a bit exaggerated. My comments are below.

1. I do not I agree with 'we present a first ML approach for revealing and explaining tissue-specific functional consequences of genetic variation...'. The problem of prioritizing genes by their tissue-specific functionality using ML approach has been taken on previously, also cited by the authors, notably, Greene et al. and Somepalli et al. Admittedly, the feature set used in this work is far greater than those used previously, but that is a quantitative, as opposed to qualitative advance. In fact, even the method of choice - XGBoost, is the same as before. I think the authors to up front acknowledge this and clarify the advance their work represents.

We changed the text to acknowledge previous studies and to clarify the advance of our work (page 4 last paragraph, page 5 first and second paragraphs, and page 15 first Discussion paragraph).

2. Gold sets can make a substantial difference in the reported performance. Continuing with comment #1, authors should also clarify how their gold set differs from those previous curated gold sets, which seem to also rely on OMIM and HPO.

We compared our work to the study by Somepalli et al., which prioritized genes by their predicted likelihood to “serve any function in a specific tissue or context”. TRACE, in

contrast, focused specifically on prioritizing disease genes affecting a specific tissue. This difference is reflected in the gold set used by each method. Somepalli et al.'s gold set was extracted from the study of Feiglin et al., *Cell Systems* 2017 (PMID: 28822752). In both methods, the positive subset of the gold set associated diseases with genes according to OMIM. However, in (PMID: 28822752) "all evidence codes linking genes to diseases were included", whereas TRACE used only OMIM associations where the molecular basis of the disorder is known. The negative subset of the gold set used by Somepalli et al included gene-tissue pairs where the gene was *not expressed* in the tissue. The negative set of TRACE, in contrast, included genes and tissues with *no disease association*. This clarification was added to page 16 middle of second paragraph. In the study of Greene et al., the gold set contained gene pairs (functionally-related gene pairs were labeled positive, unrelated pairs were labeled negative) and thus was less relevant for comparison.

3. In terms of prioritization of verified pathogenic variants, in 34% of the cases the correct variant is among the top 10% of the genes. This does represent and advance but far from being clinically actionable, given a typically large number of candidate variants. At the least, the authors should provide a discussion based on number of candidate variants in a patient cohort.

We acknowledge this limitation in page 16, end of second paragraph.

4. Along these lines, it would be useful to take a close look at the top (say, 5) candidates for each patient and do an in-depth biological/functional analysis of those to see to what extent they can be interpreted and represent and potent candidate warranting a follow-up.

We thank the reviewer for this suggestion. Per patient, we collected the top 5 candidate genes according to TRACE (265 genes total). We then checked how many of them participated in a gene ontology (GO) biological process that was specific to the disease-affected tissue of the patient, and hence could lead to tissue-selective phenotypes. Data of tissue-specific GO biological processes was obtained from Sharon et al, *Bioinformatics*, 2022 (PMID: 35015838). Out of the 265 genes, 227 genes were associated with a tissue-specific biological process. Of those, 200 genes (75% of the 265 genes, and 88% of the process-associated genes) participated in a GO biological process that was specific to the affected tissue. For example, in patient #13786 that was diagnosed with muscular dystrophy, the top 5 genes (including the verified disease gene SGCG) were all associated with GO biological processes that were specific to muscle, such as 'muscle contraction' and 'muscle organ development'. Hence, the list of top candidates per patient can indeed point to genes with tissue-relevant functions. We describe this analysis in page 14 third paragraph and page 32, 'Analysis of top five candidate genes per patient', and added the list of top candidates as Appendix Table S4D.

5. On Page 10: "The top contributing feature of each model was the high differential
6. process activity of the disease gene in its disease-affected tissue". By "high", do they mean "Max"? Otherwise, this statement doesn't make sense to me.

We indeed meant "Max" (highest), and revised the text to clarify this point (page 8, second paragraph).

7. The examples provided in Fig 2 are very nice, but they are after all hand-picked examples. Some indication of how they were arrived at needs to be provided. And does

this justify a general statement such as 'Encouraged by the interpretability of TRACE predictions'? I think this needs to be toned down.

We added an explanation for the selected examples (page 8, beginning of second paragraph). We also toned down the text and removed the general statement mentioned above (page 9, first paragraph).

8. With regards to accuracy, Somepalli et al report an auROC of 0.82 overall (not tissue-specific) based on XGB and a subset of features that the current work uses. In the current work also, XGB seems to be sufficient. Authors need to more firmly establish the superiority of TRACE over and above those reported before. Also, the integration of 5 different ML tools needs to be justified better - it's not clear based on auROC alone.

To test and establish TRACE superiority we added a comparison to the FUGUE tool of Somepalli et al. For this, we used FUGUE to rank candidate genes in patients, as we did with other tools (Fig. 5D). Across patients, TRACE was able to rank more protein-coding genes than FUGUE (median number of ranked genes across patients: TRACE 186, FUGUE 157). We then compared the rank of the verified disease gene between TRACE and FUGUE. TRACE ranked the verified disease gene better than FUGUE in 35 of the 53 cases (66%, p=0.016, Wilcoxon signed-rank test). Furthermore, when we limited the list of ranked candidate genes to those that were ranked by both TRACE and FUGUE, TRACE ranked the verified disease gene better than FUGUE in 38 of the 53 cases (72%, p=0.0042, Wilcoxon signed-rank test). We revised the text to include the comparison between TRACE and FUGUE based on the non-limited list of candidate genes (page 15 first paragraph, Fig. 5D, and Appendix Table S4).

As the reviewer commented, XGB was used by both TRACE and FUGUE of Somepalli et al. However, in TRACE, XGB performed better than other ML tools in only two of the eight tissue models with respect to auROC, and in two other tissue models with respect to auPRC. We therefore did not consider XGB to be sufficient, and instead implemented TRACE as a meta-learner that integrated 5 different ML tools. The integration is justified in page 10, second paragraph.

9. In terms of feature importance, use of SHAP values is established. However, a more direct demonstration of drop in accuracy when a class of features is excluded is most convincing. I think the discovery that tissue-specific activity of GO functions that a gene is involved is quite interesting. So, at least for this class of features, I would like to see what happens if this class of features are excluded. Do we see a substantial drop in accuracy?

To test what happens, we excluded features related to tissue-specific activity of GO processes that a gene is involved from the features dataset, and recreated TRACE models per tissue, which we denote as limited models. Next, we compared the performance between the original and limited models (Fig. 1 below). In terms of the auROC (blue bars), performance of the eight tissue models was only slightly reduced by the removal of these features (mean change of minus 0.9%). However, in terms of the auPRC (red bars), performance was much more affected by the removal of these features (mean change of minus 15%). Specifically, 4/8 tissue models showed mild to moderate change in performance (auPRC scores ranging between minus 8.3% to minus 17% difference) and 2/8 showed significant drop in performance (minus 37% and minus 44% difference). Considering these two results together, it appears that models were robust to the removal of process activity features when predicting non-disease associated genes as such (true negatives), and much more dependent on these features when predicting disease-associated genes as such (true positives, which were much scarcer). Overall, this analysis

highlights the importance of process activity features when trying to prioritize disease-associated genes, in addition to their contribution toward illuminating disease-related pathways.

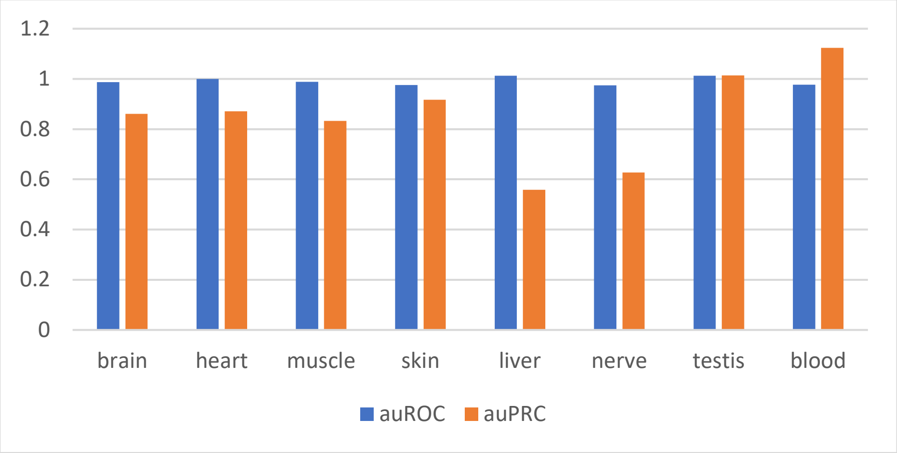

Fig. 1. auROC and auPRC of the limited versus original models. Values were normalized to the original models.

10. A particular type of accuracy assessment is currently missing. It is important to be able to ascribe a gene variant to a specific tissue. While the authors have shown that TRACE can meaningfully rank genes in a tissue, it would be critical to show that the for a given gene, based on its tissue-specific TRACE score, how often it is assigned to the correct tissue.

To test that, we compared the rank of disease genes between their correct associated tissue and other tissues. The median rank in the model of the correct associated tissue was higher than the median rank in other tissue models, except for blood-associated genes whose median rank was higher in liver. We added this analysis to the text (page 11, end of second paragraph) and as a new supplementary figure (Appendix Fig. S10).

11. TRACE application to rare disease variants is quite nice. However, I do not understand why the model is considered 'personalized'. As far as I understand, the candidate genes are being excluded and a model is trained still based on the original gold set of tissue-relevant and tissue-non-relevant genes. Neither the gold set, nor the features have anything to do with the patient (except for the exclusion of candidate genes). So what really make this model 'patient-specific'? In other words, assuming there is no overlap between candidate genes and the gold set genes, what difference could it make if the gene ranking based on original tissue model was used to infer the candidate genes?

We thank the reviewer for these comments. Indeed, the models were not patient-specific, except for the case where the gold set overlapped with candidate genes of a patient. To ensure unbiased testing for such patients, we excluded the candidate genes of the patient from the gold set. We revised the text and Fig. 1E to remove references to patient-specific models.

12. Also in this context, I would like to see the procedure applied to all tissues and then see if the correct tissue stands out for the verified candidate gene.

We applied the same procedure to all tissues. Specifically, we compared the ranks of verified disease gene between the correct (affected) tissue model and other tissue models. The ranks in the correct tissue model were significantly higher ($p<0.00011$, Mann-Whitney U test (MW), Appendix Figure S11A). This analysis was added to page 14, beginning of third paragraph.

In Methods:

13. eQTL features, what is eGene p-value if multiple eQTLs map to a gene?

We assigned each gene with a gene-specific q-value that was provided by GTEx. We clarified this in the text (page 20, 'eQTL features').

14. What is the rationale for normalized count >= 7 threshold?

We applied a threshold of 7 counts per million (linear scale, not log). This is slightly more conservative than expression cutoffs used by GTEx in previous studies (e.g., GTEx consortium, *Science* 2020). We used this cutoff since we focused only on protein-coding genes, which tend to be more highly expressed than noncoding RNAs (Mele et al, *Science* 2015), and wanted to make sure that our results were not confounded by false positive expression. This was clarified in page 19 first paragraph.

Reviewer #2:

Simonovsky et al presents a dataset and method (TRACE) for prioritization of candidate disease genes from human sequencing. The approach focus on identifying/ranking genes that could be causal in Mendelian and rare heritable diseases. To achieve this the authors collect transcriptomics data, protein-protein interactions data and ontology data and create features as input to machine learning models for prediction of disease/causal genes relevant in particular tissues. They train and apply the model on data sets where they a) hold out the causal gene, b) try to identify disease genes from non-disease genes, c) models disease risks for genes per tissue and d) try to identify disease causing genes in rare-disease patients. Overall, the collection of data sets and feature engineering is interesting and impressive. In the benchmark TRACE has good/better performance compared to other similar methods.

Major comments:

The manuscript is well written, however I found the description of the feature data sets a bit difficult to follow. I think that a supplementary figure of how the integrated tissue-based gene features were created would be very useful. For instance, explaining PPI feature generation.

We added a supplementary figure that explains how tissue-based gene features were created (Appendix Figure S1).

Additionally, the training scheme(s) was a bit confusing. For instance, for the exome sequenced patients, it is mentioned that the model is retrained? I would think that the model should be trained on the original data sets and then applied to the exome data, ie. without re-training? Please clarify.

The reason we retrained the model was to ensure that the model is not trained and tested on the same genes, otherwise we would cause data leakage. Therefore, per patient we withheld the patient`s candidate disease genes from the training dataset, which allowed us to prioritize them by an independently-trained TRACE model. We clarified this in page 13 second paragraph and page 31 last paragraph.

When using SHAP for explainability, it is influenced by correlated features. How correlated are the input features and are the SHAP values stable across replicate runs?

The features dataset included highly correlated features, for example, gene expression levels in sun-exposed and sun-not-exposed skin. Since the ML methods XGB, LR, and LR+GB are sensitive to non-contributing features (such as highly correlated features), we applied a feature selection step, using SVM, to all applications and folds of these ML methods (RF and MLP inherently handle non-contributing features). This reduced the input to a combination of the 50 most contributing features per model, thereby eliminating highly-correlated features. As an example, Fig. 2A below shows the correlation between the 50 features selected upon running the skeletal muscle tissue model. As a result, SHAP analysis was applied mostly to non-correlated features.
To check if SHAP values were stable across runs, we conducted 5 replicate runs of the muscle tissue model and analyzed each run using SHAP. Replicated runs were extremely correlated in terms of features selected (differed by one feature at most) and their SHAP values (Fig. 2B-C below).

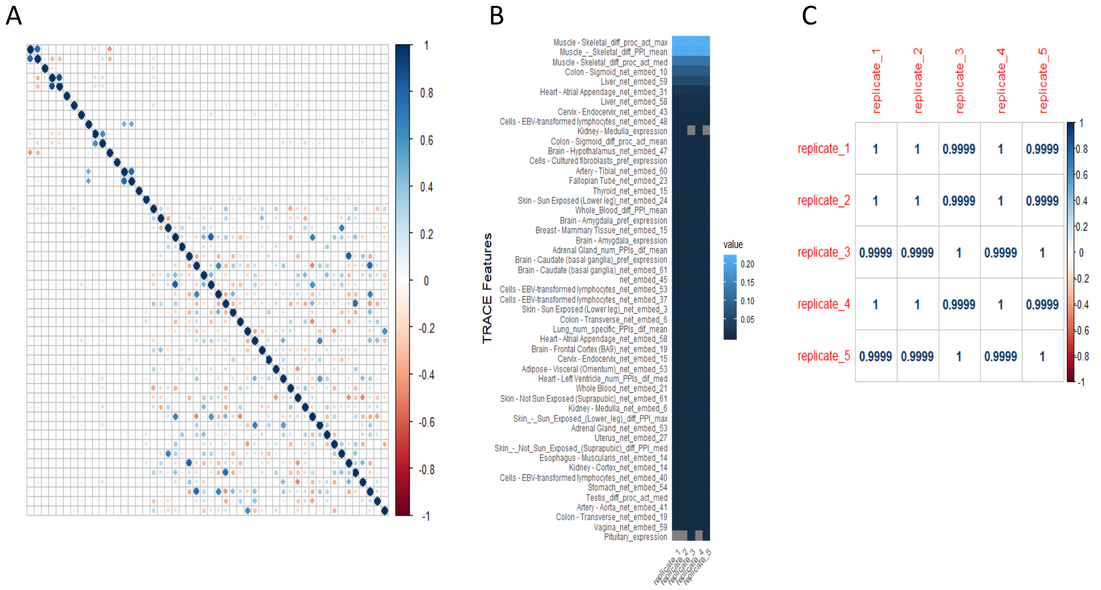

Fig. 2. Feature correlation and SHAP stability across replicate runs.
A. Correlation matrix (Pearson) of the 50 features selected by replicate run #1 across all 18,927 genes in the dataset. Preason r values between non-identical features ranged from X-y, with a median value of X and a top value of Y.
B. The heatmap shows the SHAP values of features (rows) across 5 replicated runs (columns). The 50 features selected in each run differed by one feature at the most (grey-colored square).
C. Pearson correlations of SHAP values between 5 replicate runs.

Performance for different tissues:

- For some tissues TRACE had overall better performance compared to others (Fig. S10)? Can the authors elaborate a bit on this and perhaps provide guidance for which tissues it makes less sense to use the method?

In general, better performing models (i.e., brain, heart, skeletal muscle, and skin) corresponded to tissues with larger numbers of tissue-associated disease genes. We elaborate on this on page 11 first paragraph and page 16 second paragraph.

- The difference in performance across tissue between holding out network embeddings. What is the reason for the difference, ie. for some tissues it has a big impact whereas for others it has less?

The impact is mostly seen with respect to auPRC in heart and muscle models. These closely-related tissues were shown to have a similar expression profile that is distinct from other tissues. This difference translates into differences in the protein-protein interaction networks of these tissues relative to networks of other tissues, which could explain why lack of network embeddings features reduces the models' performance.

- This is also evident when comparing with other methods where for instance pBrit has higher PR compared to TRACE on Blood and Testis (Fig S6). Do the models have different tissues that they are the "best" at?

In case of TRACE, we believe that differences in performance between tissues stemmed, at least partly, from the number of tissue-associated disease genes on which models were trained. Blood and testis had relatively lower numbers of such genes.

- Finally, how does this interact with a real clinical setting - here I guess based on the patient a potential disease causing tissue would be known from the clinician?

In the patient analyses, best performance was achieved for brain and skin, two tissue models that had relatively large numbers of tissue-associated disease genes and that performed well in cross-validation. We comment on it in page 14, third paragraph.

Following this, will TRACE give a low confidence score if a gene is input that is not associated with a disease in any of the included tissues?

TRACE scores of non-disease genes or genes associated with diseases in other tissues are presented in Fig. 4E and Appendix Fig. S8. Most of them have low TRACE scores, as expected. As for the small subset of genes with larger TRACE scores, they could either be noise or genes that are yet to be identified as tissue-associated disease genes.

I am not sure the authors can claim that they provide "mechanistic insights" into the diseases, rather they provide associations of genes to diseases?

We agree with the reviewer and removed "mechanistic insights" from the text.

Minor:

Please define "differential process activity", I could not find it in the manuscript and is not familiar with it.

We defined 'differential process activity' in page 6 second paragraph, and elaborated on it in Methods, page 22 last paragraph.

Equation 1: I am not sure I understand how IQR (a range) can be part of the divisor?

IQR refers to the difference between the 75th and 25th percentiles of the data. We defined it in page 19 under Equation 1.

Normalized counts >= 7 used as a threshold, how was this decided?

We applied a threshold of 7 counts per million (linear scale, not log). This is slightly more conservative than expression cutoffs used by GTEx in previous studies (e.g., GTEx consortium, *Science* 2020). We used this cutoff since we focused only on protein-coding genes, which tend to be more highly expressed than noncoding RNAs (Mele et al, *Science* 2015), and wanted to make sure that our results were not confounded by false positive expression. This was clarified in page 19 first paragraph.

Tissue specific interactors: A threshold of 20% is chosen, what was the rationale behind this value?

This threshold was selected because the GTEx dataset contained several samples of closely-related brain regions. A threshold of 20% meant that interactors expressed in closely-related brain sub-regions, but not in other tissues, would still be considered as brain-specific (tissue-specific). A lower threshold would imply that more brain-specific interactors would be considered as non-tissue-specific, and a higher threshold would

imply that interactors expressed in more than 10 distinct tissues would be considered as tissue-specific. A threshold of 20% was a midway between these two scenarios.

Not possible to see/read what is on Fig. S2

We enlarged the figure to present it on two pages and refined its resolution (Appendix Fig. S3).

"To assess TRACE per patient, we compiled a list of her genes that contained variants, henceforth referred to as candidate disease genes." It seems that "her" is a typo?

We changed this to refer explicitly to the patient (page 13 second paragraph).

For Blood the most important features (from SHAP) are Brain, Brain, Liver. Why not blood first? (Fig. S4)

The construction of each model is preceded by feature selection, in which certain correlated or anticorrelated features were filtered out. The brain features that were most important showed that low values in brain were predictive of blood-disease-genes (blue dots to the left of the vertical line), and high values in brain were predictive of non blood-disease-genes (red dots mostly to the left of the vertical line). High values in the liver-related feature were predictive of blood disease-genes, potentially because liver is enriched for blood cells. In support of this, we found that the top liver feature was positively correlated with the respective blood feature ($r=0.31$, $p<2e-16$).

Reviewer #3:

In their manuscript entitled "Predicting molecular mechanisms of hereditary diseases by using their tissue-selective manifestation", the authors make an impressive computational effort to address the issue of tissue-selectivity for hereditary diseases, using multiple machine learning techniques.

However, I have some major and other concerns.

Major concerns:
1) I think the pipeline is treating tissue specificity as mostly a problem of a gene-specific overexpression, while overlooking that tissue specificity for a hereditary disease can be the lack of a specific gene expression.

We included in the pipeline features that could reflect the lack of a specific gene expression. One set of features reflected the absolute expression levels of genes in every tissue, and values were set to zero in case a gene was not expressed. Another set of features reflected the relative expression of genes in each tissue versus all tissues. For example, the feature 'preferential expression in liver' associated each gene with its preferential expression in liver relative to all tissues. In this set of features, positive values reflected overexpression of a gene in the tissue, and negative values reflected under-expression. Furthermore, we previously showed that under-expression of paralogs of disease genes contributed to tissue specificity (Barshir et al, 2018; Jubran et al, 2020). Following these findings, we also included in our pipeline features that reflected overexpression and under-expression of paralogs. Hence, lack or under-expression of a gene could be observed through features in our pipeline. Lastly, we verified that TRACE is not limited to gene-specific overexpression by applying it to a subset of genes that were not overexpressed (new Appendix Fig. S9, referenced from page 11 last paragraph). Clarification about the ability of features to reflect under-expression was added to page 20 second paragraph, and a discussion of tissue-selective under-expression was added to page 17 second paragraph.

2) I understand that this is a ranking prioritization effort, but the lack of an independent validation dataset does not seem to allow the method's assessment in terms of type II error. For example, throughout the paper the authors consistently report AUC values from cross validation (CV) but i) CV can be overconfident (especially for methods like boosted decision trees which have been shown to be prone to overfitting), and ii) we don't know the algorithms performance in terms of false negatives, or the balance between sensitivity and specificity, or type I and type II error.

To address the risk of overconfidence or overestimated predictive performance in cross-validation, during the cross-validation we parallelly optimized all eight tissue models (i.e., used the same hyper-parameters in all eight models), to reduce the risk of overfitting. With respect to the use of an independent validation dataset, we externally validated the results using 'real world' data of patients (Fig. 5). In accordance with the results of the cross-validation, the tissue models of the brain and skin that performed well in cross-validation also performed well in patients analyses.
Following the reviewer’s comments and to allow detailed assessment of the algorithm's performance, we computed type I (false positives) and type II (false negatives) errors. For this, instead of the provided ranked list of genes, we repeatedly fixed a threshold for TRACE scores that would define 'tissue-associated disease genes' and 'non tissue-associated disease genes'. Per tissue model and per threshold, we computed the false positive rate (FPR, type I error) and false negative rate (FNR, type II error), sensitivity

and specificity, and precision and recall. These are available as new Appendix Figure S13 and as new tables detailing these metrics per tissue model (in folder “data/supplementary_tables/metrics” in Zenodo).

3) The previous two concerns result in the lack of confidence for the algorithm's output. For example, while the method seems to outperform other -comparable or not methods- it feels like an overkill to end up selecting half the gene candidates based on median values.

We would like to clarify that the main goal of TRACE was to enhance understanding of tissue selectivity and to provide a framework for testing additional tissue-selectivity features.
To strengthen confidence in TRACE results we added a new analysis, which compares TRACE to a recently published method that ranks genes by their functional relevance in tissue contexts (Somepalli et al, 2021, PMID: 34270548). As shown in the revised Fig. 5D, TRACE performed well relative to this method too (also see Appendix Fig. S11B and revised Appendix Table S4).
The analyses of data from patients served mostly as a sanity check of TRACE algorithm on external data. Notably, the candidate genes to which TRACE was applied were genes that remained *after* automatic filtration by widely used tools. Sieving through the remaining candidate genes requires geneticists to carry in-depth analyses that are time-consuming and often done manually, hence cutting the list of candidate genes by half can alleviate geneticists' work load. We revised the text to discuss this issue (page 14 second paragraph).

Other concerns
A. I could not find table S1 in the submitted material.

Table S1 was appended at the end of the Appendix file. It was also uploaded to Zenodo (https://sandbox.zenodo.org/record/1170299#.ZAY2OnZByUk, folder 'supplementary_tables').

B. While I particularly liked the figures for their appearance and clarity, panels are missing error bars, even if they are reporting averages.

Thank you for this comment. We added error bars to the relevant figures (Figure 4A, 4C, and Appendix Fig. S12).

C. The introduction's second paragraph is read in a generic way within a general context of Machine Learning (ML) without providing much useful information for the reader to understand the literature of ML and classification algorithms in the paper's context. The authors use a variety of ML techniques without any clear justification, while machine learning is an umbrella term. More specific examples of past use and their respective outcomes can be useful. Granted that some of this information is provided later within the results section, but it seems odd for someone to have to read the whole paper ta familiarize himself/herself with part of the literature.

We extended the introduction to include more background on ML classification algorithms and applications (page 4 last paragraph and page 5 first paragraph).

D. The excel files are provided in a way that I could not read the content.

Excel files were uploaded again to the journal's website as datasets and doublechecked, and to zenodo (https://sandbox.zenodo.org/record/1170299#.ZAY2OnZByUk, folder

'supplementary_tables'). In case of sub-tables (e.g., Appendix Table S4), each sub-table appeared in a separate excel worksheet in the same file.

3rd Apr 2023

Manuscript Number: MSB-2022-11407R
Title: Predicting molecular mechanisms of hereditary diseases by using their tissue-selective manifestation

Dear Prof. Yeger-Lotem,

Thank you for sending us your revised manuscript. My colleague Jingyi Hou is on maternity leave and I took over handling your manuscript. We have now heard back from the three reviewers who were asked to evaluate your revised study. As you will see below, the reviewers are satisfied with the performed revisions and support publication. Before we can formally accept the manuscript for publication, we would ask you to address some remaining editorial issues listed below.

- Our data editors have noted some missing information in the figure legends, please see the attached .doc file. Please make all requested text changes using the attached file and *keeping the "track changes" mode* so that we can easily access the edits made.

- The author list and affiliations related to the GTEx Consortium should be included in the Appendix (and not in a separate file).

- Our Data Integrity analyst noticed that some images from Figure 4D are reused in Figure EV1. We would ask you to add a sentence to the legend of Figure EV1 indicating the reuse of these figure panels.

- Please include callouts to Figure EV1A-B, Appendix Fig. S4A-B, Appendix Fig. S6A.

- Tables S2-S4 should be renamed to Dataset EV1-EV3. Please make sure that their callouts in the text are updated accordingly.

- The References in the Appendix should be formatted according to the Molecular Systems Biology reference style i.e. ordered alphabetically and listing the first 10 authors followed by et al.

- Please provide a "standfirst text" summarizing the study in one or two sentences (approximately 250 characters), three to four "bullet points" highlighting the main findings and a "synopsis image" (550px width and max 400px height, jpeg format) to highlight the paper on our homepage.

Please resubmit your revised manuscript online **within one month** and ideally as soon as possible. If we do not receive the revised manuscript within this time period, the file might be closed and any subsequent resubmission would be treated as a new manuscript. Please use the Manuscript Number (above) in all correspondence.

Click on the link below to submit your revised paper.

https://msb.msubmit.net/cgi-bin/main.plex

As a matter of course, please make sure that you have correctly followed the instructions for authors as given on the submission website.

Thank you for submitting this paper to Molecular Systems Biology.

Kind regards,

Maria

Maria Polychronidou, PhD
Senior Editor
Molecular Systems Biology

If you do choose to resubmit, please click on the link below to submit the revision online before 3rd May 2023.

https://msb.msubmit.net/cgi-bin/main.plex

IMPORTANT: When you send your revision, we will require the following items:

1. the manuscript text in LaTeX, RTF or MS Word format
2. a letter with a detailed description of the changes made in response to the referees. Please specify clearly the exact places in the text (pages and paragraphs) where each change has been made in response to each specific comment given
3. three to four 'bullet points' highlighting the main findings of your study
4. a short 'blurb' text summarizing in two sentences the study (max. 250 characters)
5. a 'thumbnail image' (550px width and max 400px height, Illustrator, PowerPoint or jpeg format), which can be used as 'visual title' for the synopsis section of your paper.
6. Please include an author contributions statement after the Acknowledgements section (see https://www.embopress.org/page/journal/17444292/authorguide#manuscriptpreparation)
7. Please complete the CHECKLIST available at (https://bit.ly/EMBOPressAuthorChecklist).
Please note that the Author Checklist will be published alongside the paper as part of the transparent process (https://www.embopress.org/page/journal/17444292/authorguide#transparentprocess).
8. When assembling figures, please refer to our figure preparation guideline in order to ensure proper formatting and readability in print as well as on screen:
https://bit.ly/EMBOPressFigurePreparationGuideline
See also figure legend guidelines: https://www.embopress.org/page/journal/17444292/authorguide#figureformat
9. Please note that corresponding authors are required to supply an ORCID ID for their name upon submission of a revised manuscript (EMBO Press signed a joint statement to encourage ORCID adoption).
(https://www.embopress.org/page/journal/17444292/authorguide#editorialprocess)
Currently, our records indicate that the ORCID for your account is 0000-0002-8279-7898.

Please click the link below to modify this ORCID:
Link Not Available

The system will prompt you to fill in your funding and payment information. This will allow Wiley to send you a quote for the article processing charge (APC) in case of acceptance. This quote takes into account any reduction or fee waivers that you may be eligible for. Authors do not need to pay any fees before their manuscript is accepted and transferred to the publisher.

As a matter of course, please make sure that you have correctly followed the instructions for authors as given on the submission website.

*** PLEASE NOTE *** As part of the EMBO Press transparent editorial process initiative (see our Editorial at https://dx.doi.org/10.1038/msb.2010.72 , Molecular Systems Biology will publish online a Review Process File to accompany accepted manuscripts. When preparing your letter of response, please be aware that in the event of acceptance, your cover letter/point-by-point document will be included as part of this File, which will be available to the scientific community. More information about this initiative is available in our Instructions to Authors. If you have any questions about this initiative, please contact the editorial office (msb@embo.org).

Reviewer #1:

Authors have responded to my comments adequately. I have no further comments.

Reviewer #2:

No further comments

Reviewer #3:

Based on the authors' response to all 3 reviewers, I have no further comments.

All editorial and formatting issues were resolved by the authors.

10th May 2023

Manuscript number: MSB-2022-11407RR
Title: Predicting molecular mechanisms of hereditary diseases by using their tissue-selective manifestation

Dear Esti,

Thank you again for sending us your revised manuscript. We are now satisfied with the modifications made and I am pleased to inform you that your paper has been accepted for publication.

*** PLEASE NOTE *** As part of the EMBO Publications transparent editorial process initiative (see our Editorial at https://dx.doi.org/10.1038/msb.2010.72), Molecular Systems Biology publishes online a Review Process File with each accepted manuscripts. This file will be published in conjunction with your paper and will include the anonymous referee reports, your point- by-point response and all pertinent correspondence relating to the manuscript. If you do NOT want this File to be published, please inform the editorial office at msb@embo.org within 14 days upon receipt of the present letter.

Should you be planning a Press Release on your article, please get in contact with msb@wiley.com as early as possible, in order to coordinate publication and release dates.

LICENSE AND PAYMENT:
All articles published in Molecular Systems Biology are fully open access: immediately and freely available to read, download and share.

Molecular Systems Biology charges an article processing charge (APC) to cover the publication costs. You, as the corresponding author for this manuscript, should have already received a quote with the article processing fee separately. Please let us know in case this quote has not been received.

Once your article is at Wiley for editorial production you will receive an email from Wiley's Author Services system, which will ask you to log in and will present you with the publication license form for completion. Within the same system the publication fee can be paid by credit card, an invoice or pro forma can be requested.

Payment of the publication charge and the signed Open Access Agreement form must be received before the article can be published online.

Molecular Systems Biology articles are published under the Creative Commons licence CC BY, which facilitates the sharing of scientific information by reducing legal barriers, while mandating attribution of the source in accordance to standard scholarly practice.

Proofs will be forwarded to you within the next 2-3 weeks.

Thank you very much for submitting your work to Molecular Systems Biology.

Kind regards,

Maria

Maria Polychronidou, PhD
Senior Editor
Molecular Systems Biology

EMBO Press Author Checklist

Corresponding Author Name: Esti Yeger-Lotem	
Journal Submitted to: Molecular Systems Biology	
Manuscript Number: MSB-2022-11407R	

USEFUL LINKS FOR COMPLETING THIS FORM
The EMBO Journal - Author Guidelines
EMBO Reports - Author Guidelines
Molecular Systems Biology - Author Guidelines
EMBO Molecular Medicine - Author Guidelines

Reporting Checklist for Life Science Articles (updated January
This checklist is adapted from Materials Design Analysis Reporting (MDAR) Checklist for Authors. MDAR establishes a minimum set of requirements in transparent reporting in the life sciences (see Statement of Task: 10.31222/osf.io/9sm4x). Please follow the journal's guidelines in preparing your
Please note that a copy of this checklist will be published alongside your article.

Abridged guidelines for figures
1. Data
The data shown in figures should satisfy the following conditions:
- the data were obtained and processed according to the field’s best practice and are presented to reflect the results of the experiments in an accurate and unbiased manner.
- ideally, figure panels should include only measurements that are directly comparable to each other and obtained with the same assay.
- plots include clearly labeled error bars for independent experiments and sample sizes. Unless justified, error bars should not be shown for technical
- if n<5, the individual data points from each experiment should be plotted. Any statistical test employed should be justified.
- Source Data should be included to report the data underlying figures according to the guidelines set out in the authorship guidelines on Data

2. Captions
Each figure caption should contain the following information, for each panel where they are relevant:
- a specification of the experimental system investigated (eg cell line, species name).
- the assay(s) and method(s) used to carry out the reported observations and measurements.
- an explicit mention of the biological and chemical entity(ies) that are being measured.
- an explicit mention of the biological and chemical entity(ies) that are altered/varied/perturbed in a controlled manner.
- the exact sample size (n) for each experimental group/condition, given as a number, not a range;
- a description of the sample collection allowing the reader to understand whether the samples represent technical or biological replicates (including how many animals, litters, cultures, etc.).
- a statement of how many times the experiment shown was independently replicated in the laboratory.
- definitions of statistical methods and measures:
  - common tests, such as t-test (please specify whether paired vs. unpaired), simple χ2 tests, Wilcoxon and Mann-Whitney tests, can be unambiguously identified by name only, but more complex techniques should be described in the methods section;
  - are tests one-sided or two-sided?
  - are there adjustments for multiple comparisons?
  - exact statistical test results, e.g., P values = x but not P values < x;
  - definition of ‘center values’ as median or average;
  - definition of error bars as s.d. or s.e.m.

Please complete ALL of the questions below.
Select "Not Applicable" only when the requested information is not relevant for your study.

Materials

Newly Created Materials	Information included in the manuscript?	In which section is the information available? (Reagents and Tools Table, Materials and Methods, Figures, Data Availability Section)
New materials and reagents need to be available; do any restrictions apply?	Not Applicable	

Antibodies	Information included in the manuscript?	In which section is the information available? (Reagents and Tools Table, Materials and Methods, Figures, Data Availability Section)
For **antibodies** provide the following information:<br>- Commercial antibodies: RRID (if possible) or supplier name, catalogue number and or/clone number<br>- Non-commercial: RRID or citation	Not Applicable	

DNA and RNA sequences	Information included in the manuscript?	In which section is the information available? (Reagents and Tools Table, Materials and Methods, Figures, Data Availability Section)
**Short novel DNA or RNA including primers, probes**: provide the sequences.	Not Applicable	

Cell materials	Information included in the manuscript?	In which section is the information available? (Reagents and Tools Table, Materials and Methods, Figures, Data Availability Section)
**Cell lines:** Provide species information, strain. Provide accession number in repository **OR** supplier name, catalog number, clone number, and/**OR** RRID.	Not Applicable	
**Primary cultures:** Provide species, strain, sex of origin, genetic modification status.	Not Applicable	
Report if the cell lines were recently **authenticated** (e.g., by STR profiling) and tested for mycoplasma contamination.	Not Applicable	

Experimental animals	Information included in the manuscript?	In which section is the information available? (Reagents and Tools Table, Materials and Methods, Figures, Data Availability Section)
**Laboratory animals or Model organisms:** Provide species, strain, sex, age, genetic modification status. Provide accession number in repository **OR** supplier name, catalog number, clone number, **OR** RRID.	Not Applicable	
**Animal observed in or captured from the field:** Provide species, sex, and age where possible.	Not Applicable	
Please detail **housing and husbandry conditions**.	Not Applicable	

Plants and microbes	Information included in the manuscript?	In which section is the information available? (Reagents and Tools Table, Materials and Methods, Figures, Data Availability Section)
**Plants:** provide species and strain, ecotype and cultivar where relevant, unique accession number if available, and source (including location for collected wild specimens).	Not Applicable	
**Microbes:** provide species and strain, unique accession number if available, and source.	Not Applicable	

Human research participants	Information included in the manuscript?	In which section is the information available? (Reagents and Tools Table, Materials and Methods, Figures, Data Availability Section)
If collected and within the bounds of privacy constraints report on age, sex and gender or ethnicity for all study participants.	Not Applicable	

Core facilities	Information included in the manuscript?	In which section is the information available? (Reagents and Tools Table, Materials and Methods, Figures, Data Availability Section)
If your work benefited from core facilities, was their service mentioned in the acknowledgments section?	Not Applicable	

Design

Study protocol	Information included in the manuscript?	In which section is the information available? (Reagents and Tools Table, Materials and Methods, Figures, Data Availability Section)
If study protocol has been **pre-registered, provide DOI in the manuscript**. For clinical trials, provide the trial registration number **OR** cite DOI.	Not Applicable	
Report the **clinical trial registration number** (at ClinicalTrials.gov or equivalent), where applicable.	Not Applicable	

Laboratory protocol	Information included in the manuscript?	In which section is the information available? (Reagents and Tools Table, Materials and Methods, Figures, Data Availability Section)
Provide DOI OR other citation details if **external detailed step-by-step protocols** are available.	Not Applicable	

Experimental study design and statistics	Information included in the manuscript?	In which section is the information available? (Reagents and Tools Table, Materials and Methods, Figures, Data Availability Section)
Include a statement about **sample size** estimate even if no statistical methods were used.	Not Applicable	
Were any steps taken to minimize the effects of subjective bias when allocating animals/samples to treatment (e.g. **randomization procedure**)? If yes, have they been described?	Not Applicable	
Include a statement about **blinding** even if no blinding was done.	Not Applicable	
Describe **inclusion/exclusion criteria** if samples or animals were excluded from the analysis. Were the criteria pre-established?<br><br>If sample or data points were omitted from analysis, report if this was due to attrition or intentional exclusion and provide justification.	Not Applicable	
For every figure, are **statistical tests** justified as appropriate? Do the data meet the assumptions of the tests (e.g., normal distribution)? Describe any methods used to assess it. Is there an estimate of variation within each group of data? Is the variance similar between the groups that are being statistically compared?	Yes	Results, Methods, Figure legends

Sample definition and in-laboratory replication	Information included in the manuscript?	In which section is the information available? (Reagents and Tools Table, Materials and Methods, Figures, Data Availability Section)
In the figure legends: state number of times the experiment was **replicated** in laboratory.	Not Applicable	
In the figure legends: define whether data describe **technical or biological replicates**.	Not Applicable	

Ethics

Ethics	Information included in the manuscript?	In which section is the information available? (Reagents and Tools Table, Materials and Methods, Figures, Data Availability Section)
Studies involving **human participants**: State details of **authority granting ethics approval** (IRB or equivalent committee(s), provide reference number for approval.	Not Applicable	
Studies involving **human participants**: Include a statement confirming that **informed consent** was obtained from all subjects and that the experiments conformed to the principles set out in the WMA Declaration of Helsinki and the Department of Health and Human Services Belmont Report.	Not Applicable	
Studies involving **human participants:** For publication of **patient photos**, include a statement confirming that consent to publish was obtained.	Not Applicable	
Studies involving experimental **animals**: State details of **authority granting ethics approval** (IRB or equivalent committee(s), provide reference number for approval. Include a statement of compliance with ethical regulations.	Not Applicable	
Studies involving **specimen and field samples:** State if relevant **permits** obtained, provide details of authority approving study; if none were required, explain why.	Not Applicable	

Dual Use Research of Concern (DURC)	Information included in the manuscript?	In which section is the information available? (Reagents and Tools Table, Materials and Methods, Figures, Data Availability Section)
Could your study fall under dual use research restrictions? Please check biosecurity documents and list of **select agents and toxins** (CDC): https://www.selectagents.gov/sat/list.htm	Not Applicable	
If you used a select agent, is the security level of the lab appropriate and reported in the manuscript?	Not Applicable	
If a study is subject to dual use research of concern regulations, is the name of the **authority granting approval and reference number** for the regulatory approval provided in the manuscript?	Not Applicable	

Reporting

The MDAR framework recommends adoption of discipline-specific guidelines, established and endorsed through community initiatives. Journals have their own policy about requiring specific guidelines and recommendations to complement MDAR.

Adherence to community standards	Information included in the manuscript?	In which section is the information available? (Reagents and Tools Table, Materials and Methods, Figures, Data Availability Section)
State if relevant guidelines or checklists (e.g., **ICMJE, MIBBI, ARRIVE, PRISMA**) have been followed or provided.	Not Applicable	
For **tumor marker prognostic studies,** we recommend that you follow the **REMARK** reporting guidelines (see link list at top right). See author guidelines, under 'Reporting Guidelines'. Please confirm you have followed these guidelines.	Not Applicable	
For **phase II and III randomized controlled trials**, please refer to the **CONSORT** flow diagram (see link list at top right) and submit the CONSORT checklist (see link list at top right) with your submission. See author guidelines, under 'Reporting Guidelines'. Please confirm you have submitted this list.	Not Applicable	

Data Availability

Data availability	Information included in the manuscript?	In which section is the information available? (Reagents and Tools Table, Materials and Methods, Figures, Data Availability Section)
Have **primary datasets** been deposited according to the journal's guidelines (see 'Data Deposition' section) and the respective accession numbers provided in the Data Availability Section?	Yes	Data Availability Section
Were **human clinical and genomic datasets** deposited in a public access-controlled repository in accordance to ethical obligations to the patients and to the applicable consent agreement?	Not Applicable	
Are **computational models** that are central and integral to a study available without restrictions in a machine-readable form? Were the relevant accession numbers or links provided?	Yes	Data Availability Section
If publicly available data were reused, provide the respective **data citations in the reference list**.	Yes	
